# Using chirality to influence supramolecular gelation†
†Electronic supplementary information (ESI) available. See DOI: 10.1039/c9sc02239b


**DOI:** 10.1039/c9sc02239b

**Published:** 2019-07-03

**Authors:** Kate McAulay, Bart Dietrich, Hao Su, Michael T. Scott, Sarah Rogers, Youssra K. Al-Hilaly, Honggang Cui, Louise C. Serpell, Annela M. Seddon, Emily R. Draper, Dave J. Adams

**Affiliations:** a School of Chemistry , University of Glasgow , Glasgow , G12 8QQ , UK . Email: dave.adams@glasgow.ac.uk; b Department of Chemical and Biomolecular Engineering , Whiting School of Engineering , Johns Hopkins University , 3400 North Charles Street , Baltimore , MD 21218 , USA; c ISIS Pulsed Neutron Source , Rutherford Appleton Laboratory , Didcot , OX11 0QX , UK; d School of Life Sciences , University of Sussex , Falmer , UK; e Chemistry Department , College of Science , Mustansiriyah University , Baghdad , Iraq; f School of Physics , HH Wills Physics Laboratory , University of Bristol , Tyndall Avenue , Bristol , BS8 1TL , UK; g Bristol Centre for Functional Nanomaterials , HH Wills Physics Laboratory , University of Bristol , Tyndall Avenue , Bristol , BS8 1TL , UK

## Abstract

Most low molecular weight gelators are chiral, with racemic mixtures often unable to form gels. Here, we show an example where all enantiomers, diastereomers and racemates of a single functionalized dipeptide can form gels. At high pH, different self-assembled aggregates are formed and these directly template the structures formed in the gel. Hence, solutions and gels with different properties can be accessed simply by varying the chirality. This opens up new design rules for the field.

## Abstract 2

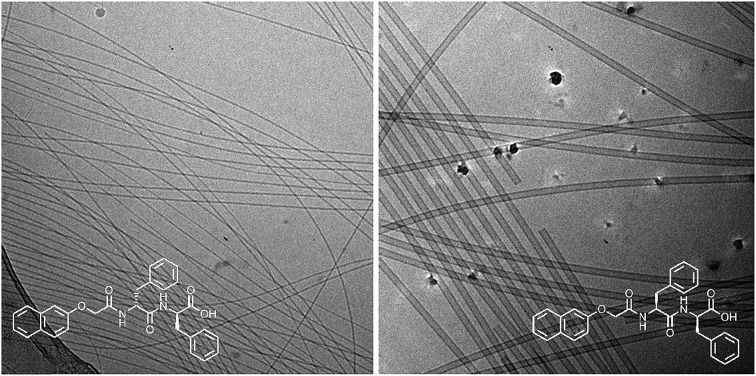
Different self-assembled structures can be formed by varying the chirality of a functionalised dipeptide allowing gels with different properties to be prepared.

## Introduction

Low molecular weight gels are formed when a small molecule self-assembles into structures that entangle and immobilise the solvent.^1^ There are many classes of compound that can be effective gelators.^2^ Whilst chirality is not a pre-requisite for a molecule to be an effective gelator,^3^ many gelators are chiral and in these cases it is most common that the enantiomers are usually equally effective as gelators.^4^,^5^ However, the racemic mixtures are typically poor gelators if any gel is formed at all.^6^,^7^ It is rare for a racemate to be more effective than the chiral analogues.^8^–^11^

Gels in organic solvents are usually formed by a thermal trigger. A suspension of the gelator is heated until complete dissolution occurs. Cooling leads to gelation. Hence, assembly occurs from freely dissolved molecule to self-assembled structures. In water, the situation is often more complicated. Thermal triggers can again be used, but other triggers are often used. A common example is to use a pH change to induce gelation; the molecule is dispersed in water at one pH, but the solubility is markedly decreased (leading to self-assembly into structures that entangle to form a gel) at a different pH. Even at the pH where the molecules are dispersed, they may be surfactant-like in nature, meaning self-assembly occurs at both pH values, but into different structures. Hence the gelator is never fully dissolved.

There are many examples of highly efficient hydrogelators based on oligopeptides.^12^ In general, one enantiomer is used. There are a small number of examples where this is not the case. Marchesan *et al.* showed that changing the chirality in the N-terminus of a tripeptide could result in a non-gelator becoming a gelator, with for example VFF becoming a gelator when the d-isomer of the terminal valine was used.^13^,^14^ In rare examples, a mixture of two enantiomers has been shown to lead to gels with improved properties.^9^,^15^,^16^

An effective class of gelator is that of dipeptides functionalized with a range of aromatic groups.^5^,^17^ Again, the majority of these are chiral, and the chirality is important. For example, Yang *et al.* reported naphthalene-dipeptides that formed gels on reducing the pH.^18^ Both the l-and d-versions were equally efficient gelators, but the racemic mixture was found to not be able to form gels. The chirality of the amino acid was found to lead to the fibres formed being left-handed or right-handed. Similarly, inversion of handedness has been observed for dialanines when the chirality was changed.^19^ In a rare example of examining a racemate, Wang *et al.* showed that the racemate of a substituted cyclo(Glu–Glu) could form gels and that these were able to recover more quickly than either pure enantiomer.^20^

This class of gelator is very interesting. Most commonly the terminal carboxylic acid is used to drive the gelation. At high pH, the carboxylate is sufficient to allow the molecules to be dispersed effectively. On decreasing the pH, protonation of the carboxylic acid leads to a significant decrease in the solubility and gelation can occur.^18^ At high pH, micellar aggregates are formed.^21^ If the molecule is not extremely hydrophobic, these aggregates tend to be spherical in nature, and not persistent. For example, NMR spectroscopy is generally able to measure the expected concentration, although structures can be detected by light and small-angle scattering.^22^ We have interpreted this as being due to the molecules being in equilibrium with the micellar aggregates, as would be the case for most conventional surfactants. When the molecules are more hydrophobic, worm-like aggregates are formed instead.^21^,^23^ This results in only a fraction of the molecules being detected by NMR spectroscopy, implying that the equilibrium is such that the molecules spend most of the time in the aggregate. Small angle scattering shows the presence of anisotropic structures, and the solutions tend to be highly viscous.^22^ Interestingly, gels can be formed by lowering the pH of both types of solution, implying that there are either two mechanisms by which self-assembly leads to gels, or that the self-assembled structures at high pH do not direct the gelation mechanism.

To date, the field has mainly focused on dipeptides based on the naturally occurring amino acids. As mentioned, almost all examples of this class that form gels also use the l-amino acids. d-amino acids are used in some specific cases to reduce the susceptibility of the dipeptide to enzymatic degradation.^24^ To the best of our knowledge, there have been no reports of gels formed from racemic functionalized-dipeptides.

## Results and discussion

**Fig. 1:**
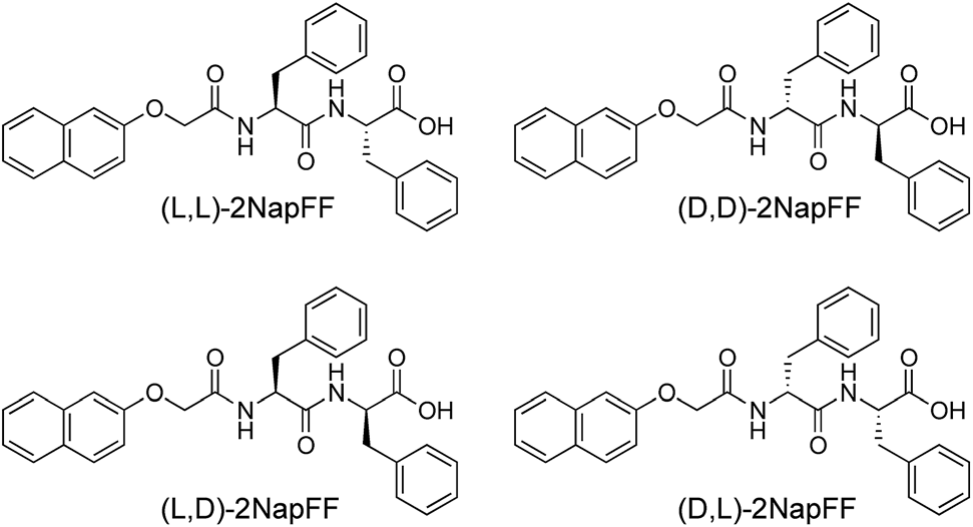
The different gelators used here. In addition, (rac)-2NapFF was prepared from racemic phenylalanine, and contains all of the (l,l)-, (d,d)-, (l,d)- and (d,l)-2NapFF. (mix)-2NapFF was prepared by mixing (l,l)-2NapFF and (d,d)-2NapFF.

Here, we focus on different enantiomers and diastereomers of the gelator 2NapFF. We have extensively discussed the self-assembly and gelation of 2NapFF previously, but always focused on the dipeptide prepared from the l-amino acids. This (l,l)-2NapFF forms worm-like micelles at high pH and translucent gels when the pH is decreased.^21^,^22^ In this study, we prepared (l,l)-2NapFF, (d,d)-2NapFF, (l,d)-2NapFF, (d,l)-2NapFF and (rac)-2NapFF, where the (rac)-2NapFF was prepared using racemic phenylalanine (Fig. 1). As such, the (rac)-2NapFF should (and does) contain all of the enantiomers and diastereomers (see Fig. S73†). From these, we further prepared (mix)-2NapFF by simply mixing an equal ratio of the (l,l)-2NapFF and the (d,d)-2NapFF.

**Fig. 2:**
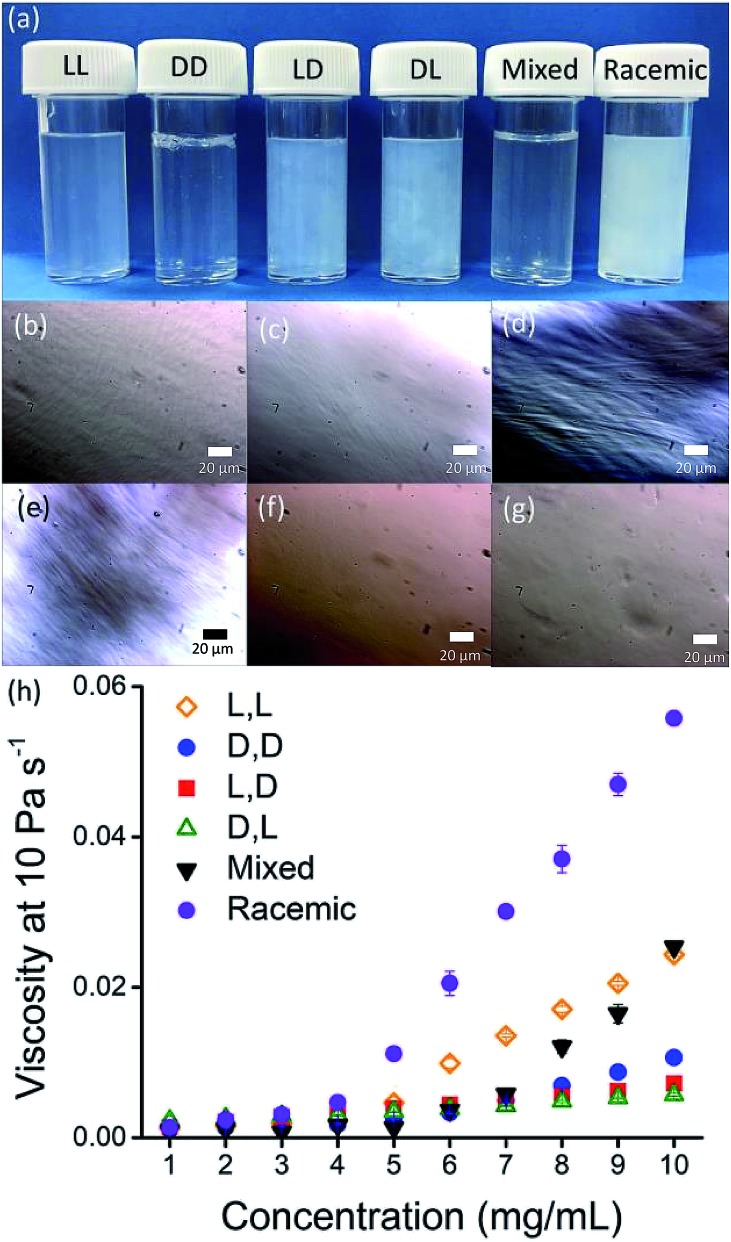
(a) Photographs of solutions of 2NapFF, from left to right (l,l)-, (d,d)-, (l,d)-, (d,l)-, (mix)- and (rac)-2NapFF with corresponding polarised optical microscopy for (b) (l,l)-; (c) (d,d)-; (d) (l,d)-; (e) (l,d)-; (f) (mix)-; (g) (rac)-2NapFF; (h) viscosity data for 2NapFF solutions.

Solutions were prepared from all of the enantiomers and diastereomers of 2NapFF at a concentration of 10 mg mL^–1^ and at a pH of 11 (Fig. 2a). (l,d)- and (d,l)-2NapFF solutions show the presence of birefringence by polarised optical microscopy (Fig. 2b). All form viscous solutions at high pH, which is indicative of the formation of worm-like micelles. The viscosity increases with concentration as expected (Fig. 2c). Interestingly, in some cases, there is a change in gradient, perhaps implying a change in micellar phase. The absolute viscosities depend on the system. The (rac)-2NapFF system is significantly more viscous than the others. Interestingly, the viscosities of the (l,l)- and (d,d)-2NapFF are not identical.

**Fig. 3:**
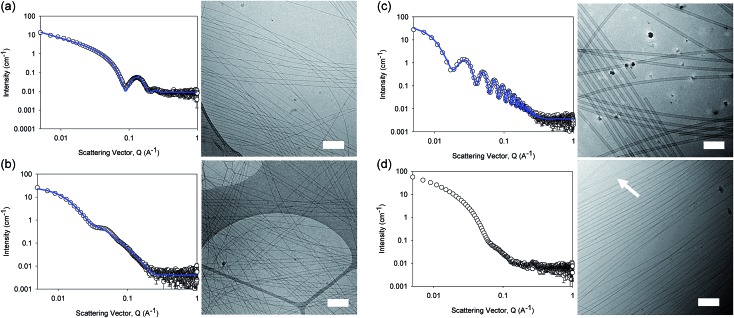
SANS scattering and cryo-TEM images for solutions at high pH. (a) (d,d)-2NapFF; (b) (mix)-2NapFF; (c) (l,d)-2NapFF; (d) (rac)-2NapFF. In all cases, the black circles represent the SANS data and the blue lines show the fit to the data. For the cryo-EM, the scale bar represents 200 nm. The arrow in (d) highlights a thin-walled nanotube.

The differences in viscosity imply that there are different structures present in solution. This was verified by small angle neutron scattering (SANS) and cryo-TEM. The fits to the SANS data show that (d,d)-2NapFF forms long, worm-like hollow tubes at high pH with an overall radius of 3.6 ± 0.1 nm, and a wall thickness of 1.9 ± 0.1 nm (Fig. 3a and Table S1†). As expected (l,l)-2NapFF forms very similar structures, in agreement with our previous studies (Fig. S3a†).^21^,^22^ The cryo-TEM for the (l,l)-2NapFF (Fig. S4†) and (d,d)-2NapFF (Fig. 3a) shows the presence of long wormlike structures in agreement with our previous data for (l,l)-2NapFF. As such, the differences in viscosity suggest perhaps a slight difference in persistence length between the structures which is not picked up by the fit.

Whilst (mix)-2NapFF was formed by simply mixing solutions of (l,l)-2NapFF and (d,d)-2NapFF, the scattering data are very different to either of the two components (Fig. 3b). This implies that this mixture does not simply contain a self-sorted mixture of structures formed from the two enantiomers,^25^ but rather a distinct new structure is formed.

The best fit to the data is again a hollow cylinder with an overall radius of 5.3 ± 0.1 nm, and a wall thickness of 2.2 ± 0.1 nm (although a polydispersity in radius of 0.5 has to be included to obtain an appropriate goodness of fit to the data). The cryo-TEM shows structures in agreement with these data (Fig. 3b). The scattering data for the (l,d)-2NapFF and (d,l)-2NapFF are very similar to one another, but very different to all of the other systems (Fig. 3c and S3†). The fits to the data for (l,d)-2NapFF and (d,l)-2NapFF imply that these are long, thin walled nanotubes with a diameter of 13.4 nm, and a wall thickness of 1.3 nm. Cryo-TEM shows that the structures formed are indeed thin-walled nanotubes (Fig. 3c and S9†). Finally, the scattering data for (rac)-2NapFF is also different to that of both (l,l)- and (d,d)-2NapFF (Fig. 3d). The data were not fitted on the basis of the cryo-TEM, since the images show a clear co-existence of species with different morphologies. Both thinner structures as in (l,l)-, (d,d)-, and (mix)-2NapFF are present, alongside thin-walled nanotubes as for (l,d)- and (d,l)-2NapFF (highlighted by an arrow in Fig. 3d).

Our interpretation of all of this data is that the molecules are acting as surfactants at high pH, with the structures adopted being affected by the chirality of the molecule. The molecular packing will be different depending on the chirality and hence the self-assembled structures formed will be likewise different. In the (rac)-2NapFF, mixing is clearly not preferential and so co-existing structures are formed.

**Fig. 4:**
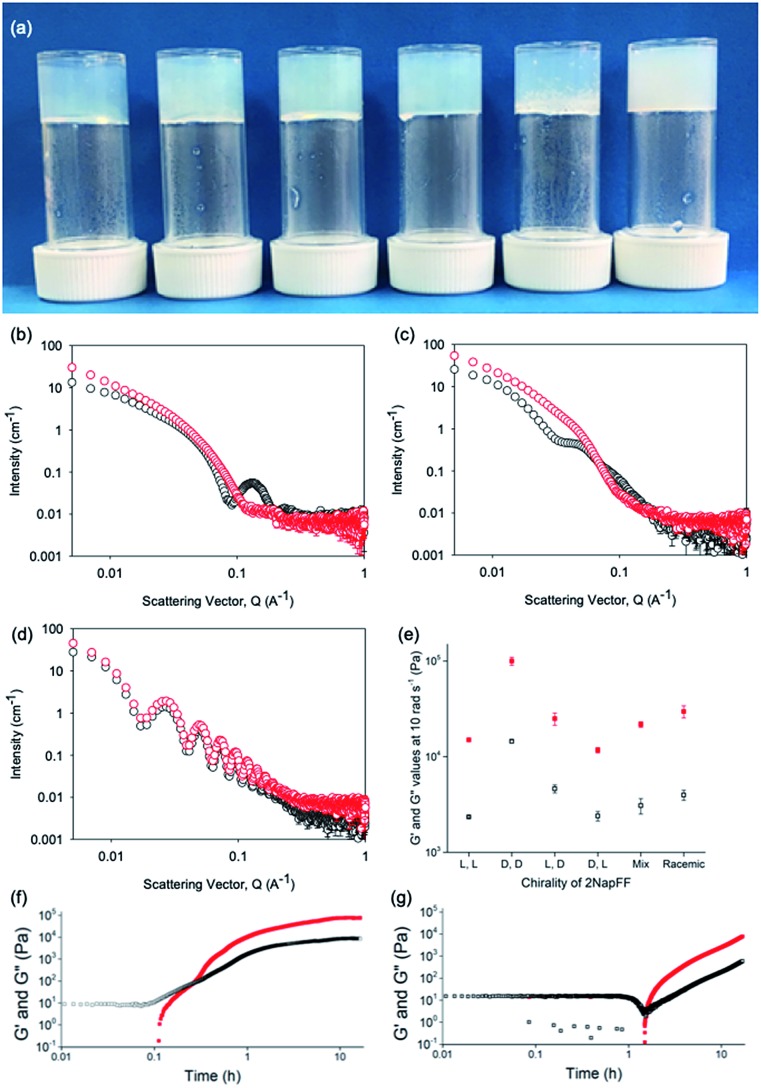
(a) Photographs of gels formed from (left to right) (l,l)-, (d,d)-, (l,d)-, (d,l)-, (mix)- and (rac)-2NapFF. Air bubbles can be seen in the mixed system arising from swirling the viscous solution to mix with GdL; (b–d) Comparison of SANS data for (l,l)-2NapFF (b), (mix)-2NapFF (c) and (l,d)-2NapFF (d) in the solution state (black) and gel state (red). (e) Rheological properties of the final gels from the different 2NapFF; (f) time sweep for gelation of (l,l)-2NapFF after addition of GdL. (g) Time sweep for gelation of (l,d)-2NapFF after addition of GdL. For (d–f), G′ in red and G′′ in black.

Typically, gels can be formed from such dipeptides by lowering the pH of the system. The current understanding of these dipeptide systems is that only the chiral examples will form gels, with racemic mixtures forming precipitates.^18^ We have previously shown that (l,l)-2NapFF forms gels when the pH of the solution is decreased.^22^ To achieve homogeneous and reproducible gels, we used the slow hydrolysis of glucono-δ-lactone (GdL) to bring about the pH change.^26^,^27^ All of the systems examined here form gels (Fig. 4a). This is surprising, as for other naphthalene-dipeptides, Yang *et al.* have shown that the mixtures of enantiomers do not form gels.^18^

The structures that lead to the gel network were again probed by SANS (cryo-TEM was also attempted, but the gels were sufficiently robust to make imaging difficult; note, we have shown that drying leads to artefacts in imaging^28^ so no attempt to collect SEM or TEM data was made).

From the SANS data, the (l,l)- and (d,d)-2NapFF lose the signature of the hollow core (Fig. 4a and S12†), and the data are best fitted to a flexible elliptical cylinder with a radius of around 3.0 nm and an axis ratio of around 2 (Fig. S13 and Table S2†). Examination of the Kuhn lengths obtained from these global fits shows that the (l,l)-2NapFF is significantly more flexible than the (d,d)-2NapFF. Interestingly, the rheological data shows that the (d,d)-2NapFF is a much stronger gel than that of the (l,l)-2NapFF (see below). There is no immediate reason as to why changing the chirality should have such a dramatic effect on the gel properties. The SANS data show that hollow cylinders are formed at high pH, and elliptical cylinders at low pH. Our current hypothesis is that manner in which this transition occurs may not be the same for the two enantiomers, leading to different packing in the gel state, even if the packing is equal and opposite in the micellar state.

For the SANS data from these gels, we can consider the gradients on the log *I*(*q*) *vs. Q* plot. For (d,d)-2NapFF, there are two distinct gradients: 0.007 < *Q* < 0.031, which has a slope of –1.5, and at *Q* > 0.03, with a slope of –3.9. These values are also replicated in the log *I*(*q*) *vs. Q* plot of (l,l)-2NapFF (slope 1 = –1.6, slope 2 = –3.9). The value of the slope at lower *Q* is indicative of scattering of a polydisperse non-rigid rod network.^29^,^30^ The decay at higher *Q* is in agreement with a q^–4^ variation in intensity of the Porod region, indicating a sharp interface between the gel fibres and solvent.

As mentioned above, global fitting of the data for both the (l,l)- and (d,d)-2NapFF gel indicates a good fit to a flexible cylinder with an elliptical cross section. To confirm this, plots of ln(*Q*^*α*^*I*) *vs.* Q^2^ where *α* = 1 for a rigid rod, and 2 for a ribbon-like fibre with an elliptical (or rectangular) cross-section were examined. The best linear fit of the data was to a plot where *α* = 2. A value for the cross-sectional thickness of the scattering object (*t*) can be extracted from this data and showed that for the (d,d)-2NapFF gel, *t* = 5.3 nm. Based on the radius of the global fit, the thickness here was found to be 5.4 nm, which is in good agreement. Similarly for the (l,l)-2NapFF, *t* = 6.3 nm, with a diameter from the global fit found to be 6.0 nm. This confirms the elliptical nature of the fibres in the gels.

The SANS data for the gels formed from both the (mix)-2NapFF (Fig. 4b) and (rac)-2NapFF (Fig. S13†) could also both be fitted to a flexible elliptical cylinder model, with radii of 3.1 nm and 5.2 nm and an axis ratio of 1.7 and 1.6 respectively. The fit to the data for the (rac)-2NapFF is poorer as might be expected if there are still co-existing species. The same analysis as above was performed on the (rac)-2NapFF gel and (mix)-2NapFF gel. For the (rac)-2NapFF gel, *t* = 10.6 nm, corresponding well to the global fit which gave 10.2 nm. In the case of the (mix)-2NapFF gel, *t* = 6.8 nm, which compares to a value from the global fit of 6.1 nm. In all cases, prior to the linear region in the ln(*Q*^2^*I*) *vs. Q*^2^ plot there is a bump as *Q* tends to 0 which is suggestive of thicker bundles of fibres which form the gel network.

For (l,d)- and (d,l)-2NapFF, the SANS data for the structures present in the gel are very similar to those at high pH (Fig. 4c and S12†). This implies that these structures are protonated during gelation and presumably locked in. Hence, the structures formed at high pH determine those in the gel state and these can be controlled by the chirality of the dipeptide used. Further discussion of the SANS data is available in the ESI.†

The differences shown by the SANS data lead to differences in the properties of the final gels. For (l,l)-2NapFF, we have previously shown that there are two apparent p*K*_a_ values,^31^ which can be linked to changes in aggregation as the pH is decreased. Gels are formed below the lowest apparent p*K*_a_ (around pH 6).^22^ The apparent p*K*_a_ values found here are determined by the chirality of the dipeptide. The (d,l)- and (l,d)- have lower apparent p*K*_a_ (Fig. S16†), whilst the (rac)-2NapFF shows a very high first p*K*_a_ of around 9.5, and a second high apparent p*K*_a_ of around 7.5. These differences in apparent p*K*_a_ link directly to the pH at which gelation occurs. Using GdL to adjust the pH allows us to monitor the gelation with time. By following the gelation with time, it is clear that the gelators with the higher apparent p*K*_a_ start to gel at earlier times (Fig. S17†). For example, the (l,l)-2NapFF starts to gel at 15 minutes (Fig. 4e), whilst the (l,d)-2NapFF only starts to gel at 90 minutes (Fig. 4f). The final gels are all relatively similar in terms of absolute values of the storage (G′) and loss (G′′) moduli (Fig. 4d). As noted above, the (d,d)-2NapFF forms a stiffer gel than that of the (l,l)-2NapFF, which correlates with the SANS data.

**Fig. 5:**
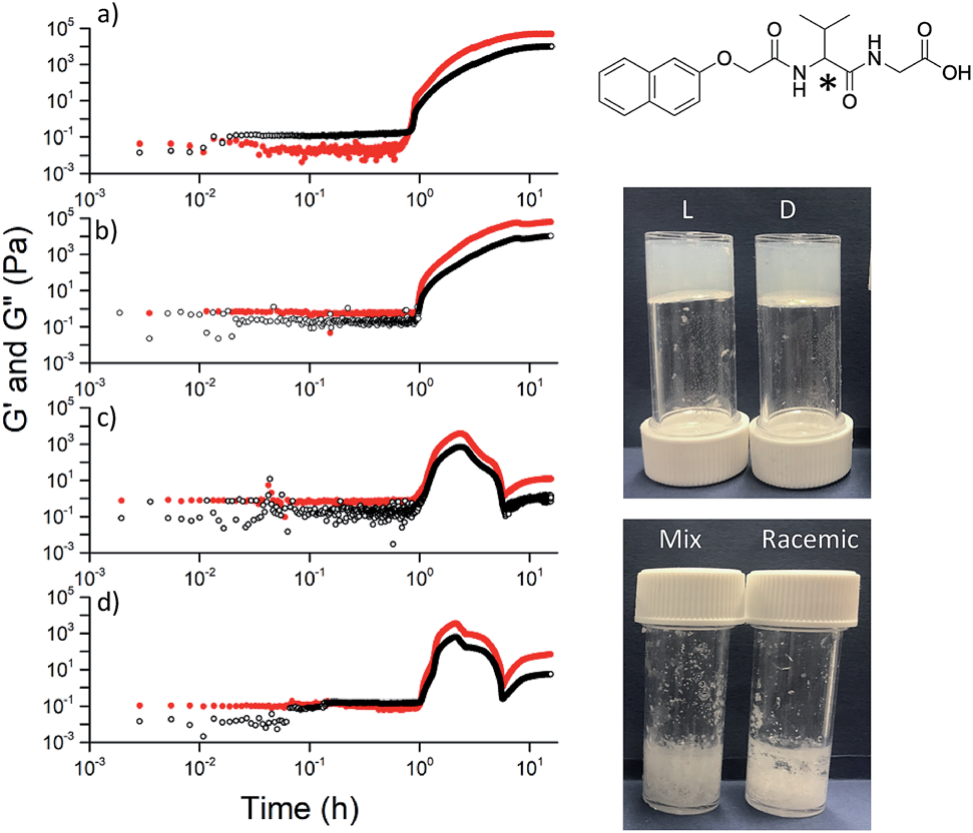
For dipeptides that do not form viscous solutions at high pH, only the individual enantiomers form gels when the pH is decreased. On the left, the rheology with time is shown for (a) (l)-; (b) (d)-; (c) (mix)-; (d) (rac)-2NapVG (structure top right with chiral centre shown by *). The photographs show the final materials after 18 hours.

The observation that gels can be formed from all of the versions is not restricted to 2NapFF. However, there is a necessity for the functionalized dipeptide to form anisotropic structures at high pH. For example, the functionalized dipeptide 2NapVG shown in Fig. 5 forms spherical structures at high pH and hence a low viscosity.^22^ We have previously shown that such dipeptides that do not form viscous solutions first undergo a transition from spherical micelles to worm-like structures as the pH is decreased, beginning at the apparent p*K*_a_. These then laterally associate and entangle to form the gel network.^32^ For this dipeptide, gels can be formed when decreasing the pH of either the (l)- or the (d)-enantiomer, but the racemic mixture (formed either from the racemic amino acid or by mixing enantiomers) does not result in a gel. Similar results are observed for other dipeptides that form spherical structures at high pH (Fig. S20, S21 and S22†). Instead, precipitation occurs, as expected from the observations of Yang *et al.*^18^ However, other dipeptides that form viscous solutions at high pH can result in gels using different chiralities (Fig. S23, S24 and S25†). Hence, if the functionalised dipeptide is to be able to form gels when the peptide sequence is achiral, it seems that there is the requirement for viscous solutions to be formed at high pH. Unlike the case of the dipeptides that form spherical micellar structures at high pH, where a transition from spheres to worm-like structures occurs and then gelation,^32^ those that form viscous solutions at high pH clearly already contain structures that can be locked in as the pH decreases and the charge is removed.

## Conclusions

We have shown that different self-assembled structures are formed at high pH from the possible enantiomers and diastereomers of a functionalized dipeptide in water. On decreasing the pH, gels are formed in all cases which is surprising considering the current literature in this field. Importantly, the gels are formed from structures that are very similar to those at high pH, showing that the initial aggregates template the gel structure. Until now, the assumption has been made that a single enantiomer is required for effective gelation. We have shown that this is not the case. This work opens up the possibility to tune self-assembled structure and gel properties by varying the chirality of the dipeptide.

## Conflicts of interest

There are no conflicts to declare.

## Supplementary Material

Supplementary information
