# Supplementary material for: Using chirality to influence supramolecular gelation
Source: Chem Sci. 2019 Jul 3;10(33):7801–6. doi: 10.1039/c9sc02239b (PMC6761870; doi:10.1039/c9sc02239b)
Supplement: Supplementary file 1 [file SC-010-C9SC02239B-s001.pdf]

Electronic Supplementary Material (ESI) for Chemical Science.
This journal is © The Royal Society of Chemistry 2019

Using Chirality to Influence Supramolecular Gelation

Kate McAulay,[a] Bart Dietrich,[a] Hao Su,[b] Michael T. Scott,[a] Sarah Rogers,[c] Youssra K. Al-Hilaly,[d,e] Honggang Cui,[b] Louise C. Serpell,[d] Annela M. Seddon,[f,g] Emily R. Draper[a] and Dave J. Adams[a,*]

a School of Chemistry, University of Glasgow, Glasgow, G12 8QQ, U.K.

Email: dave.adams@glasgow.ac.uk

b Department of Chemical and Biomolecular Engineering, Whiting School of Engineering, Johns Hopkins University, 3400 North Charles Street, Baltimore, MD 21218, USA

c ISIS Pulsed Neutron Source, Rutherford Appleton Laboratory, Didcot, OX11 0QX, U.K.

d School of Life Sciences, University of Sussex, Falmer, BN1 9QG, U.K.

e Chemistry Department, College of Science, Mustansiriyah University, Baghdad, Iraq

f School of Physics, HH Wills Physics Laboratory, Tyndall Avenue, University of Bristol, Bristol, BS8 1TL, U.K.

g Bristol Centre for Functional Nanomaterials, HH Wills Physics Laboratory, Tyndall Avenue, University of Bristol, Bristol, BS8 1TL, U.K.

Supporting Information

Methods

Gelators. Full synthetic details for the gelators can be found in the Supporting Information. All other chemicals were purchased from Sigma Aldrich, Alfa, TCI and Fluorochem.

Stock Solutions. Gelator stock solutions (5 or 10 mg/mL) were prepared in Falcon Tubes by suspending 500 mg of gelator in deionised water and adding one molar equivalent of sodium hydroxide solution (0.1 M, NaOH, Sigma Aldrich) such that the final volume was 50 mL. The solutions were stirred at 1200 rpm overnight to allow complete dissolution of the gelator. Next, the pH of each solution was measured and adjusted, if needed, to pH 11 ± 0.1 with either NaOH (1 M) or HCl (1 M).

In the case of 2NapFF solutions at lower concentration, aliquots of the original 10 mg/mL stock solutions were added to Sterilin vials and the overall volume of sample was made up to 1 mL by the addition of water at pH 11 (a mixture of deionized water and NaOH). Dilutions were performed using a 1 mL pipette tip to ensure the same shear history for each sample.

For SANS measurements solutions were prepared as described above, with the H_2O and NaOH replaced with D_2O and NaOD.

Gels. Gel samples were prepared in Sterilin vials by the addition of 2 mL stock solution to 3 molar equivalents of glucono-δ-lactone (GdL). The vials were gently rotated by hand to ensure completed dissolution of GdL and left to stand overnight quiescently. Rheology data was collected 18 hours after the addition of GdL.

For SANS measurements gel samples were prepared with the deuterated gelator solutions and GdL.

pH Measurements. pH measurements were performed using a FC200 pH probe (HANNA Instruments) with a 6 mm x 10 mm conical tip. The accuracy of the pH measurements is quoted as ± 0.1.

pK_a Titrations. The apparent pK_a values of 10 mg/mL 2NapFF solutions were determined via titration by the addition of aliquots of a 0.1 M HCl solution. The pH values were recorded after each addition of acid when a stable pH value was achieved. To prevent gel formation, the solutions were constantly stirred to ensure the sample was liquid throughout the entire experiment.

Rheology. Rheological measurements were carried out using Anton Paar Physica MCR301 and M101 Rheometers. For the frequency and strain sweeps, a cup and vane (ST10-4V-8.8/97.5-SN42404) system, with a measuring gap of 1.8 mm, was used so that measurements could be directly performed in the 7 mL Sterilin vials. Frequency sweeps were performed from 1 rad s^{-1} to 100 rad s^{-1} at a constant strain of 0.5 %. Strain weeps were performed from 0.1 %

to 1000 % at a frequency of 10 rad s^{-1}. This method ensured that 0.5 % strain was in the viscoelastic region required for measuring the frequency sweep.

For the 2NapFF time sweeps, measurements were performed using a cup and vane (ST10-4V-8.8/97.5-SN42404) system, with a measuring gap of 1.8 mm. 2 mL of 10 mg/mL stock solution was added to GdL in a Sterilin vial and measurements were performed directly in the vial. To prevent evaporation of the sample the top of the vial was covered with tissue saturated with deionized water then Al foil was added on top, while avoiding contact with the vane. For the other gelators 2 mL of the 10 mg/mL stock solutions were added to predetermined masses of GdL. The vial was gently swirled to ensure the complete dissolution of GdL. Next, 1 mL of the stock and GdL solution was pipetted on to the rheometer flat plat using a 1 mL pipette tip. Time sweep measurements were performed at 25 °C using a using a 50 mm sandblasted parallel plate and a measuring gap of 0.8 mm. A constant frequency of 10 rad s^{-1} and a strain of 0.5 % was applied. The storage (G') and loss (G") modulus were measured over time for 16 hours.

For the viscosity measurements, a concentration range of 1 – 10 mg/mL was studied. The samples were poured on to the rheometer plate to minimise shear effects. A 50 mm cone and plate system was used at 25°C. The gap distance between the geometry and lower flat plate was 0.1 mm. The viscosity of each solution was recorded with the rotation shear rate varying from 1 to 100 s^{-1}.

Circular Dichroism. CD spectra were collected using Jasco J-715 spectropolarimeter. 10 µL of the sample was placed into 0.01 mm demountable spectrosil quartz cuvette (Starna Optiglass, Hainault, UK) and the CD spectra were measured at 21 °C between 500 and 180 nm with a data pitch of 0.1 nm. The bandwidth was set to 1 nm with a scanning speed of 100 nm min^{-1} and a response time of 2 s.

Confocal Microscopy. A Zeiss LSM 710 confocal microscope was used to take confocal images. The objective used was a LD EC Epiplan NEUFLUAR 50x (0,55 DIC). The samples were stained with 2 µL/mL of a 0.1 wt % Nile Blue solution and excited using a HeNe laser at 634 nm. 1 mL samples were prepared in vials with GdL. After complete dissolution of GdL 400 µL of the sample was pipetted into the centre of the confocal dishes. Tissue saturated with deionized water was carefully wrapped inside the dish with no contact between the tissue and the sample, to ensure a humid environment to prevent evaporation of the sample. Next the confocal dish was sealed with parafilm and left overnight to gel before analysis.

Cryo-TEM. Cryogenic TEM imaging was performed using a FEI Tecnai 12 TWIN Transmission Electron Microscope, operating at 100 kV. 6 µL of the sample solution was placed on a holey carbon film supported on a TEM copper grid (Electron Microscopy Services,

Hatfield, PA). All the TEM grids used for cryo-TEM imaging were treated with plasma air to render the lacey carbon film hydrophilic. A thin film of the sample solution was produced using the Vitrobot with a controlled humidity chamber (FEI). After loading of the sample solution, the lacey carbon grid was blotted using pre-set parameters and plunged instantly into a liquid ethane reservoir precooled by liquid nitrogen. The vitrified samples were then transferred to a cryo-holder and cryo-transfer stage, which was cooled by liquid nitrogen. To prevent sublimation of vitreous water, the cryo-holder temperature was maintained below −170 °C during the imaging process. All images were recorded by a SIS Megaview III wide-angle CCD camera.

Optical Microscopy. Optical microscope images were collected using a Nikon Eclipse LV100 microscope with a Nikon Plan ELWD 50x/0.60 lens attached to an Infinity2-1C camera. Images were collected under polarised and non-polarised light.

Small Angle Neutron Scattering. SANS measurements of the gelator solutions and gel samples were performed using the SANS2D time-of-flight diffractometer (STFC ISIS Pulsed Neutron Source, Oxfordshire, UK). A simultaneous Q-range [$Q = 4\pi \sin(\theta/2)/\lambda$, where θ is the scattering angle] of 0.005 to 1.0 $Å^{-1}$ was achieved using an incident wavelength (λ) range of 1.75 to 16.5 Å and employing two 1 m^2 detectors. The small-angle detector was positioned 4 m from the sample and offset vertically 60 mm and sideways 100 mm. The wide-angle detector was positioned 2.4 m from the sample, offset sideways by 980 mm and rotated to face the sample. The incident neutron beam was collimated to 8 mm diameter. Samples were housed in 2 mm pathlength quartz cuvettes and measured for 60 minutes each. The 'raw' scattering data were normalised to the incident neutron wavelength distribution, corrected for the linearity and efficiency of the detector response and the measured neutron transmission (i.e., absorbance) using the Mantid framework.[32] They were then placed on an absolute scale by comparison with the expected scattering from a partially-deuterated polystyrene blend of known composition and molecular weights in accordance with established procedures.[33] The background scattering from a quartz cell containing D_2O was then subtracted. Data fitting is discussed on page S13.

1. Additional Figures

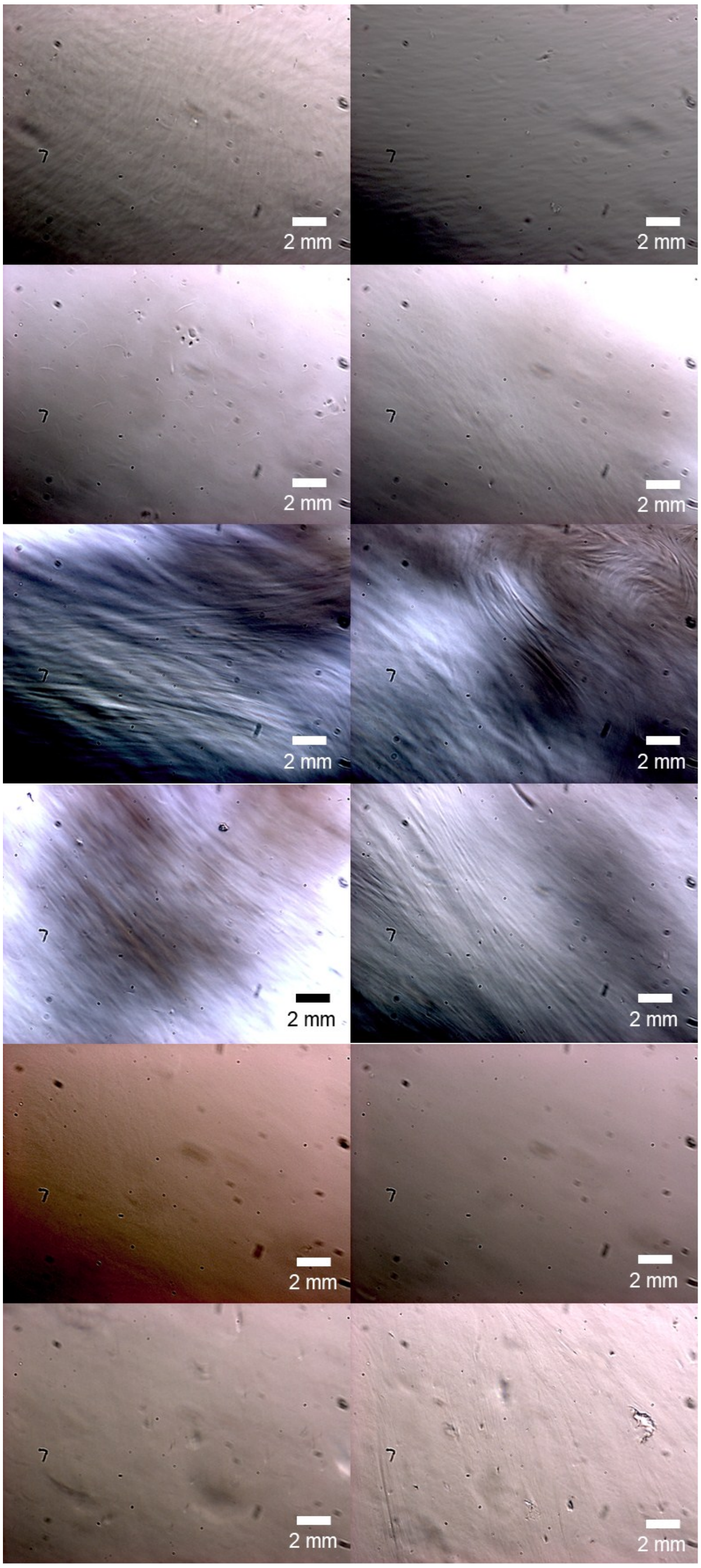

Figure S1. Further polarised optical microscope images of 2NapFF solutions at a concentration of 10 mg/mL at 50 x magnification. Top to bottom: (L,L)-, (D,D)-, (L,D)-, (D,L)-, (mix), and (rac)-2NapFF.

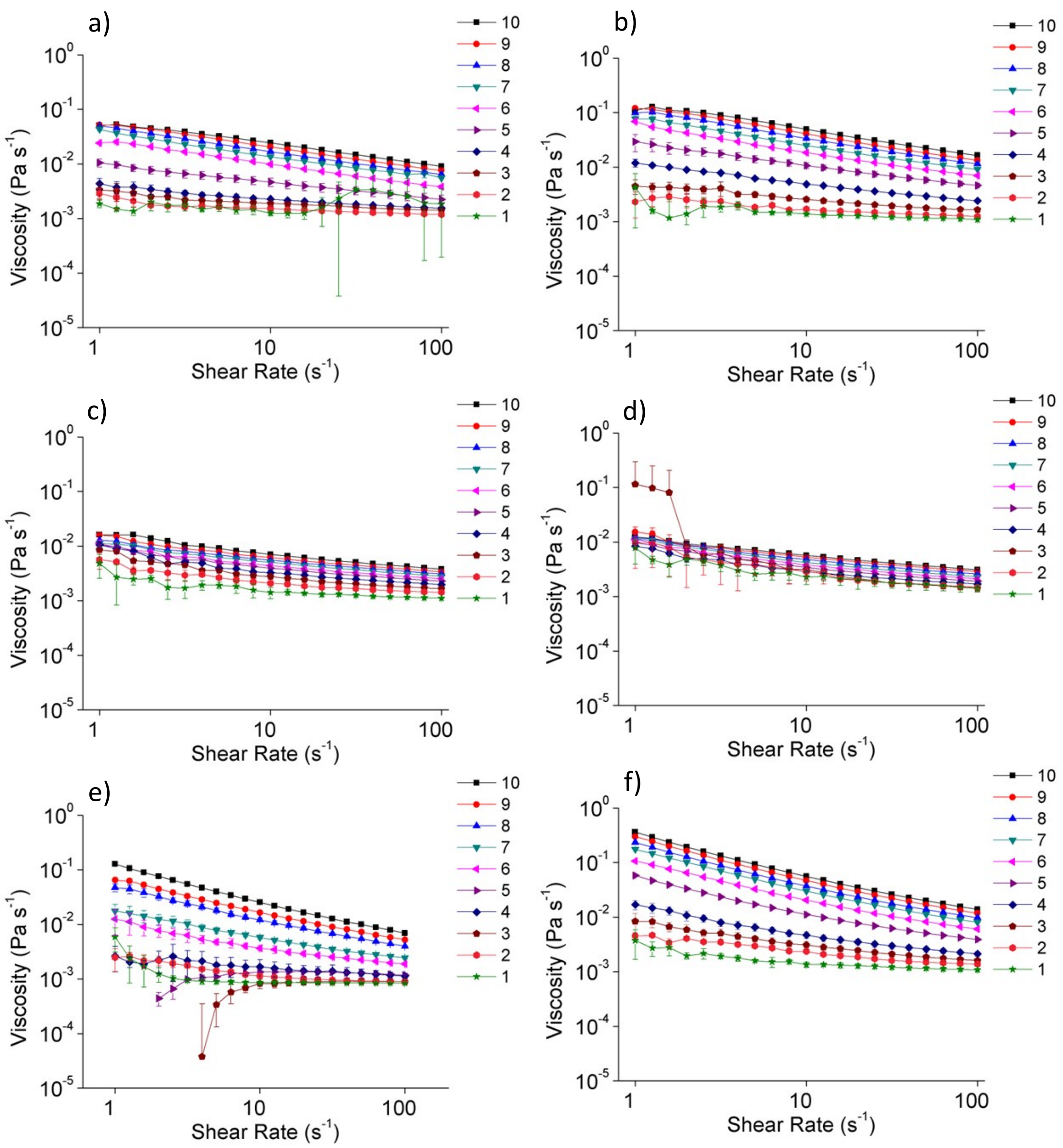

Figure S2. Viscosity measurements for 1-10 mg/mL 2NapFF solutions at pH 11 (a) (L,L)-; (b) (D,D)-; (c) (L,D)-; (d) (D,L)-; (e) (mix)-; (f) (rac)-2NapFF. Three repeats of each sample were measured, with the average plotted with error bars as standard deviations. The numbers indicate the concentration of 2NapFF solutions.

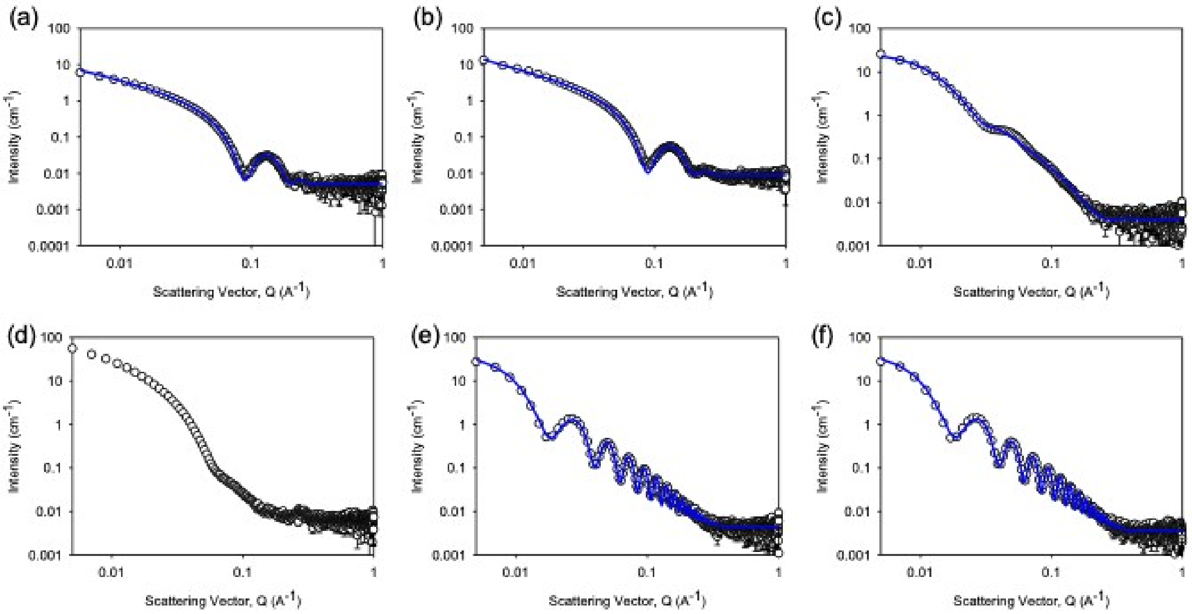

Figure S3. SANS scattering for (a) (L,L)-2NapFF; (b) (D,D)-2NapFF; (c) (mix)-2NapFF; (d) (rac)-2NapFF; (e) (L,D)-2NapFF; (f) (D,L)-2NapFF. In all cases, the black circles represent the SANS data and the blue lines show the fit to the data.

	**(L,L)-2NapFF**	**(D,D)-2NapFF**	**(L,D)-2NapFF**	**(D,L)-2NapFF**	**(mix)-2NapFF**	**(rac)-2NapFF**
Background (cm^{-1})	0.0051 ± $6.25x10^{-5}$	0.0088 ± $6.48x10^{-5}$	0.0044 ± $5.88x10^{-5}$	0.0036 ± $4.73x10^{-5}$	0.004*	
Scale	$3.08x10^{-3}$ ± $6.26x10^{-4}$	$5.49x10^{-3}$ ± $2.39x10^{-5}$	$5.20x10^{-3}$ ± $5.72x10^{-5}$	$5.36x10^{-3}$ ± $5.16x10^{-5}$	$4.85x10^{-3}$ ± $2.24x10^{-5}$	
Radius (Å)	16.6 ± 0.1	17.0 ± 0.1	133.8 ± 0.1	133.8 ± 0.1	53.2 ± 0.1	
Thickness (Å)	19.7 ± 0.2	19.1 ± 0.1	13.7 ± 0.2	13.8 ± 0.1	22.0 ± 0.1	
Length (Å)	810 ± 37	>1000	560.9 ± 5.54	569.3 ± 5.6	436.6 ± 3.7	
Polydispersity in radius					0.5*	
χ^2	1.8359	4.8173	4.4679	4.8742	7.0457	

Table S1. Summary of fits to the SANS data for the solutions of the different 2NapFF. Each data set was fitted using a hollow cylinder model. No fit was attempted for the (rac)-2NapFF (see main text). For (mix)-2NapFF, a fixed polydispersity in radius of 0.5 was chosen on the basis of quality of fit to the data.

For the (L,D)- and (D,L)-2NapFF, the fits to a hollow cylinder gave the lowest χ^2 values. However, the lengths are surprisingly short and do not agree with the observations by cryo-

TEM of long structures (see below). A satisfactory fit could also be achieved to a core shell model (where the core SLD is set to the SLD of the solvent). In these cases, the χ^2 values were less good, but the fits imply that the structures have a reasonable length. In both cases however the values for the radius and thickness were very similar. Hence, we interpret the length from the fit to the hollow cylinder as being the Kuhn length.

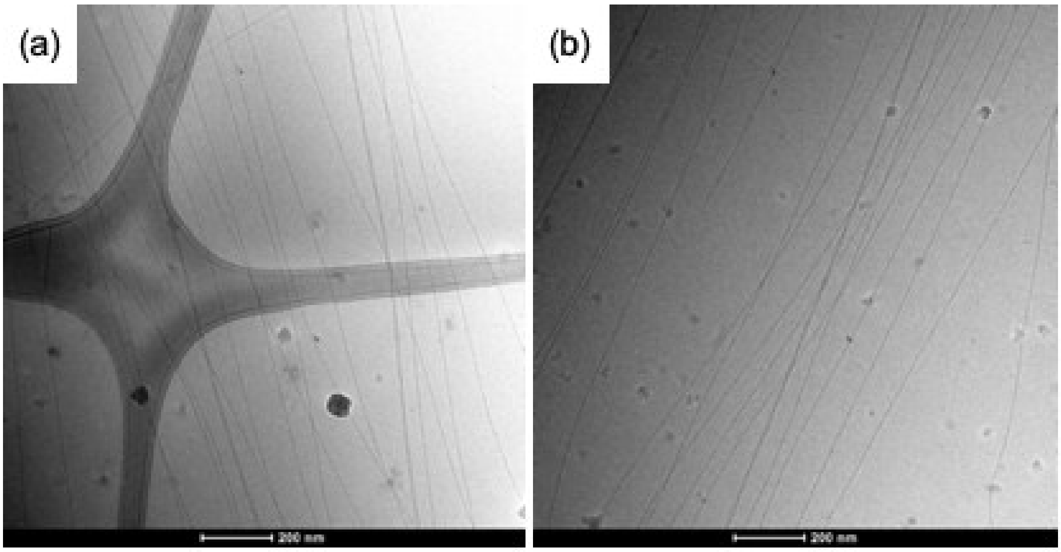

Figure S4. Cryo-TEM images of (L,L)-2NapFF. The scale bar represents 200 nm in both cases.

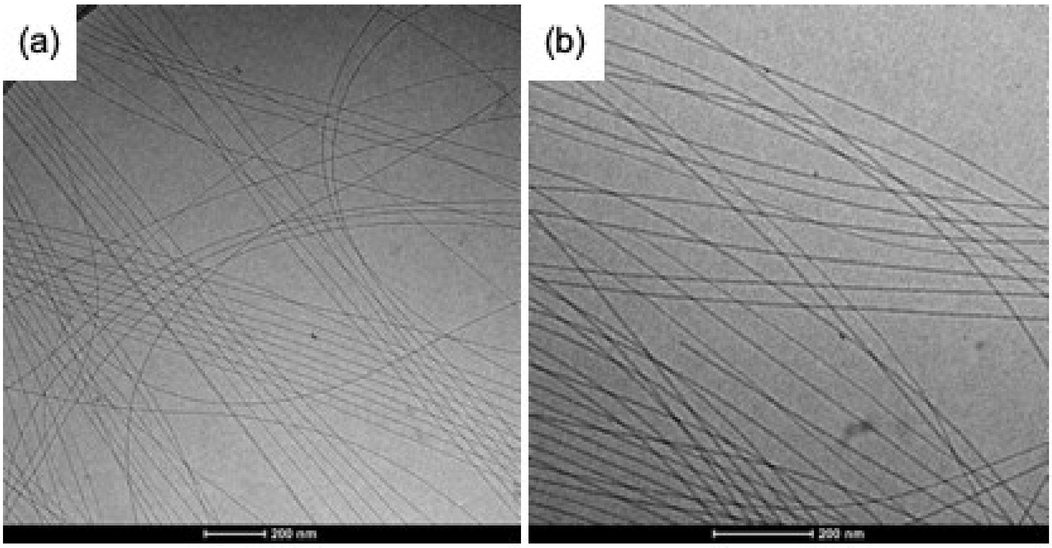

Figure S5. Cryo-TEM images of (D,D)-2NapFF. The scale bar represents 200 nm in both cases.

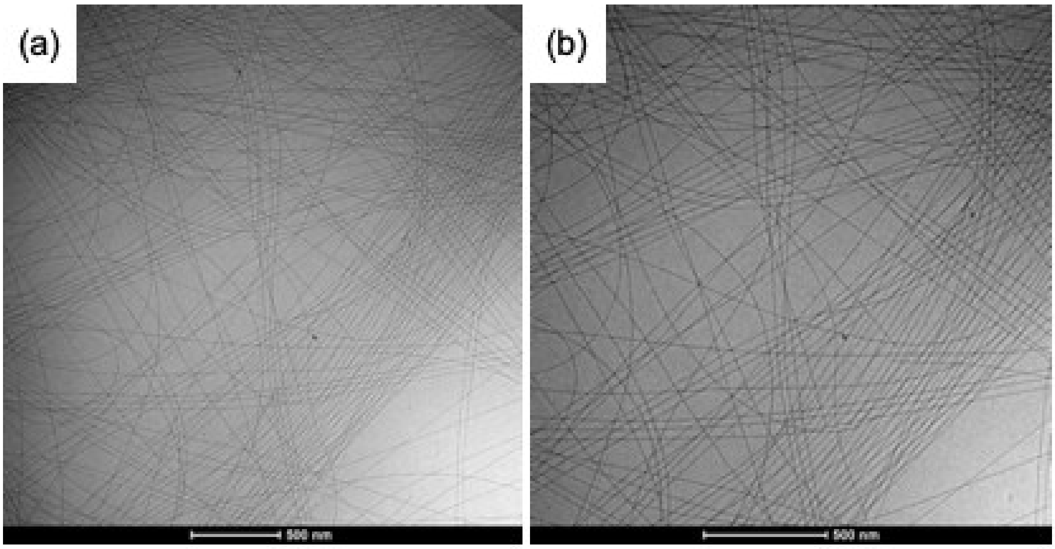

Figure S6. Cryo-TEM images of (mix)-2NapFF. The scale bar represents 500 nm in both cases.

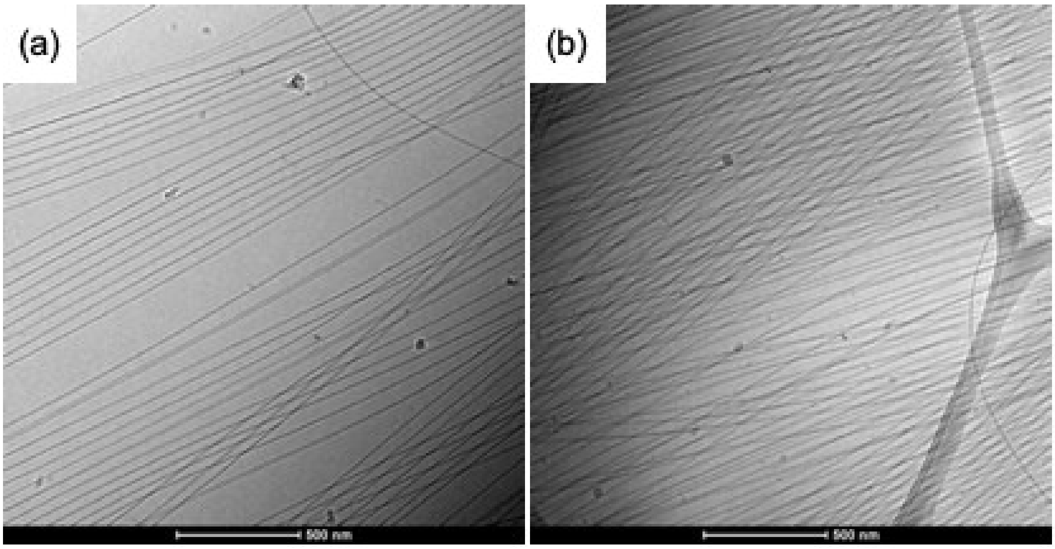

Figure S7. Cryo-TEM images of (rac)-2NapFF. The scale bar represents 500 nm in both cases.

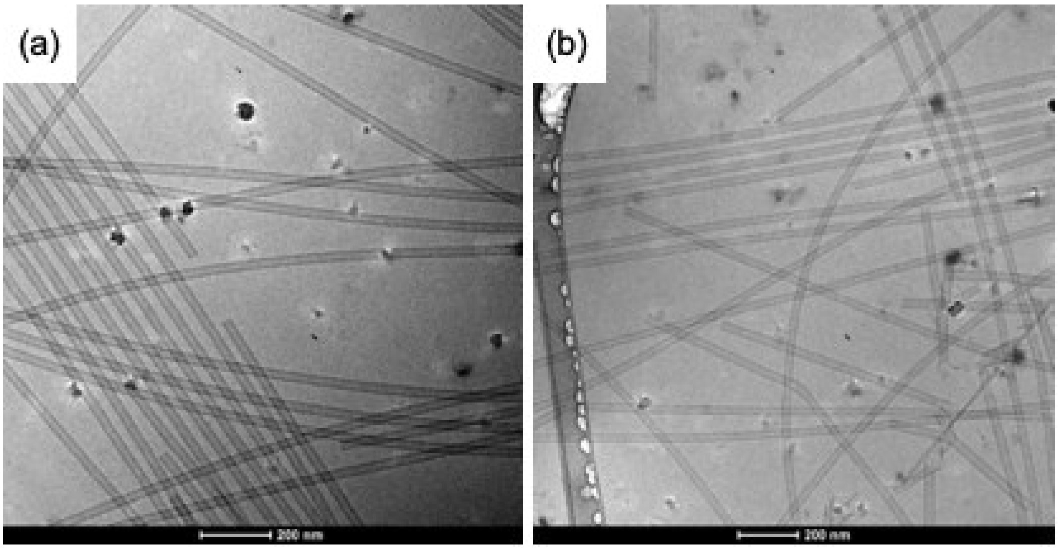

Figure S8. Cryo-TEM images of (L,D)-2NapFF. The scale bar represents 200 nm in both cases.

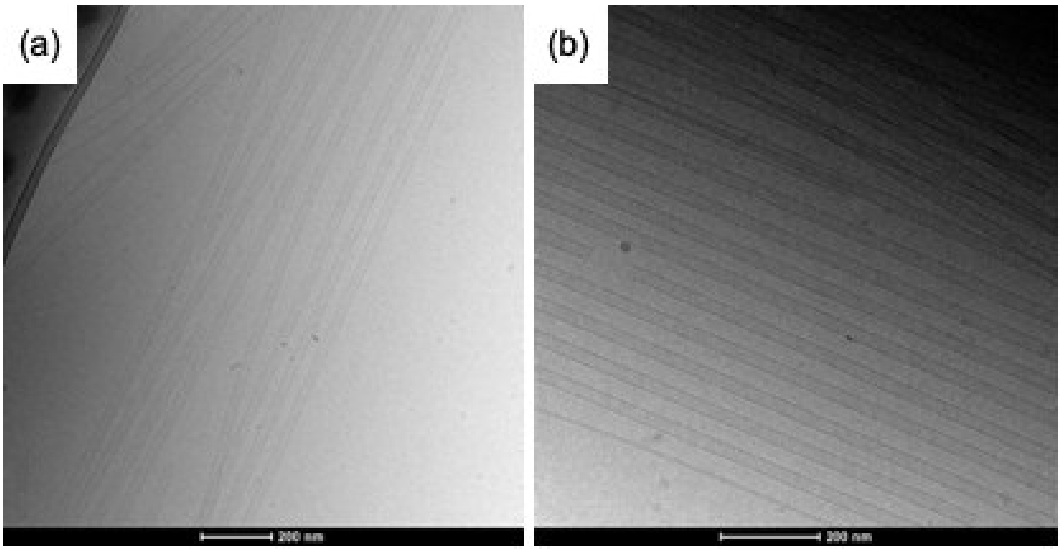

Figure S9. Cryo-TEM images of (D,L)-2NapFF. The scale bar represents 200 nm in both cases.

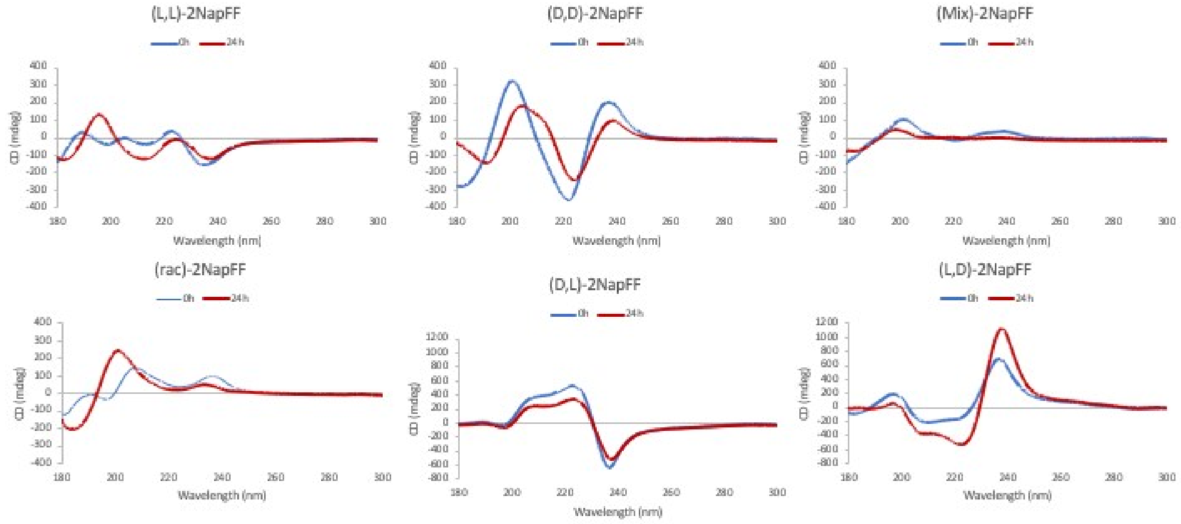

Figure S10. CD for the solutions of the 2NapFF. All data were collected at 10 mg/mL and pH 11. The signals are complicated to interpret at this concentration as both the phenylalanines and the naphthalene rings will absorb. In general, the data are consistent with the different chiralities, showing opposite signals as expected at 230 and 240 nm. We highlight however that direction of the signal for the naphthalenes may not always be the same (the induced helicity for the naphthalene comes from the packing of the dipeptides) and we have shown previously that positive or negative signals are possible even for (L,L)-dipeptides.[1] On top of this, there are peak overlaps and likely some LD contributions due to the anisotropic structures present and the pipetting into a thin cell.

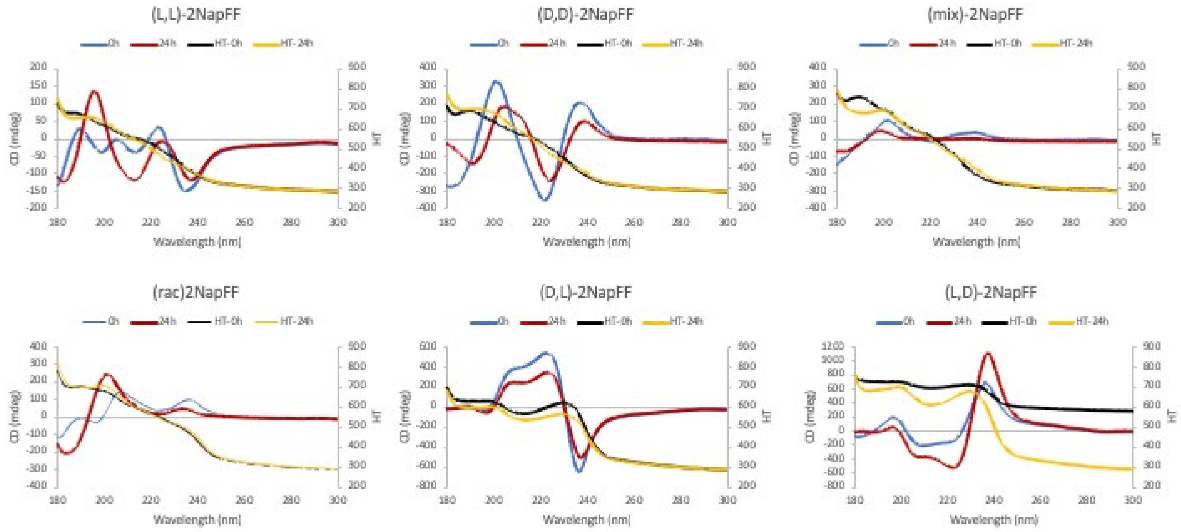

Figure S11. HT and absorption data for the solutions of 2NapFF. All data were collected at 10 mg/mL and pH 11.

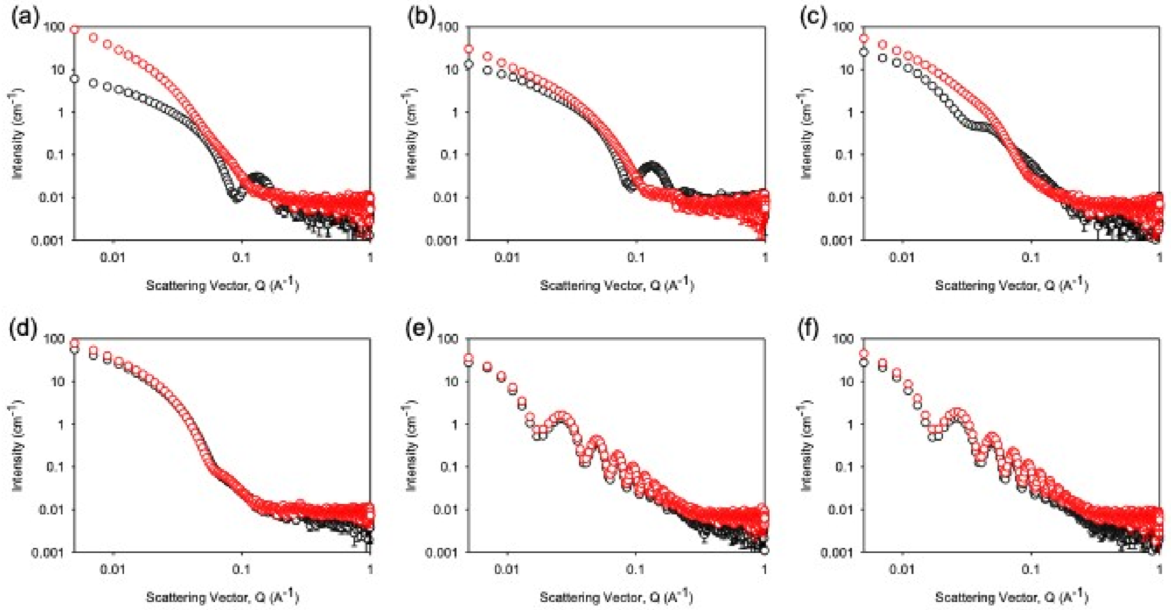

Figure S12. Comparison of SANS scattering from solutions (black) and gels (red) for (a) (L,L)-2NapFF; (b) (D,D)-2NapFF; (c) (mix)-2NapFF; (d) (rac)-2NapFF; (e) (L,D)-2NapFF; (f) (D,L)-2NapFF.

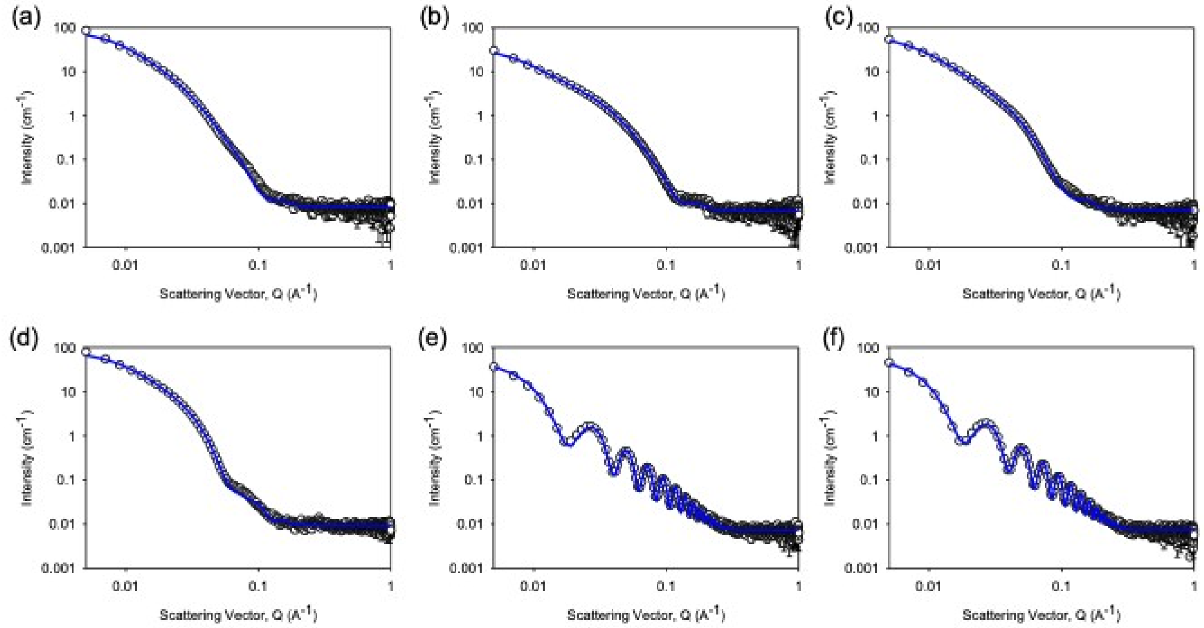

Figure S13. SANS scattering for gels formed from (a) (L,L)-2NapFF; (b) (D,D)-2NapFF; (c) (mix)-2NapFF; (d) (rac)-2NapFF; (e) (L,D)-2NapFF; (f) (D,L)-2NapFF. In all cases, the black circles represent the SANS data and the blue lines show the fit to the data.

	(L,L)-2NapFF	(D,D)-2NapFF	(L,D)-2NapFF	(D,L)-2NapFF	(mix)-2NapFF	(rac)-2NapFF
Background (cm^{-1})	0.0082 ± $6.99x10^{-5}$	0.0067 ± $6.57x10^{-5}$	0.007 ± $5.88x10^{-5}$	0.007 ± $6.54x10^{-5}$	0.0066 ± $5.87x10^{-5}$	0.009 ± $5.14x10^{-5}$
Scale	$1.40x10^{-3}$ ± $5.96x10^{-5}$	$2.22x10^{-3}$ ± $9.97x10^{-6}$	$5.26x10^{-3}$ ± $4.01x10^{-5}$	0.0061 ± $4.18x10^{-5}$	0.0029 ± $1.41x10^{-5}$	0.0034 ± $1.19x10^{-5}$
Radius (Å)	29.8 ± 0.3	27.1 ± 0.1	132.6 ± 0.1	132.4 ± 0.1	30.5 ± 0.1	51.9 ± 0.1
Thickness (Å)			16.4 ± 0.1	16.5 ± 0.1		
Length (Å)	2618 ± 124	1147 ± 27	543.4 ± 4.65	558 ± 4.7	2157 ± 32	902 ± 8
Kuhn Length (Å)	55 ± 4	286.7 ± 11			90 ± 2	226 ± 12
Axis Ratio	2.64 ± 0.2	2.22 ± 0.1			1.74 ± 0.01	1.58 ± 0.01
χ^2	9.3469	1.7144	7.1186	6.5512	3.8440	4.2636

Table S2. Summary of fits to the SANS data for the gels formed for the different 2NapFF chiralities. The data for (L,L)-, (D,D)-, (mix)-, and (rac)-2NapFF were fitted to a flexible elliptical cylinder model. The data for (L,D)- and (D,L)-2NapFF were fitted to a hollow cylinder model.

Further analysis of SANS data of gels

Log-log plots of I(q) v q for the gels formed from (L,L)-2NapFF, (D,D)-2NapFF, (mix)-2NapFF and (rac)-2NapFF show significant differences from the SANS data taken from the corresponding solutions (Fig. S10). By considering the gradients of these graphs, we can qualitatively describe the samples. These plots are given in Figure S12. The SANS data for the gels formed from (L,L)-2NapFF and (D,D)-2NapFF show two distinct gradients, one at a lower q region ($0.007<q<0.03 Å^{-1}$) which has a value of -1.6 in the (L,L)-2NapFF and -1.5 in the (D,D)-2NapFF. The second gradient at $q>0.03 Å^{-1}$ has a value in both cases of -3.9. In the case of the lower q slope, this value is indicative of scattering from a polydisperse non-rigid rod network.[2, 3] The decay at higher Q is in agreement with a q^{-4} variation in intensity of the Porod region, indicating a sharp interface between the gel fibres and solvent. For the samples formed by (mix)-2NapFF and (rac)-2NapFF, the lower q slope has values of -1.7 and -2 respectively. The slope in the Porod region is now much steeper (taking values of -4.7 and -5.5). This deviation is possibly unsurprising as the ideal conditions under which the Porod

exponent has a value of -4 is reliant on there being an infinitely sharp transition between the two phases within the material which may not be seen in real samples. It is also of note that in (rac)-2NapFF, at higher q, there is a third gradient of -2.6, commonly observed in scattering for self-similar rough surfaces such as those seen in gels.[4]

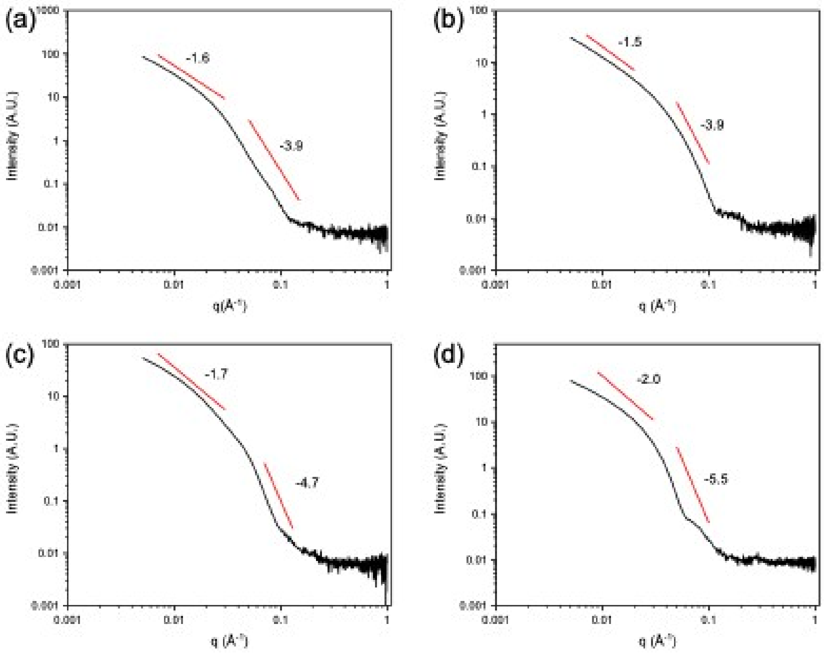

Figure S14. Log-log plots of SANS data for (a) (L,L)-2NapFF; (b) (D,D)-2NapFF; (c) (mix)-2NapFF; (d) (rac)-2NapFF. The gradients, shown in red, have been added by generating a function plot in Origin.

A global fit of each of the SANS patterns shows that the data sets can be best described by a flexible cylinder with an elliptical cross-section. These are shown in Figure S11, with a summary of the fits shown in Table S2. This agrees with the observation in the log-log plots that the data are non-rigid rods. Minor radii, axis ratios and Kuhn lengths are given in Table S2. Due to the q range of the experiment, it is not possible to fit an overall length to the data. The radii of the (L,L)-, (D,D)- and (mix)-2NapFF are similar, at approximately 2.7-3.0Å, whereas the (rac)-2NapFF has a larger radius of approximately 5.1nm. It is clear from the cryo-TEM of the solution of the (rac)-2NapFF that the sample contains a mixture of fibres and tubes and is very polydisperse. It is likely that this polydispersity persists in the gel phase. Examination of the Kuhn lengths obtained from these global fits shows that the (L,L)-2NapFF is significantly more flexible than the (D,D)-2NapFF; interestingly the rheological data shows that the (D,D)-2NapFF is a much stronger gel than that of the (L,L)-2NapFF. There is no obvious reason as to why changing the chirality should have such a dramatic effect on the gel

properties and this will form the basis of further work. The (mix)-2NapFF sample can be seen to be less flexible than the (L,L)-2NapFF but more than the (D,D)-2NapFF. This can be rationalised on the assumption that there is no sorting of the molecules into individual (L,L)-2NapFF and (D,D)-2NapFF fibres and that all fibres will contain some (L,L)-2NapFF and some (D,D)-2NapFF molecules, although each fibre contains a different (and random) amount of each. Thus the overall flexibility of the sample will lie between that of the (L,L)-2NapFF and the (D,D)-2NapFF. The Kuhn length of the (rac)-2NapFF sample shows that it is stiffer than the (L,L)-2NapFF or the (mix)-2NapFF, but less stiff than the (D,D)-2NapFF. As now the sample contains all of the possible combinations of enantiomers, the resulting sample is seen, as highlighted above, to be a mix of fibres and tubes and to have contributions to the stiffness from the packing of each combination of chiral centres.

As an additional confirmation of the size and shape of the fibres within the gel, a modified Guinier plot was produced, by plotting $\ln(Q^{\alpha}I)$ vs Q^2 where $\alpha = 1$ for a rigid rod, and 2 for a ribbon-like fibre with an elliptical (or rectangular) cross-section.[4] It was found that the best linear fit of the data was to a plot where $\alpha = 2$. These modified Guinier plots are shown in Figure S13. A value for the cross-sectional thickness of the scattering object (t) can be extracted from this data[3] and showed that for the RR, t = 5.3 nm. Based on the radius of the global fit, the thickness here was found to be 5.4 nm, which is in good agreement. Similarly, for the SS, t = 6.3 nm, with a diameter from the global fit found to be 6.0nm. The same analysis was performed on the racemic gel and mixed gel. For the racemic gel, t = 10.6 nm, corresponding well to the global fit which gave a diameter of 10.2 nm. In the case of the mixed gel, t = 6.8 nm, which compares to a value of the diameter from the global fit of 6.1nm. In all cases, prior to the linear region in the $\ln(Q^2I)$ v Q^2 plot there is a bump as Q tends to 0 which is suggestive of thicker bundles of fibres which form the gel network.[4]

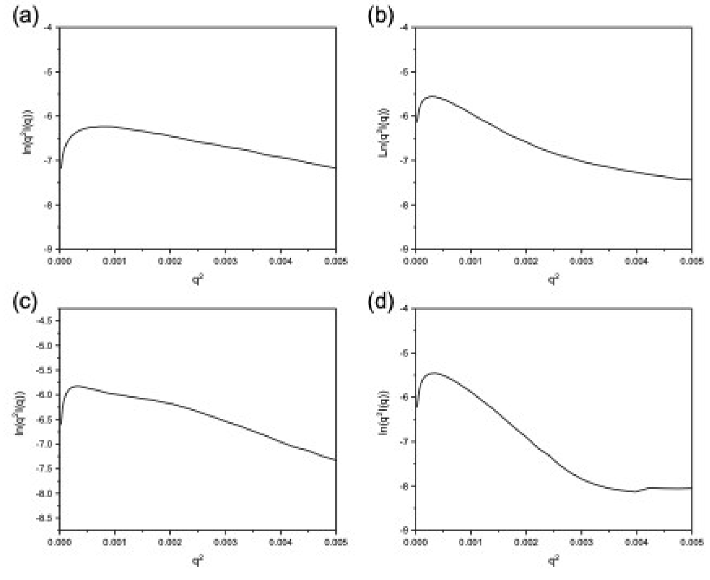

Figure S15. Plots of ln(q^2I(q)) v q^2 to determine the cross section of the fibre in (a) (L,L)-2NapFF; (b) (D,D)-2NapFF; (c) (mix)-2NapFF; (d) (rac)-2NapFF.

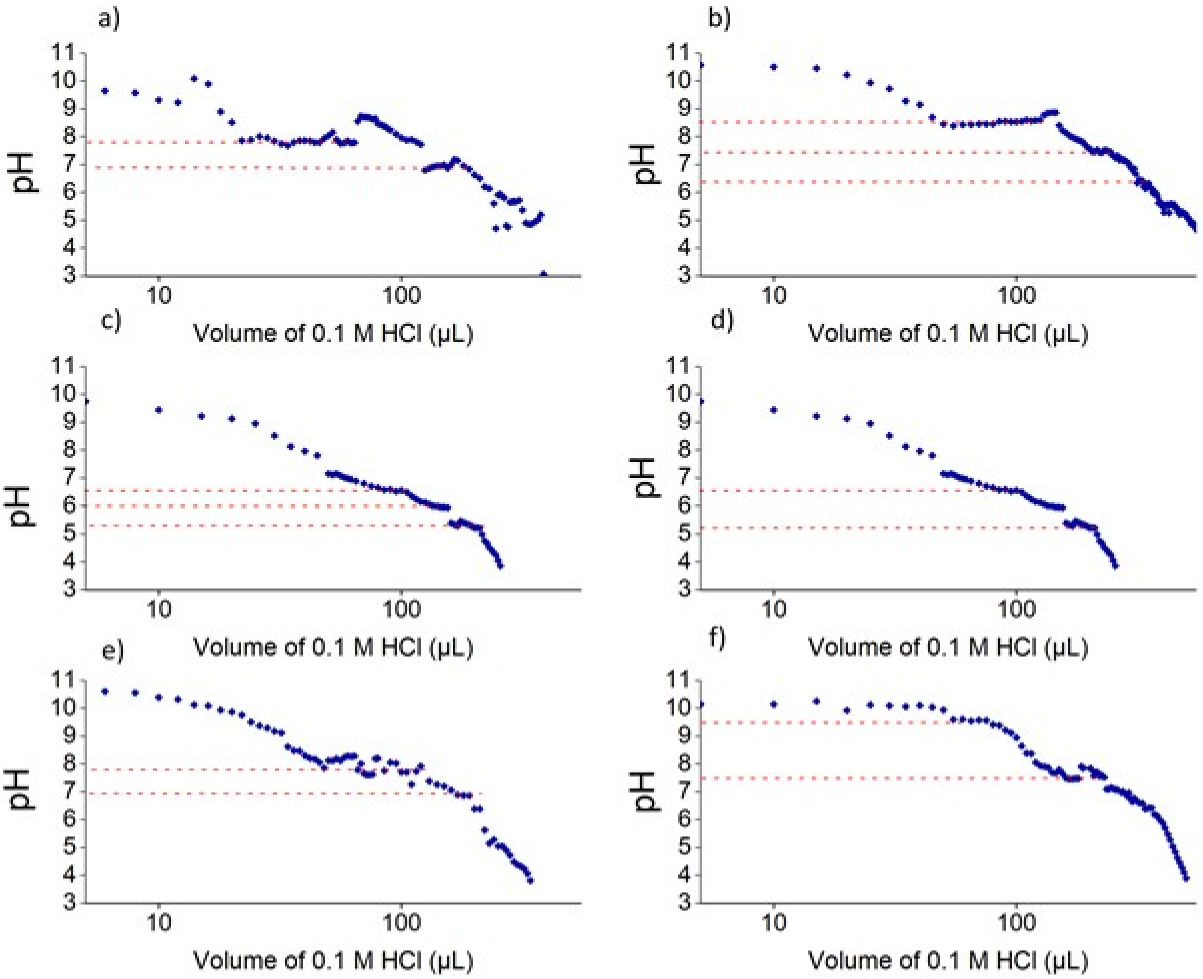

Figure S16. Apparent pK_a Titrations for different 2NapFF: (a) (L,L)-2NapFF; (b) (D,D)-2NapFF; (c) (L,D)-2NapFF; (d) (D,L)-2NapFF; (e) (mix)-2NapFF; (f) (rac)-2NapFF. Dashed lines give the pH at the apparent pK_a.

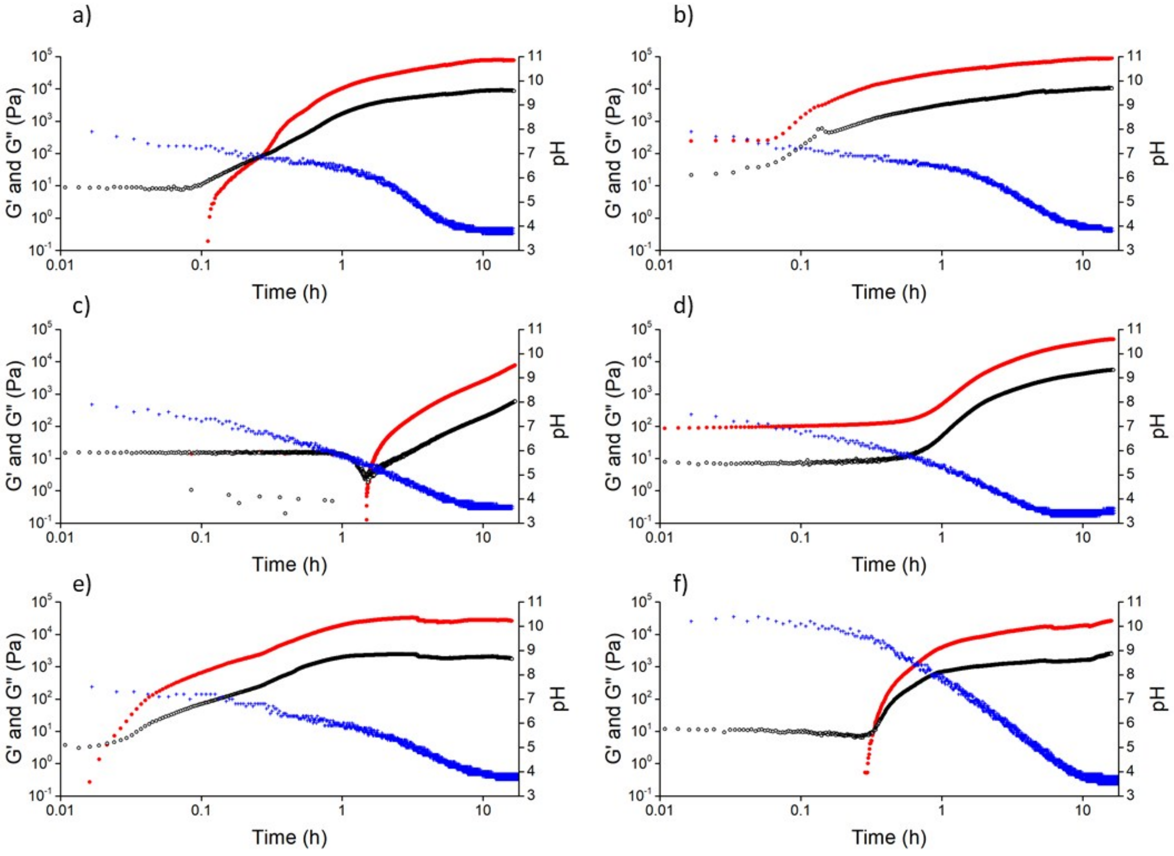

Figure S17. Rheological time sweeps and pH measurements for gelation of the 2NapFF solutions by addition of GdL: (a) (L,L)-2NapFF; (b) (D,D)-2NapFF; (c) (L,D)-2NapFF; (d) (D,L)-2NapFF; (e) (mix)-2NapFF; (f) (rac)-2NapFF. Closed symbols represent G', open symbols represent G'' and crosses represent pH.

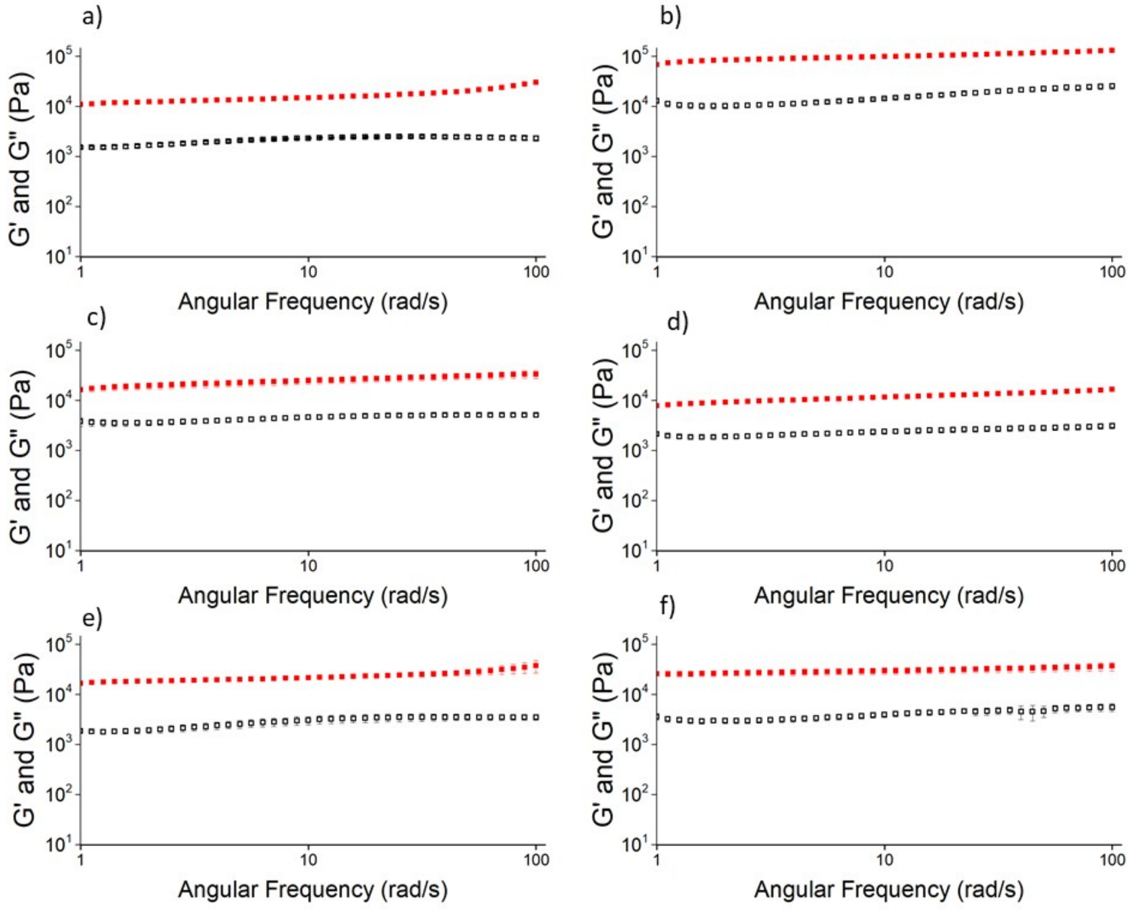

Figure S18. Frequency sweeps for 10 mg/mL 2NapFF samples: a) (L,L)-2NapFF; (b) (D,D)-2NapFF; (c) (L,D)-2NapFF; (d) (D,L)-2NapFF; (e) (mix)-2NapFF; (f) (rac)-2NapFF. Closed symbols represent G' and open symbols represent G''.

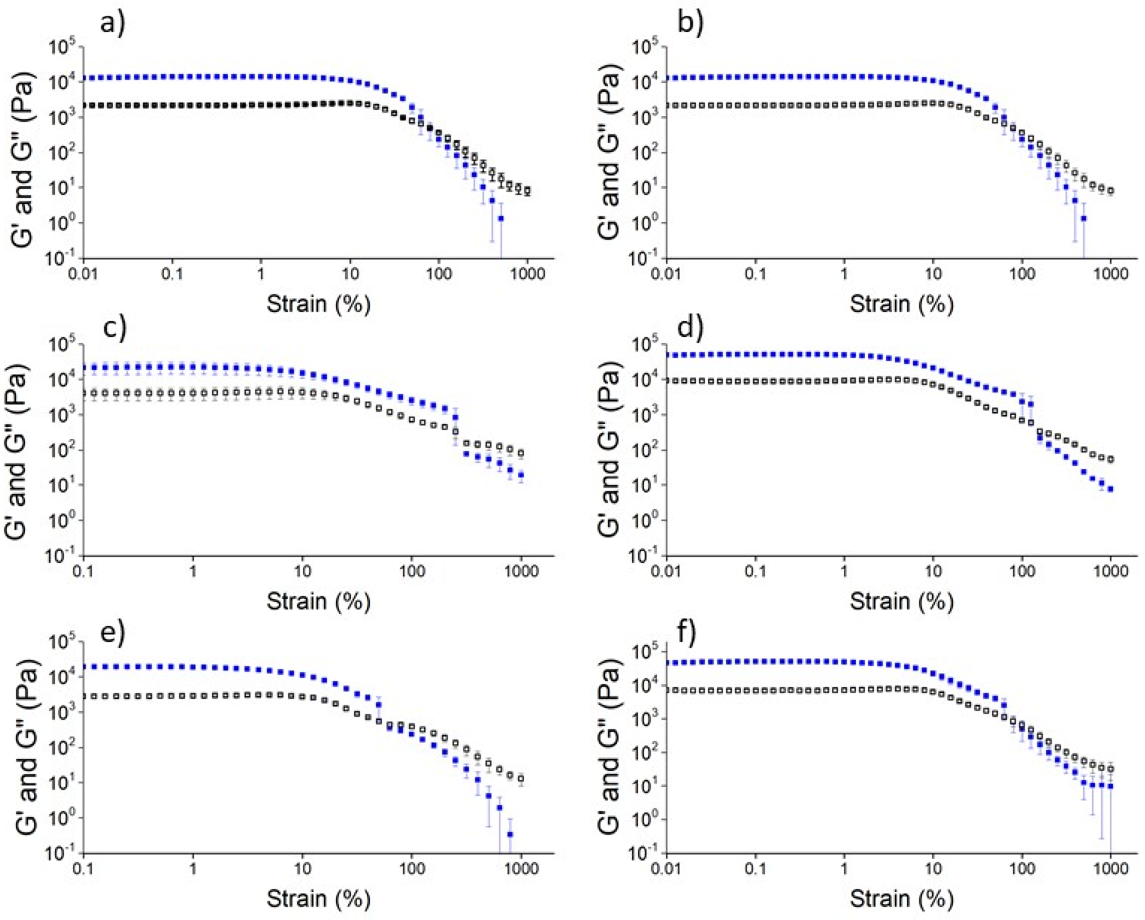

Figure S19. Strain sweeps for 10 mg/mL 2NapFF gel samples: a) (L,L)-2NapFF; (b) (D,D)-2NapFF; (c) (L,D)-2NapFF; (d) (D,L)-2NapFF; (e) (mix)-2NapFF; (f) (rac)-2NapFF. Closed symbols represent G' and open symbols represent G".

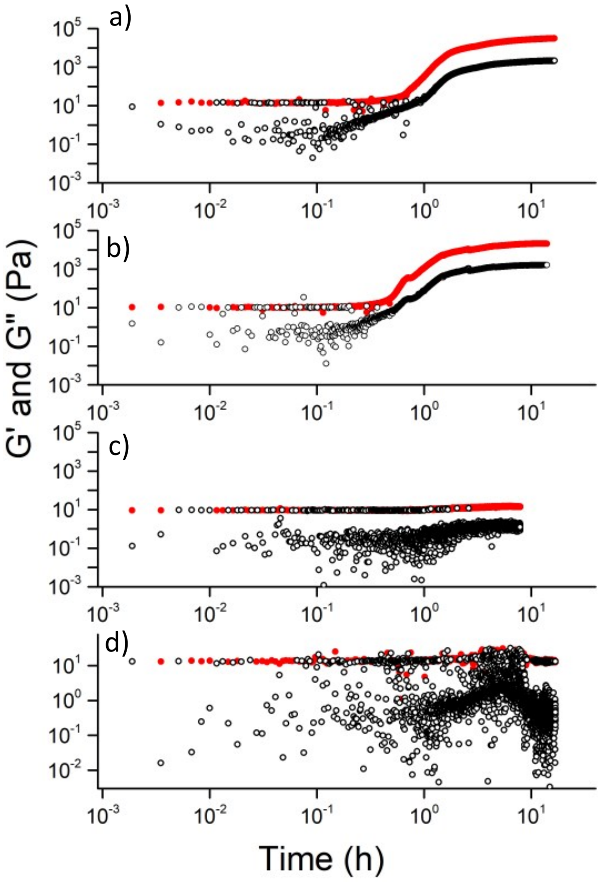

[redacted]

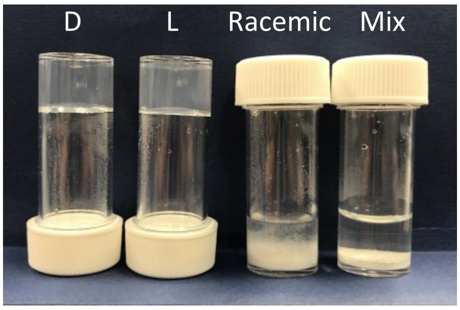

Figure S20. Time Sweeps for 5 mg/mL BrNapAG solutions with GdL: (a) (L)-, b) (D)-, c) (mix)- and (d) (rac)-BrNapAG. Closed symbols represent G' and open symbols represent G". Picture shows the samples after 24 hours and the molecular structure is given. The chiral centre is shown by the *.

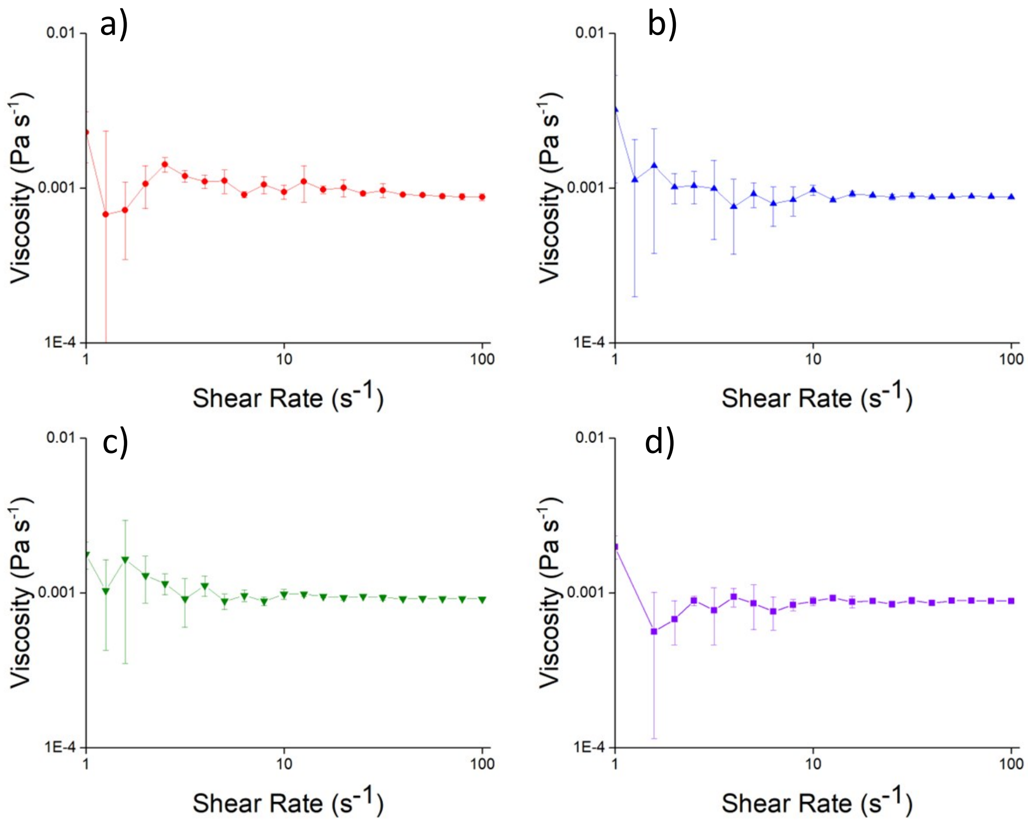

Figure S21. Viscosity data for BrNapAG 5 mg/mL at pH 11: (a) (L)-, b) (D)-, c) (mix)- and (d) (rac)-BrNapAG.

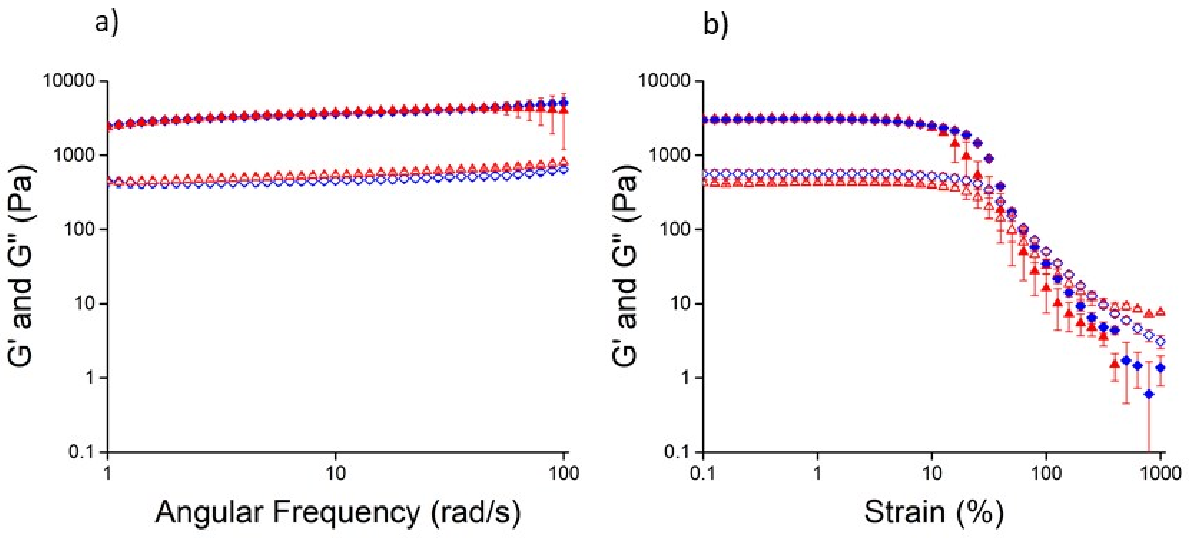

Figure S22. Frequency (a) and strain (b) sweeps of (L)-BrNapAG (red) and (D)-BrNapAG (diamonds). Closed symbols represent G' and open symbols represent G''.

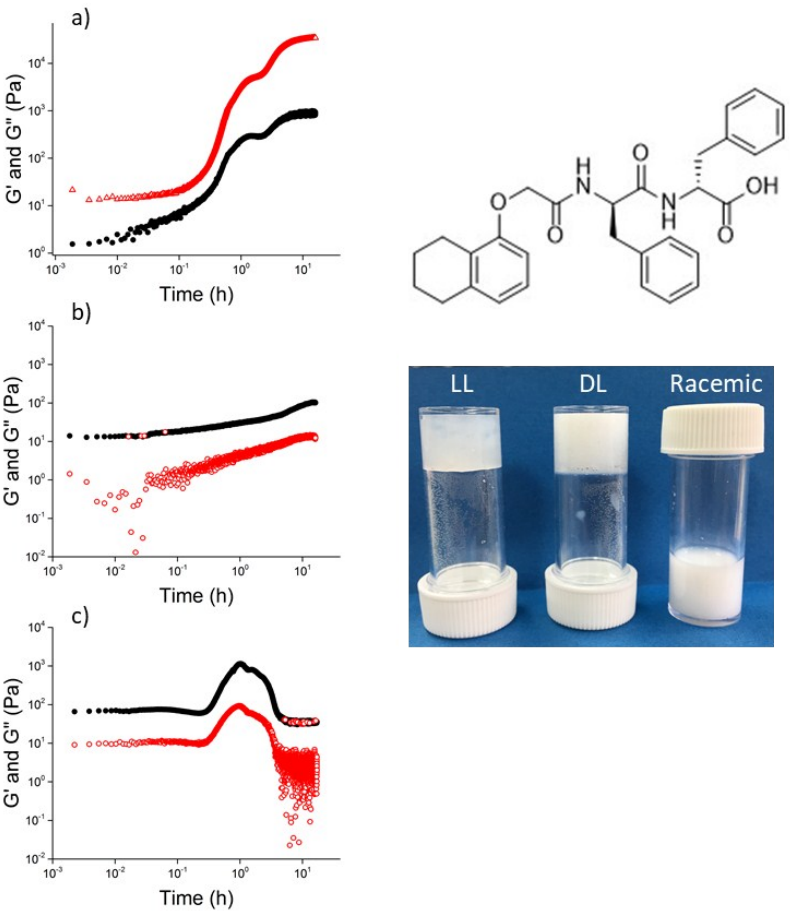

Figure 23. Time Sweeps for 1ThNapFF solutions (10 mg/mL) with GdL: (a) (L,L)-; (b) (D,L)-; (c) (rac)-1ThNapFF. Closed symbols represent G' and open symbols represent G''. Picture shows the samples after 24 hours and the molecular structure is given for the (L,L)-1ThNapFF.

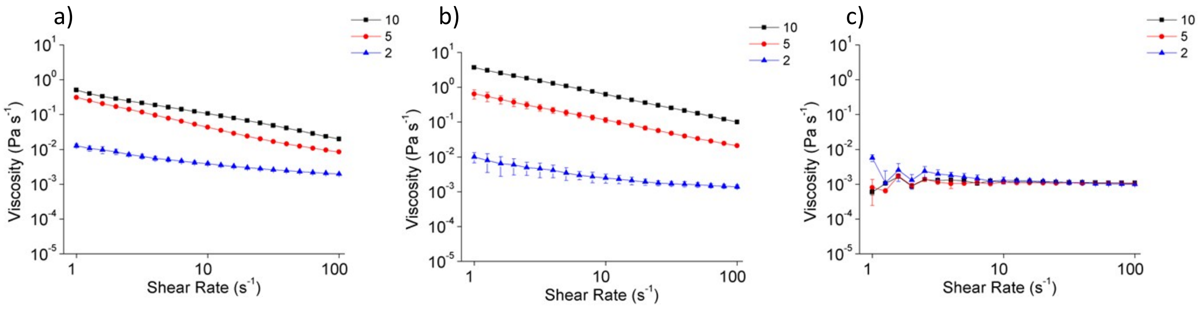

Figure S24. Viscosity data for 1ThNapFF solutions at pH 11 and 2, 5 and 10 mg/mL. An average of three measurements is shown with error bars representing standard deviation. Numbers indicate the concentration of 1ThNapFF solution: (a) (L,L)-; (b) (D,L)-; (c) (rac)-1ThNapFF.

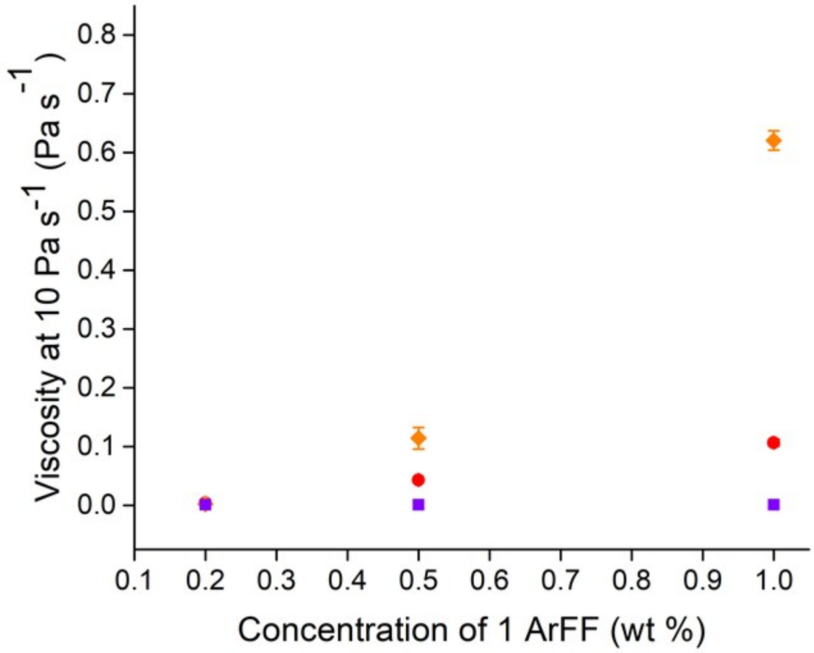

Figure S25. Viscosity data for different chiral conformations of 1ThNapFF at pH 11 for 2, 5 and 10 mg/mL at a shear rate of 10 s^{-1}. Red circle symbols show data for (L,L)-; mustard diamond data are for (D,L)-; purple square data are for (rac)-1ThNapFF.

2. Synthetic Details

All reagents and solvents were purchased from the usual commercial suppliers and used as received. NMR spectra were obtained on Bruker Avance III or Avance III HD 400 or 500 MHz machines at the University of Glasgow. Chemical shifts are in ppm, coupling constants in Hz. Proton and carbon assignments were aided by COSY and HSQC experiments, respectively, where required. Proton spectra are referenced to residual solvent peaks at 2.50 ppm (DMSO-d_6) or 2.05 ppm (acetone-d_6). Carbon spectra are referenced to residual solvent peaks at 39.52 ppm (DMSO-d_6). Mass spectra were recorded at the University of Glasgow on a Bruker micrOTOFQ. MarvinSketch 16.11.28 (2016) was used for naming of chemical structures (ChemAxon, http://www.chemaxon.com).

The synthetic step which effects the Boc deprotection of the dipeptide ester (*e.g.* **DH-002** to **DH-003**) works well with either trifluoroacetic acid or hydrogen chloride solution. The trifluoroacetic acid method however relies on precipitating the product from excess diethyl ether. This removes trace impurities as well as excess trifluoroacetic acid, which is otherwise difficult to remove. In the case of the (*rac*,*rac*) derivatives (*e.g.* **ED-006** and subsequent steps), there was concern that precipitation from diethyl ether might skew the isomeric distribution of the mixture due to differential solubilities of its components. It was indeed found that initial trituration of the evaporated reaction mixture with diethyl ether provided a white solid which was highly enriched in the (*S*,*S*) and (*R*,*R*) isomers, while subsequent crystallisation of the mother liquor provided a white solid highly enriched in the (*R*,*S*) and (*S*,*R*) isomers. The yields for both crops of isomers were roughly equal and represented a 73 % total yield. The deprotection of the (*rac*,*rac*) compound was instead carried out with hydrogen chloride in diethyl ether or dioxane. The workup for this reaction is simple evaporation of the excess HCl and solvent, however it provides no means of washing out impurities. The precursor to this step (**ED-006**) must therefore be pure, or any impurities will remain in the product.

(L,L)-1ThNapFF was prepared as described elsewhere.[5] 2-Naphthoxyacetic acid and 6-bromo-2-naphthoxyacetic acid were prepared as described previously.[6]

Ethyl (2*S*)-2-[(2*S*)-2-{[(*tert*-butoxy)carbonyl]amino}-3-phenylpropanamido]-3-phenylpropanoate (DH-002)

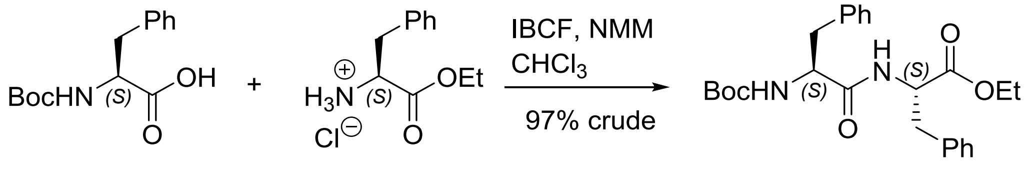

BocPheOH **HCl·PheOEt** **DH-002**

To a solution of Boc-*L*-phenylalanine (5.28 g, 19.9 mmol) in chloroform (50 mL) were added *iso*-butyl chloroformate (1 eq, 2.58 mL) and *N*-methylmorpholine (1 eq, 2.19 mL) and the mixture was stirred for one hour. *L*-Phenylalanine ethyl ester hydrochloride (1 eq, 4.57 g) and *N*-methylmorpholine (1 eq, 2.19 mL) were then added and the mixture was stirred overnight. The now clear solution was diluted with chloroform and washed in turn with 1M hydrochloric acid, water, and brine, dried ($MgSO_4$), and evaporated under reduced pressure. The crude title compound was obtained as an off-white solid (8.49 g, 97 %) and used in the next step without further purification. A small amount was purified *via* column chromatography (eluting with 1:99 ethyl acetate/dichloromethane) to yield an analytical sample. Pronounced effects of restricted rotation are observed in the NMR data of **DH-002**, with the signals for several protons and carbons being split approximately 1:4 into two rotational isomers. These are denoted Rot-1 and Rot-2 in the NMR data below. When the NMR experiment is carried out at higher temperature, the split signals coalesce, as expected for rotamers.

δ_H (500 MHz, DMSO-d_6, 25 °C) 8.38 (0.2H, d, *J* 6.75, Rot-1 $N\underline{H}CH^{*}CO_2Et$), 8.32 (0.8H, d, *J* 7.50, Rot-2 $N\underline{H}CH^{*}CO_2Et$), 7.30-7.16 (10H, m, $\underline{H}_{Ar}$), 6.85 (0.8H, d, *J* 8.80, Rot-2 $N\underline{H}Boc$), 6.41 (0.2H, d, *J* 8.50, Rot-1 $N\underline{H}Boc$), 4.47 (1H, dd, *J* 14.33, 7.73, $C\underline{H}^{*}NHBoc$), 4.18 (0.8H, td, *J* 9.65, 4.00, Rot-2 $C\underline{H}^{*}CO_2Et$), approx. 4.08-4.02 (0.2H, Rot-1 $C\underline{H}^{*}CO_2Et$), 4.03 (2H, q, *J* 7.10, $C\underline{H}_2CH_3$), 3.05-2.95 (2H, m, $PhC_a\underline{H}_2$), 2.89 (1H, dd, *J* 13.80, 3.95, $PhC_b\underline{H}_aH_b$), 2.67 (1H, dd, *J* 13.73, 10.68, $PhC_bH_a\underline{H}_b$), 1.28 (7.2H, s, Rot-2 $C(C\underline{H}_3)_3$), 1.14 (1.8H, s, Rot-1 $C(C\underline{H}_3)_3$), 1.09 (3H, t, *J* 7.10, $CH_2C\underline{H}_3$). δ_H (500 MHz, DMSO-d_6, 80 °C) 7.95 (1H, d, *J* 7.30, $N\underline{H}CH^{*}CO_2Et$), 7.29-7.16 (10H, m, $\underline{H}_{Ar}$), 6.39 (1H, br s, $N\underline{H}Boc$), 4.55 (1H, td, *J* 7.82, 6.33, $C\underline{H}^{*}NHBoc$), 4.22 (1H, td, *J* 9.08, 4.70, $C\underline{H}^{*}CO_2Et$), 4.07 (2H, q, *J* 7.09, $C\underline{H}_2CH_3$), 3.09-2.93 (3H, m, $PhC\underline{H}_2$), 2.74 (1H, dd, *J* 13.95, 9.55, $PhC\underline{H}_2$), 1.30 (9H, s, $C(C\underline{H}_3)_3$), 1.14 (3H, t, *J* 7.10, $CH_2C\underline{H}_3$). δ_C (100 MHz, DMSO-d_6, 25 °C) 171.79, 171.25, and 155.07 ($\underline{C}$=O), 138.03, 136.96, 129.12 (broad, likely two overlapping peaks), 128.20, 127.93, 126.52, and 126.12 ($\underline{C}_{Ar}$), 77.96 ($\underline{C}(CH_3)_3$), 60.47 ($\underline{C}H_2CH_3$), 55.44 ($\underline{C}H^{*}CO_2Et$), 53.54 ($\underline{C}H^{*}NHBoc$), 37.41 ($Ph\underline{C}_bH_2$), 36.75 ($Ph\underline{C}_aH_2$), 28.08 (Rot-2 $C(\underline{C}H_3)_3$), 27.73 (Rot-1 $C(\underline{C}H_3)_3$), 13.88 ($CH_2\underline{C}H_3$). δ_C (125 MHz,

DMSO-d_6, 80 °C) 170.97, 170.57, and 154.40 ($\underline{C}$=O), 137.47, 136.59, 128.64, 128.60, 127.69, 127.46, 126.00, 125.64 ($\underline{C}_{Ar}$), 77.82 ($\underline{C}(CH_3)_3$), 60.03 ($\underline{C}H_2CH_3$), 55.29 ($\underline{C}H^*CO_2Et$), 53.04 ($\underline{C}H^*NHBoc$), 37.30 ($Ph\underline{C}_bH_2$), 36.68 ($Ph\underline{C}_aH_2$), 27.66 ($C(\underline{C}H_3)_3$), 13.36 ($CH_2\underline{C}H_3$). HRMS (ESI) m/z: $[M+Na]^+$ calcd for $C_{25}H_{32}N_2NaO_5$ 463.2203; found 463.2190.

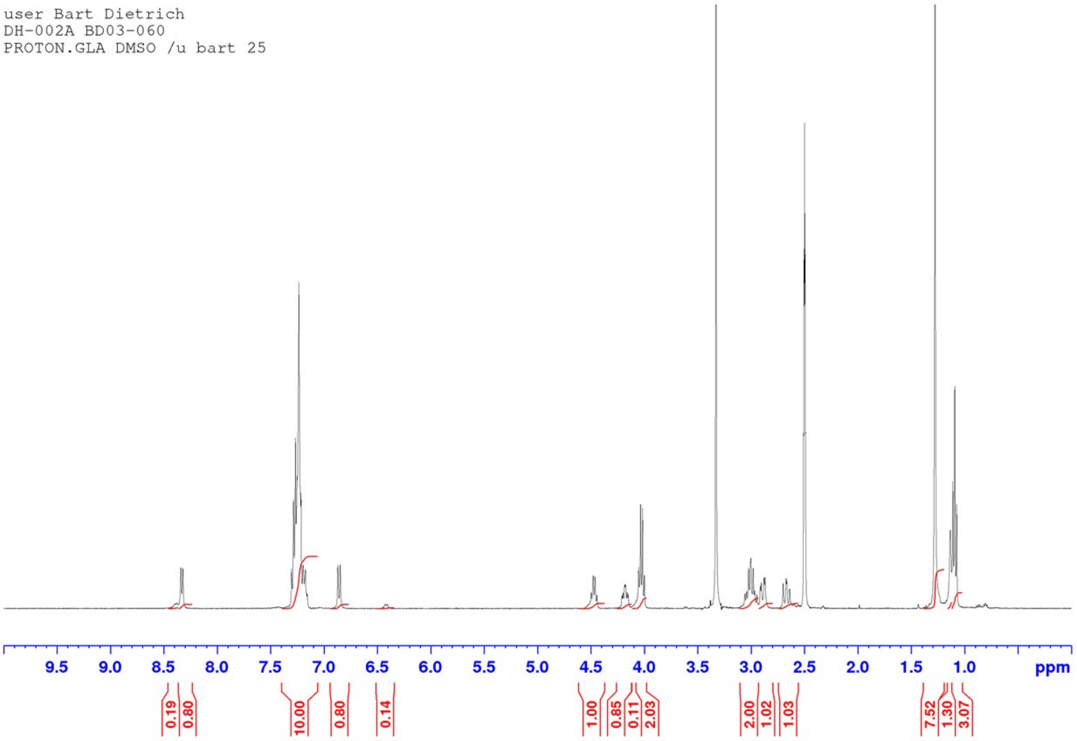

Figure S26. Proton NMR of **DH-002** at 25 °C.

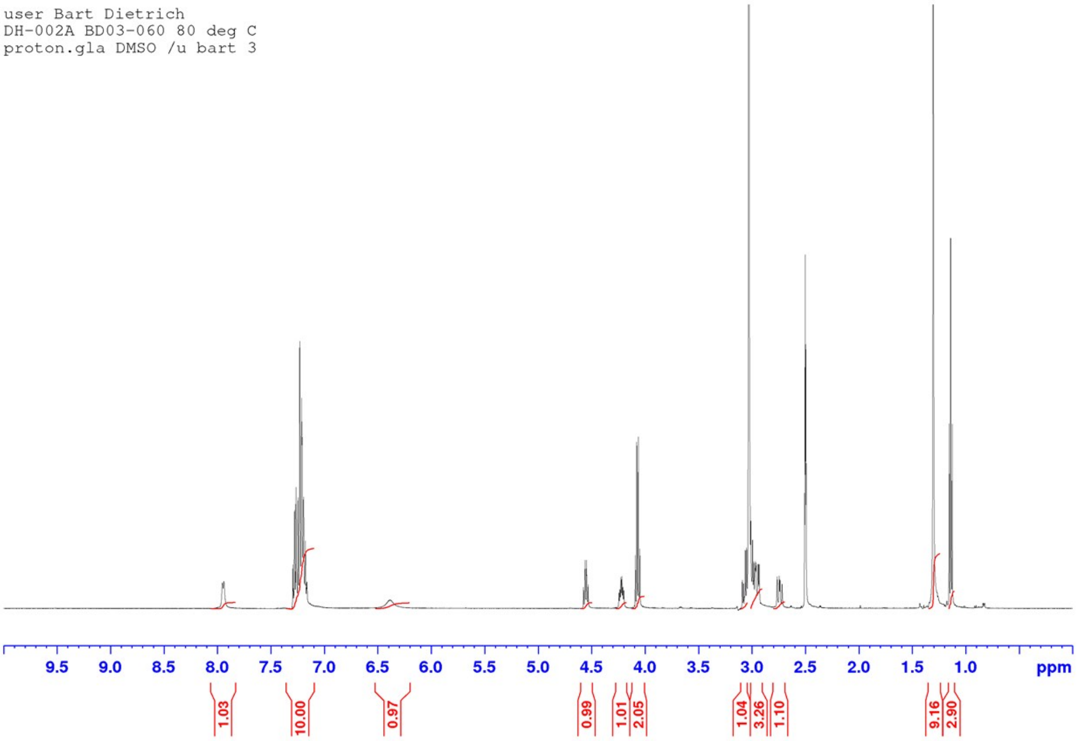

Figure S27. Proton NMR of **DH-002** at 80 °C.

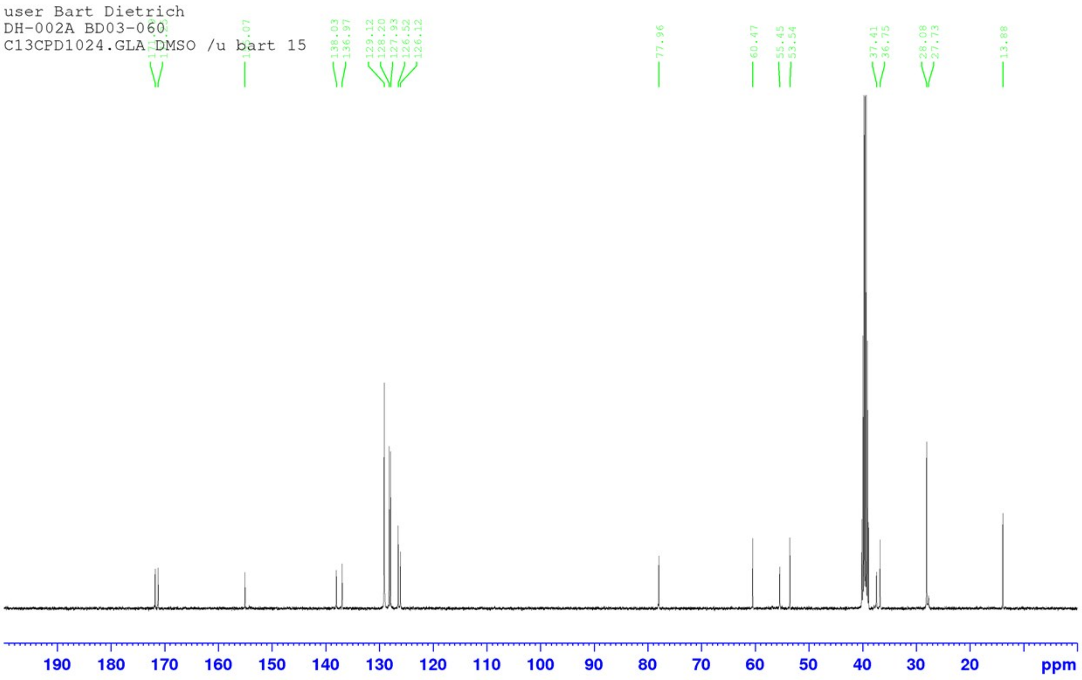

Figure S28. Carbon NMR of **DH-002** at 25 °C.

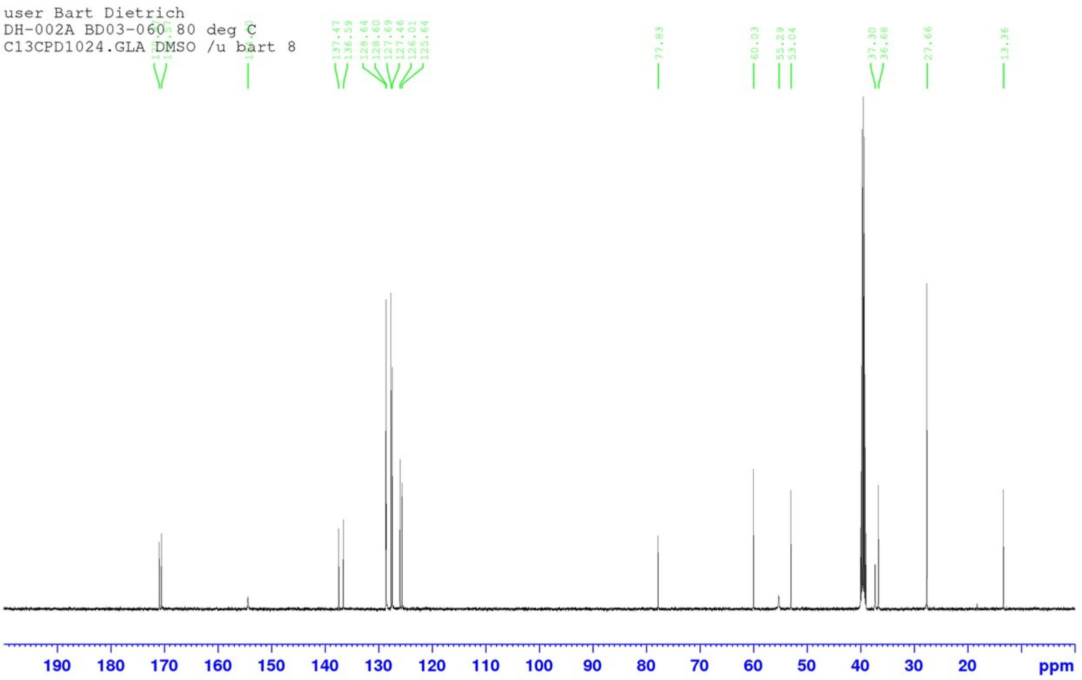

Figure S29. Carbon NMR of **DH-002** at 80 °C.

Ethyl (2*S*)-2-[(2*S*)-2-amino-3-phenylpropanamido]-3-phenylpropanoate trifluoroacetate salt (DH-003)

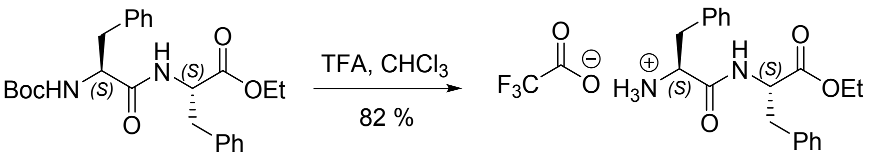

DH-002 **DH-003**

To a solution of **DH-002** (8.21 g, 18.6 mmol) in chloroform (30 mL) was added trifluoroacetic acid (15 mL, approx. 10 eq) and the mixture was stirred overnight. After this time, the reaction was concentrated under reduced pressure to remove most of the excess TFA. The resulting viscous oil was dissolved in chloroform (*ca.* 30 mL), poured into diethyl ether (*ca.* 400 mL) and stirred overnight. The precipitate was filtered off, washed in the filter with a few small portions of diethyl ether and dried under reduced pressure. The title compound was thus obtained as a white solid (6.95 g, 82 %). A small amount of residual diethyl ether is seen in both the proton and carbon NMR spectra.

δ_H (400 MHz, DMSO-d_6) 9.00 (1H, d, *J* 7.56, N$\underline{H}$), 8.13 (3H, br s, N$\underline{H}_3^+$), 7.35-7.23 (10H, m, $\underline{H}_{Ar}$), 4.55 (1H, dd, *J* 14.16, 7.76, C$\underline{H}^*NH_3^+$), 4.06 (2H, q, *J* 7.10, C$\underline{H}_2CH_3$), 4.08-4.03 (1H, m, C$\underline{H}^*CO_2Et$), 3.14-2.90 (4H, m, PhC$\underline{H}_2$), 1.11 (3H, t, *J* 7.10, $CH_2C\underline{H}_3$). δ_C (100 MHz, DMSO-d_6) 170.74 and 168.32 (CH-$\underline{C}$=O), 158.29 (q, *J* 31.08, CF_3-$\underline{C}$=O), 136.73, 134.80, 129.60, 129.16, 128.51, 128.40, 127.18, and 126.76 ($\underline{C}_{Ar}$), 117.29 (q, *J* 299.82, $\underline{C}F_3$), 60.79 ($\underline{C}H^*CO_2Et$), 53.97 ($\underline{C}H^*NH_3^+$), 53.16 ($\underline{C}H_2CH_3$), 36.92 (Ph$\underline{C}H_2$), 36.79 (Ph$\underline{C}H_2$), 13.94 ($CH_2\underline{C}H_3$). HRMS (ESI) m/z: $[M-TFA+H]^+$ calcd for $C_{20}H_{25}N_2O_3$ 341.1860; found 341.1845.

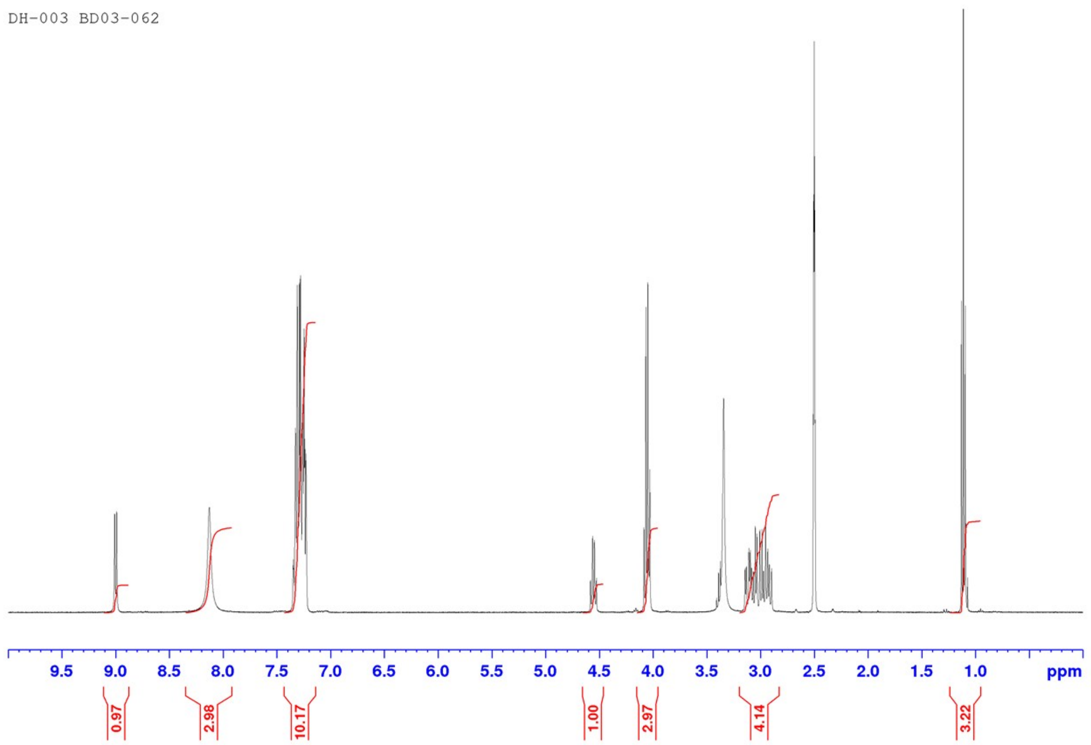

Figure S30. Proton NMR of **DH-003**.

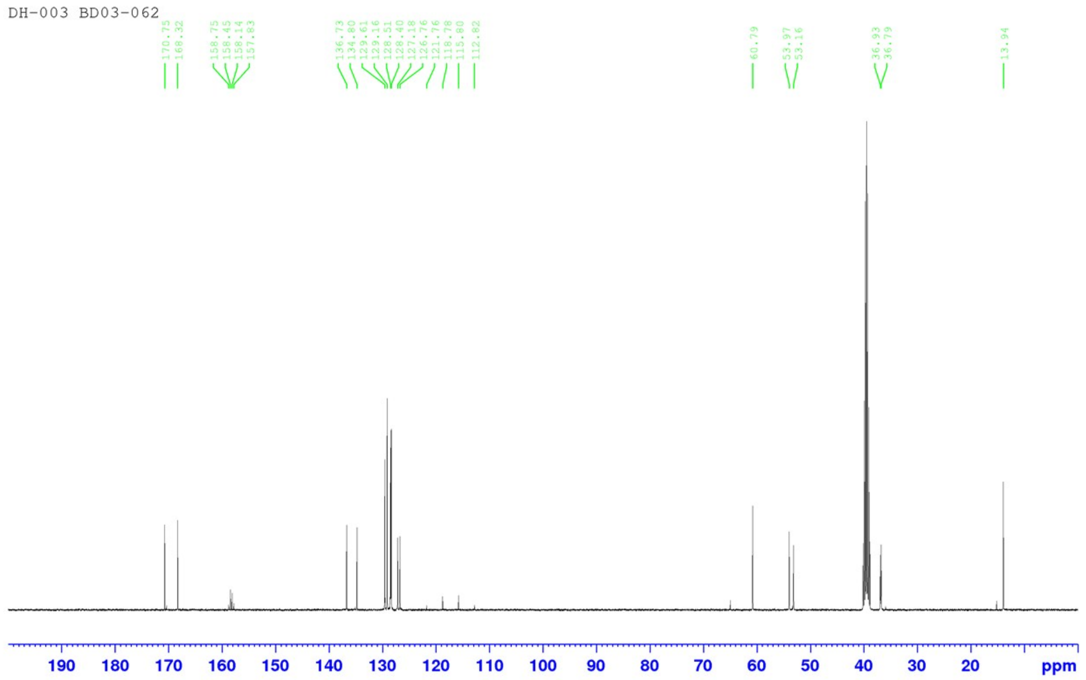

Figure 31. Carbon NMR of **DH-003**.

<u>Methyl (2*R*)-2-[(2*R*)-2-{[(*tert*-butoxy)carbonyl]amino}-3-phenylpropanamido]-3-phenylpropanoate (DF-003)</u>

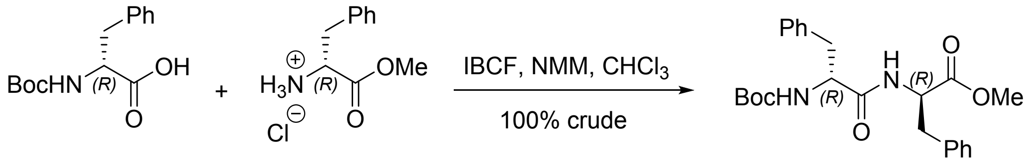

DF-003

To a solution of *N*-Boc-*D*-phenylalanine (1.14 g, 4.30 mmol) in chloroform (25 mL) was added *N*-methylmorpholine (1 eq, 472 μL) and *iso*butyl chloroformate (1 eq, 558 μL) and the mixture was stirred for 20 minutes. After this time, *D*-phenylalanine methyl ester hydrochloride (1 eq, 927 mg) and another portion of *N*-methylmorpholine (1 eq, 472 μL) were added and the reaction was stirred overnight. After this time, it was diluted with chloroform, washed in turn with 1 M hydrochloric acid, Na_2CO_3 (aq), water, and brine, dried ($MgSO_4$) and evaporated under reduced pressure. Crude **DF-003** was thus obtained as a white solid (1.86 g, 100%) and used as is in the next step. A small amount was purified *via* column chromatography (1:99 ethyl acetate / dichloromethane) to obtain an analytical sample.

Pronounced effects of restricted rotation are observed in the NMR data of **DF-003**, with the signals for several protons and carbons being split approximately 1:4 into two rotational isomers. These are denoted Rot-1 and Rot-2 in the NMR data below. When the NMR experiment is carried out at higher temperature, the split signals coalesce, as expected for rotamers.

δ_H (400 MHz, DMSO-d_6, 25 °C) 8.39 (0.2H, d, *J* 6.32, Rot-1 $N\underline{H}CH^*CO_2Me$), 8.33 (0.8H, d, *J* 7.68, Rot-2 $N\underline{H}CH^*CO_2Me$), 7.30-7.16 (10H, m, Ph), 6.85 (0.8H, d, *J* 8.68, Rot-2 $N\underline{H}Boc$), 6.41 (0.2H, d, *J* 8.12, Rot-1 $N\underline{H}Boc$), 4.50 (1H, dd, *J* 13.80, 7.96, $C\underline{H}^*NHBoc$), 4.17 (0.8H, td, *J* 9.53, 3.90, Rot-2 $C\underline{H}^*CO_2Me$), 4.09-4.02 (0.2H, m, Rot-1 $C\underline{H}^*CO_2Me$), 3.60 (0.6H, s, Rot-1 $OC\underline{H_3}$), 3.58 (2.4H, s, Rot-2 $OC\underline{H_3}$), 3.04 (1H, dd, *J* 13.92, 5.88, $PhC\underline{H_a}H_bCH^*NHBoc$), 2.96 (1H, dd, *J* 13.74, 8.38, $PhCH_a\underline{H_b}CH^*NHBoc$), 2.88 (1H, dd, *J* 13.84, 4.08, $PhC\underline{H_a}H_bCH^*CO_2Me$), 2.66 (1H, dd, *J* 13.82, 10.50, $PhCH_a\underline{H_b}CH^*CO_2Me$), 1.28 (7.2H, s, Rot-2 $C(C\underline{H_3})_3$), 1.14 (1.8H, s, Rot-1 $C(C\underline{H_3})_3$). δ_H (500 MHz, DMSO-d_6, 80 °C) 7.97 (1H, d, *J* 6.60, $N\underline{H}$), 7.29-7.16 (10H, m, Ph), 6.39 (1H, br s, $N\underline{H}$), 4.58 (1H, dd, *J* 14.00, 7.85, $C\underline{H}^*$), 4.21 (1H, td, 8.90, 4.75, $C\underline{H}^*$), 3.61 (3H, s, $OC\underline{H_3}$), 3.10-2.92 (3H, m, $PhC\underline{H_2}$), 2.73 (1H, dd, *J* 13.85, 9.50, $PhC\underline{H_2}$), 1.30 (9H, s, $C(C\underline{H_3})_3$). δ_C (100 MHz, DMSO-d_6, 25 °C) 171.85, 171.81, and 155.10 ($\underline{C}$=O), 138.03, 137.00, 129.16, 129.12, 128.26, 127.97, 126.58, and 126.16 ($\underline{C}_{Ar}$), 78.00 ($\underline{C}(CH_3)_3$), 56.90 (Rot-1 $\underline{C}H^*CO_2Me$), 55.51 (Rot-2 $\underline{C}H^*CO_2Me$), 53.51 ($\underline{C}H^*NHBoc$), 51.84 ($O\underline{C}H_3$), 37.42 ($Ph\underline{C}H_2CH^*CO_2Me$), 36.71 ($Ph\underline{C}H_2CH^*NHBoc$), 28.11 (Rot-2 $C(\underline{C}H_3)_3$), 27.74 (Rot-1 $C(\underline{C}H_3)_3$). □$_C$ (125 MHz, DMSO-d_6, 80 °C) 171.12, 171.04, and 154.46 ($\underline{C}$=O), 137.46, 136.61, 128.69, 128.60, 127.77, 127.52, 126.08, and 125.70 ($\underline{C}_{Ar}$), 77.92 ($\underline{C}(CH_3)_3$), 55.40 ($\underline{C}H^*CO_2Me$), 53.02 ($\underline{C}H^*NHBoc$), 51.26 ($O\underline{C}H_3$), 37.33 ($Ph\underline{C}H_2CH^*CO_2Me$), 36.67 ($Ph\underline{C}H_2CH^*NHBoc$), 27.70 ($C(\underline{C}H_3)_3$). HRMS (ESI) m/z: $[M+Na]^+$ calcd for $C_{24}H_{30}N_2NaO_5$ 449.2047; found 449.2046.

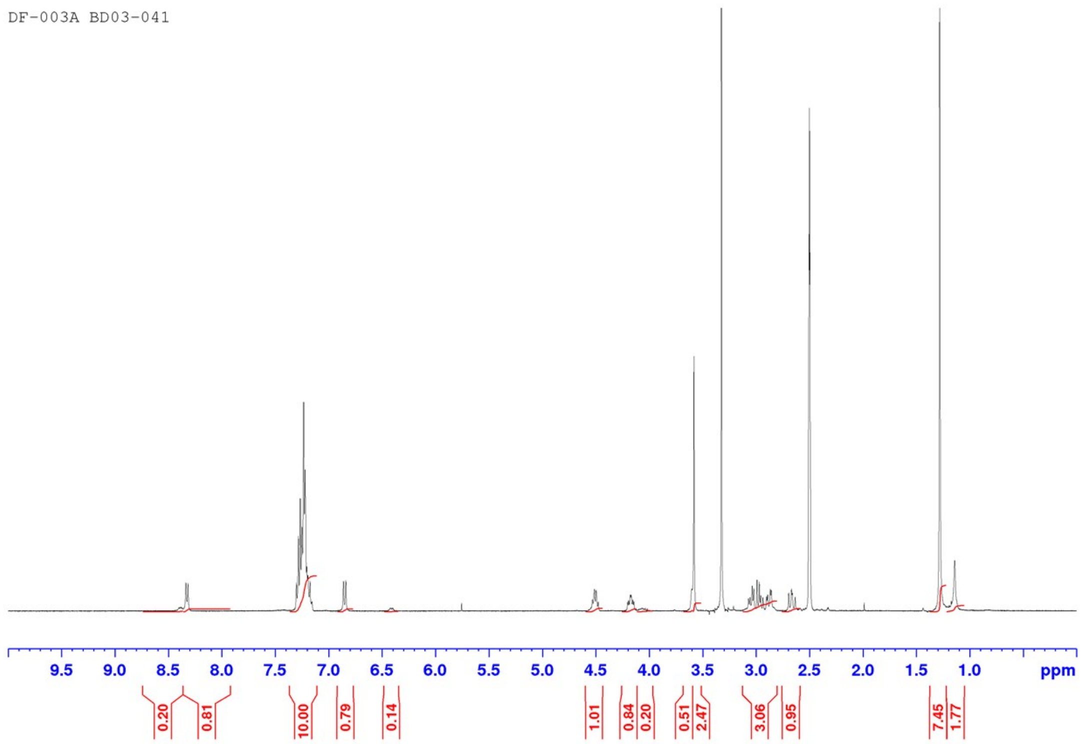

Figure S32. Proton NMR of **DF-003** at 25 °C.

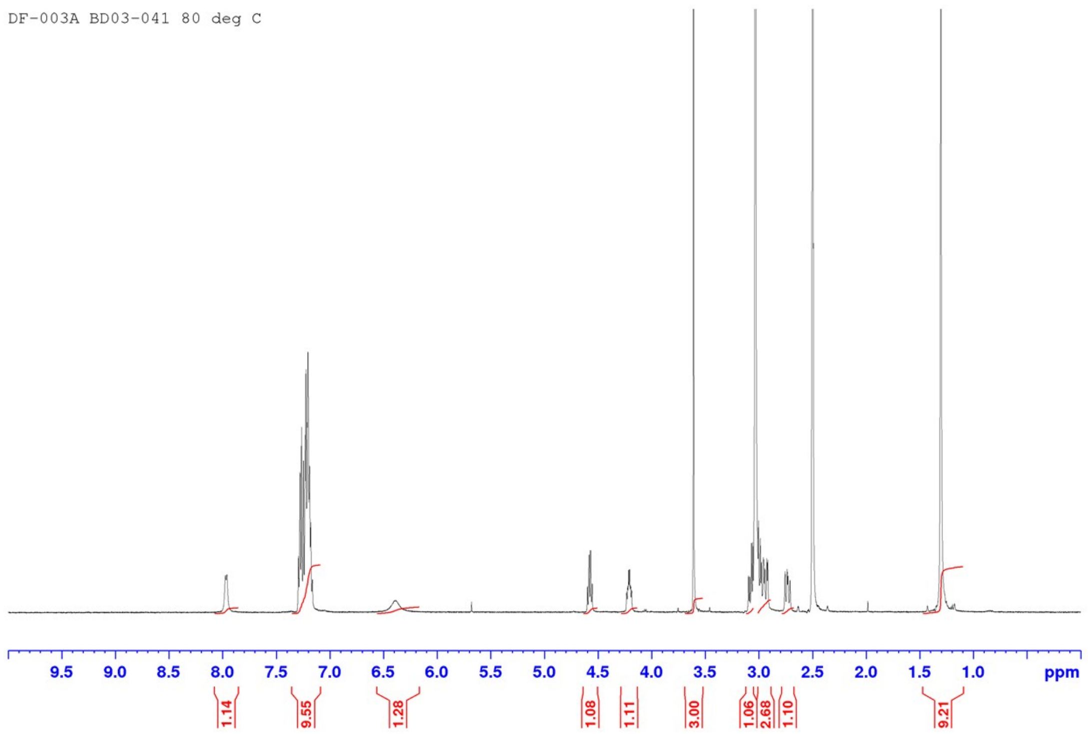

Figure S33. Proton NMR of **DF-003** at 80 °C.

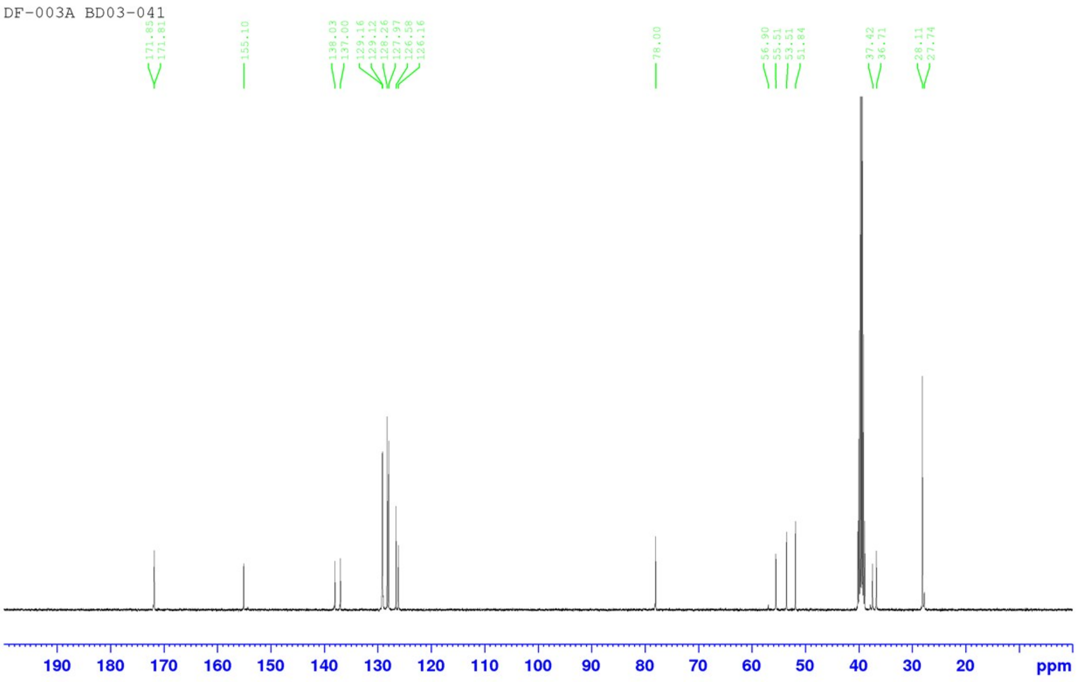

Figure S34. Carbon NMR of **DF-003** at 25 °C.

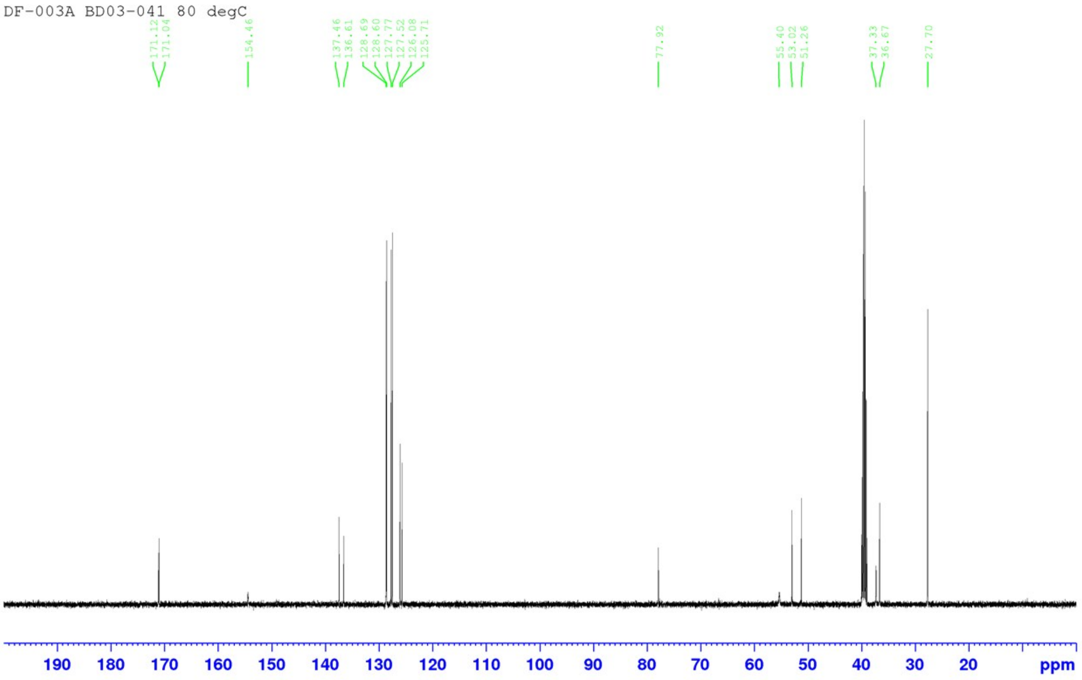

Figure S35. Carbon NMR of **DF-003** at 80 °C.

Methyl (2*R*)-2-[(2*R*)-2-amino-3-phenylpropanamido]-3-phenylpropanoate trifluoroacetic acid salt (DF-004)

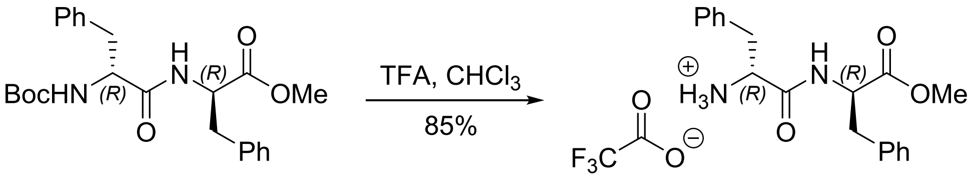

DF-003 **DF-004**

To a solution of **DF-003** (1.65 g, 3.87 mmol) in chloroform (10 mL) was added trifluoroacetic acid (*ca.* 10 eq, 3 mL) and the mixture was stirred at ambient temperature overnight. After this time, TLC indicated the absence of starting material. The reaction mixture was poured into diethyl ether (*ca.* 200 mL) and stirred for 1 hour. The precipitate was filtered off, washed in the filter with additional diethyl ether, and dried under vacuum at 50 °C overnight. The title compound **DF-004** was thus obtained as a white solid (1.45 g, 85%) containing < 0.5% (NMR) residual diethyl ether, which was observed in the proton and carbon NMR spectra.

δ_H (400 MHz, DMSO-d_6) 8.98 (1H, d, *J* 7.64, $N\underline{H}$), 8.10 (3H, br s, NH_3^+), 7.36-7.22 (10H, m, $\underline{H}_{Ar}$), 4.60-4.55 (1H, m, $H_3N^+C\underline{H}^*$), 4.03 (1H, dd, *J* 8.16, 5.28, $HNC\underline{H}^*$), 3.61 (3H, s, $OC\underline{H}_3$), 3.12-3.05 (2H, m, $PhC\underline{H}_2$), 3.00-2.89 (2H, m, $PhC\underline{H}_2$). δ_C (100 MHz, DMSO-d_6) 171.16 ($\underline{C}O_2Me$), 168.27 ($\underline{C}ONH$), 158.23 (q, *J* 31.03, F_3C-$\underline{C}$=O^-), 136.74, 134.76, 129.57, 129.08, 128.49, 128.40, 127.14, and 126.74 ($\underline{C}_{Ar}$), 117.30 (q, *J* 300.92, $\underline{C}F_3$), 53.88 ($H_3N^+\underline{C}H^*$), 53.14 ($NH\underline{C}H^*$), 52.00 ($O\underline{C}H_3$), 36.88 and 36.67 ($Ph\underline{C}H_2$). HRMS (ESI) m/z: [M^+] calcd for $C_{19}H_{23}N_2O_3$ 327.1703; found 327.1699.

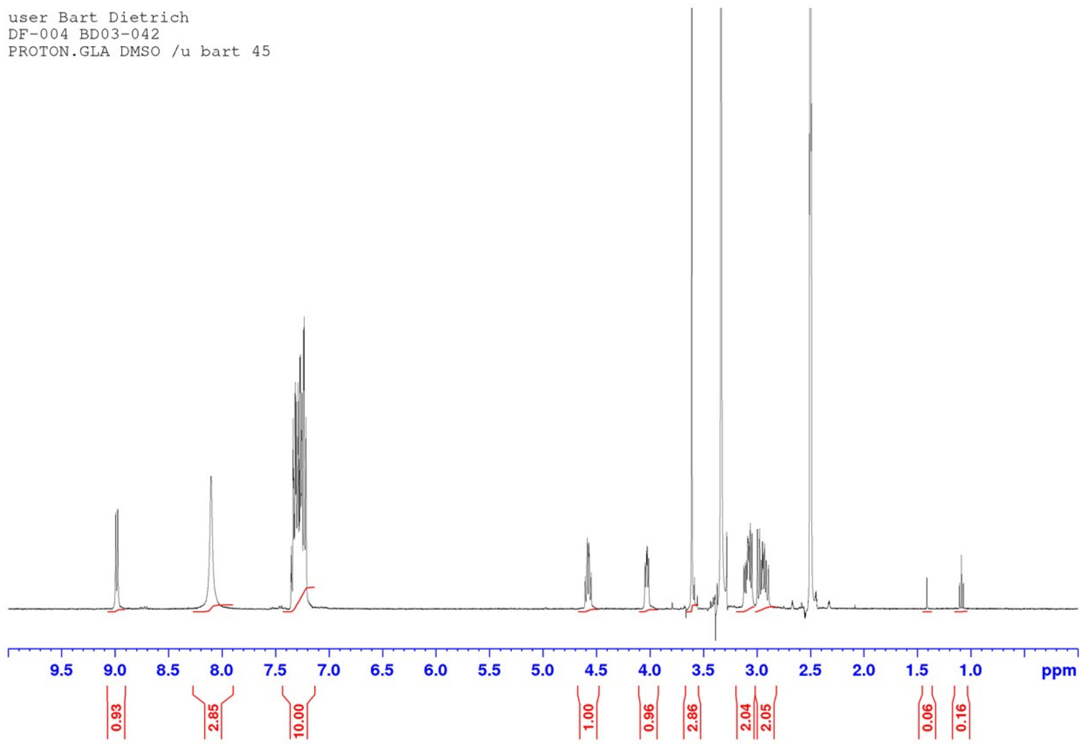

Figure S36. Proton NMR of **DF-004**.

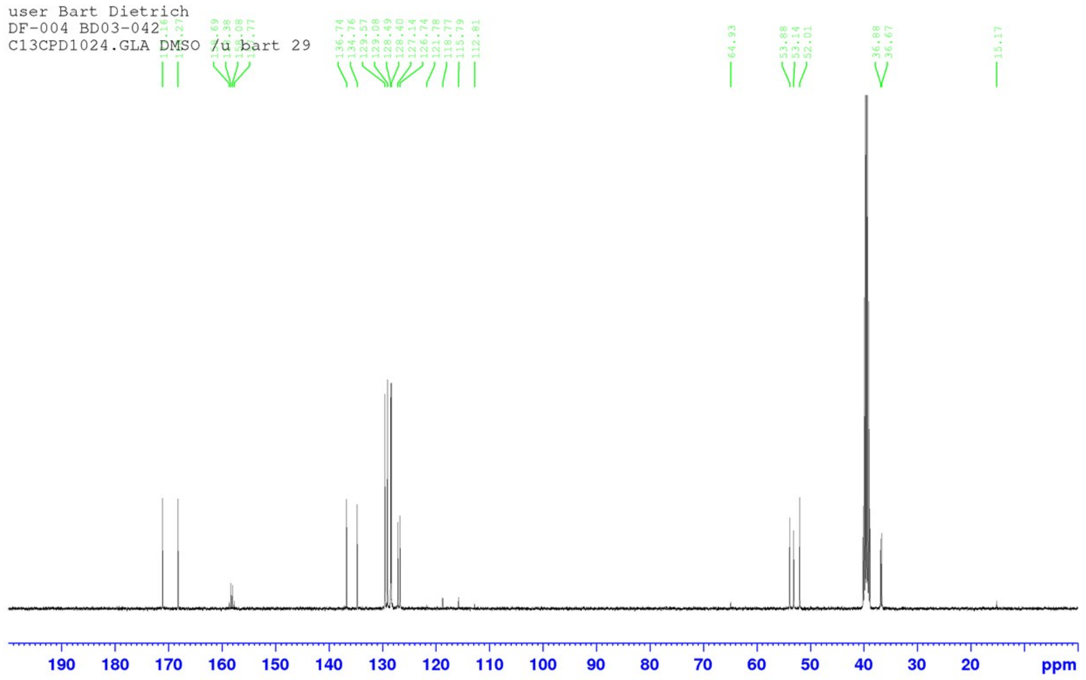

Figure S37. Carbon NMR of **DF-004**.

Methyl (2*R*)-2-[(2*S*)-2-{[(*tert*-butoxy)carbonyl]amino}-3-phenylpropanamido]-3-phenylpropanoate (EE-012)

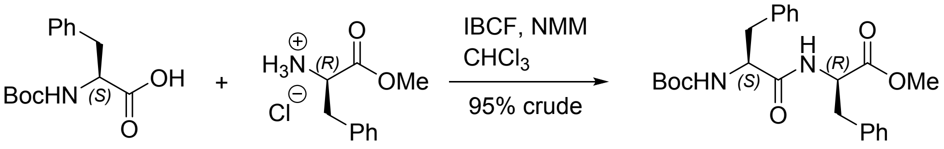

EE-012

To a suspension of *N*-Boc-*L*-phenylalanine (1.07 g, 4.03 mmol) in chloroform (25 mL) was added *iso*butyl chloroformate (1.02 eq, 533 µL) followed by *N*-methylmorpholine (1.1 eq, 487 µL) and the mixture was stirred for 30 minutes. *D*-phenylalanine methyl ester hydrochloride (1 eq, 869 mg) and another portion of *N*-methylmorpholine (1.1 eq, 487 µL) were added and the mixture was stirred overnight. It was diluted with chloroform, washed in turn with 1 M hydrochloric acid, water, and brine, dried ($MgSO_4$), and evaporated under reduced pressure, affording the crude title compound as a white solid (1.63 g). Purification *via* column chromatography (1:9 ethyl acetate/dichloromethane, *ca.* 12×3 cm, wet-loaded) afforded the title compound as a white solid (1.40 g, 81%). The NMR data suggests the presence of a rotameric mixture (*ca* 2:8, denoted Rot-1 and Rot-2 below).

δ_H (400 MHz, DMSO-d$_6$) 8.51 (0.2H, d, *J* 7.60, Rot-1 N<u>H</u>CH*CO_2Me), 8.44 (0.8H, d, *J* 8.12, Rot-2 N<u>H</u>CH*CO_2Me), 7.28-7.13 (10H, m, <u>H</u>$_{Ar}$), 6.75 (0.8H, d, *J* 8.84, Rot-2 N<u>H</u>Boc), 6.33 (0.2H, d, *J* 7.16, Rot-1 N<u>H</u>Boc), 4.62-4.56 (0.2H, m, Rot-1 C<u>H</u>*CO_2Me), 4.52 (0.8H, td, *J* 8.73, 5.14, Rot-2 C<u>H</u>*CO_2Me), 4.17 (0.8H, td, *J* 9.47, 3.98, Rot-2 C<u>H</u>*NHBoc), 4.07-3.98 (0.2H, m, Rot-1 C<u>H</u>*NHBoc), 3.64 (3H, s, OC<u>H</u>$_3$), 3.06 (1H, dd, *J* 13.69, 5.12, PhC<u>H</u>$_a$H$_b$CH*CO_2Me), 2.88 (1H, dd, *J* 13.67, 9.54, PhCH$_a$<u>H</u>$_b$CH*CO_2Me), 2.67 (1H, dd, *J* 13.65, 3.88, PhC<u>H</u>$_a$H$_b$CH*NHBoc), 2.60-2.39 (1H, m, PhCH$_a$<u>H</u>$_b$CH*NHBoc overlapped by DMSO peak), 1.28 (7.2H, s, Rot-2 C(C<u>H</u>$_3$)$_3$), 1.20 (1.8H, s, Rot-1 C(C<u>H</u>$_3$)$_3$). δ_C (100 MHz, DMSO-d$_6$) 171.84, 171.61, and 155.05 (<u>C</u>=O), 138.00, 137.03, 129.17, 129.15, 128.19, 127.88, 126.56, and 126.07 (<u>C</u>$_{Ar}$), 77.91 (<u>C</u>(CH$_3$)$_3$), 56.93 (Rot-1 <u>C</u>H*NHBoc), 55.29 (Rot-2 <u>C</u>H*NHBoc), 53.35 (Rot-2 <u>C</u>H*CO_2Me), 53.20 (Rot-1 <u>C</u>H*CO_2Me), 51.89 (O<u>C</u>H$_3$), 37.52 (Ph<u>C</u>H$_2$CH*NHBoc), 36.96 (Ph<u>C</u>H$_2$CH*CO_2Me), 28.09 (Rot-2 C(<u>C</u>H$_3$)$_3$), 27.72 (Rot-1 C(<u>C</u>H$_3$)$_3$). HRMS (ESI) m/z: $[M+Na]^+$ calcd for $C_{24}H_{30}N_2NaO_5$ 449.2047; found 449.2058.

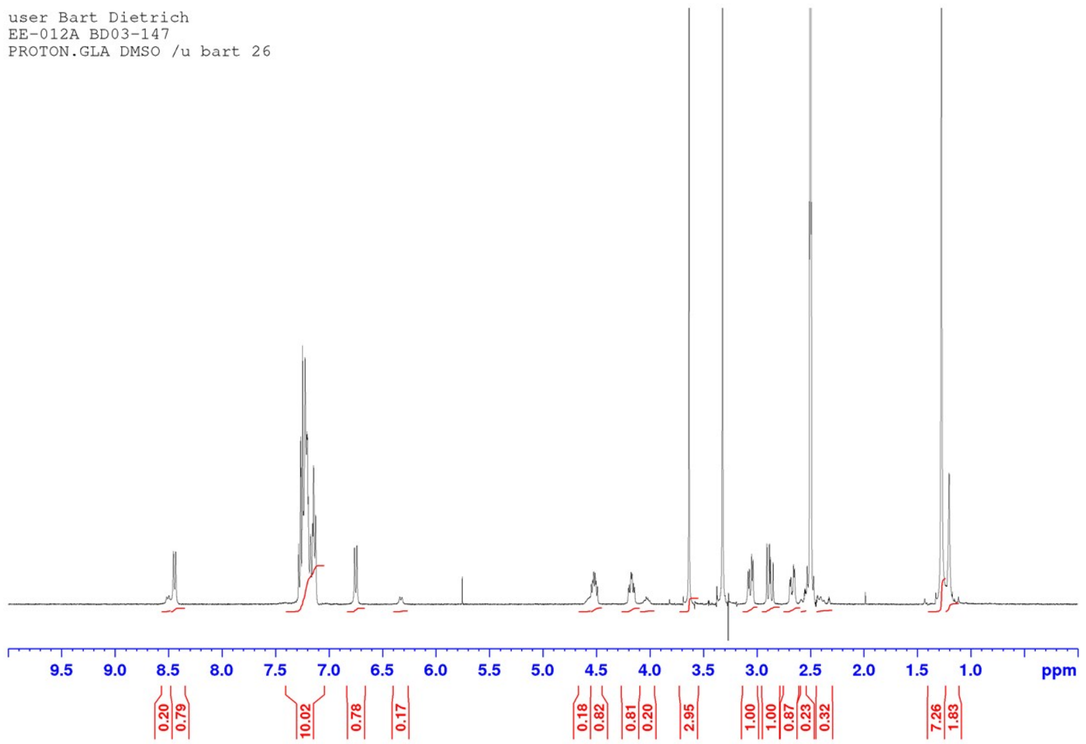

Figure S38. Proton NMR of **EE-012**.

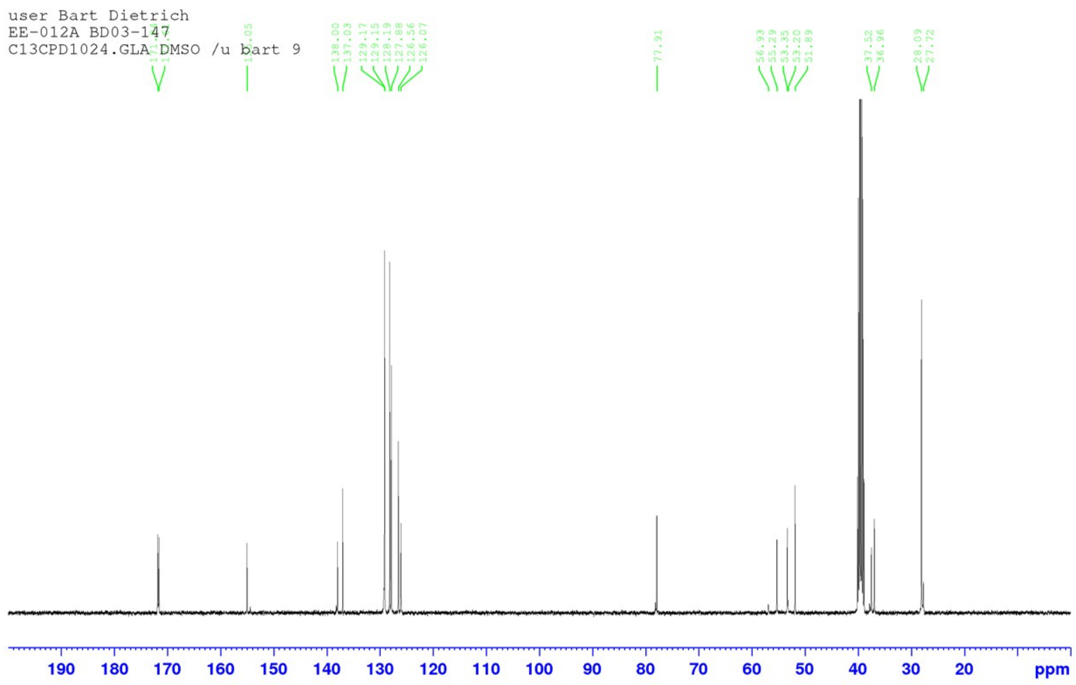

Figure S39. Carbon NMR of **EE-012**.

Methyl (2*R*)-2-[(2*S*)-2-amino-3-phenylpropanamido]-3-phenylpropanoate hydrochloride (EF-001)

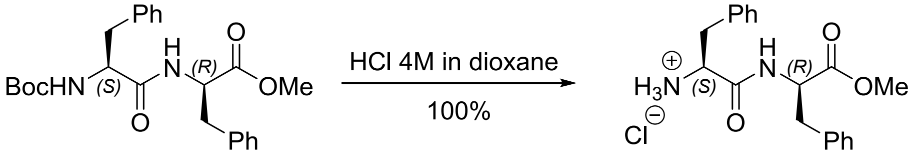

EE-012 **EF-001**

To a solution of **EE-012** (1.36 g, 3.18 mmol) in 1,4-dioxane (15 mL) was added hydrogen chloride 4M in 1,4-dioxane (16 mL, *ca.* 20 eq) and the mixture was stirred overnight. After this time, TLC indicated the absence of starting material. The reaction mixture was evaporated *in vacuo* and then evaporated again from acetonitrile. The title compound was thus obtained as a white foam (1.18 g, 102 %) containing a small amount (1.6%) of dioxane which is seen in the NMR spectra, and not purified any further. The NMR data also suggest the presence of a rotameric mixture (*approx.* 7:93, denoted Rot-1 and Rot-2 below).

δ_H (400 MHz, DMSO-d$_6$) 9.13 (0.93H, d, *J* 8.20, Rot-2 N$\underline{H}$), 8.98 (0.07H, d, *J* 8.24, Rot-1 N$\underline{H}$), 8.12 (3H, br s, N$\underline{H}_3^+$), 7.31-7.20 (8H, m, $\underline{H}_{Ar}$), 7.03-7.01 (2H, m, $\underline{H}_{Ar}$), 4.61-4.55 (1H, m, N$\underline{H}$CH*), 4.05 (1H, dd, *J* 7.60, 5.28, C$\underline{H}^*$NH$_3^+$), 3.65 (3H, s, OC$\underline{H}_3$), 3.04 (1H, dd, *J* 13.71, 5.22, PhC$\underline{H}_a$H$_b$CH*NH), 2.90 (1H, dd, *J* 14.11, 5.14, PhC$\underline{H}_a$H$_b$CH*NH$_3^+$), 2.83 (1H, dd, *J* 13.75, 9.50, PhCH$_a\underline{H}_b$CH*NH), 2.71 (1H, dd, *J* 14.03, 7.78, PhCH$_a\underline{H}_b$CH*NH$_3^+$). δ_C (100 MHz, DMSO-d$_6$, not all carbons resolved) 171.39 and 167.96 ($\underline{C}$=O), 136.90, 134.63, 129.62, 129.24, 128.31, 126.96, and 126.74 ($\underline{C}_{Ar}$), 53.82 (NH$\underline{C}$H*), 53.07 ($\underline{C}$H*NH$_3^+$), 52.03 (O$\underline{C}$H$_3$), 36.83 and 36.60 (Ph$\underline{C}$H$_2$). HRMS (ESI) m/z: [M+Na]$^+$ calcd for $C_{19}H_{22}N_2NaO_3$ 349.1523; found 349.1514.

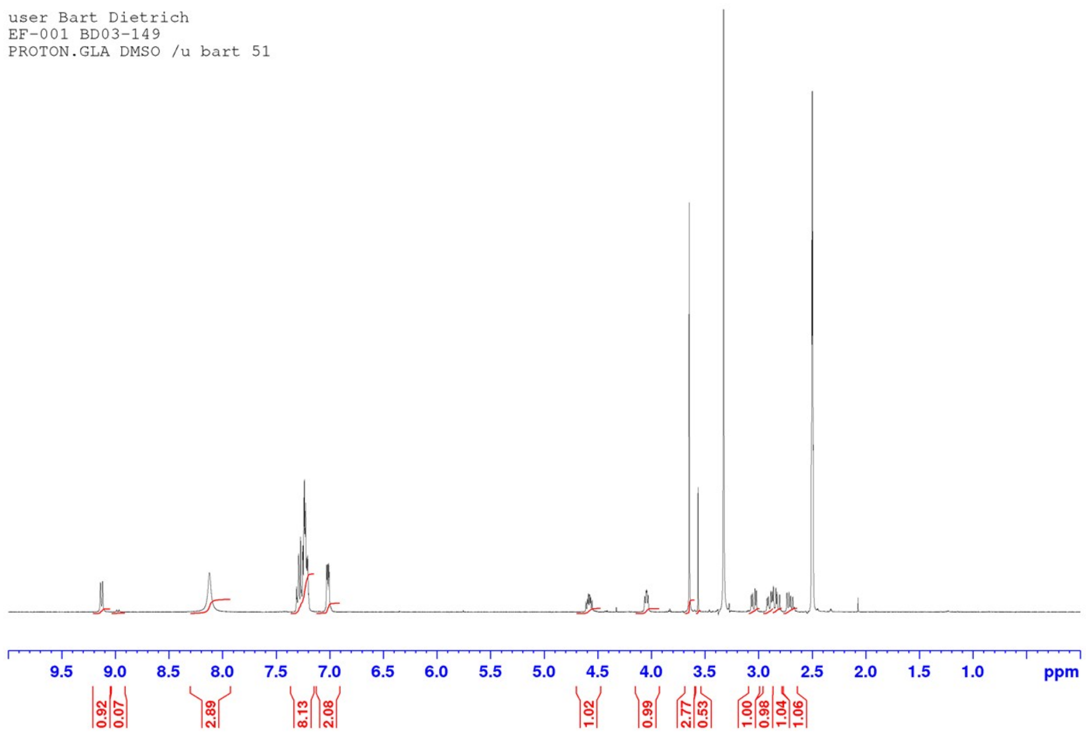

Figure S40. Proton NMR of **EF-001**.

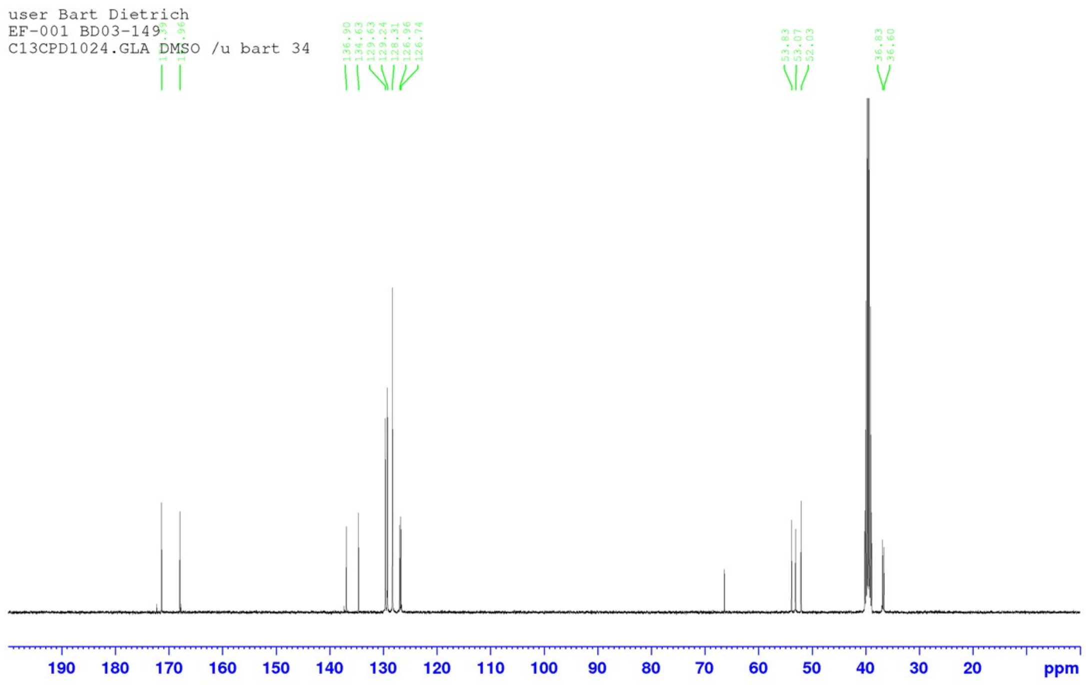

Figure S41. Carbon NMR of **EF-001**.

Ethyl (2*S*)-2-[(2*R*)-2-{[(*tert*-butoxy)carbonyl]amino}-3-phenylpropanamido]-3-phenylpropanoate (ED-008)

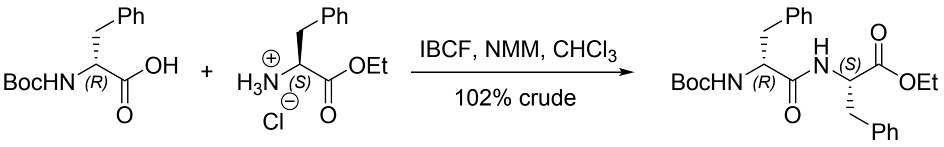

ED-008

To a solution of *N*-Boc-*D*-phenylalanine (1.93 g, 7.27 mmol) in chloroform (40 mL) was added *N*-methylmorpholine (1.1 eq, 879 μL) followed by *iso*butyl chloroformate (1.01 eq, 952 μL) and the mixture was stirred for 30 minutes. After this time, another portion of *N*-methylmorpholine (1.1 eq, 879 μL) was added, followed by *L*-phenylalanine ethyl ester hydrochloride (1 eq, 1.67 g) (effervescence is observed) and the reaction was stirred overnight. The reaction mixture was diluted with chloroform and washed in turn with 1M hydrochloric acid, water, and brine, dried ($MgSO_4$), and evaporated under reduced pressure. The title compound was obtained in 102% crude yield (3.28 g) as a pale-yellow oil, which solidified on standing. This was used without further purification in the next step. A small amount was purified *via* column chromatography (eluting with 1:99 ethyl acetate/dichloromethane) to afford an analytical sample. Proton NMR data indicates a *ca.* 2:8 rotameric mixture, with some carbon signals also doubled due to the presence of rotamers. The rotamers are denoted Rot-1 and Rot-2 below.

δ_H (400 MHz, DMSO-d_6) 8.48 (0.2H, d, *J* 7.88, Rot-1 N$\underline{H}$C$_a$H*), 8.42 (0.8H, d, *J* 8.00, Rot-2 N$\underline{H}$C$_a$H*), 7.28-7.14 (10H, m, $\underline{H}_{Ar}$), 6.77 (0.8H, d, *J* 8.80, Rot-2 N$\underline{H}$C$_b$H*), 6.33 (0.2H, d, *J* 8.52, Rot-1 N$\underline{H}$C$_b$H*), 4.59-4.52 (0.2H, m, Rot-1 NHC$_a\underline{H}^*$), 4.50-4.45 (0.8H, m, Rot-2 NHC$_a\underline{H}^*$), 4.18 (1H, td, *J* 9.45, 4.10, NHC$_b\underline{H}^*$), 4.08 (2H, q, *J* 7.10, C$\underline{H}_2$CH$_3$), 3.04 (1H, dd, *J* 13.59, 5.38, NHC$_a$H*C$\underline{H}_m$H$_n$Ph), 2.88 (1H, dd, *J* 13.57, 9.32, NHC$_a$H*CH$_m\underline{H}_n$Ph), 2.69 (1H, dd, *J* 13.65, 3.84, NHC$_b$H*C$\underline{H}_m$H$_n$Ph), 2.62-2.53 (1H, m, NHC$_b$H*CH$_m\underline{H}_n$Ph), 1.28 (7.2H, s, Rot-2 C(C$\underline{H}_3$)$_3$), 1.20 (1.8H, s, Rot-1 C(C$\underline{H}_3$)$_3$), 1.14 (3H, t, *J* 7.10, CH$_2$C$\underline{H}_3$). δ_C (100 MHz, DMSO-d_6) 171.66, 171.34, and 155.08 ($\underline{C}$=O), 138.03, 137.03, 129.20, 129.16, 128.17, 127.89, 126.55, and 126.09 ($\underline{C}_{Ar}$), 77.90 ($\underline{C}$(CH$_3$)$_3$), 60.58 ($\underline{C}$H$_2$CH$_3$), 55.35 (NH$\underline{C}_b$H*), 53.47 (NH$\underline{C}_a$H*), 37.55 (NHC$_b$H$^*\underline{C}$H$_2$Ph), 36.98 (NHC$_a$H$^*\underline{C}$H$_2$Ph), 28.09 (C($\underline{C}$H$_3$)$_3$), 13.92 (CH$_2\underline{C}$H$_3$). HRMS (ESI) m/z: $[M+Na]^+$ calcd for $C_{25}H_{32}N_2NaO_5$ 463.2203; found 463.2186.

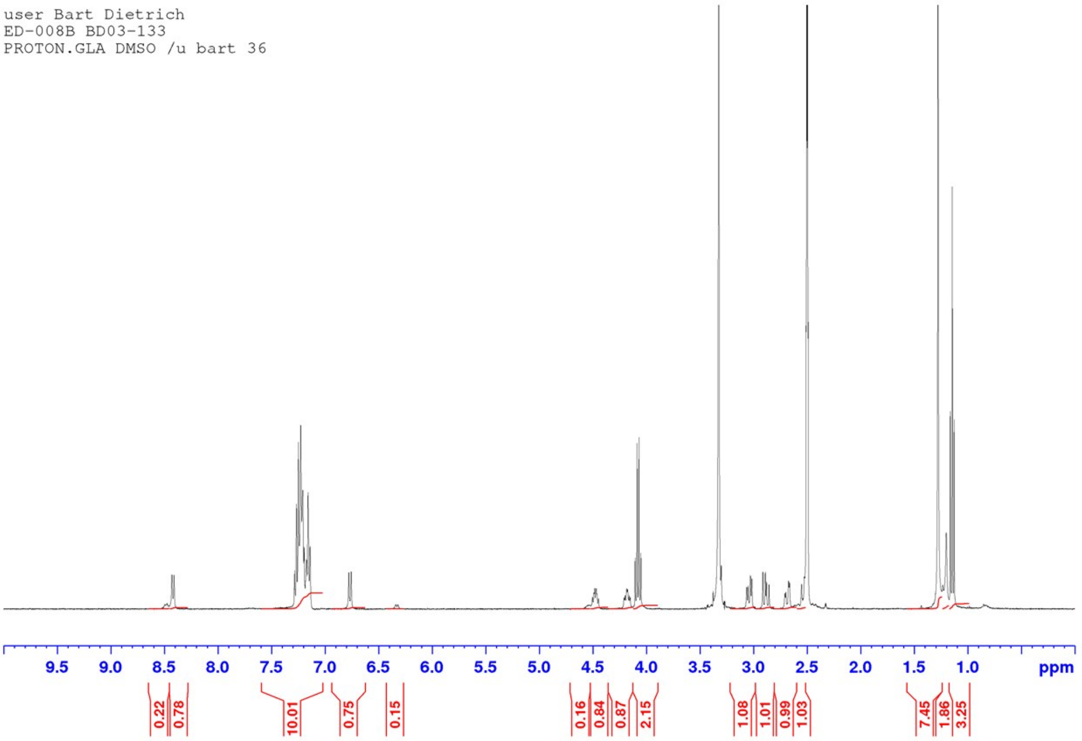

Figure S42. Proton NMR of **ED-008**.

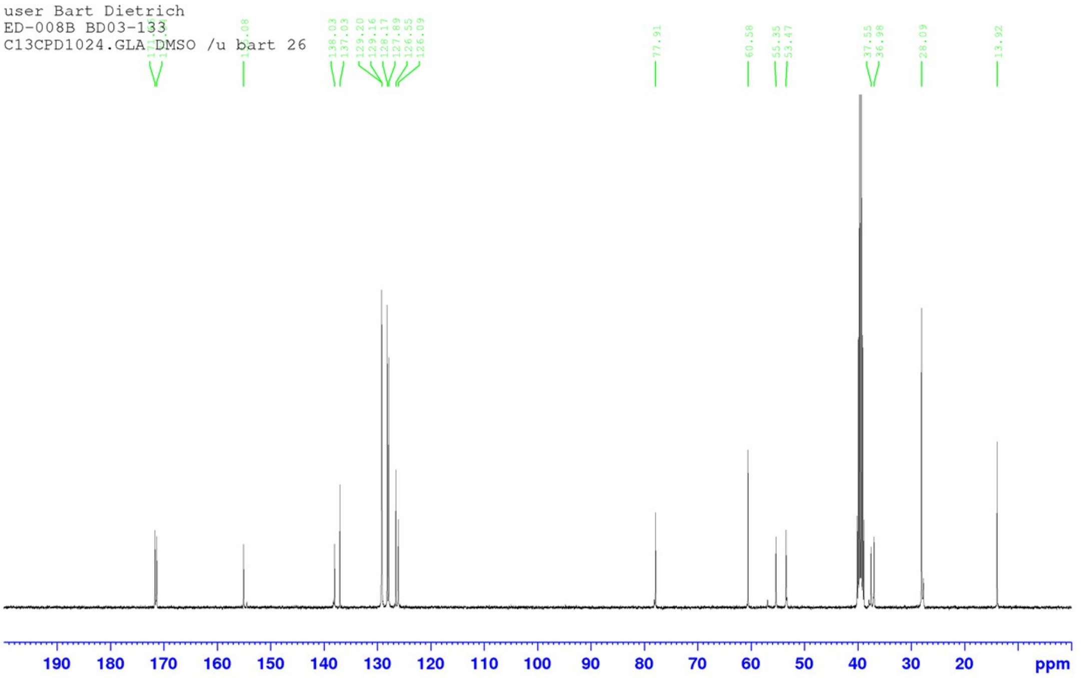

Figure S43. Carbon NMR of **ED-008**.

Ethyl (2*S*)-2-[(2*R*)-2-amino-3-phenylpropanamido]-3-phenylpropanoate trifluoroacetate (ED-009)

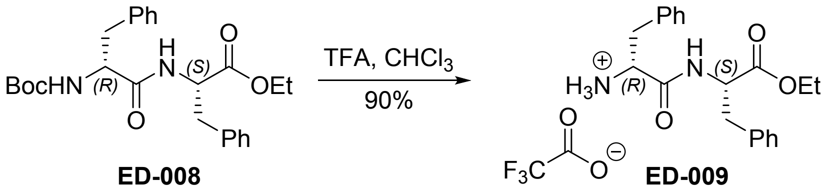

To a solution of **ED-008** (3.09 g, 7.02 mmol) in chloroform (20 mL) was added trifluoroacetic acid (10 mL, *ca.* 20 eq) and the mixture was stirred for 40 hours. After this time, it was poured into diethyl ether, which failed to result in the expected precipitate of title compound. *Iso*-octane was added and the mixture evaporated under reduced pressure, affording a sticky yellow oil still containing trifluoroacetic acid. This was dissolved in a small amount of diethyl ether, poured into *n*-hexane and left to sediment overnight. The supernatant (which contained the bulk of excess trifluoroacetic acid) was discarded and the sticky residue dissolved in dichloromethane and evaporated under reduced pressure. The title compound was thus obtained as a white foam (2.87 g, 90%) and not purified any further. Traces of diethyl ether are observed in the NMR spectra.

δ_H (400 MHz, DMSO-d$_6$) 9.03 (1H, d, *J* 8.16, N$\underline{H}$), 8.12 (3H, br s, N$\underline{H}_3^+$), 7.31-7.19 (8H, m, $\underline{H}_{Ar}$), 7.04-7.02 (2H, m, $\underline{H}_{Ar}$), 4.60-4.54 (1H, m, EtO$_2$C-C$\underline{H}^*$), 4.09 (2H, q, *J* 7.10, C$\underline{H}_2$CH$_3$), 4.10-4.03 (1H, br, H$_3$N$^+$-C$\underline{H}^*$), 3.02 (1H, dd, *J* 13.69, 5.60, EtO$_2$C-CH*-C$\underline{H}_a$H$_b$Ph), 2.88 (1H, dd, *J* 14.03, 5.14, H$_3$N$^+$-CH*-C$\underline{H}_a$H$_b$Ph), 2.82 (1H, dd, *J* 13.67, 9.22, EtO$_2$C-CH*-CH$_a\underline{H}_b$Ph), 2.70 (1H, dd, *J* 14.05, 7.92, H$_3$N$^+$-CH*-CH$_a\underline{H}_b$Ph), 1.15 (3H, t, *J* 7.10, CH$_2$C$\underline{H}_3$). δ_C (100 MHz, DMSO-d$_6$) 170.93 and 168.00 ($\underline{C}$=O), 158.24 (1C, q, *J* 31.51, F$_3$C-$\underline{C}$=O), 136.70, 134.59, 129.52, 129.23, 128.44, 128.33, 127.10, and 126.77 ($\underline{C}_{Ar}$), 117.16 (1C, q, *J* 299.16, $\underline{C}$F$_3$), 60.83 (H$_3$N$^+$-$\underline{C}$H*), 53.68 (EtO$_2$C-$\underline{C}$H*), 53.14 ($\underline{C}$H$_2$CH$_3$), 37.10 (EtO$_2$C-CH*-$\underline{C}$H$_2$Ph), 36.91 (H$_3$N$^+$-CH*-$\underline{C}$H$_2$Ph), 13.93 (CH$_2\underline{C}$H$_3$). HRMS (ESI) m/z: [M-TFA+Na]$^+$ calcd for $C_{20}H_{24}N_2NaO_3$ 363.1679; found 363.1671.

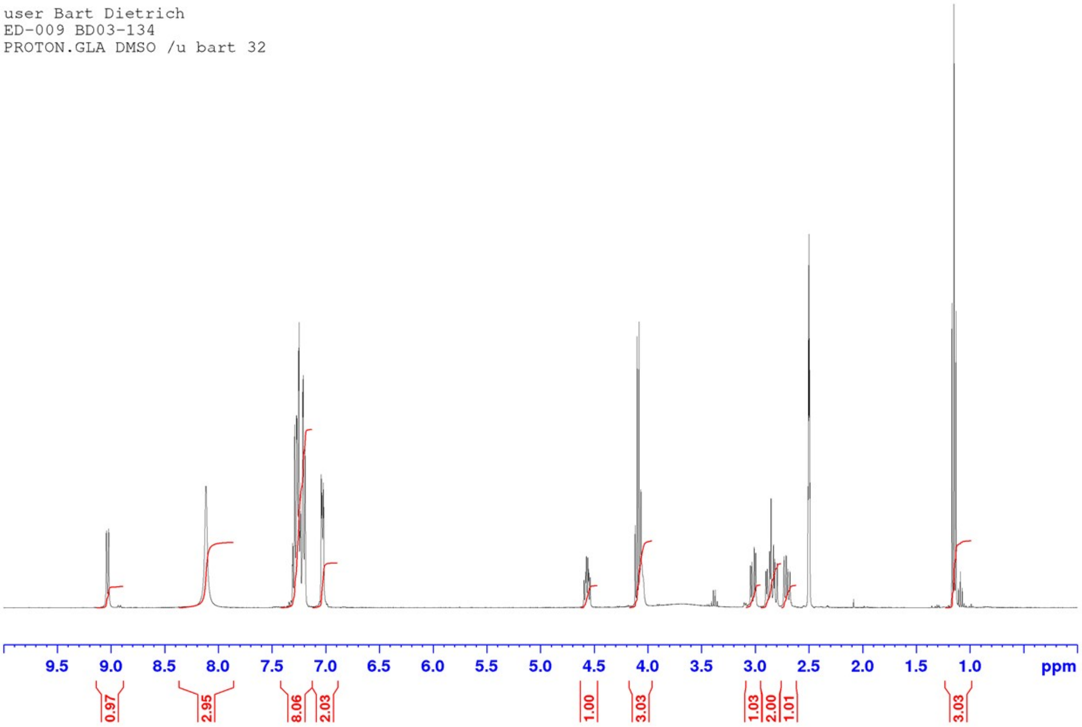

Figure S44. Proton NMR of **ED-009**.

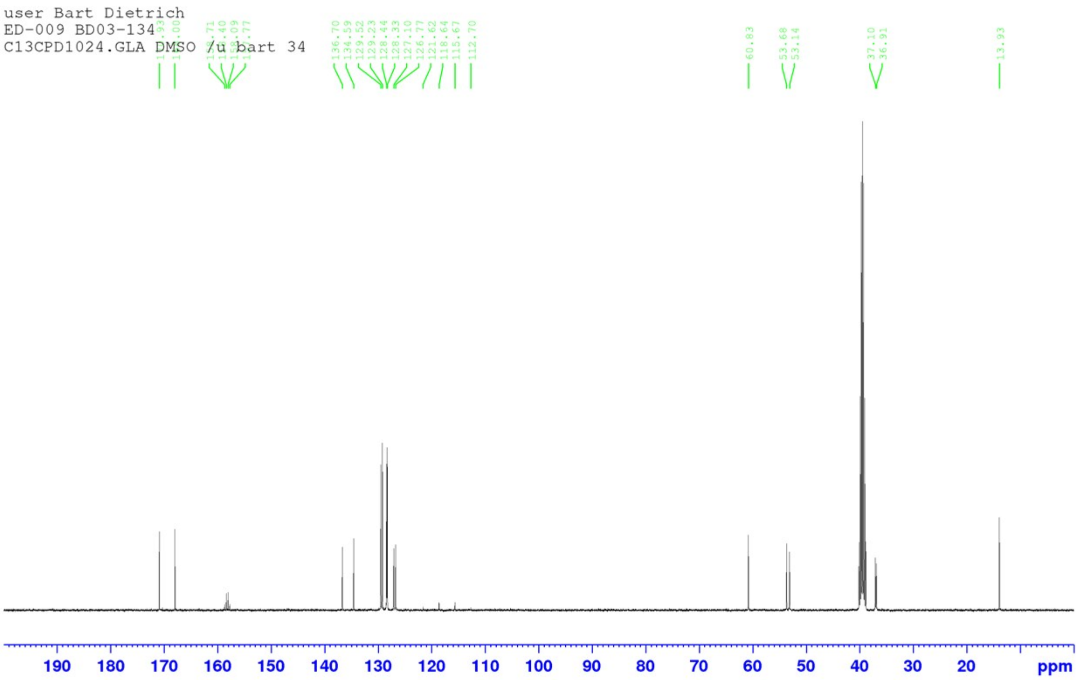

Figure S45. Carbon NMR of **ED-009**.

<u>Ethyl 2-(2-{[(*tert*-butoxy)carbonyl]amino}-3-phenylpropanamido)-3-phenylpropanoate (ED-006)</u>

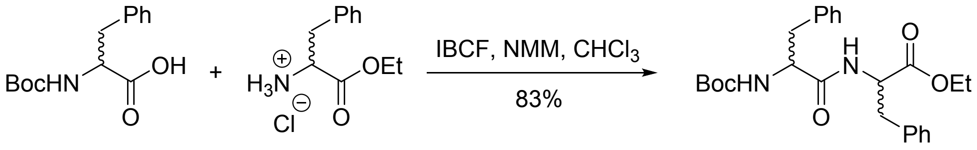

ED-006

To a suspension of *N*-Boc-*DL*-phenylalanine (1.24 g, 4.67 mmol) in chloroform (30 mL) was added *iso*butyl chloroformate (1.02 eq, 618 μL) and *N*-methylmorpholine (1.1 eq, 565 μL) and the mixture was stirred for 30 minutes. After this time, *DL*-phenylalanine ethyl ester hydrochloride (1 eq, 1.07 g) and another portion of *N*-methylmorpholine (1.1 eq, 565 μL) were added and the mixture stirred overnight. After this time, it was diluted with chloroform, washed in turn with 1 M hydrochloric acid, saturated aqueous sodium carbonate, water, and brine, dried ($MgSO_4$), filtered and evaporated under reduced pressure. The crude material was thus obtained as a white solid. Purification *via* column chromatography (1:99 ethyl acetate/dichloromethane, *ca.* 10×3.5 cm, wet-loaded) afforded the title compound as a white solid (1.71 g, 83%). The NMR spectra obtained for the material are complex due to the presence of diastereoisomers but identical to overlays of the corresponding spectra of the (*S*,*S*)/(*R*,*R*) and (*S*,*R*)/(*R*,*S*) analogues. Additionally, proton NMR suggests the presence of rotational isomers.

δ_H (400 MHz, DMSO-d_6) 8.48 (0.1H, d, *J* 7.96, N<u>H</u>), 8.42 (0.4H, d, *J* 8.08, N<u>H</u>), 8.39-8.33 (0.1H, m, N<u>H</u>), 8.32 (0.4H, d, *J* 7.60, N<u>H</u>), 7.30-7.14 (10H, m, $\underline{H}_{Ar}$), 6.85 (0.4H, d, *J* 8.76, N<u>H</u>), 6.77 (0.4H, d, *J* 8.76, N<u>H</u>), 6.41 (0.1H, d, *J* 8.08, N<u>H</u>), 6.33 (0.1H, d, *J* 7.16, N<u>H</u>), 4.60-4.45 (1H, m, C$\underline{H}^*$), 4.21-4.15 (0.8H, m, C$\underline{H}^*$), 4.11-4.00 (2.2H, C$\underline{H}^*$ and C$\underline{H}_2$CH$_3$), 3.06-2.95 (1.5H, m, PhC$\underline{H}_2$), 2.91-2.86 (1H, m, PhC$\underline{H}_2$), 2.70-2.55 (1.5H, m, PhC$\underline{H}_2$), 1.28 (7.2H, s, C(C$\underline{H}_3$)$_3$), 1.20 (1H, s, C(C$\underline{H}_3$)$_3$), 1.16-1.13 (2.3H, m, C(C$\underline{H}_3$)$_3$ and CH$_2$C$\underline{H}_3$), 1.09 (1.5H, t, *J* 7.10, CH$_2$C$\underline{H}_3$). δ_C (100 MHz, DMSO-d_6) 171.80, 171.63, 171.32, 171.25, and 155.06 (<u>C</u>=O), 138.03, 137.02, 136.97, 129.19, 129.12, 128.20, 128.16, 127.94, 127.88, 126.53, 126.12, and 126.07 ($\underline{C}_{Ar}$), 77.96 and 77.89 (<u>C</u>(CH$_3$)$_3$), 60.57 and 60.48 (<u>C</u>H$_2$CH$_3$), 55.44, 55.32, 53.54, and 53.44 (<u>C</u>H*), 37.50, 37.41, 36.94, and 36.74 (Ph<u>C</u>H$_2$), 28.08 and 27.73 (C(<u>C</u>H$_3$)$_3$), 13.91 and 13.88 (CH$_2$<u>C</u>H$_3$). HRMS (ESI) m/z: $[M+Na]^+$ calcd for $C_{25}H_{32}N_2NaO_5$ 463.2203; found 463.2180.

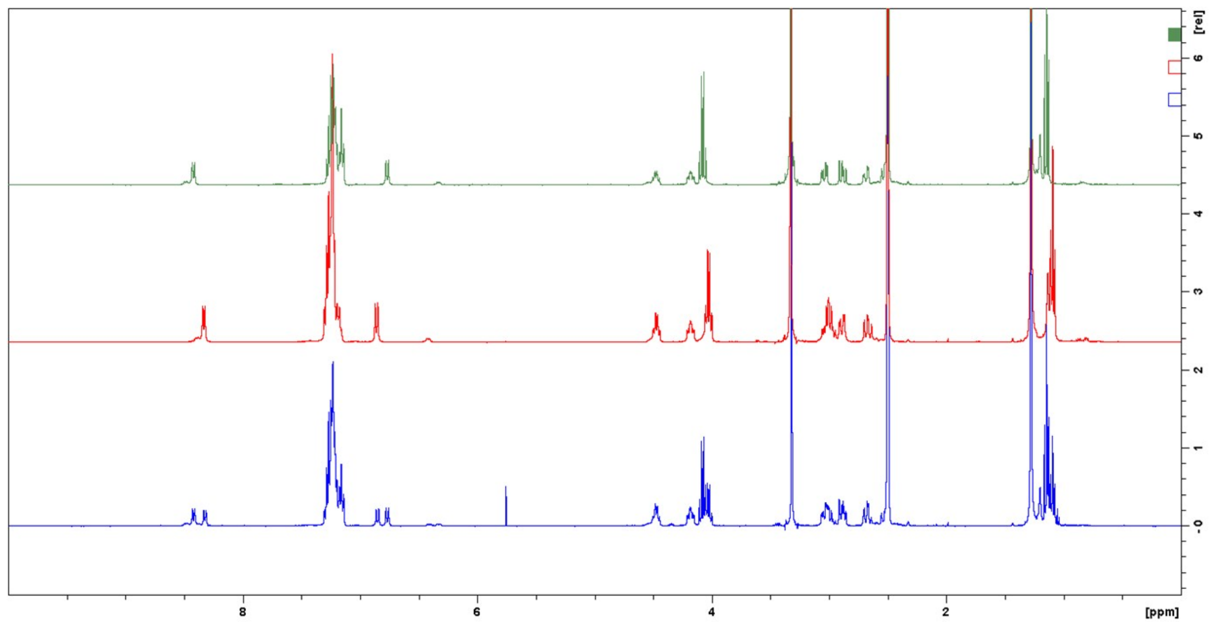

Figure S46. Overlay of the proton NMR spectra of stereoisomers of **ED-006**: top: (*D*,*L*) (**ED-008**), middle: (*L*,*L*) (**DH-002**), bottom: (*rac*) (**ED-006**).

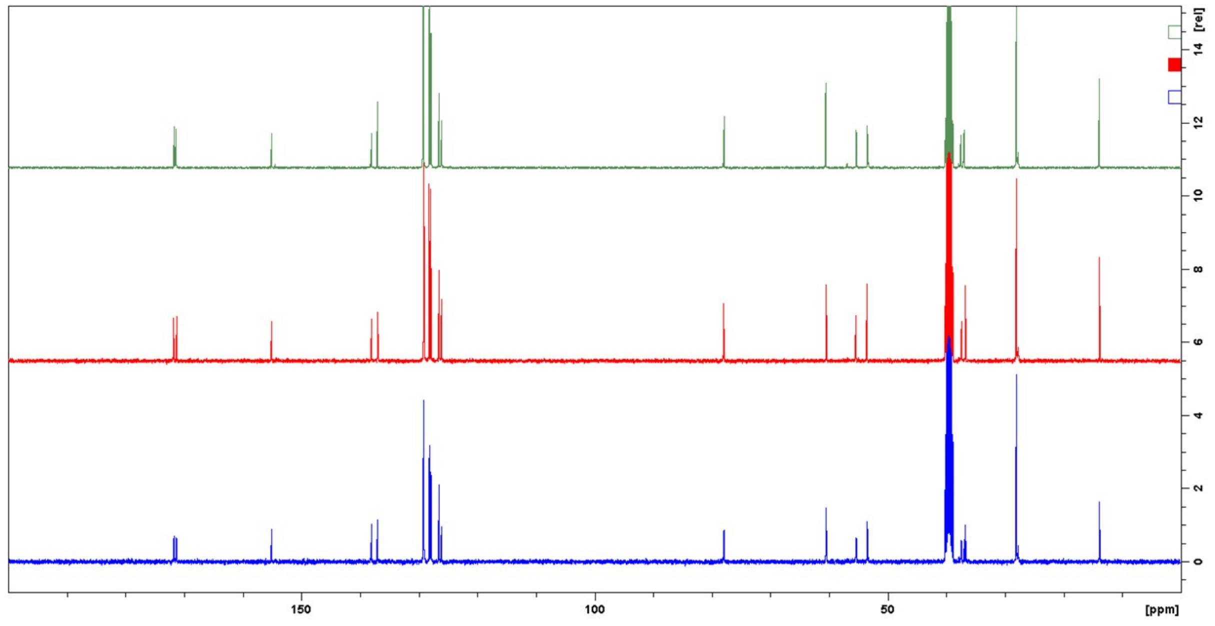

Figure S47. Overlay of the carbon NMR spectra of stereoisomers of **ED-006**: top: (D,L) (**ED-008**), middle: (*L*,*L*) (**DH-002**), bottom: (*rac*) (**ED-006**).

Ethyl 2-(2-amino-3-phenylpropanamido)-3-phenylpropanoate (EE-005)

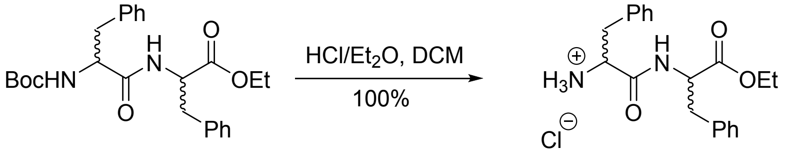

ED-006 **EE-005**

To a solution of **ED-006** (1.66 g, 3.76 mmol) in dichloromethane (15 mL) was added a solution of hydrogen chloride in diethyl ether (1.0 M, 20 mL, *ca.* 5.3 eq) and the mixture was stirred overnight. After this time, TLC still indicated the presence of unreacted starting material. Another 10 mL of hydrogen chloride in diethyl ether were added and stirring continued overnight. After this time, TLC indicated the absence of starting material. The reaction mixture was evaporated under reduced pressure, affording the title compound as a white foam (1.42 g, 100%). NMR data is complex due to the presence of a mixture of diastereomers and rotamers.

δ_H (400 MHz, DMSO-d_6) 9.14 (0.45H, d, *J* 7.44, N<u>H</u>), 9.10 (0.45H, d, *J* 8.12, N<u>H</u>), 8.99 (0.1H, d, *J* 8.60, N<u>H</u>), 8.16 (3H, br s, $N\underline{H_3}^+$), 7.34-7.20 (9H, m, $\underline{H}_{Ar}$), 7.05-6.98 (1H, m, $\underline{H}_{Ar}$), 4.57-4.50 (1H, m, $C\underline{H}^*$), 4.11-4.02 (3H, m, $C\underline{H}^*$ and $C\underline{H_2}CH_3$), 3.18-2.71 (4H, m, $PhC\underline{H_2}$), 1.15 (1.5H, t, *J* 7.12, $CH_2C\underline{H_3}$), 1.11 (1.5H, t, *J* 7.10, $CH_2C\underline{H_3}$). δ_C (100 MHz, DMSO-d_6) 170.91, 170.70, 168.16, and 167.93 ($\underline{C}$=O), 136.84, 136.83, 134.92, 134.66, 129.69, 129.62, 129.25, 129.20, 128.35, 128.30, 127.01, 126.97, 126.73, and 126.65 ($\underline{C}_{Ar}$), 60.75 and 60.66 ($\underline{C}H_2CH_3$), 54.11, 53.88, 53.14, and 53.06 ($\underline{C}H^*$), 36.95 and 36.66 ($Ph\underline{C}H_2$), 13.95 and 13.91 ($CH_2\underline{C}H_3$). HRMS (ESI) m/z: $[M-HCl+Na]^+$ calcd for $C_{20}H_{24}N_2NaO_3$ 363.1679; found 363.1664.

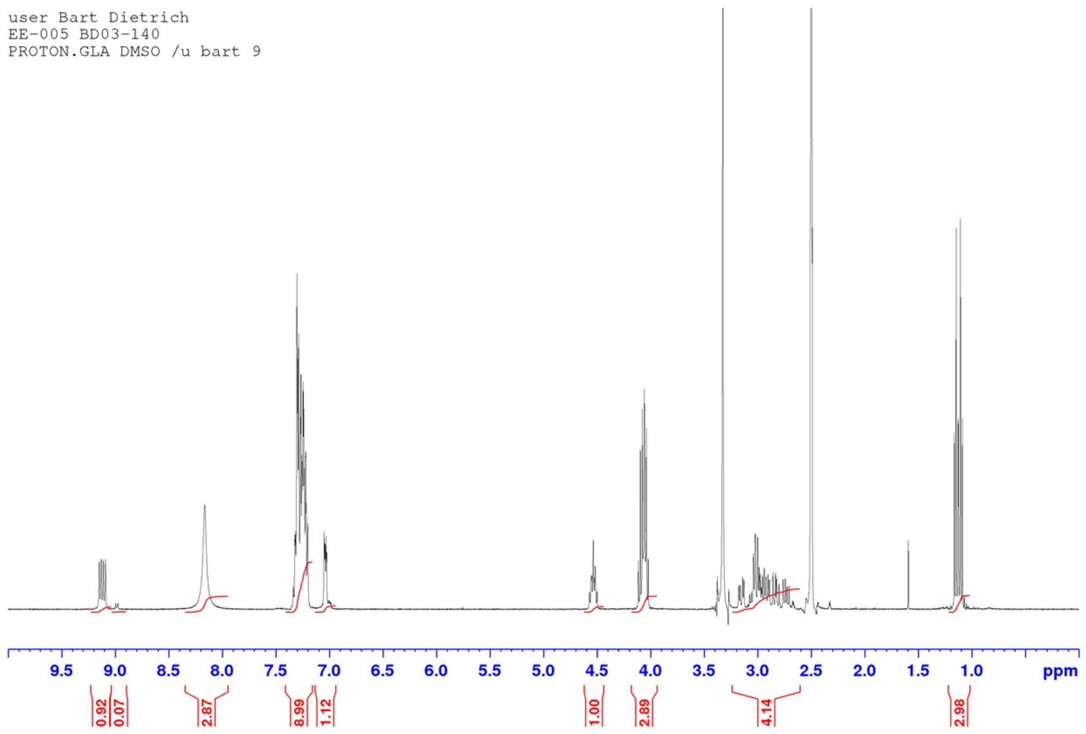

Figure S48. Proton NMR of **EE-005**.

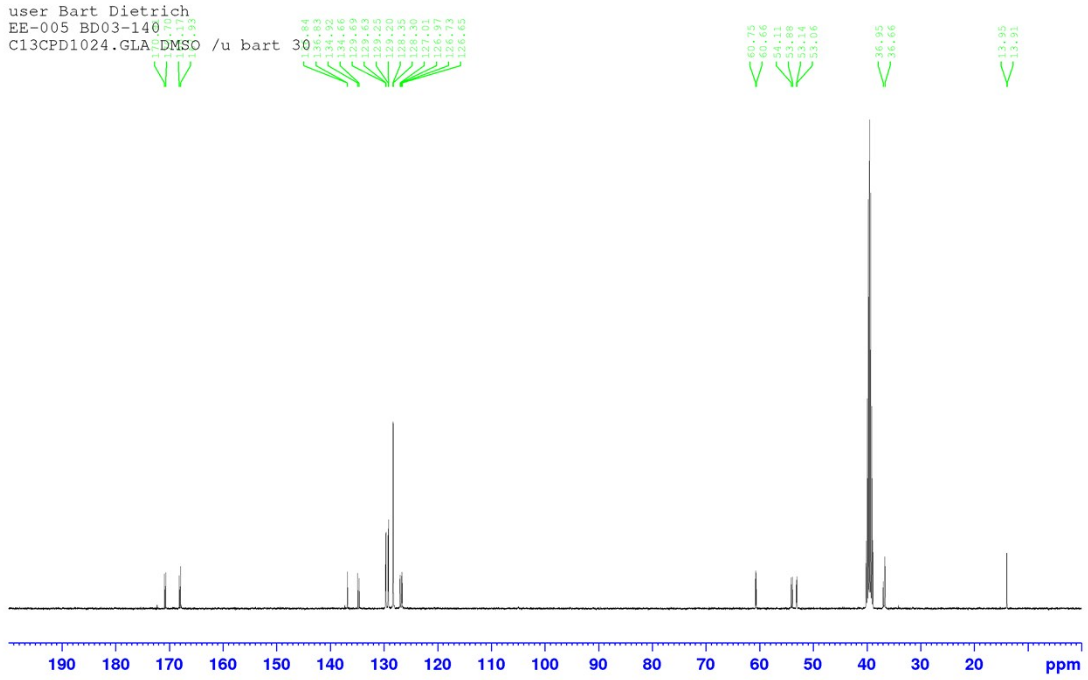

Figure S49. Carbon NMR of **EE-005**.

Ethyl (2*S*)-2-[(2*S*)-2-[2-(naphthalen-2-yloxy)acetamido]-3-phenylpropanamido]-3-phenylpropanoate (DK-001)

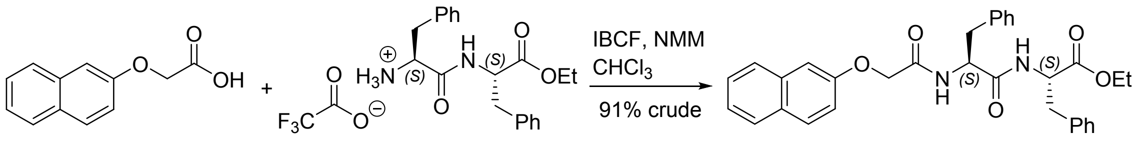

DG-001 **DH-003** **DK-001**

To a suspension of **DG-001** (3.03 g, 15.0 mmol) in chloroform (50 mL) were added *iso*-butyl chloroformate (1 eq, 1.95 mL) and *N*-methylmorpholine (1 eq, 1.65 mL) and the mixture was stirred for one hour. A solution of **DH-003** (1 eq, 6.82 g) and *N*-methylmorpholine (1 eq, 1.65 mL) in chloroform (50 mL) was then added and the reaction was stirred for 3 days at room temperature. After this time, it was diluted with chloroform and washed in turn with 1M hydrochloric acid, saturated aqueous sodium carbonate solution, water, and brine. After drying over magnesium sulfate and evaporation *in vacuo*, the crude title compound was obtained as a light-brown solid (7.20 g, 91 %). This was used in the next step without further purification. A small amount was purified *via* column chromatography (eluting with 1:99 ethyl acetate/dichloromethane) to yield a sample suitable for characterisation.

δ_H (400 MHz, DMSO-d$_6$) 8.61 (1H, d, *J* 7.48, N$\underline{H}$), 8.16 (1H, d, *J* 8.56, N$\underline{H}$), 7.85-7.82 (2H, m, $\underline{H}_{Ar}$), 7.73 (1H, d, *J* 8.08, $\underline{H}_{Ar}$), 7.46 (1H, ddd, *J* 8.09, 6.91, 1.15, $\underline{H}_{Ar}$), 7.36 (1H, ddd, *J* 8.04, 6.92, 1.12, $\underline{H}_{Ar}$), 7.28-7.12 (12H, m, $\underline{H}_{Ar}$), 4.66 (1H, td, *J* 9.04, 4.20, C$\underline{H}^*$), 4.54 (2H, s, OC$\underline{H}_2$), 4.51-4.46 (1H, m, C$\underline{H}^*$), 4.04 (2H, q, *J* 7.11, C$\underline{H}_2$CH$_3$), 3.05-2.93 (3H, m, PhC$_a\underline{H}_2$ and PhC$_b\underline{H}_a$H$_b$), 2.85 (1H, dd, *J* 13.86, 9.70, PhC$_b$H$_a\underline{H}_b$), 1.09 (3H, t, *J* 7.10, CH$_2$C$\underline{H}_3$). δ_C (100 MHz, DMSO-d$_6$) 171.19, 170.95, and 167.23 ($\underline{C}$=O), 155.48, 137.45, 136.93, 134.01, 129.32, 129.17, 129.07, 128.73, 128.20, 127.96, 127.47, 126.76, 126.53, 126.38, 126.26, 123.82, 118.43, and 107.32 ($\underline{C}_{Ar}$), 66.68 (O$\underline{C}$H$_2$), 60.51 ($\underline{C}$H$_2$CH$_3$), 53.69 ($\underline{C}$H*), 53.16 ($\underline{C}$H*), 37.45 (Ph$\underline{C}_b$H$_2$), 36.68 (Ph$\underline{C}_a$H$_2$), 13.89 (CH$_2\underline{C}$H$_3$). HRMS (ESI) m/z: $[M+Na]^+$ calcd for $C_{32}H_{32}N_2NaO_5$ 547.2203; found 547.2197.

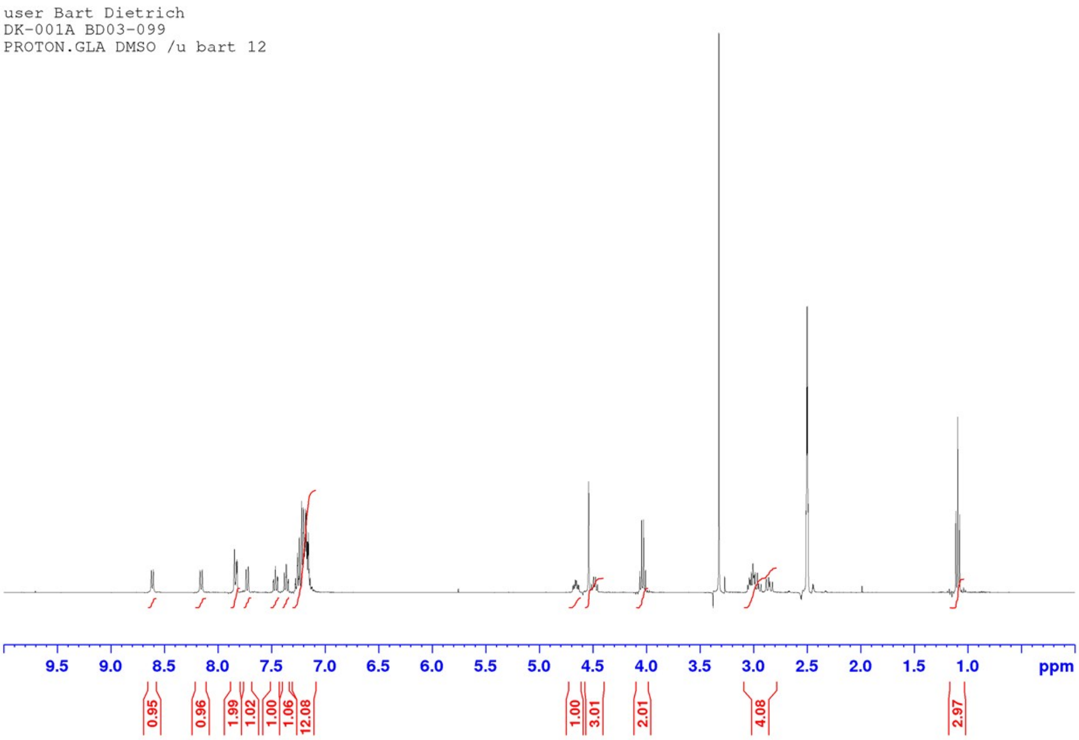

Figure S50. Proton NMR of **DK-001**.

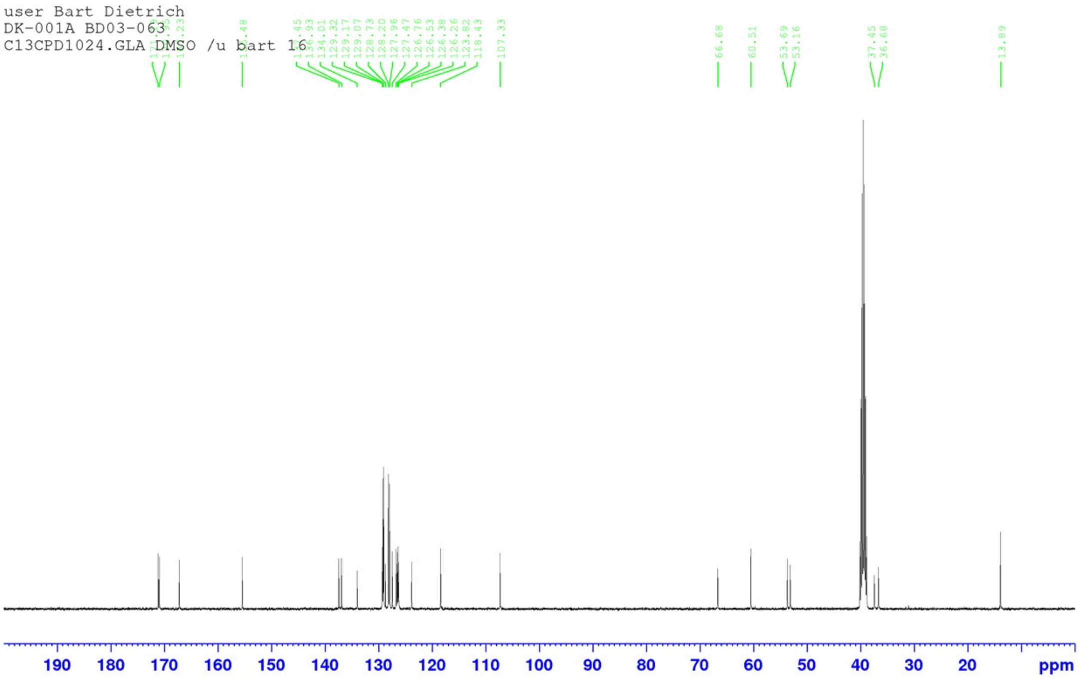

Figure S51. Carbon NMR of **DK-001**.

(2*S*)-2-[(2*S*)-2-[2-(Naphthalen-2-yloxy)acetamido]-3-phenylpropanamido]-3-phenylpropanoic acid (DK-002)

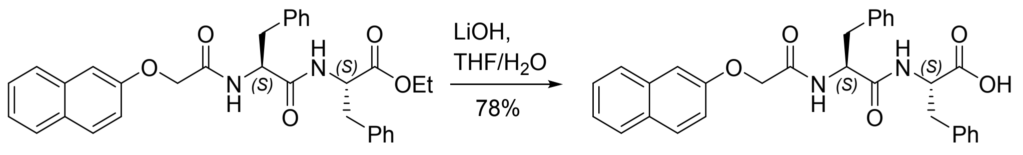

DK-001 **DK-002**

To a solution of **DK-001** (6.50 g, 12.4 mmol) in tetrahydrofuran (100 mL) was added a solution of lithium hydroxide (4 eq, 1.19 g) in water (100 mL) and the cloudy mixture was stirred overnight. After this time, the clear solution was poured into 1 M hydrochloric acid (*ca.* 600 mL) and stirred for 90 minutes. The resulting precipitate was filtered off, washed in the filter with water, then dried by azeotropic distillation with acetonitrile. The title compound was thus obtained as a white solid (4.79 g, 78 %) in sufficient purity (< 0.4 % acetonitrile by NMR).

δ_H (400 MHz, DMSO-d$_6$) 12.81 (1H, br s, COOH), 8.44 (1H, d, *J* 7.84, N$_a$H), 8.12 (1H, d, *J* 8.56, N$_b$H), 7.84-7.82 (2H, m, H$_{Ar}$), 7.72 (1H, d, *J* 8.12, H$_{Ar}$), 7.48-7.44 (1H, m, H$_{Ar}$), 7.38-7.34 (1H, m, H$_{Ar}$), 7.26-7.13 (12H, m, H$_{Ar}$), 4.64 (1H, dt, *J* 9.08, 4.16, C$_b$H*), 4.53 (2H, s, OCH$_2$), 4.50-4.44 (1H, m, C$_a$H*), 3.07 (1H, dd, *J* 14.02, 5.34, PhCH'H"C$_a$H*), 3.02 (1H, dd, *J* 14.16, 4.40, PhCH'H"C$_b$H*), 2.92 (1H, dd, *J* 13.92, 8.76, PhCH'H"C$_a$H*), 2.85 (1H, dd, *J* 13.82, 9.62, PhCH'H"C$_b$H*). δ_C (100 MHz, DMSO-d$_6$) 172.76, 170.88, and 167.24 (C=O), 155.51, 137.54, 137.38, 134.05, 129.38, 129.28, 129.15, 128.77, 128.21, 128.00, 127.53, 126.82, 126.48, 126.45, 126.28, 123.88, 118.48, and 107.34 (C$_{Ar}$), 66.70 (OCH$_2$), 53.53 (C$_a$H*), 53.26 (C$_b$H*), 37.45 (PhCH$_2$C$_a$H*), 36.71 (PhCH$_2$C$_b$H*). HRMS (ESI) m/z: [M+Na]$^+$ calcd for $C_{30}H_{28}N_2NaO_5$ 519.1890; found 519.1907.

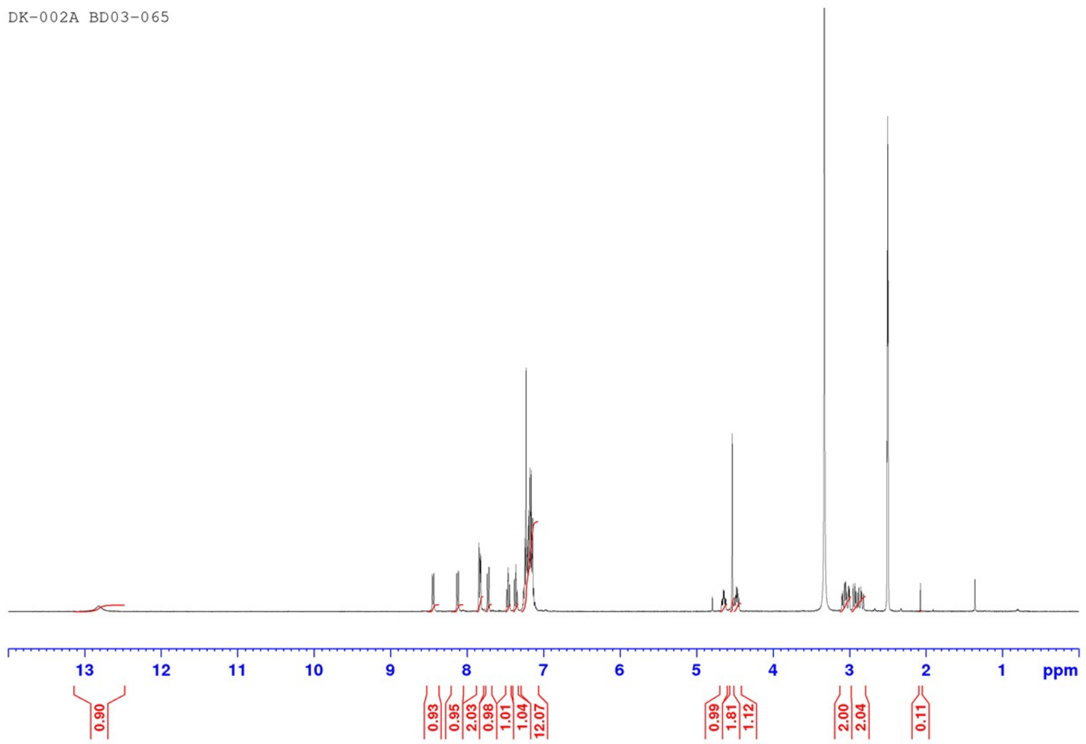

Figure S52. Proton NMR of **DK-002**.

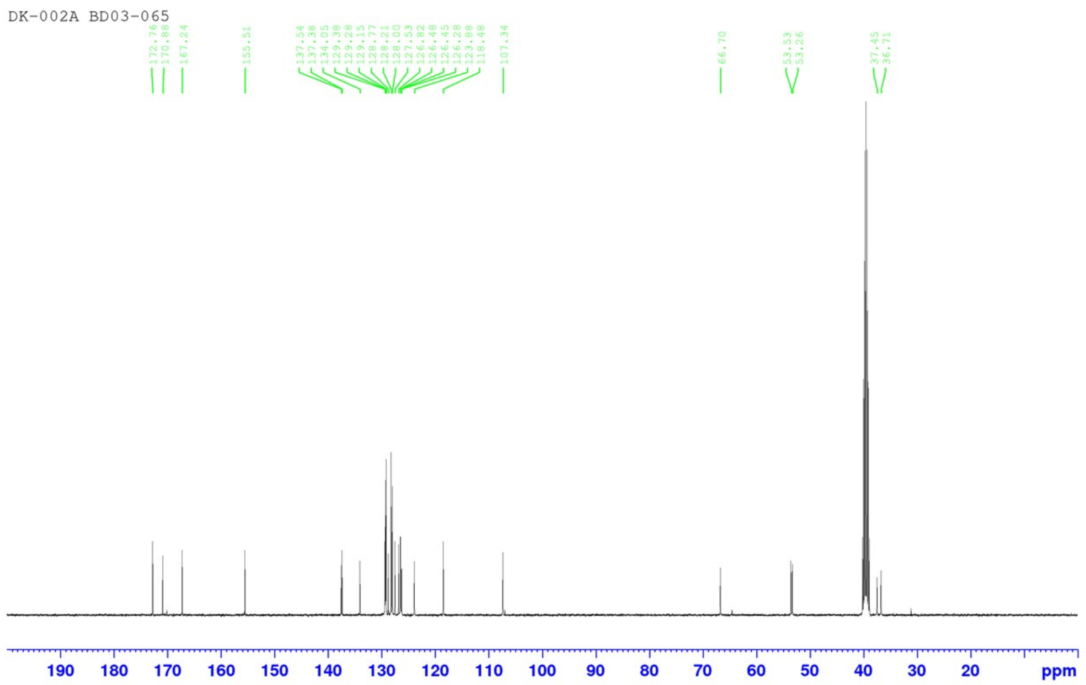

Figure S53. Carbon NMR of **DK-002**.

Methyl (2*R*)-2-[(2*R*)-2-[2-(naphthalen-2-yloxy)acetamido]-3-phenylpropanamido]-3-phenylpropanoate (DG-002)

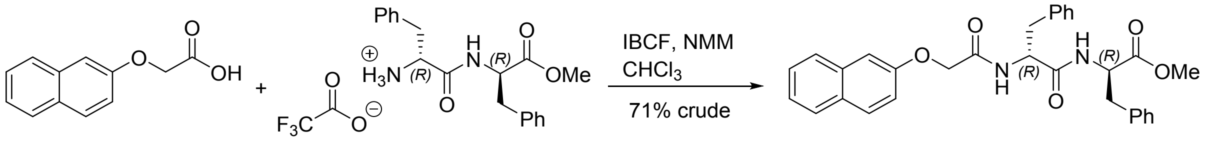

To a suspension of **DG-001** (1 eq, 615 mg) in chloroform (15 mL) was added *iso*butyl chloroformate (1 eq, 394 μL) followed by *N*-methylmorpholine (1 eq, 334 μL) and the mixture was stirred for 20 minutes. To the now clear solution was added **DF-004** (1.34 g, 3.04 mmol) and another portion of *N*-methylmorpholine (1 eq, 334 μL) and the reaction was left stirring overnight. After this time, it was diluted with chloroform, washed in turn with 1M hydrochloric acid, saturated sodium carbonate solution, water, and brine, dried ($MgSO_4$), and evaporated under reduced pressure. The title compound was thus obtained as a pale yellow-brown solid in 71% (1.10 g) crude yield and used as is in the next step. A small amount was purified *via* column chromatography (eluting with 1:9 ethyl acetate/dichloromethane) to obtain a sample for characterisation. Residual ethyl acetate (< 2%) is observed in the NMR data obtained.

δ_H (400 MHz, DMSO-d$_6$) 8.62 (1H, d, *J* 7.56, N$\underline{H}$), 8.16 (1H, d, *J* 8.60, N$\underline{H}$), 7.85-7.82 (2H, m, $\underline{H}_{Ar}$), 7.72 (1H, d, *J* 8.12, $\underline{H}_{Ar}$), 7.46 (1H, ddd, *J* 8.10, 6.94, 1.14, $\underline{H}_{Ar}$), 7.36 (1H, ddd, *J* 8.05, 6.93, 1.13, $\underline{H}_{Ar}$), 7.27-7.12 (12H, m, $\underline{H}_{Ar}$), 4.65 (1H, td, *J* 9.04, 4.40, C$\underline{H}^*$), 4.54 (2H, s, OC$\underline{H}_2$), 4.54-4.48 (1H, m, C$\underline{H}^*$), 3.59 (3H, s, OC$\underline{H}_3$), 3.06-2.92 (3H, m, Ph$_a$C$\underline{H}_2$ and Ph$_b$C$\underline{H}_a$H$_b$), 2.84 (1H, dd, *J* 13.78, 9.50, Ph$_b$CH$_a\underline{H}_b$). δ_C (100 MHz, DMSO-d$_6$) 171.68, 170.92, and 167.21 ($\underline{C}$=O), 155.48, 137.42, 136.93, 134.01, 129.33, 129.18, 129.03, 128.73, 128.23, 127.97, 127.48, 126.76, 126.55, 126.39, 126.25, 123.83, 118.43, and 107.32 ($\underline{C}_{Ar}$), 66.67 (O$\underline{C}$H$_2$), 53.62 ($\underline{C}$H*), 53.16 ($\underline{C}$H*), 51.83 (O$\underline{C}$H$_3$), 37.42 (Ph$_b\underline{C}$H$_2$), 36.61 (Ph$_a\underline{C}$H$_2$). HRMS (ESI) m/z: $[M+Na]^+$ calcd for $C_{31}H_{30}N_2NaO_5$ 533.2047; found 533.2040.

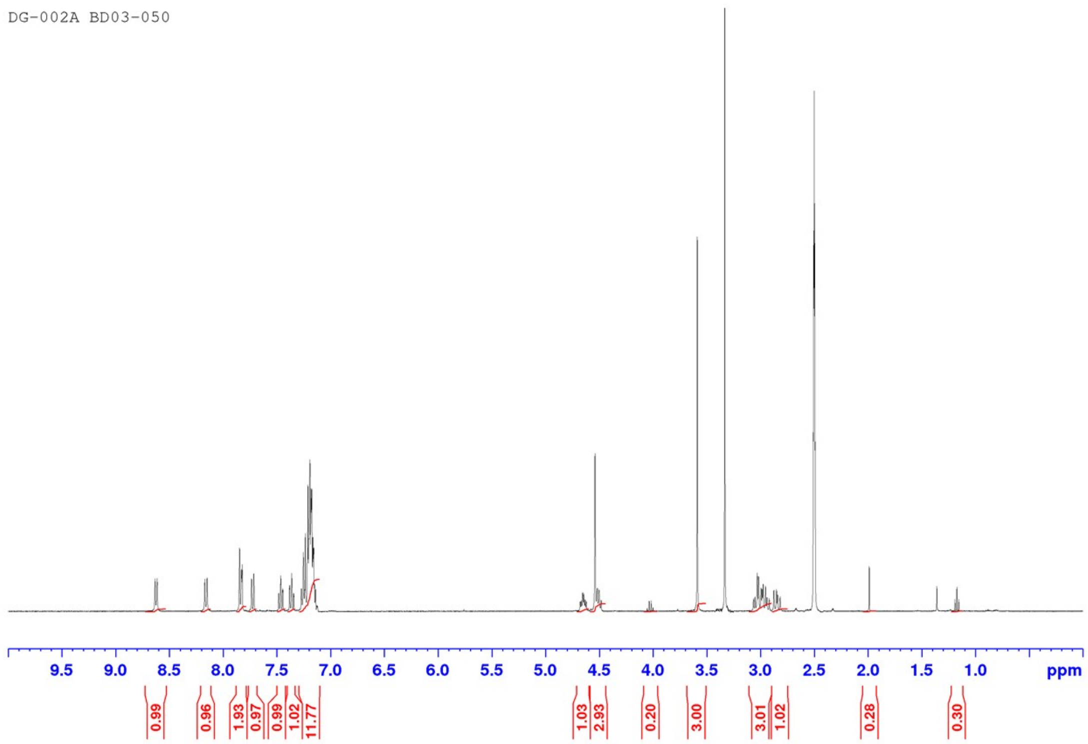

Figure S54. Proton NMR of **DG-002**.

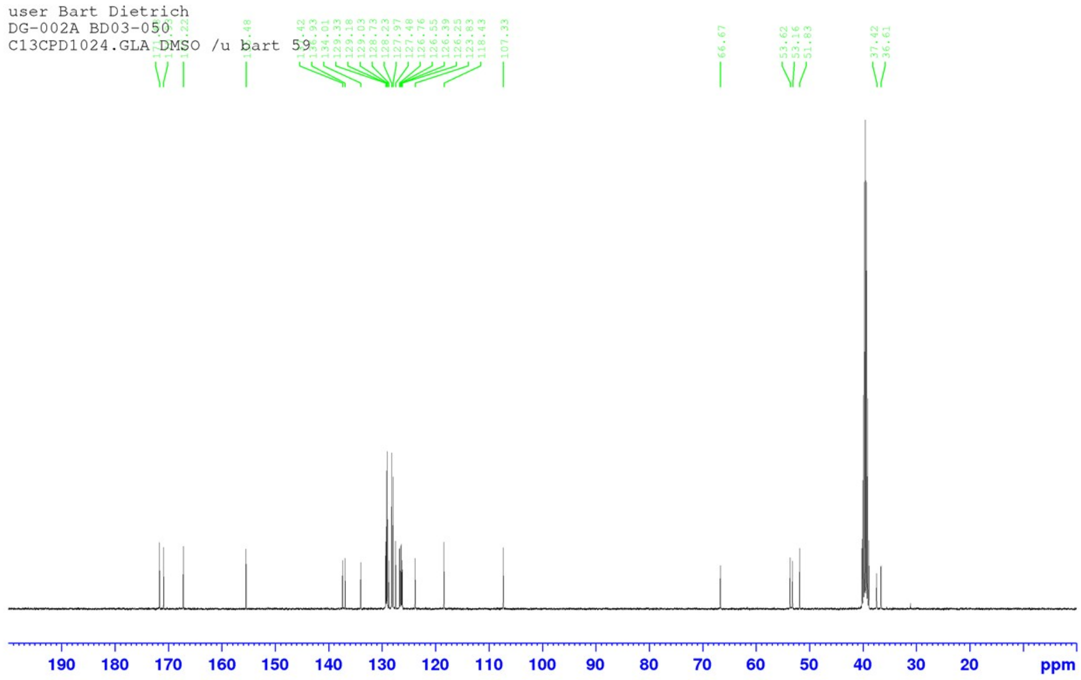

Figure S55. Carbon NMR of **DG-002**.

(2*R*)-2-[(2*R*)-2-[2-(Naphthalen-2-yloxy)acetamido]-3-phenylpropanamido]-3-phenylpropanoic acid (DG-003)

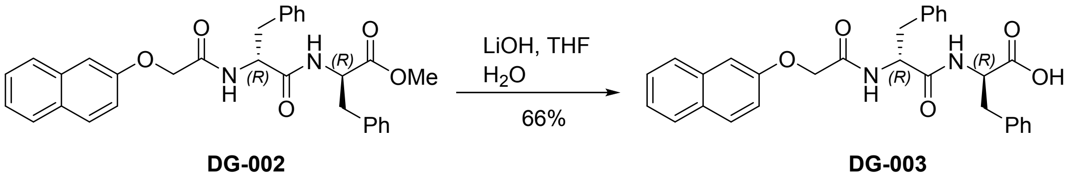

To a solution of **DG-002** (4.10 g, 8.03 mmol) in tetrahydrofuran (30 mL) was added a solution of lithium hydroxide (4 eq, 769 mg) in water (30 mL) and the mixture was stirred overnight. After this time, the reaction presented as a turbid mixture which cleared up after the addition of some more water. This was poured into 1M hydrochloric acid (300 mL) and stirred for 30 minutes. The solids were filtered off and washed in the filter with several portions of water, then dried by azeotropic distillation with acetonitrile on a rotary evaporator. The title compound was thus obtained as an off-white solid (2.62 g, 66%). Trace (< 1 %) dichloromethane is seen in the NMR spectrum.

δ_H (400 MHz, DMSO-d_6) 12.81 (1H, br s, COO$\underline{H}$), 8.44 (1H, d, *J* 7.92, N$\underline{H}$), 8.12 (1H, d, *J* 8.60, N$\underline{H}$), 7.84-7.82 (2H, m, $\underline{H}_{Ar}$), 7.72 (1H, d, *J* 8.16, $\underline{H}_{Ar}$), 7.48-7.44 (1H, m, $\underline{H}_{Ar}$), 7.38-7.34 (1H, m, $\underline{H}_{Ar}$), 7.26-7.13 (12H, m, $\underline{H}_{Ar}$), 4.64 (1H, td, *J* 9.01, 4.03, C$\underline{H}^*$), 4.53 (2H, s, OC$\underline{H}_2$), 4.50-4.45 (1H, m, C$\underline{H}^*$), 3.07 (1H, dd, *J* 14.06, 5.38, PhC$_a\underline{H}_a$H$_b$), 3.02 (1H, dd, *J* 14.12, 4.32, PhC$_b\underline{H}_a$H$_b$), 2.92 (1H, dd, *J* 13.92, 8.76, PhC$_a$H$_a\underline{H}_b$), 2.85 (1H, dd, *J* 13.72, 9.60, PhC$_b$H$_a\underline{H}_b$). δ_C (100 MHz, DMSO-d_6) 172.71, 170.83, and 167.22 ($\underline{C}$=O), 155.50, 137.51, 137.36, 134.03, 129.36, 129.25, 129.13, 128.76, 128.18, 127.97, 127.50, 126.79, 126.45, 126.43, 126.25, 123.86, 118.45, and 107.35 ($\underline{C}_{Ar}$), 66.70 (O$\underline{C}$H$_2$), 53.50 ($\underline{C}$H*), 53.24 ($\underline{C}$H*), 37.43 (Ph$\underline{C}_b$H$_2$), 36.70 (Ph$\underline{C}_a$H$_2$). HRMS (ESI) m/z: $[M+Na]^+$ calcd for $C_{30}H_{28}N_2NaO_5$ 519.1890; found 519.1884.

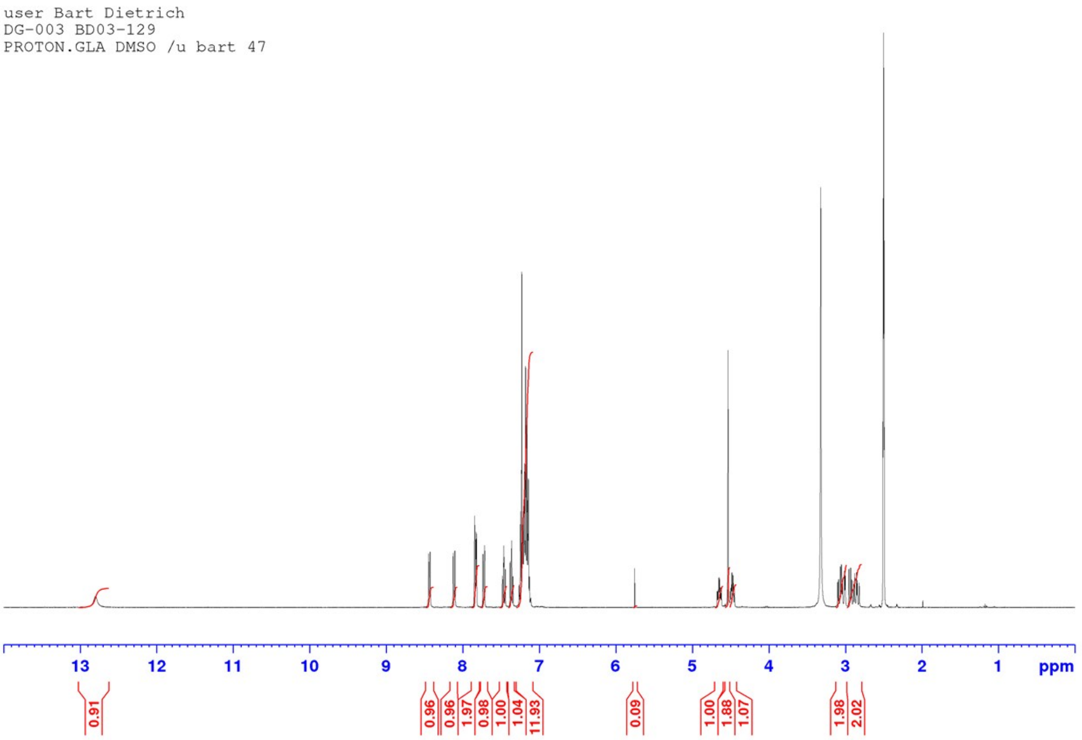

Figure S56. Proton NMR of **DG-003**.

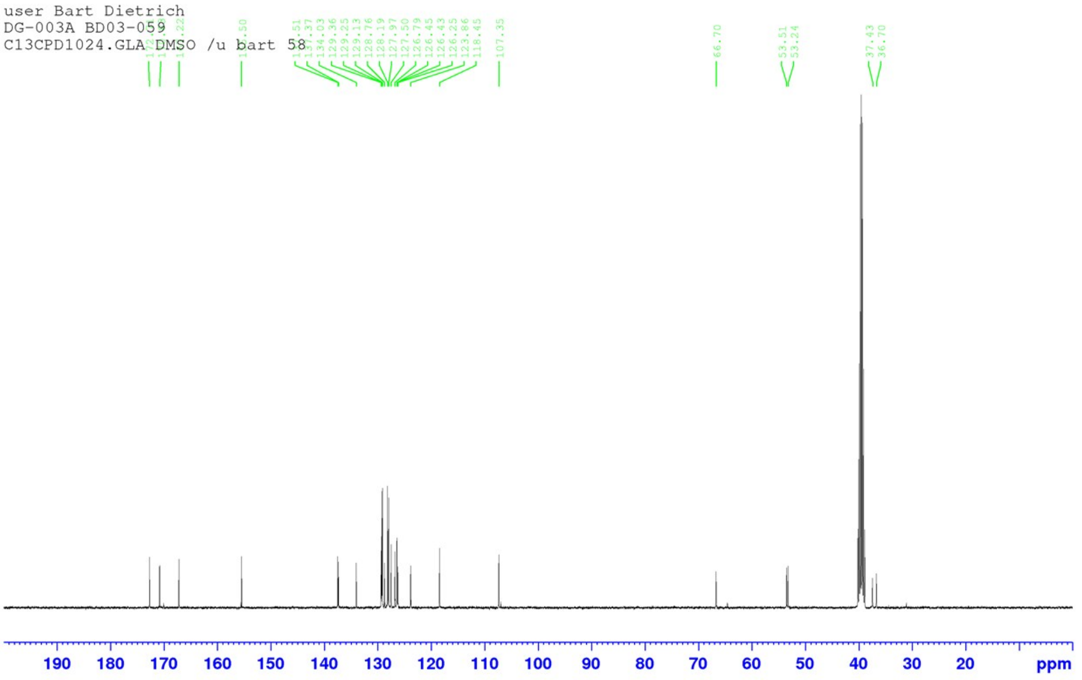

Figure S57. Carbon NMR of **DG-003**.

Methyl (2*R*)-2-[(2*S*)-2-[2-(naphthalen-2-yloxy)acetamido]-3-phenylpropanamido]-3-phenylpropanoate (EF-003)

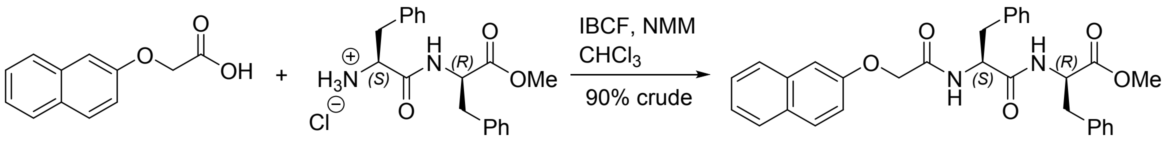

EF-001 **EF-003**

To a suspension of 2-(naphthalen-2-yloxy)acetic acid (658 mg, 3.25 mmol) in chloroform (20 mL) were added *iso*butyl chloroformate (1.02 eq, 430 µL) and *N*-methylmorpholine (1.1 eq, 393 µL) and the mixture was stirred for 10 minutes. After this time, **EF-001** (1 eq, 1.18 g) and another portion of *N*-methylmorpholine (1.1 eq, 393 µL) were added and the mixture was stirred overnight. It was then diluted with chloroform, washed in turn with 1M hydrochloric acid and brine, dried ($MgSO_4$), and evaporated to dryness *in vacuo*. Crude **EF-003** was thus obtained as a grey solid (1.50 g, 90%) and used as is in the next step. A small amount was purified *via* column chromatography (1:9 ethyl acetate/dichloromethane) to afford a sample for characterisation. This was evaporated from acetonitrile to obtain a white solid. Traces of acetonitrile are observed in the NMR.

δ_H (400 MHz, DMSO-d_6) 8.71 (1H, d, *J* 8.12, N<u>H</u>), 8.09 (1H, d, *J* 8.64, N<u>H</u>), 7.84-7.82 (2H, m, $\underline{H}_{Ar}$), 7.73 (1H, d, *J* 8.20, $\underline{H}_{Ar}$), 7.46 (1H, ddd, *J* 8.11, 6.97, 1.15, $\underline{H}_{Ar}$), 7.36 (1H, ddd, *J* 8.06, 6.90, 1.16, $\underline{H}_{Ar}$), 7.29-7.24 (4H, m, $\underline{H}_{Ar}$), 7.22-7.16 (3H, m, $\underline{H}_{Ar}$), 7.12-7.11 (3H, m, $\underline{H}_{Ar}$), 7.01-6.99 (2H, m, $\underline{H}_{Ar}$), 4.64 (1H, td, *J* 8.90, 4.32, C$\underline{H}^*$), 4.55 (2H, s, OC$\underline{H}_2$), 4.55-4.49 (1H, m, C$\underline{H}^*$), 3.62 (3H, s, OC$\underline{H}_3$), 3.08 (1H, dd, *J* 13.61, 5.00, PhC$\underline{H}_2$), 2.86 (1H, dd, *J* 13.69, 10.00, PhC$\underline{H}_2$), 2.75 (1H, dd, *J* 13.55, 4.26, PhC$\underline{H}_2$), 2.64 (1H, dd, *J* 13.63, 9.18, PhC$\underline{H}_2$). δ_C (100 MHz, DMSO-d_6) 171.86, 170.76, and 167.19 (<u>C</u>=O), 155.48, 137.28, 137.08, 134.02, 129.33, 129.22, 129.14, 128.74, 128.23, 127.92, 127.48, 126.75, 126.62, 126.40, 126.22, 123.83, 118.42, and 107.32 ($\underline{C}_{Ar}$), 66.66 (O$\underline{C}H_2$), 53.52 ($\underline{C}H^*$), 53.23 ($\underline{C}H^*$), 51.94 (O$\underline{C}H_3$), 37.69 (Ph$\underline{C}H_2$), 36.91 (Ph$\underline{C}H_2$). HRMS (ESI) m/z: $[M+Na]^+$ calcd for $C_{31}H_{30}N_2NaO_5$ 533.2047; found 553.2033.

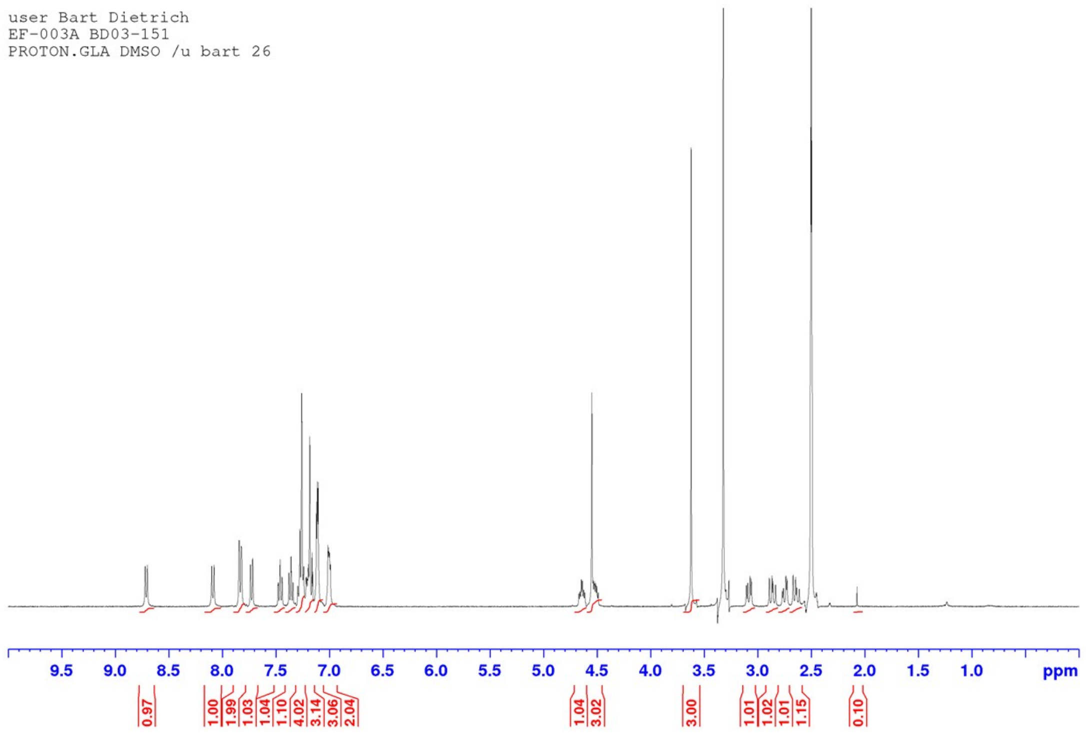

Figure S58. Proton NMR of **EF-003**.

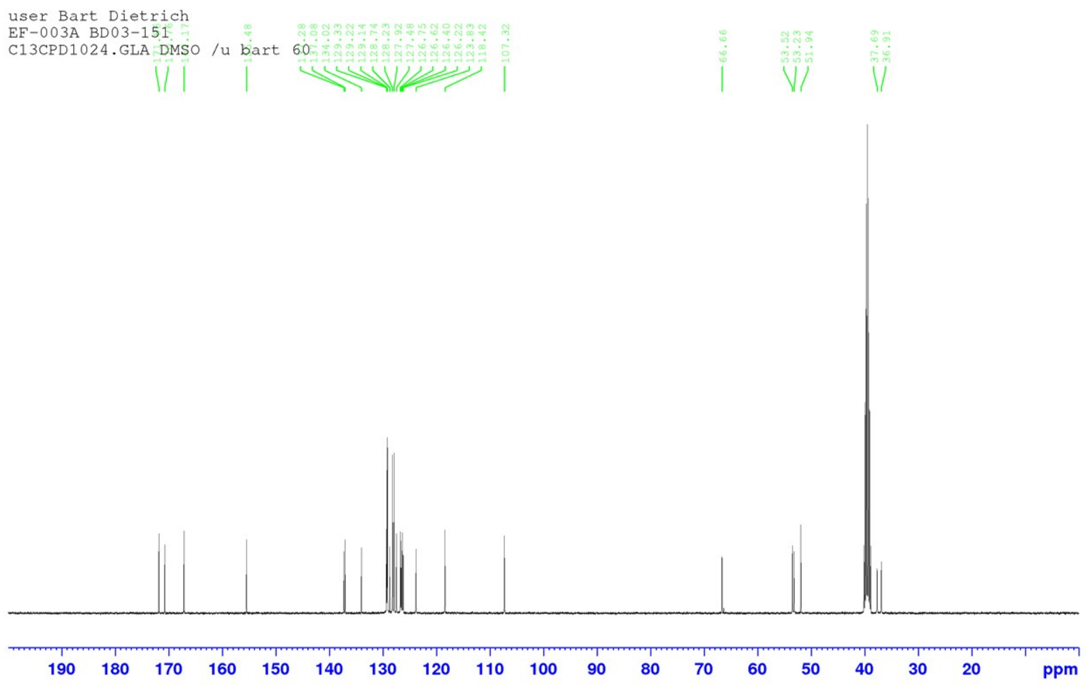

Figure S59. Carbon NMR of **EF-003**.

(2*R*)-2-[(2*S*)-2-[2-(Naphthalen-2-yloxy)acetamido]-3-phenylpropanamido]-3-phenylpropanoic acid (EF-006)

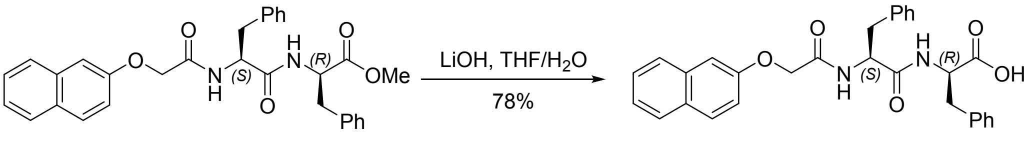

EF-003 **EF-006**

To a solution of **EF-003** (1.41 g, 2.76 mmol) in tetrahydrofuran (15 mL) was added a solution of lithium hydroxide (4 eq, 264 mg) in water (15 mL) and the mixture was stirred overnight. After this time, TLC indicated the absence of starting material. The reaction mixture was poured into 1M hydrochloric acid (300 mL) and stirred for 1 hour. The solids were filtered off, washed with water, then a small portion of acetonitrile (this removes a residual brown colour), and dried under vacuum. The title compound was this obtained as a white solid (1.07 g, 78%). The NMR data indicates the presence of *ca.* 0.2% acetonitrile.

δ_H (400 MHz, DMSO-d$_6$) 12.85 (1H, br s, COOH), 8.58 (1H, d, *J* 8.36, NH), 8.05 (1H, d, *J* 8.60, NH), 7.84-7.82 (2H, m, H_{Ar}), 7.73 (1H, d, *J* 8.12, H_{Ar}), 7.46 (1H, ddd, *J* 8.12, 6.95, 1.17, H_{Ar}), 7.36 (1H, ddd, *J* 8.07, 6.91, 1.17, H_{Ar}), 7.27-7.26 (4H, m, H_{Ar}), 7.21-7.15 (3H, m, H_{Ar}), 7.10-7.08 (3H, m, H_{Ar}), 6.98-6.96 (2H, m, H_{Ar}), 4.65 (1H, td, *J* 8.84, 4.24, CH*), 4.54 (2H, s, OCH$_2$), 4.50-4.44 (1H, m, CH*), 3.11 (1H, dd, *J* 13.69, 4.56, Ph$_a$CH$_a$H$_b$), 2.84 (1H, dd, *J* 13.67, 10.10, Ph$_a$CH$_a$H$_b$), 2.75 (1H, dd, *J* 13.71, 4.10, Ph$_b$CH$_a$H$_b$), 2.62 (1H, dd, *J* 13.63, 9.18, Ph$_b$CH$_a$H$_b$). δ_C (100 MHz, DMSO-d$_6$) 172.85, 170.59, and 167.12 (C=O), 155.48, 137.49, 137.31, 134.03, 129.36, 129.27, 129.17, 128.75, 128.18, 127.90, 127.49, 126.78, 126.52, 126.42, 126.18, 123.85, 118.42, and 107.34 (C_{Ar}), 66.69 (OCH$_2$), 53.49 (CH*), 53.24 (CH*), 37.76 (Ph$_b$CH$_2$), 37.02 (Ph$_a$CH$_2$). HRMS (ESI) m/z: [M+Na]$^+$ calcd for $C_{30}H_{28}N_2NaO_5$ 519.1890; found 519.1878.

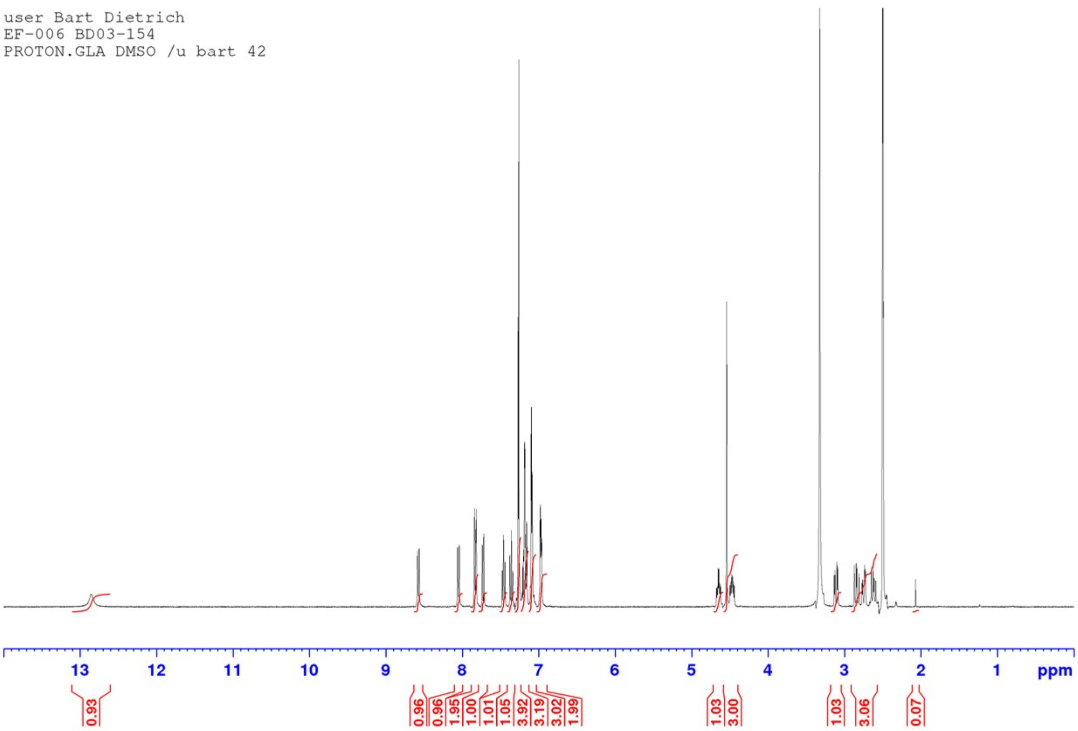

Figure S60. Proton NMR of **EF-006**.

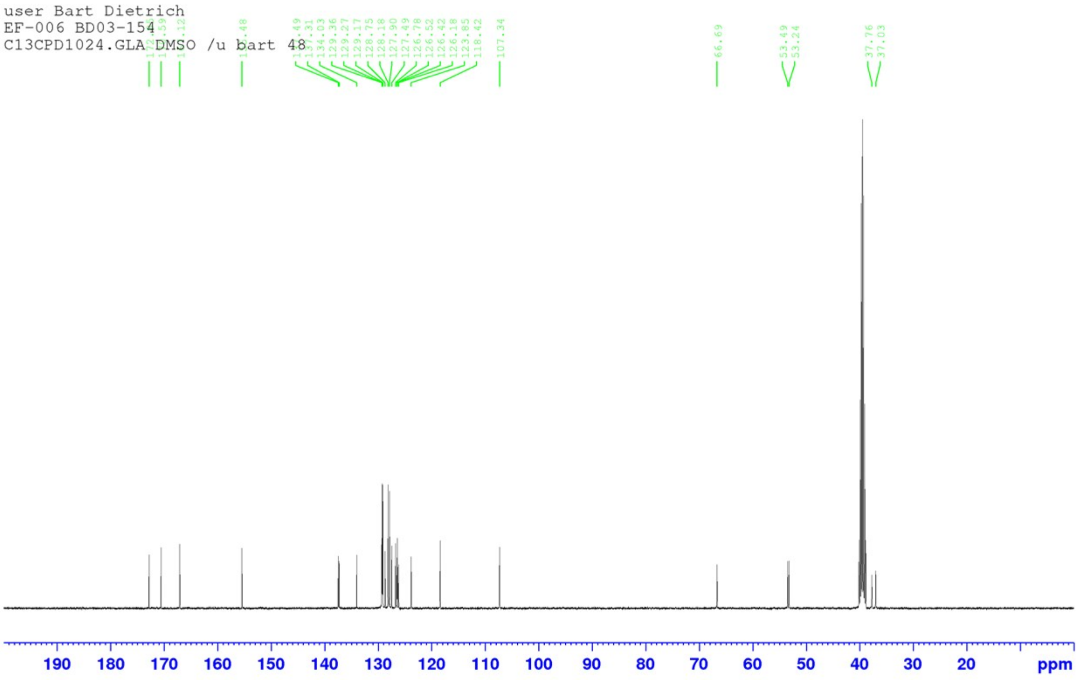

Figure S61. Carbon NMR of **EF-006**.

Ethyl (2*S*)-2-[(2*R*)-2-[2-(naphthalen-2-yloxy)acetamido]-3-phenylpropanamido]-3-phenylpropanoate (EE-003)

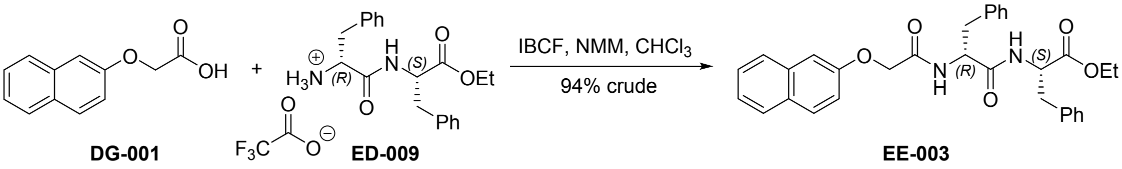

To a suspension of **DG-001** (534 mg, 2.64 mmol) in chloroform (20 mL) was added *N*-methylmorpholine (1.1 eq, 319 µL) and *iso*butyl chloroformate (1.02 eq, 349 µL). After stirring for 20 minutes, **ED-009** (1 eq, 1.20 g) and another portion of *N*-methylmorpholine (1.1 eq, 319 µL) were added and the reaction mixture stirred for 3 days. It was then diluted with chloroform, washed in turn with 1 M hydrochloric acid, water, and brine, dried ($MgSO_4$), filtered and evaporated to dryness under reduced pressure. The title compound **EE-003** was thus obtained as a camel solid (1.30 g, 94 % crude) and used in the next step without futher purification. A small amount was purified for characterisation *via* column chromatography (1:9 ethyl acetate/dichloromethane).

δ_H (400 MHz, DMSO-d_6) 8.69 (1H, d, *J* 8.08, $N\underline{H}C_aH^*$), 8.11 (1H, d, *J* 8.64, $N\underline{H}C_bH^*$), 7.84-7.82 (2H, m, $\underline{H}_{Ar}$), 7.73 (1H, d, *J* 8.12, $\underline{H}_{Ar}$), 7.46 (1H, ddd, *J* 8.14, 6.92, 1.20, $\underline{H}_{Ar}$), 7.36 (1H, ddd, *J* 8.07, 6.91, 1.17, $\underline{H}_{Ar}$), 7.29-7.10 (10H, m, $\underline{H}_{Ar}$), 7.04-7.01 (2H, m, $\underline{H}_{Ar}$), 4.65 (1H, td, *J* 8.87, 4.38, $NHC_b\underline{H}^*$), 4.55 (2H, s, $OC\underline{H}_2$), 4.51-4.45 (1H, m, $NHC_a\underline{H}^*$), 4.06 (2H, q, *J* 7.10, $C\underline{H}_2CH_3$), 3.06 (1H, dd, *J* 13.65, 5.32, $NHC_aH^*C\underline{H}_mH_nPh$), 2.87 (1H, dd, *J* 13.63, 9.78, $NHC_aH^*CH_m\underline{H}_nPh$), 2.77 (1H, dd, *J* 13.69, 4.28, $NHC_bH^*C\underline{H}_mH_nPh$), 2.66 (1H, dd, *J* 13.69, 9.32, $NHC_bH^*CH_m\underline{H}_nPh$), 1.12 (3H, t, *J* 7.10, $CH_2C\underline{H}_3$). δ_C (100 MHz, DMSO-d_6) 171.35, 170.77, and 167.16 ($\underline{C}$=O), 155.48, 137.31, 137.07, 134.02, 129.33, 129.23, 129.14, 128.73, 128.21, 127.92, 127.47, 126.75, 126.60, 126.39, 126.22, 123.82, 118.42, and 107.31 ($\underline{C}_{Ar}$), 66.66 ($O\underline{C}H_2$), 60.63 ($\underline{C}H_2CH_3$), 53.62 ($NH\underline{C}_aH^*$), 53.25 ($NH\underline{C}_bH^*$), 37.70 ($NHC_bH^*\underline{C}H_2Ph$), 36.94 ($NHC_aH^*\underline{C}H_2Ph$), 13.92 ($CH_2\underline{C}H_3$). HRMS (ESI) m/z: $[M+Na]^+$ calcd for $C_{32}H_{32}N_2NaO_5$ 547.2203; found 547.2184.

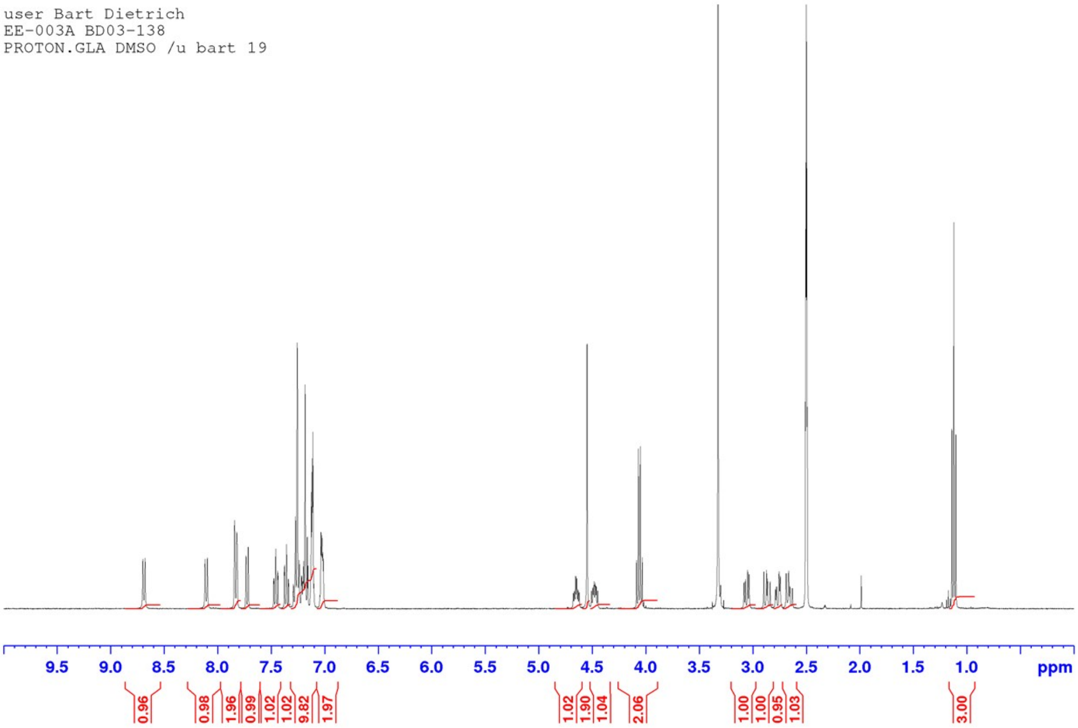

Figure S62. Proton NMR of **EE-003**.

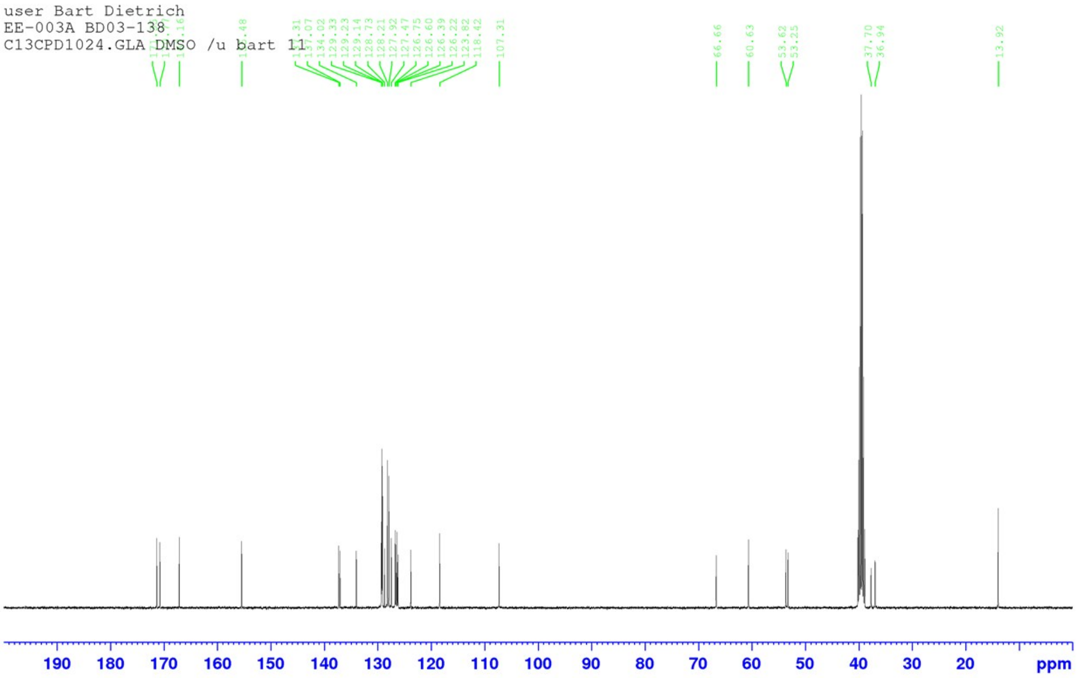

Figure S63. Carbon NMR of **EE-003**.

(2*S*)-2-[(2*R*)-2-[2-(Naphthalen-2-yloxy)acetamido]-3-phenylpropanamido]-3-phenylpropanoic acid (EE-006)

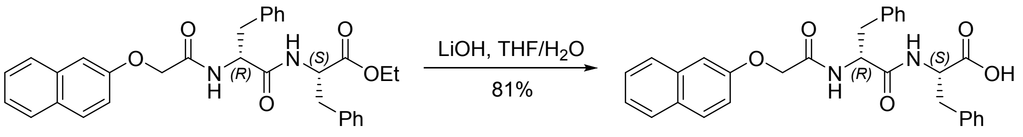

EE-003 **EE-006**

To a solution of **EE-003** (1.19 g, 2.27 mmol) in tetrahydrofuran (20 mL) was added a solution of lithium hydroxide (4 eq, 217 mg) in water (20 mL) and the mixture was stirred overnight. After this time, TLC indicated the absence of starting material. The reaction mixture was poured into 1 M hydrochloric acid and stirred for 1 hour. The solids were filtered off, then washed with 1 M hydrochloric acid and water. A residual brown colour carried over from the starting material was removed by slurrying in acetonitrile, sonication and filtration. The title compound was obtained as a white solid (909 mg, 81%) containing < 0.3% acetonitrile by NMR.

δ_H (400 MHz, DMSO-d$_6$) 12.86 (1H, br s, COO$\underline{H}$), 8.58 (1H, d, *J* 8.32, N$\underline{H}$C$_m$H*), 8.05 (1H, d, *J* 8.60, N$\underline{H}$C$_n$H*), 7.84-7.82 (2H, m, $\underline{H}_{Ar}$), 7.73 (1H, d, *J* 8.08, $\underline{H}_{Ar}$), 7.46 (1H, ddd, *J* 8.13, 6.92, 1.20, $\underline{H}_{Ar}$), 7.36 (1H, ddd, *J* 8.08, 6.90, 1.18, $\underline{H}_{Ar}$), 7.27-7.26 (4H, m, $\underline{H}_{Ar}$), 7.22-7.15 (3H, m, $\underline{H}_{Ar}$), 7.10-7.08 (3H, m, $\underline{H}_{Ar}$), 6.98-6.96 (2H, m, $\underline{H}_{Ar}$), 4.65 (1H, td, *J* 8.86, 4.20, C$_n\underline{H}^*$), 4.54 (2H, s, OC$\underline{H}_2$), 4.50-4.44 (1H, m, C$_m\underline{H}^*$), 3.11 (1H, dd, *J* 13.69, 4.60, PhC$\underline{H}_a$H$_b$C$_m$H*), 2.84 (1H, dd, *J* 13.63, 10.06, PhCH$_a\underline{H}_b$C$_m$H*), 2.75 (1H, dd, *J* 13.67, 4.10, PhC$\underline{H}_a$H$_b$C$_n$H*), 2.62 (1H, dd, *J* 13.65, 9.20, PhCH$_a\underline{H}_b$C$_n$H*). δ_C (100 MHz, DMSO-d$_6$) 172.84, 170.58, and 167.11 ($\underline{C}$=O), 155.48, 137.48, 137.30, 134.02, 129.35, 129.26, 129.16, 128.74, 128.17, 127.89, 127.48, 126.77, 126.51, 126.42, 126.18, 123.84, 118.41, and 107.33 ($\underline{C}_{Ar}$), 66.68 (O$\underline{C}$H$_2$), 53.49 ($\underline{C}_m$H*), 53.23 ($\underline{C}_n$H*), 37.75 (Ph$\underline{C}$H$_2$C$_n$H*), 37.02 (Ph$\underline{C}$H$_2$C$_m$H*). HRMS (ESI) m/z: [M+Na]$^+$ calcd for $C_{30}H_{28}N_2NaO_5$ 519.1890; found 519.1867.

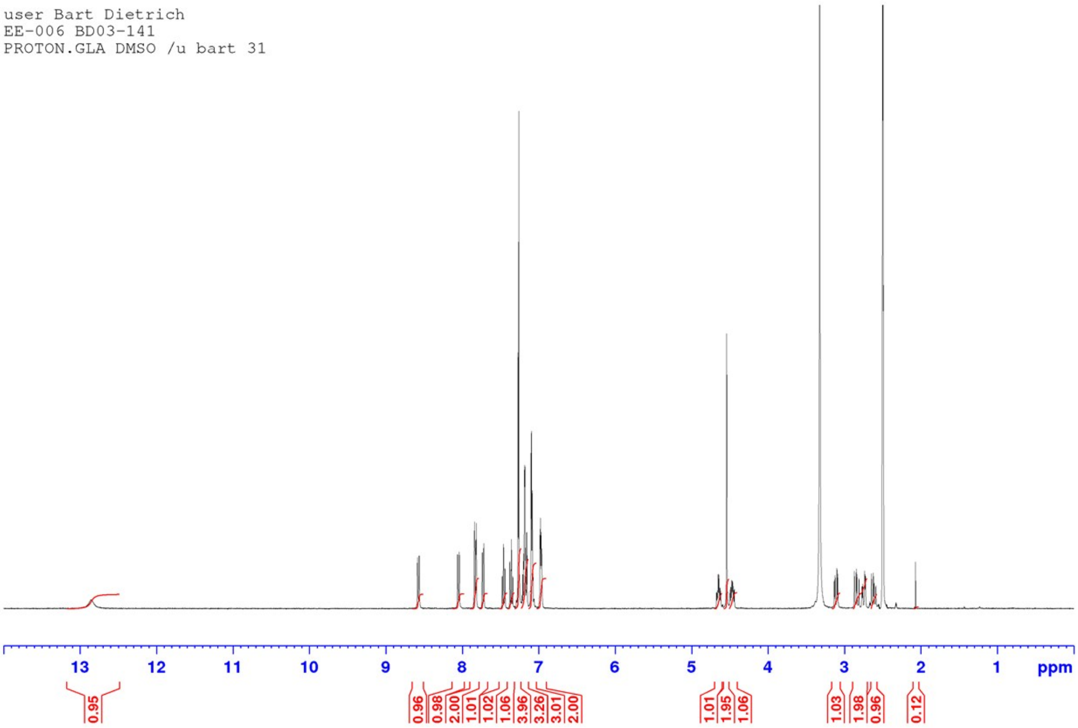

Figure S64. Proton NMR of **EE-006**.

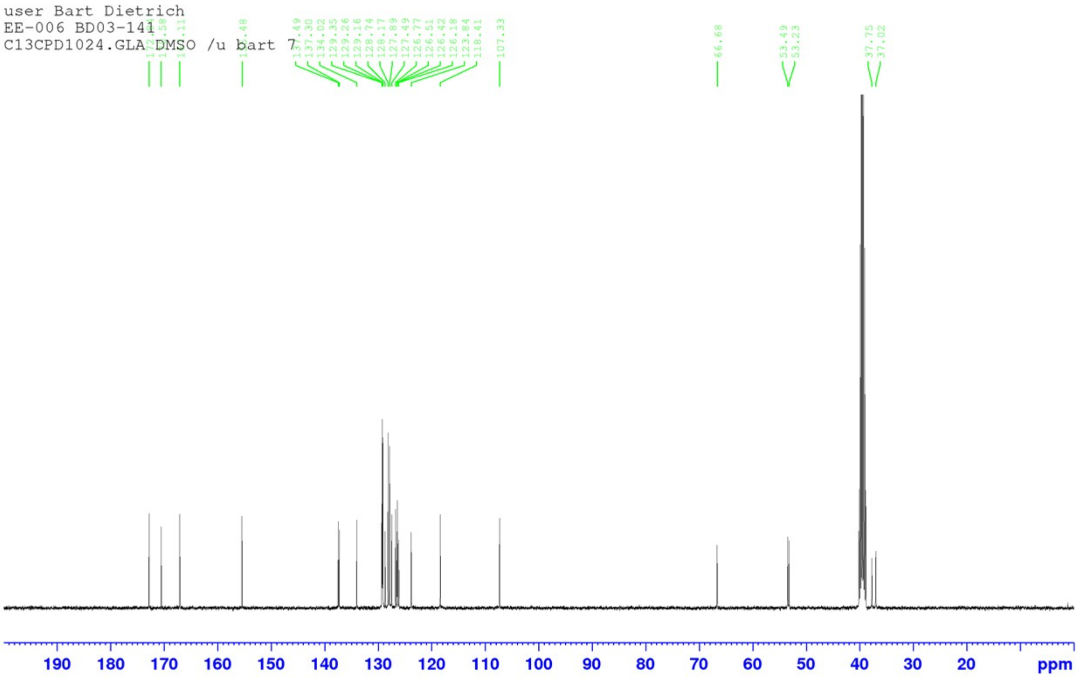

Figure S65. Carbon NMR of **EE-006**.

Ethyl (2*S*)-3-phenyl-2-[(2*R*)-3-phenyl-2-[2-(5,6,7,8-tetrahydronaphthalen-1-yloxy)acetamido]propanamido]propanoate (EG-006)

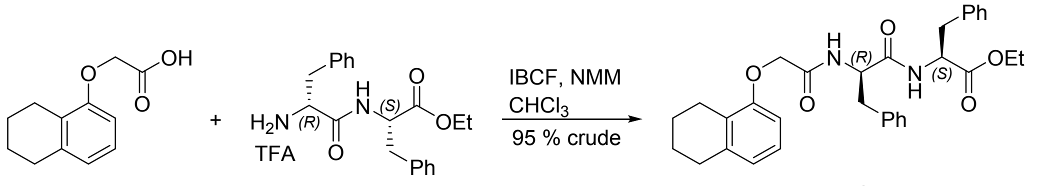

ED-009 **EG-006**

To a solution of 2-(5,6,7,8-tetrahydronaphthalen-1-yloxy)acetic acid (733 mg, 3.55 mmol) in chloroform (25 mL) were added *iso*butyl chloroformate (1.02 eq, 470 µL) and *N*-methylmorpholine (1.1 eq, 429 µL) and the mixture was stirred for 10 minutes. After this time, **ED-009** (1 eq, 1.62 g) and another portion of *N*-methylmorpholine (1.1 eq, 429 µL) were added and the reaction was stirred overnight. After this time it was diluted with chloroform, washed in turn with 1M hydrochloric acid and brine, dried ($MgSO_4$), and evaporated to dryness under reduced pressure. Crude **EG-006** was thus obtained as a white solid (1.79 g, 95%) and used as is in the next step. A small amount was purified *via* column chromatography (1:9 ethyl acetate/dichloromethane) to afford a sample for characterisation. One aliphatic carbon is not resolved in the carbon NMR.

δ_H (400 MHz, DMSO-d_6) 8.70 (1H, d, *J* 8.12, N<u>H</u>), 7.71 (1H, d, *J* 8.56, N<u>H</u>), 7.32-7.19 (5H, m, $\underline{H}_{Ar}$), 7.17-7.14 (3H, m, $\underline{H}_{Ar}$), 6.97-6.91 (3H, m, $\underline{H}_{Ar}$), 6.66 (1H, d, *J* 7.60, $\underline{H}_{Ar}$), 6.49 (1H, d, *J* 8.00, $\underline{H}_{Ar}$), 4.67 (1H, td, *J* 8.40, 4.48, C$\underline{H}^*$), 4.50-4.44 (1H, m, C$\underline{H}^*$), 4.42 (1H, d, *J* 14.81, OC$\underline{H}_a$$H_b$), 4.35 (1H, d, *J* 14.73, OC$H_a$$\underline{H}_b$), 4.09 (2H, q, *J* 7.10, C$\underline{H}_2$$CH_3$), 3.08 (1H, dd, *J* 13.61, 5.16, PhC$\underline{H}_2$), 2.87 (1H, dd, *J* 13.61, 9.92, PhC$\underline{H}_2$), 2.79 (1H, dd, *J* 13.63, 4.42, PhC$\underline{H}_2$), 2.68-2.63 (3H, m, PhC$\underline{H}_2$ and (C$\underline{H}_2$)$_4$), 2.55-2.41 (2H, m, (C$\underline{H}_2$)$_4$, overlapped by DMSO peak), 1.71-1.64 (2H, m, (C$\underline{H}_2$)$_4$), 1.14 (3H, t, *J* 7.10, CH_2C$\underline{H}_3$). δ_C (100 MHz, DMSO-d_6) 171.38, 170.56, and 167.25 ($\underline{C}$=O), 155.15, 137.91, 137.09, 136.86, 129.24, 129.22, 128.25, 127.91, 126.64, 126.25, 125.69, 125.07, 121.82, and 108.41 ($\underline{C}_{Ar}$), 66.74 (O$\underline{C}H_2$), 60.66 ($\underline{C}H_2CH_3$), 53.70 ($\underline{C}H^*$), 52.72 ($\underline{C}H^*$), 37.84 (Ph$\underline{C}H_2$), 36.91 (Ph$\underline{C}H_2$), 28.98, 22.57, and 22.31 (($\underline{C}H_2$)$_4$), 13.93 ($CH_2\underline{C}H_3$). HRMS (ESI) m/z: $[M+Na]^+$ calcd for $C_{32}H_{36}N_2NaO_5$ 551.2516; found 551.2505.

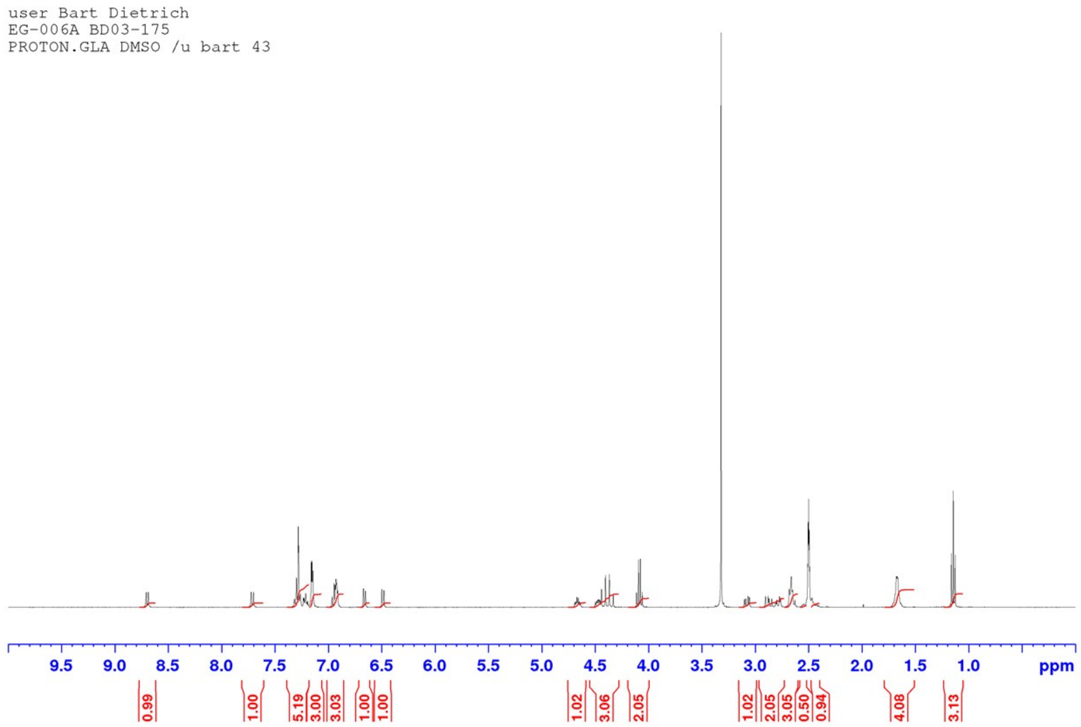

Figure S66. Proton NMR of **EG-006**.

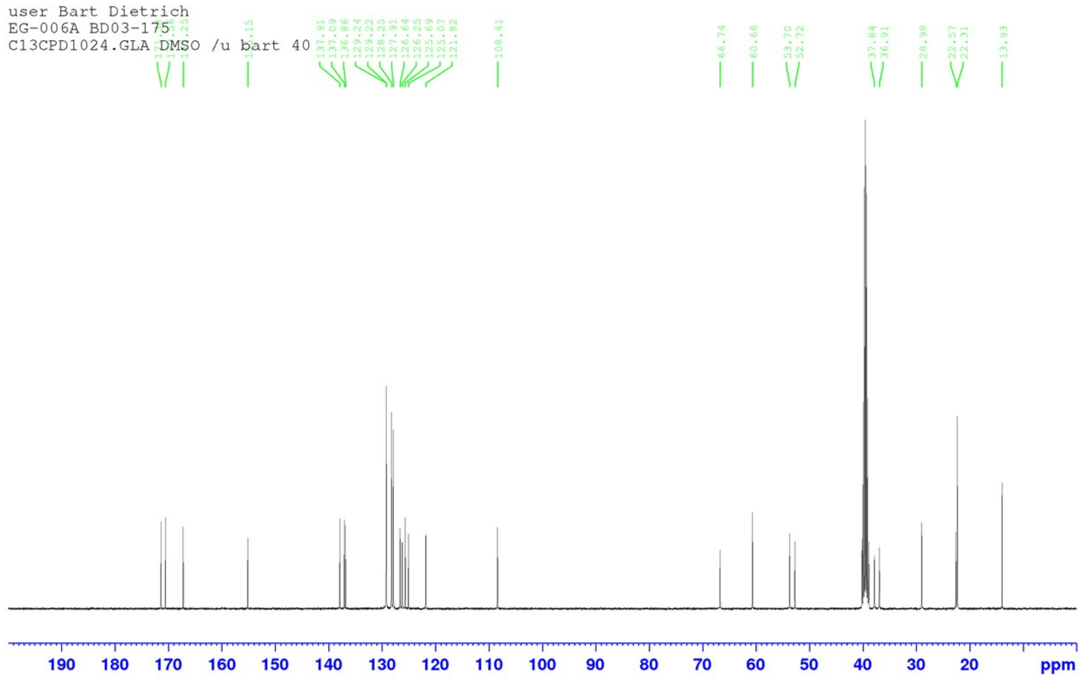

Figure S67. Carbon NMR of **EG-006**.

<u>(2*S*)-3-Phenyl-2-[(2*R*)-3-phenyl-2-[2-(5,6,7,8-tetrahydronaphthalen-1-yloxy)acetamido]propanamido]propanoic acid (EH-001)</u>

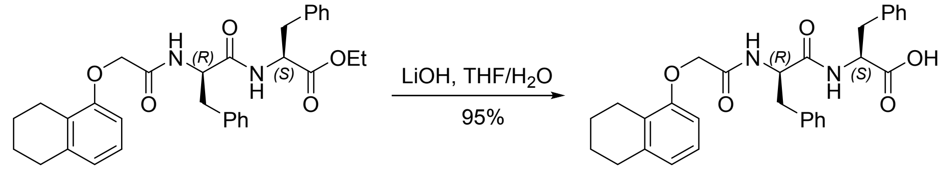

EG-006 **EH-001**

To a solution of **EG-006** (1.68 g, 3.18 mmol) in tetrahydrofuran (20 mL) was added a solution of lithium hydroxide (4 eq, 304 mg) in water (10 mL) and the mixture was stirred overnight. I was then poured into 1M hydrochloric acid (*ca.* 200 mL) and stirred for three hours. The solids were filtered, washed in the filter with 1 M hydrochloric acid, then water, and suction dried. Further drying by azeotropic distillation from acetonitrile afforded the title compound as a white solid (1.51 g, 95%).

δ_H (400 MHz, DMSO-d$_6$) 12.86 (1H, br s, COO$\underline{H}$), 8.59 (1H, d, *J* 8.36, N$\underline{H}$C$_b$H*), 7.68 (1H, d, *J* 8.52, N$\underline{H}$C$_a$H*), 7.32-7.29 (4H, m, $\underline{H}_{Ar}$), 7.24-7.18 (1H, m, $\underline{H}_{Ar}$), 7.16-7.10 (3H, m, $\underline{H}_{Ar}$), 6.95 (1H, t, *J* 7.88, $\underline{H}_{Ar}$), 6.86-6.85 (2H, m, $\underline{H}_{Ar}$), 6.66 (1H, d, *J* 7.56, $\underline{H}_{Ar}$), 6.50-6.48 (1H, d, *J* 8.04, $\underline{H}_{Ar}$), 4.67 (1H, td, *J* 8.35, 4.46, C$_a\underline{H}^*$), 4.48-4.42 (1H, m, C$_b\underline{H}^*$), 4.42 (1H, d, *J* 14.73, OC$\underline{H}_a$H$_b$), 4.35 (1H, d, *J* 14.73, OCH$_a\underline{H}_b$), 3.13 (1H, dd, *J* 13.67, 4.42, C$_b$H*C$\underline{H}_a$H$_b$Ph), 2.84 (1H, dd, *J* 13.67, 10.30, C$_b$H*CH$_a\underline{H}_b$Ph), 2.77 (1H, dd, *J* 13.63, 4.22, C$_a$H*C$\underline{H}_a$H$_b$Ph), 2.68-2.65 (2H, m, (C$\underline{H}_2$)$_4$), 2.62 (1H, dd, *J* 13.87, 8.42, C$_a$H*CH$_a\underline{H}_b$Ph), 2.54-2.41 (2H, m, overlapped by DMSO peak, (C$\underline{H}_2$)$_4$), 1.70-1.63 (4H, m, (C$\underline{H}_2$)$_4$). δ_H (400 MHz, acetone-d$_6$) 11.25 (1H, br s, COO$\underline{H}$), 7.61 (1H, d, *J* 8.20, N$\underline{H}$), 7.33-7.27 (5H, m, N$\underline{H}$ and $\underline{H}_{Ar}$), 7.24-7.20 (1H, m, $\underline{H}_{Ar}$), 7.18-7.14 (3H, m, $\underline{H}_{Ar}$), 7.02-6.96 (3H, m, $\underline{H}_{Ar}$), 6.70 (1H, d, *J* 7.60, $\underline{H}_{Ar}$), 6.61 (1H, d, *J* 8.12, $\underline{H}_{Ar}$), 4.84-4.79 (1H, m, C$\underline{H}^*$), 4.73 (1H, td, *J* 8.50, 4.96, C$\underline{H}^*$), 4.43 (1H, d, *J* 14.69, OC$\underline{H}_a$H$_b$), 4.34 (1H, d, *J* 14.69, OCH$_a\underline{H}_b$), 3.21 (1H, dd, *J* 13.89, 4.92, PhC$\underline{H}_2$), 3.05 (1H, dd, *J* 13.95, 5.42, PhC$\underline{H}_2$), 2.99 (1H, dd, *J* 13.91, 8.82, PhC$\underline{H}_2$), 2.92 (1H, dd, *J* 13.85, 7.04, PhC$\underline{H}_2$), 2.73-2.70 (2H, m, (C$\underline{H}_2$)$_4$), 2.60-2.46 (2H, m, (C$\underline{H}_2$)$_4$), 1.77-1.69 (4H, m, (C$\underline{H}_2$)$_4$). δ_C (100 MHz, DMSO-d$_6$) 172.86, 170.37, and 167.19 ($\underline{C}$=O), 155.13, 137.93, 137.54, 136.84, 129.28, 129.24, 128.23, 127.88, 126.56, 126.21, 125.73, 125.07, 121.84, and 108.42 ($\underline{C}_{Ar}$), 66.73 (O$\underline{C}$H$_2$), 53.60 ($\underline{C}_b$H*), 52.66 ($\underline{C}_a$H*), 37.89 (C$_a$H$^*\underline{C}$H$_2$Ph), 36.96 (C$_b$H$^*\underline{C}$H$_2$Ph), 28.99, 22.56, and 22.31 (($\underline{C}$H$_2$)$_4$). HRMS (ESI) m/z: [M+Na]$^+$ calcd for $C_{30}H_{32}N_2NaO_5$ 523.2203; found 523.2184.

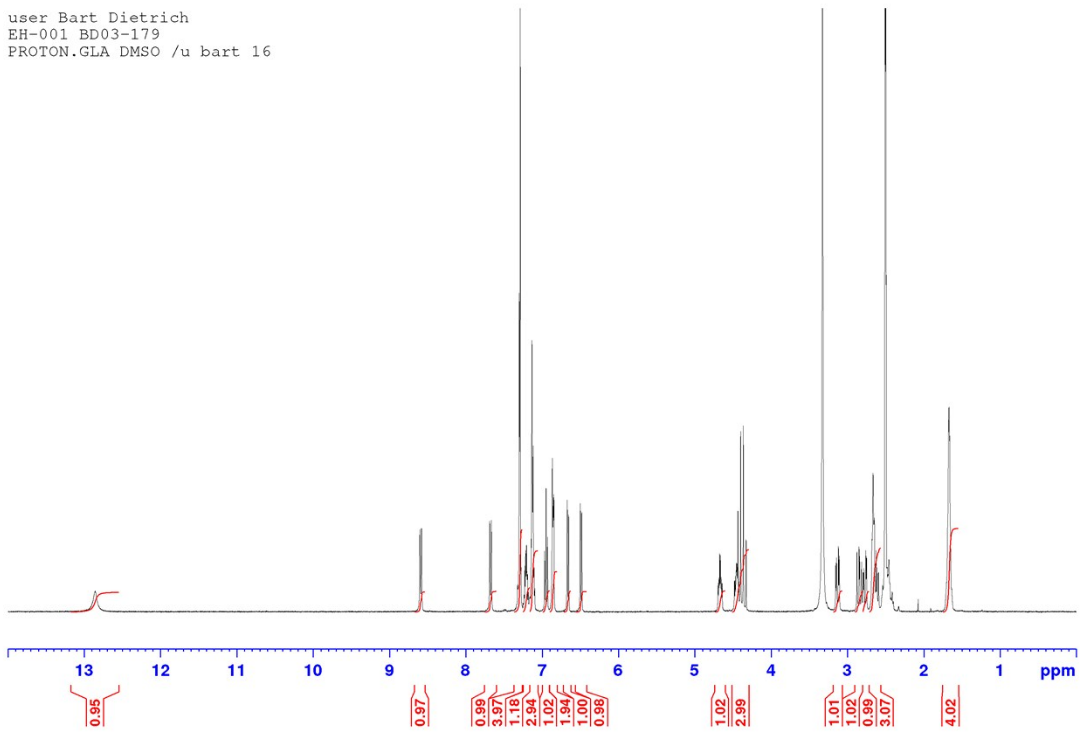

Figure S68. Proton NMR of **EH-001** in DMSO-d_6.

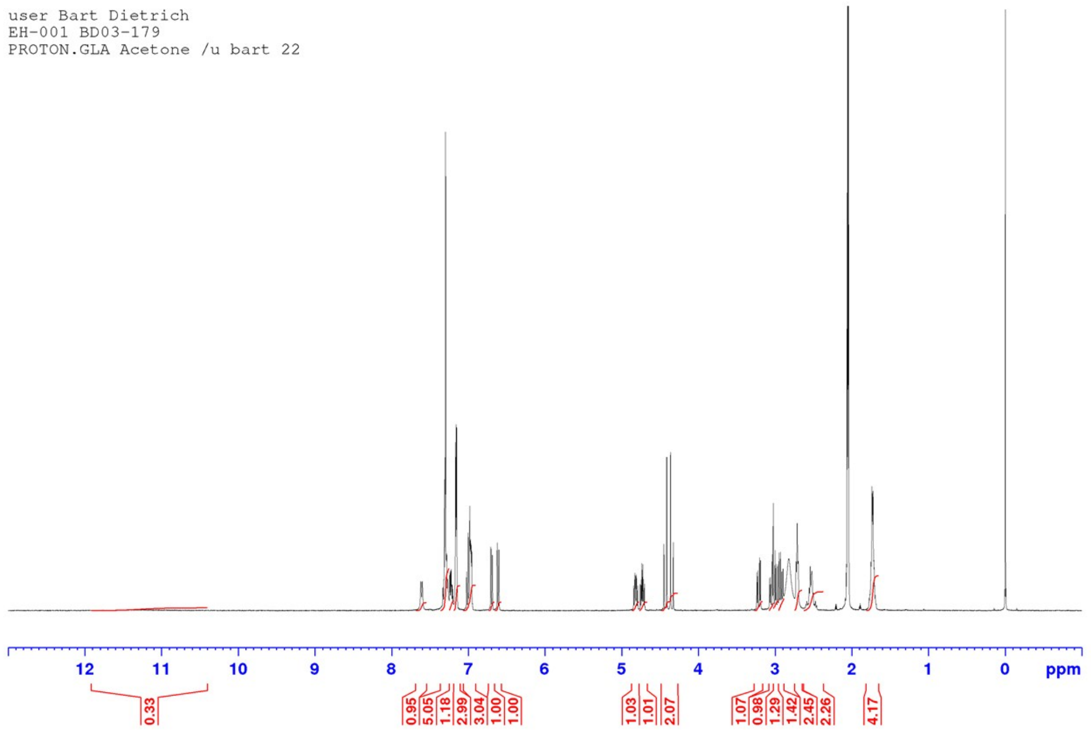

Figure S69. Proton NMR of **EH-001** in acetone-d_6.

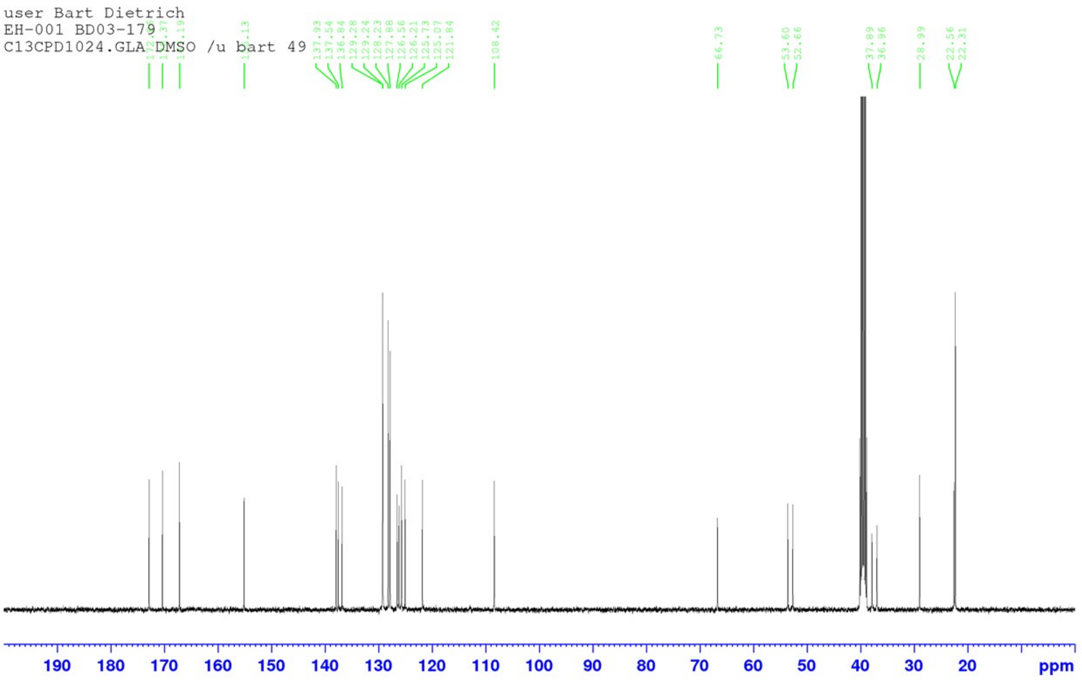

Figure S70. Carbon NMR of **EH-001**.

Ethyl 2-{2-[2-(naphthalen-2-yloxy)acetamido]-3-phenylpropanamido}-3-phenylpropanoate (EE-007)

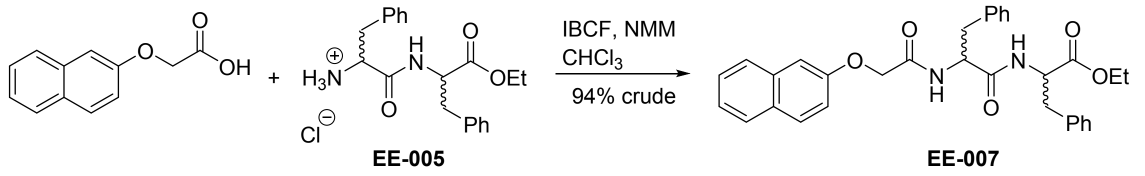

To a suspension of 2-(naphthalen-2-yloxy)acetic acid (751 mg, 3.71 mmol) in chloroform (20 mL) were added *iso*butyl chloroformate (1.01 eq, 486 μL) and *N*-methylmorpholine (1.1 eq, 449 μL) and the mixture was stirred for 30 minutes. **EE-005** (1 eq, 1.40 g) and another portion of *N*-methylmorpholine (1.1 eq, 449 μL) were added and the mixture was stirred overnight. After this time, it was diluted with chloroform, washed in turn with 1M hydrochloric acid and brine, dried ($MgSO_4$), and evaporated to dryness under reduced pressure. Crude **EE-007** was thus obtained as a grey foam (1.84 g, 94%) and used as is in the next step. A small amount was purified *via* column chromatography (1:9 ethyl acetate/dichloromethane) to afford a sample for characterisation (sticky white solid). The NMR spectra of the material are complex

due to the presence of diastereoisomers but identical to an overlay of the individual spectra of the enantiopure (*S*,*R*) (or (*R*,*S*)) and (*S*,*S*) (or (*R*,*R*)) isomers.

δ_H (400 MHz, DMSO-d$_6$) 8.69 (0.5H, d, *J* 8.04, N<u>H</u>), 8.61 (0.5H, d, *J* 7.48, N<u>H</u>), 8.16 (0.5H, d, *J* 8.48, N<u>H</u>), 8.11 (0.5H, d, *J* 8.48, N<u>H</u>), 7.85-7.82 (2H, m, $\underline{H}_{Ar}$), 7.73 (1H, d, *J* 8.12, $\underline{H}_{Ar}$), 7.48 (1H, m, $\underline{H}_{Ar}$), 7.38-7.34 (1H, m, $\underline{H}_{Ar}$), 7.29-7.11 (11H, m, $\underline{H}_{Ar}$), 7.04-7.01 (1H, m, $\underline{H}_{Ar}$), 4.69-4.62 (1H, m, C$\underline{H}^*$), 4.55 (1H, s, OC$\underline{H}_2$), 4.54 (1H, s, OC$\underline{H}_2$), 4.51-4.45 (1H, m, C$\underline{H}^*$), 4.06 (1H, q, *J* 7.12, C$\underline{H}_2$CH$_3$), 4.04 (1H, q, *J* 7.11, C$\underline{H}_2$CH$_3$), 3.08-2.93 (2H, m, PhC$\underline{H}_2$), 2.90-2.82 (1H, m, PhC$\underline{H}_2$), 2.77 (0.5H, dd, *J* 13.65, 4.12, PhC$\underline{H}_2$), 2.66 (0.5H, dd, *J* 13.61, 9.28, PhC$\underline{H}_2$), 1.12 (1.5H, t, *J* 7.08, CH$_2$C$\underline{H}_3$), 1.09 (1.5H, t, *J* 7.10, CH$_2$C$\underline{H}_3$). δ_C (100 MHz, DMSO-d$_6$) 171.42, 171.27, 171.04, 170.85, 167.31, and 167.24 ($\underline{C}$=O), 155.53, 137.51, 137.36, 137.11, 136.97, 134.07, 129.38, 129.29, 129.24, 129.20, 129.13, 128.78, 128.26, 128.02, 127.97, 127.52, 126.81, 126.65, 126.59, 126.43, 126.32, 126.27, 123.87, 118.49, 107.34, and 107.33 ($\underline{C}_{Ar}$), 66.71 (O$\underline{C}$H$_2$), 60.70 and 60.58 ($\underline{C}$H$_2$CH$_3$), 53.76, 53.68, 53.32, and 53.24 ($\underline{C}$H*), 37.76, 37.51, 36.99, and 36.74 (Ph$\underline{C}$H$_2$), 13.95 and 13.94 (CH$_2\underline{C}$H$_3$). HRMS (ESI) m/z: [M+Na]$^+$ calcd for $C_{32}H_{32}N_2NaO_5$ 547.2203; found 547.2185.

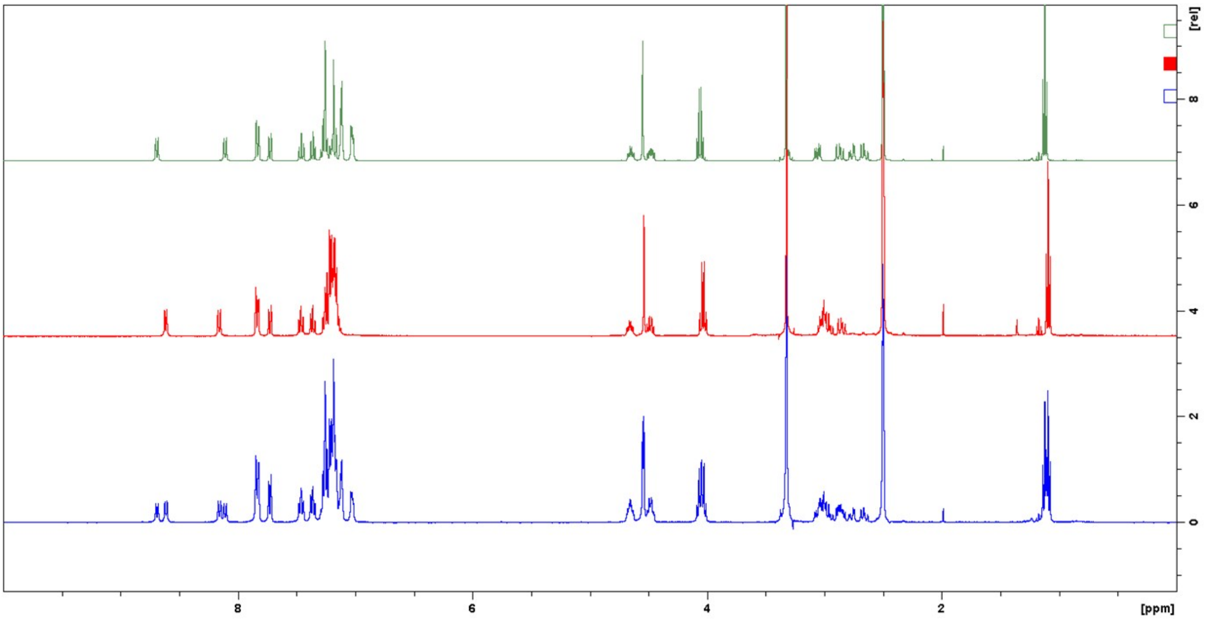

Figure S71. Overlay of the proton NMR spectra of isomers of **EE-007**: top: (*D*,*L*) (**EE-003**), middle: (*L*,*L*) (**DK-001**), bottom: (*rac*) (**EE-007**).

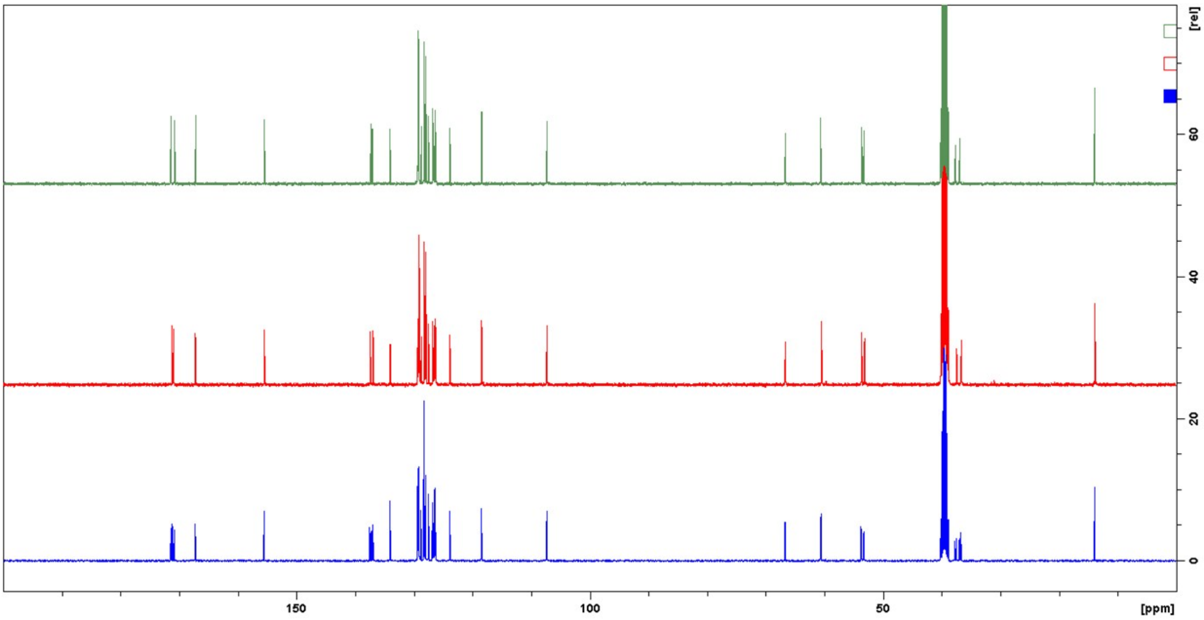

Figure 72. Overlay of the carbon NMR spectra of isomers of **EE-007**: top: (*D*,*L*) (**EE-003**), middle: (*L*,*L*) (**DK-001**), bottom: (*rac*) (**EE-007**).

2-{2-[2-(Naphthalen-2-yloxy)acetamido]-3-phenylpropanamido}-3-phenylpropanoic acid (EE-009)

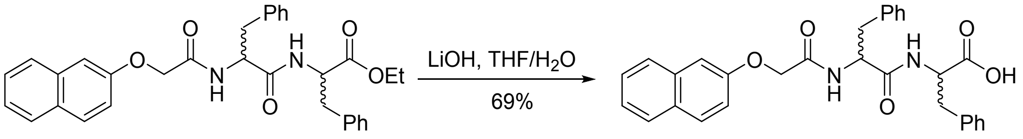

EE-007 **EE-009**

To a solution of crude **EE-007** (1.71 g, 3.26 mmol) in tetrahydrofuran (20 mL) was added a solution of lithium hydroxide (4 eq, 312 mg) and the mixture was stirred overnight. After this time, the mixture was poured into 1 M hydrochloric acid (350 mL). Some precipitate was observed alongside a yellow oil collecting at the bottom of the flask. The materials excluded from the aqueous layer were extracted with dichloromethane (2x) and the combined organics were washed in turn with 1 M hydrochloric acid, then brine, dried ($MgSO_4$), and evaporated under reduced pressure. The resulting light brown foam (1.53 g) was washed with a small amount of acetonitrile and dried under vacuum, affording the title compound as a white solid (957 mg, 59%). NMR analysis indicated a shift in the ratio of (S,S)/(R,R) to (S,R)/(R,S) isomers from the expected 50:50 to *approx.* 62:38. Meanwhile, the acetonitrile washings had formed a white crystalline precipitate. This was filtered off, washed with a small amount of acetonitrile and dried under vacuum. This second crop of title compound (219 mg, 10%) consisted of an *approx.* 9:91 ratio of (S,S)/(R,R) to (S,R)/(R,S) isomers. Combining both crops and

homogenising the mixture (by dissolution in boiling acetonitrile and subsequent evaporation under vacuum) afforded the final batch of title compound (1.12 g, 69%) as a white solid and in close (44:56) to the expected (50:50) ratio of (S,S)/(R,R) to (S,R)/(R,S) isomers. NMR data is complex due to the presence of diastereoisomers but is identical to an overlay of the NMR spectra of optically pure (S,S) (or (R,R)) and (S,R) (or (R,S)) isomers.

δ_H (400 MHz, DMSO-d$_6$) 12.84 (1H, br s, COOH), 8.58 (0.56H, d, *J* 8.44, NH), 8.44 (0.44H, d, *J* 7.80, NH), 8.12 (0.44H, d, *J* 8.56, NH), 8.05 (0.56H, d, *J* 8.52, NH), 7.84-7.82 (2H, m, H_{Ar}), 7.73 (1H, d, *J* 8.36, H_{Ar}), 7.48-7.44 (1H, m, H_{Ar}), 7.38-7.34 (1H, m, H_{Ar}), 7.27-7.08 (11H, m, H_{Ar}), 6.98-6.96 (1H, m, H_{Ar}), 4.67-4.62 (1H, m, CH*), 4.54-4.53 (2H, m, OCH$_2$), 4.50-4.44 (1H, m, CH*), 3.13-3.00 (1.5H, m, PhCH$_2$), 2.95-2.81 (1.5H, m, PhCH$_2$), 2.77-2.59 (1H, m, PhCH$_2$). δ_C (100 MHz, DMSO-d$_6$) 172.84, 172.69, 170.81, 170.58, 167.20, and 167.10 (C=O), 155.48, 137.49, 137.35, 137.30, 134.02, 129.35, 129.26, 129.23, 129.16, 129.11, 128.74, 128.17, 127.96, 127.89, 127.49, 126.77, 126.51, 126.42, 126.23, 126.18, 123.84, 118.43, 118.41, 107.35, and 107.33 (C_{Ar}), 66.69 (OCH$_2$), 53.48 and 53.22 (CH*), 37.75, 37.42, 37.02, and 36.69 (PhCH$_2$). HRMS (ESI) m/z: [M+Na]$^+$ calcd for $C_{30}H_{28}N_2NaO_5$ 519.1890; found 519.1871.

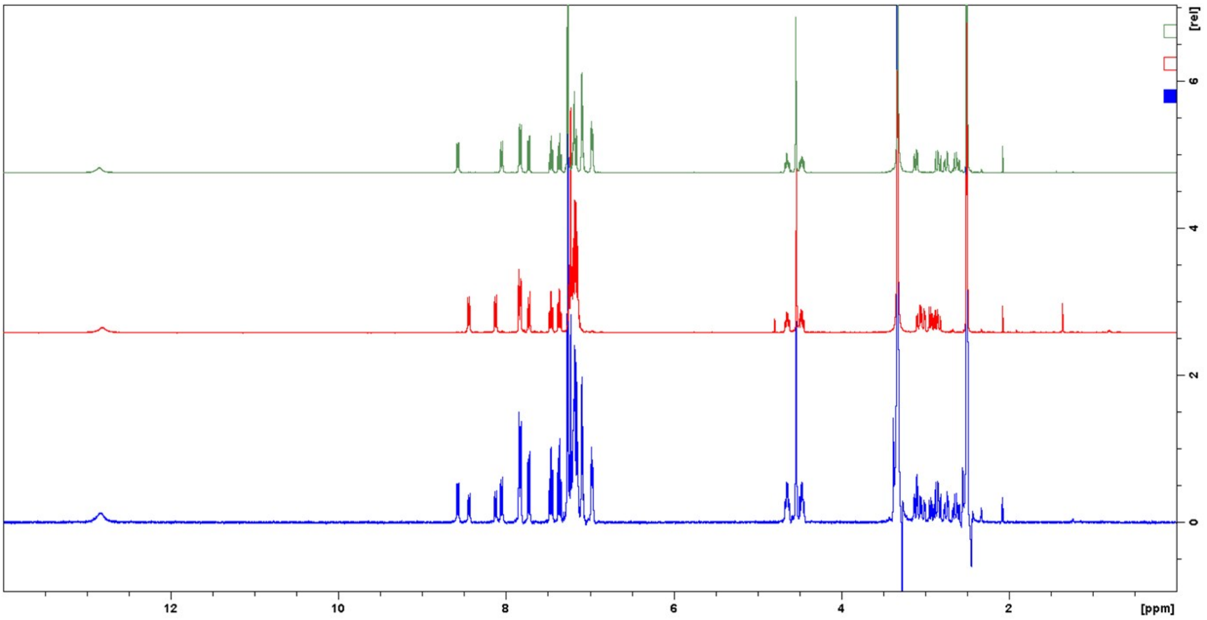

Figure 73. Overlay of the proton NMR spectra of isomers of **EE-009**: top: (*D*,*L*) (**EE-006**), middle: (*L*,*L*) (**DK-002**), bottom: (*rac*) (**EE-009**). This Figure shows an overlay of the proton NMR spectra of the (D,L), (L,L), and (rac) isomers (top, middle, and bottom, respectively). The spectra of the (L,D) and (D,D) isomers are not shown, but they are identical to those of their (D,L) and (L,L) enantiomers, as expected. The differences between the spectra of the diastereoisomers (D,L) and (L,L) (and by inference (D,L) and (D,D), (L,D) and (L,L), and (L,D) and (D,D)) are most clearly observed in the regions 8.70-8.00 ppm (the NH protons) and 3.20-2.55 ppm (the benzylic protons).

Any racemisation during the synthesis of the optically pure 2NapFF isomers would result in the formation of (at least) one of the diastereoisomers. Since the proton NMR spectra of the diastereoisomers are obviously distinct in the regions of interest, their examination allows us to pronounce on the optical purity of the final compounds (see expansions), at least to the extent that the sample concentrations and instrument acquisition parameters used allow. The benzylic region of the (D,L) isomer (EE-006) contains no observable signal from its diastereomers, while the NH region shows a hint of the NH peaks of its diastereomers in the baseline. Tentative integration of these signals suggests a less than 2% contribution of diastereomers in EE-006. Similarly, the benzylic region of the (L,L) isomer (DK-002) shows a barely distinguishable hint of diastereomer signals in the baseline, while the NH region shows weak peaks which, when integrated, would suggest at worst a 5% contribution of diastereomers to DK-002. Although not discussed above, the NMR spectra of the (L,D) and (D,D) compounds (EF-006 and DG-003, respectively) present a similar picture.

Integrations of the diastereoisomer peaks in the spectrum of (rac)-2NapFF (EE-009) allow us to estimate the relative contributions of (D,L)/(L,D) and (D,D)/(L,L) pairs to the mixture. The NH peaks of the (L,L)/(D,D) components are visibly smaller than those of their (L,D)/(D,L) stereoisomers. Their relative integrals suggest at worst an 8% skew from true racemate in favour of the (L,D)/(D,L) enantiomeric pair, that is a 42:58 distribution, or 16% d.e. A similar exercise can be carried out on the benzylic region of the spectrum. Although slightly less straightforward due to peak overlaps, the result should be more reliable as no exchangeable protons are involved. Calculations suggest at worst a 4% skew from true racemate in favour of the (L,D)/(D,L) enantiomeric pair, that is a 46:54 distribution, or 8% d.e. The presence of skew in the first place is explained by how EE-009 was purified: fractional crystallisation provided two oppositely enriched crops which were recombined and homogenised, as detailed in the experimental section. Various losses (predominantly in the mother liquor but also samples removed for characterisation) resulted in a deviation from the expected 50:50 stereoisomeric distribution. The NMR data of EE-009 do not allow to estimate the distribution of enantiomers in the mixture, but enantiomeric enrichment is not expected in a racemic environment.

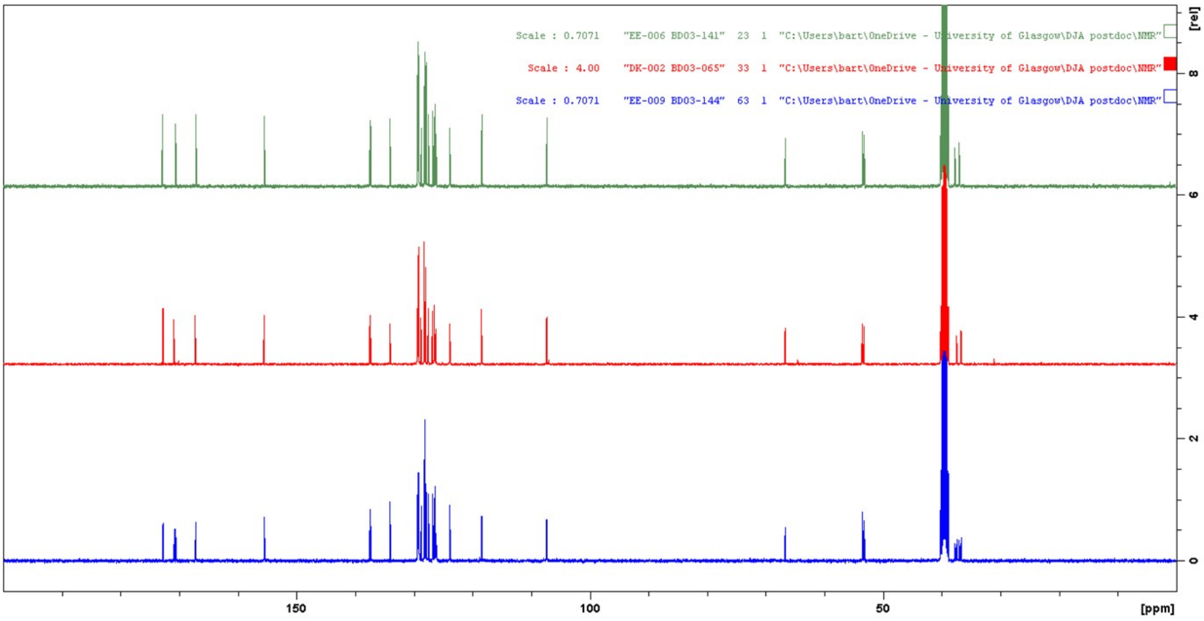

Figure 74. Overlay of the carbon NMR spectra of isomers of **EE-009**: top: (*D*,*L*) (**EE-006**), middle: (*L*,*L*) (**DK-002**), bottom: (*rac*) (**EE-009**).

Ethyl 3-phenyl-2-{3-phenyl-2-[2-(5,6,7,8-tetrahydronaphthalen-1-yloxy)acetamido]propanamido}propanoate (EG-002)

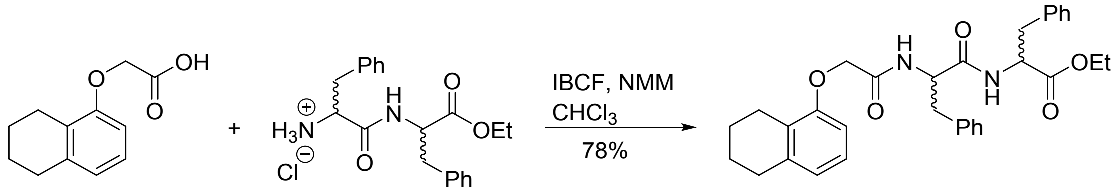

To a solution of 2-(5,6,7,8-tetrahydronaphthalen-1-yloxy)acetic acid (323 mg, 1.57 mmol) in chloroform (10 mL) was added *N*-methylmorpholine (1.1 eq, 190 µL) followed by *iso*butyl chloroformate (1.05 eq, 214 µL) and the mixture was stirred for 15 minutes. **EE-005** (1 eq, 591 mg) was then added, followed by another portion of *N*-methylmorpholine (1.1 eq, 190 µL) and the reaction mixture was stirred overnight. After this time, it was diluted with chloroform, washed in turn with 1 M hydrochloric acid, water, and brine, dried ($MgSO_4$), and evaporated under reduced pressure. The resulting pale-yellow, viscous oil was purified *via* column chromatography (1:9 ethyl acetate/dichloromethane, wet-loaded, *ca.* 8×3 cm), affording the title compound as an oil, which solidified on standing (649 mg, 78%). Proton and carbon NMR spectra are complex due to the presence of isomers, but are identical to overlays of the individual spectra of the pure (S,S) and (R,S) isomers.

δ_H (400 MHz, DMSO-d_6) 8.70 (0.5H, d, *J* 8.12, NH), 8.62 (0.5H, d, *J* 7.60, NH), 7.76 (0.5H, d, *J* 8.32, NH), 7.71 (0.5H, d, *J* 8.52, NH), 7.32-7.14 (9H, m, H_{Ar}), 6.96-6.91 (2H, m, H_{Ar}), 6.66 (1H, d, *J* 7.60, H_{Ar}), 6.48 (1H, dd, *J* 7.80, 5.64, H_{Ar}), 4.69-4.63 (1H, m, CH^*), 4.52-4.44 (1H, m, CH^*), 4.44-4.31 (2H, m, OCH_2), 4.09 (1H, q, *J* 7.06, CH_2CH_3), 4.05 (1H, q, *J* 7.07, CH_2CH_3), 3.10-2.68 (4H, m, $PhCH_2$), 2.68-2.63 (2H, m, CH_2CH_2), 2.54-2.42 (2H, m, overlapped by DMSO peak, CH_2CH_2), 1.72-1.63 (4H, m, CH_2CH_2), 1.14 (1.5H, t, *J* 7.18, CH_2CH_3), 1.11 (1.5H, t, *J* 7.18, CH_2CH_3). δ_C (100 MHz, DMSO-d_6) 171.36, 171.21, 170.71, 170.54, 167.31, 167.24, 155.15, 155.14, 137.90, 137.09, 136.94, 136.85, 129.27, 129.23, 129.20, 129.08, 128.24, 128.21, 127.97, 127.90, 126.63, 126.54, 126.32, 126.24, 125.69, 125.08, 125.06, 121.81, 108.42, 108.41, 66.73, 60.65, 60.56, 53.69, 53.63, 52.71, 37.82, 37.59, 36.89, 36.74, 28.97, 22.56, 22.30, 13.93, 13.90. HRMS (ESI) m/z: $[M+Na]^+$ calcd for $C_{32}H_{36}N_2NaO_5$ 551.2516; found 551.2502.

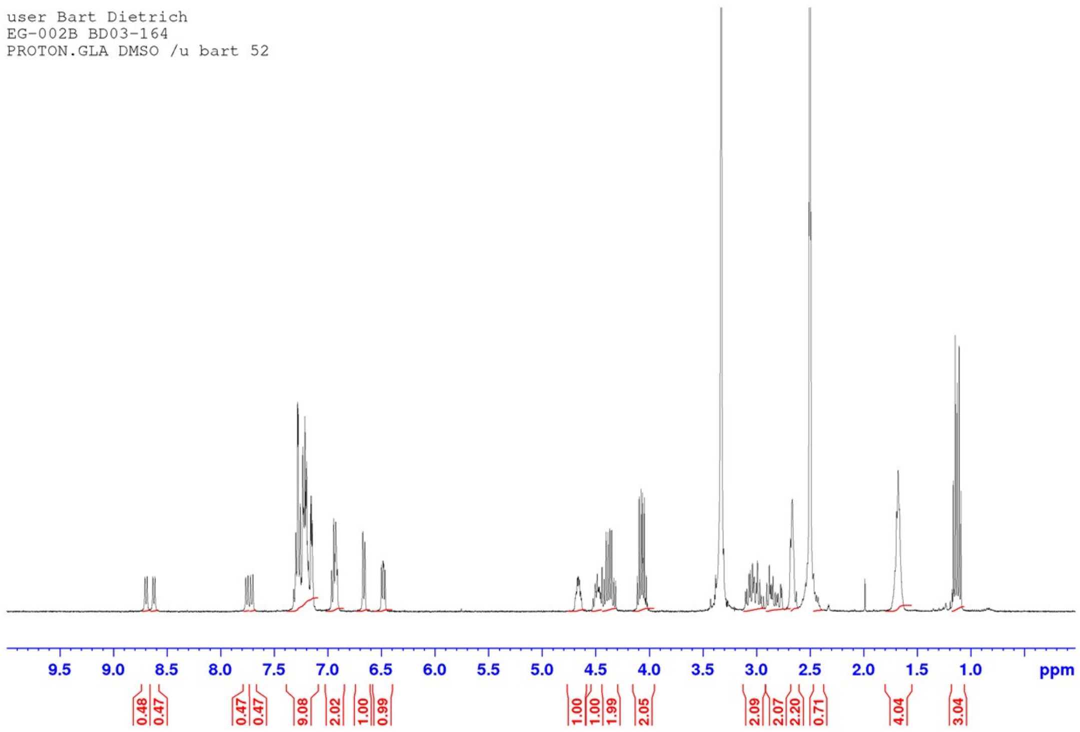

Figure 75. Proton NMR of **EG-002**.

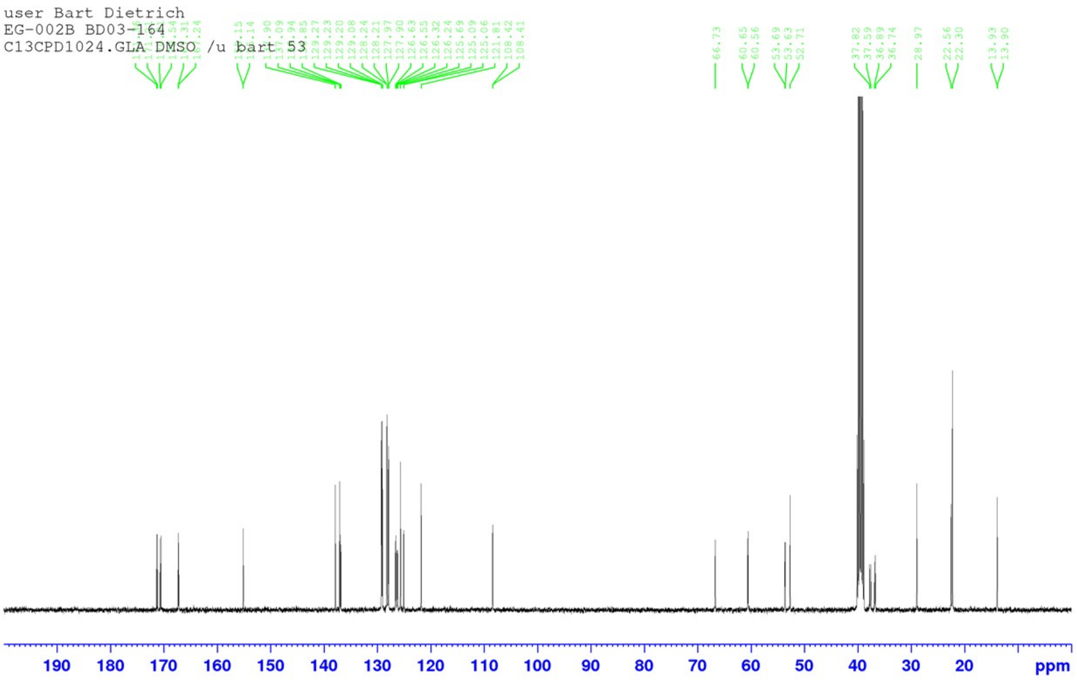

Figure 76. Carbon NMR of **EG-002**.

3-Phenyl-2-{3-phenyl-2-[2-(5,6,7,8-tetrahydronaphthalen-1-yloxy)acetamido]propanamido}propanoic acid (EG-003)

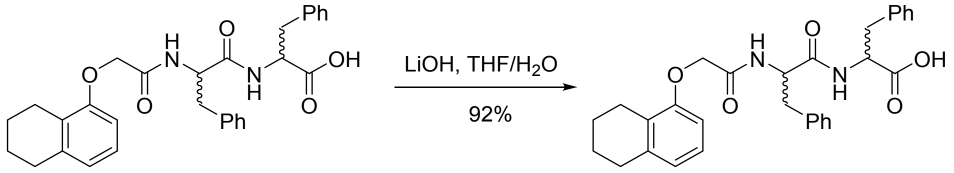

EG-002 **EG-003**

To a solution of **EG-002** (597 mg, 1.13 mmol) in tetrahydrofuran (10 mL) was added a solution of lithium hydroxide (4 eq, 108 mg) in water (10 mL) and the mixture was stirred overnight. After this time, it was poured into 1 M hydrochloric acid (*ca.* 300 mL), resulting in a sticky precipitate. This was extracted with dichloromethane (2×), the combined organics were washed with brine, dried ($MgSO_4$), and evaporated under reduced pressure, then further dried by azeotropic distillation from acetonitrile. The title compound **EG-003** was thus obtained as a white foam (520 mg, 92%) in >99% NMR purity (balance acetonitrile). Proton and carbon NMR spectra are complex due to the presence of isomers, but are identical to overlays of the individual spectra of pure (*S*,*S*) and (*R*,*S*) isomers.

δ_H (400 MHz, DMSO-d_6) 8.59 (0.5H, d, *J* 8.36, N$\underline{H}$), 8.46 (0.5H, d, *J* 7.96, N$\underline{H}$), 7.73 (0.5H, d, *J* 8.52, N$\underline{H}$), 7.68 (0.5H, d, *J* 8.56, N$\underline{H}$), 7.32-7.09 (9H, m, $\underline{H}_{Ar}$), 6.95 (1H, td, *J* 7.87, 1.90, $\underline{H}_{Ar}$), 6.87-6.84 (1H, m, $\underline{H}_{Ar}$), 6.66 (1H, d, *J* 7.64, $\underline{H}_{Ar}$), 6.50-6.46 (1H, m, $\underline{H}_{Ar}$), 4.69-4.62 (1H, m, C$\underline{H}^*$), 4.50-4.42 (1H, m, C$\underline{H}^*$), 4.43-4.31 (2H, m, OC$\underline{H}_2$), 3.15-2.59 (4H, m, PhC$\underline{H}_2$), 2.69-2.41 (4H, m, C$\underline{H}_2$CH_2), 1.72-1.63 (4H, m, C$\underline{H}_2$CH_2). δ_C (100 MHz, DMSO-d_6) 172.86, 172.72, 170.57, 170.37, 167.30, 167.20, 155.15, 155.13, 137.93, 137.55, 137.38, 137.11, 136.84, 129.35, 129.28, 129.24, 129.13, 128.23, 128.18, 127.98, 127.88, 126.57, 126.46, 126.31, 126.21, 125.73, 125.10, 125.07, 121.85, 108.46, 108.42, 66.74, 53.61, 53.47, 52.73, 52.66, 37.89, 37.57, 36.96, 36.74, 28.99, 22.57, 22.31. HRMS (ESI) m/z: $[M+Na]^+$ calcd for $C_{30}H_{32}N_2NaO_5$ 523.2203; found 523.2202.

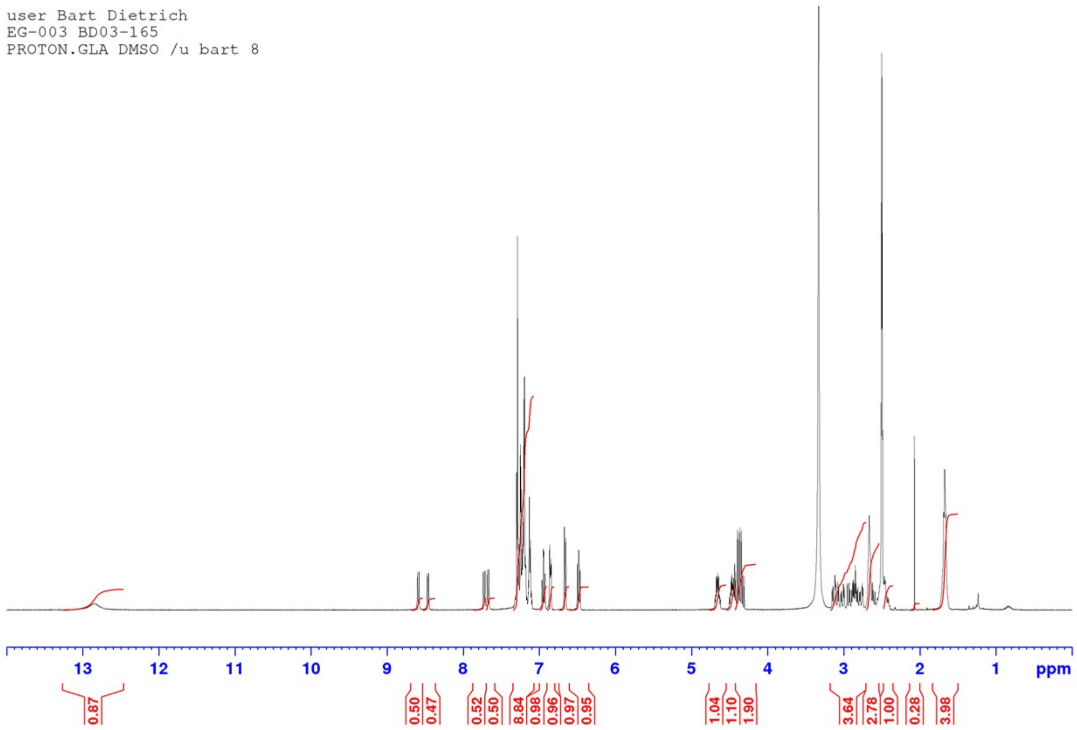

Figure 77. Proton NMR of **EG-003**.

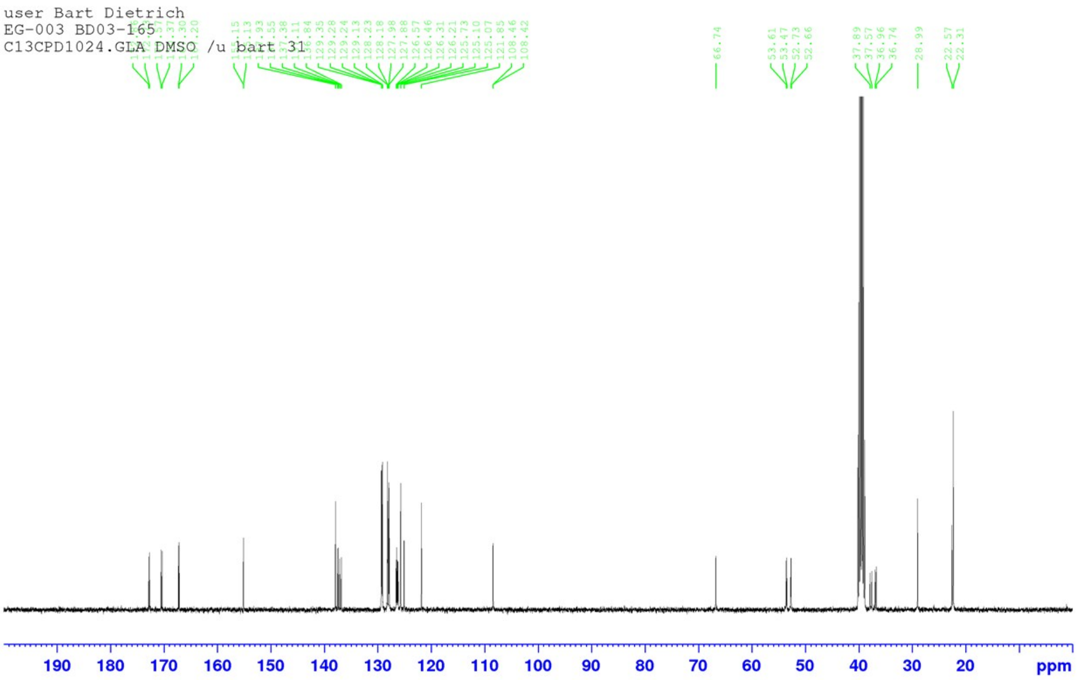

Figure 78. Carbon NMR of **EG-003**.

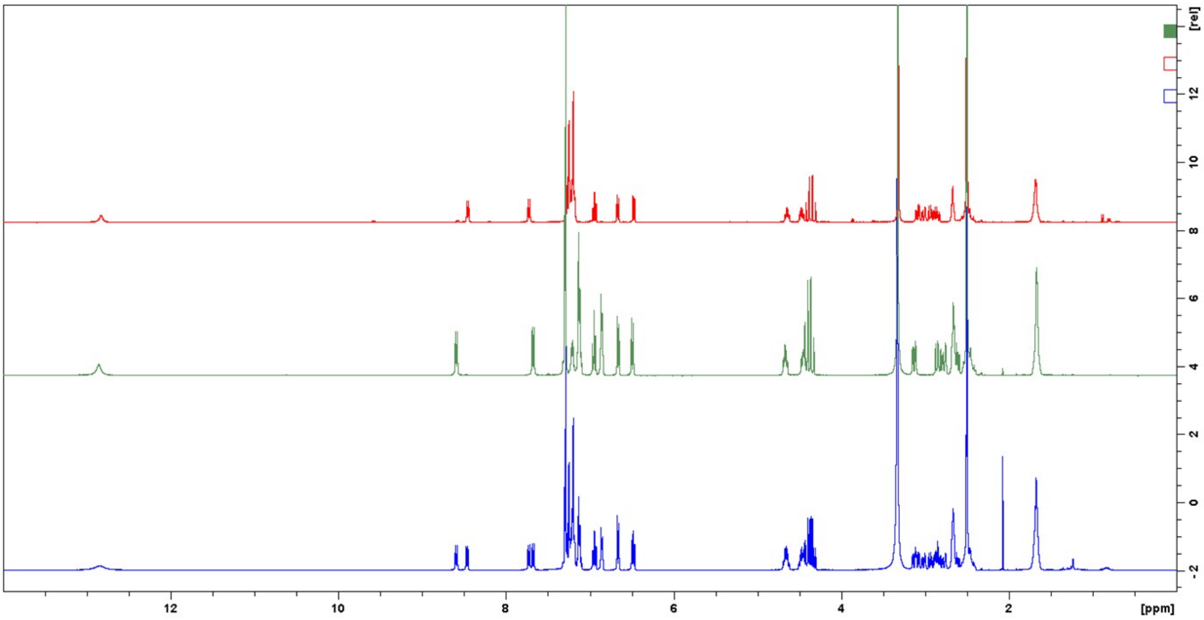

Figure 79. Overlay of the proton NMR spectra of stereoisomers of **EG-003**: top: (*L*,*L*), middle: (*D*,*L*) (**EH-001**), bottom: (*rac*) (**EG-003**).

Methyl (2S)-3-methyl-2-[2-(naphthalen-2-yloxy)acetamido]butanoate

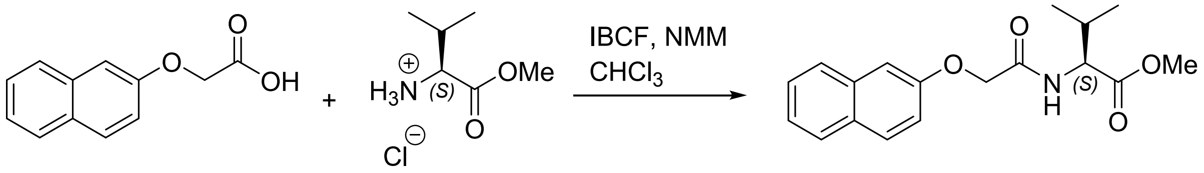

In a typical synthesis, 2-napthoxyacetic acid (2.76 g, 13.7 mmol) was suspended in chloroform (50 mL) and cooled in an ice bath. Valine ethyl ester (either the L-, the D-, or rac-) (3.44 g, 13.7 mmol) was added into a separate flask and suspended in chloroform (40 mL). To each flask was added *N*-methylmorpholine (1.50 mL, 13.7 mmol). Isobutylchloroformate (1.78 mL, 13.7 mmol) was added to the flask containing the 2-napthoxyacetic acid. After stirring for around 30 seconds, the contents of the other flask were added, and the flask rinsed with chloroform (10 mL) which was also added. After stirring overnight, allowing the temperature to increase to room temperature as the ice melts, the solution was washed sequentially with water (100 mL), dilute hydrochloric acid (100 mL, 1 M), and water (100 mL). The solution was dried with magnesium sulfate, filtered and the solvent removed in vacuo. The resulting solid was used directly in the next step with no purification.

(**2S)-3-Methyl-2-[2-(naphthalen-2-yloxy)acetamido]butanoic acid.**

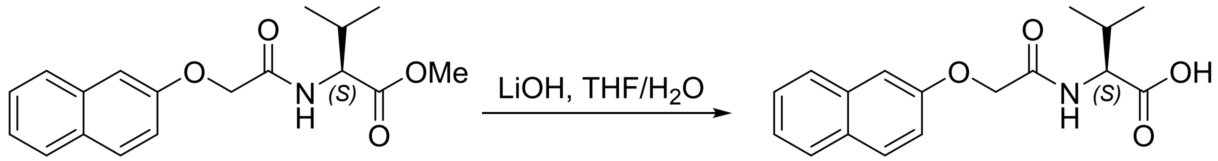

The solid from the above was dissolved in tetrahydrofuran (20 mL). Water (5 mL) was added, followed by lithium hydroxide (0.25 g). After a short period (around 5 minutes), the initial emulsion-like solution became transparent. A small sample was removed (around 0.1 mL) and added to water (10 mL). If any precipitate resulted, the solution was stirred for a further 5 minutes and re-checked. When no precipitate resulted, water (200 mL) was added to the solution, followed by hydrochloric acid (1M) until the pH was around 3. After stirring for around 5 minutes, the white solid was collected by filtration and dried on the filter. The solid was then recrystallised from ethanol to provide a pure white solid (2NapVOH).

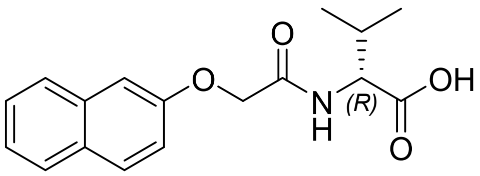

2NapV(D)OH. ^{1}H NMR δ_H (400 MHz, DMSO-d_6) 12.80 (1H, br s, COO<u>H</u>), 8.21 (1H, d, *J* 8.60, N<u>H</u>), 7.86-7.83 (2H, m, $\underline{H}_{Ar}$), 7.75 (1H, d, *J* 8.12, $\underline{H}_{Ar}$), 7.48-7.44 (1H, m, $\underline{H}_{Ar}$), 7.38-7.34 (1H, m, $\underline{H}_{Ar}$), 7.27-7.23 (2H, m, $\underline{H}_{Ar}$), 4.76 (1H, d, *J* 14.61, $OC\underline{H}_aH_b$), 4.71 (1H, d, *J* 14.65, $OCH_a\underline{H}_b$), 4.25 (1H, dd, *J* 8.60, 5.64, $C\underline{H}^*$), 2.17-2.09 (1H, m, $C\underline{H}(CH_3)_2$), 0.90 (6H, d, *J* 6.80, $CH(C\underline{H}_3)_2$). ^{13}C NMR δ_C (100 MHz, DMSO-d_6) 172.72, 167.71, 155.66, 134.01, 129.31, 128.68, 127.50, 126.62, 126.45, 123.78, 118.54, 107.19, 66.59, 56.84, 29.85, 19.10, 17.90. HRMS (EI) m/z: $[M]^+$ calcd for $C_{17}H_{19}NO_4$ 301.1314; found 301.1304.

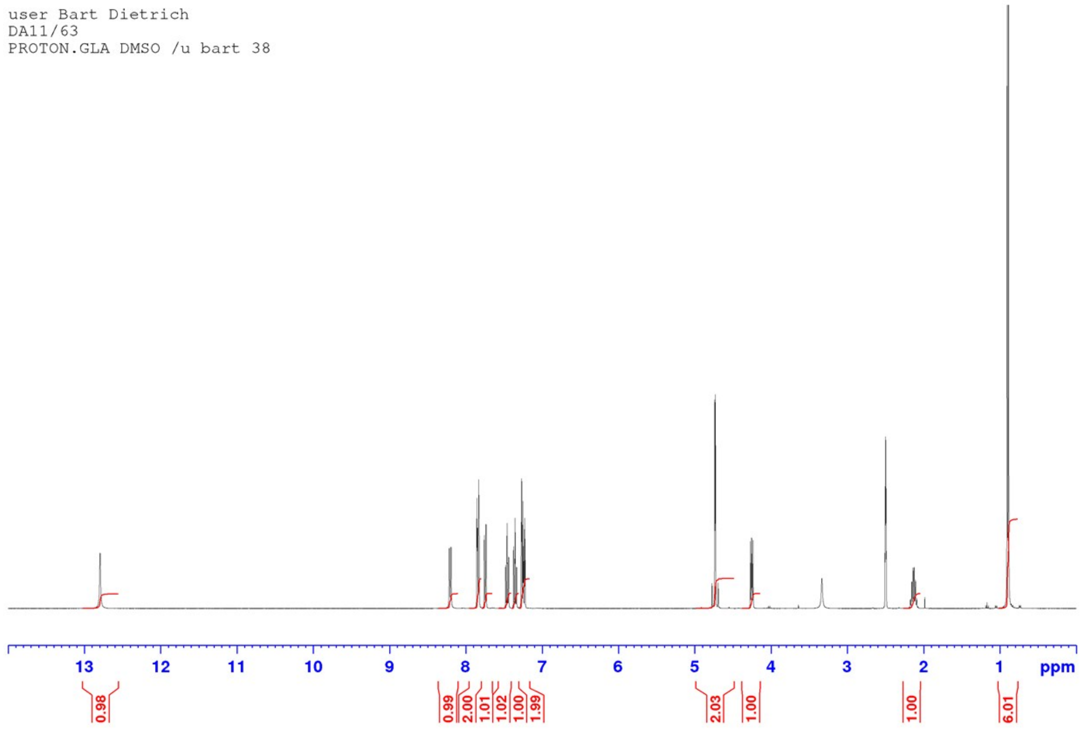

Figure 80: Proton NMR of 2NapV(D)OH.

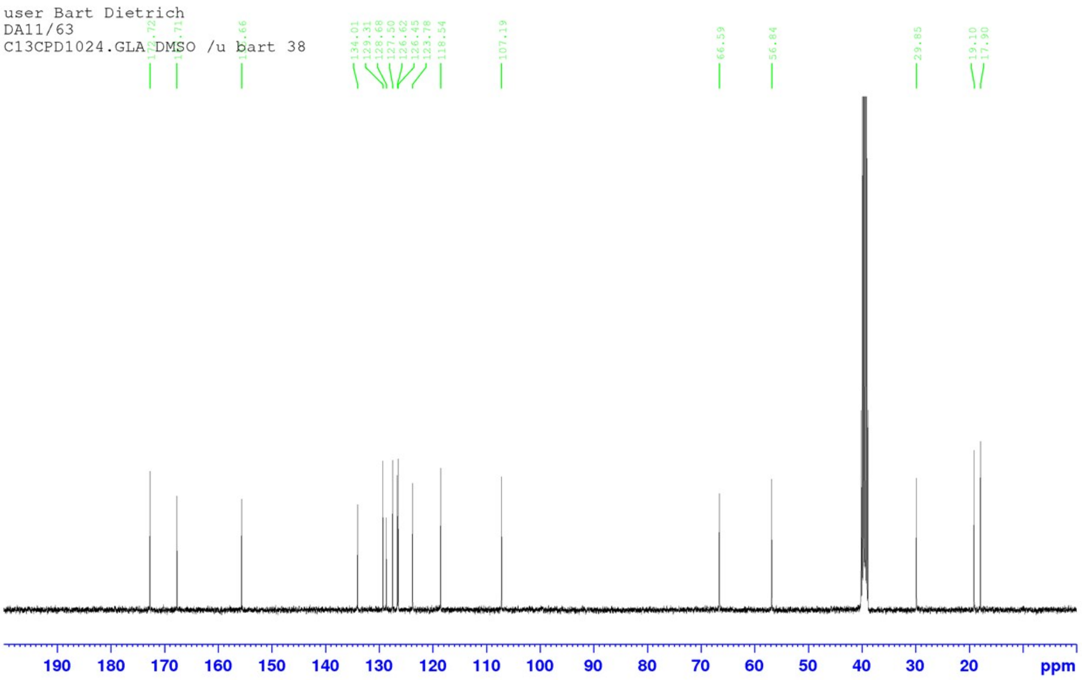

Figure 81: Carbon NMR of 2NapV(D)OH.

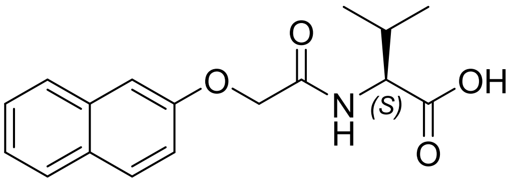

2NapV(L)OH. ^{1}H NMR δ_H (400 MHz, DMSO-d$_6$) 12.80 (1H, br s, COO<u>H</u>), 8.41 (0.13H, d, *J* 8.16, N<u>H</u>), 8.20 (0.87H, d, *J* 8.64, N<u>H</u>), 7.86-7.83 (2H, m, $\underline{H}_{Ar}$), 7.75 (1H, d, *J* 8.08, $\underline{H}_{Ar}$), 7.46 (1H, ddd, *J* 8.15, 6.91, 1.23, $\underline{H}_{Ar}$), 7.36 (1H, ddd, *J* 8.05, 6.91, 1.15, $\underline{H}_{Ar}$), 7.27-7.22 (2H, m, $\underline{H}_{Ar}$), 4.75 (1H, d, *J* 14.89, $OC\underline{H}_aH_b$), 4.71 (1H, d, *J* 14.56, $OCH_a\underline{H}_b$), 4.25 (1H, dd, *J* 8.66, 5.70, $C\underline{H}^*$), 2.17-2.07 (1H, m, $C\underline{H}(CH_3)_2$), 0.89 (6H, d, *J* 6.84, $CH(C\underline{H}_3)_2$). ^{13}C NMR δ_C (100 MHz, DMSO-d$_6$) 172.76, 167.75, 155.67, 134.03, 129.34, 128.69, 127.52, 126.64, 126.47, 123.80, 118.57, 107.19, 66.59, 56.86, 29.87, 19.13, 17.92. HRMS (ESI) m/z: $[M+Na]^+$ calcd for $C_{17}H_{19}NNaO_4$ 324.1206; found 324.1202.

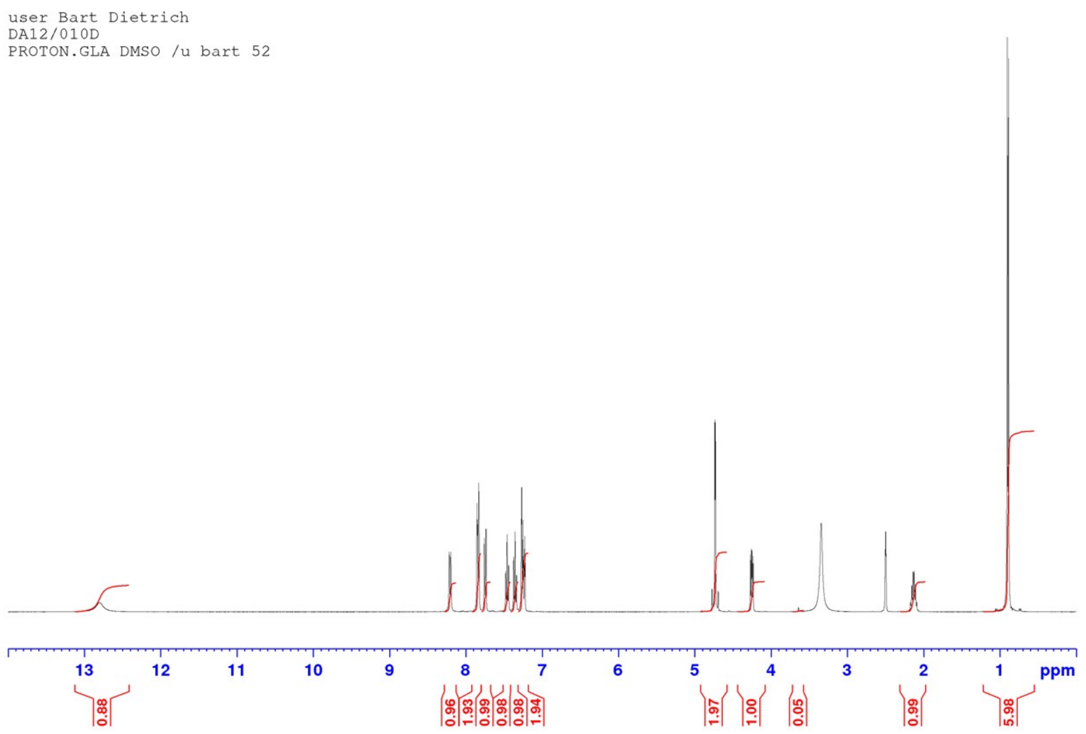

Figure 82: Proton NMR of 2NapV(L)OH.

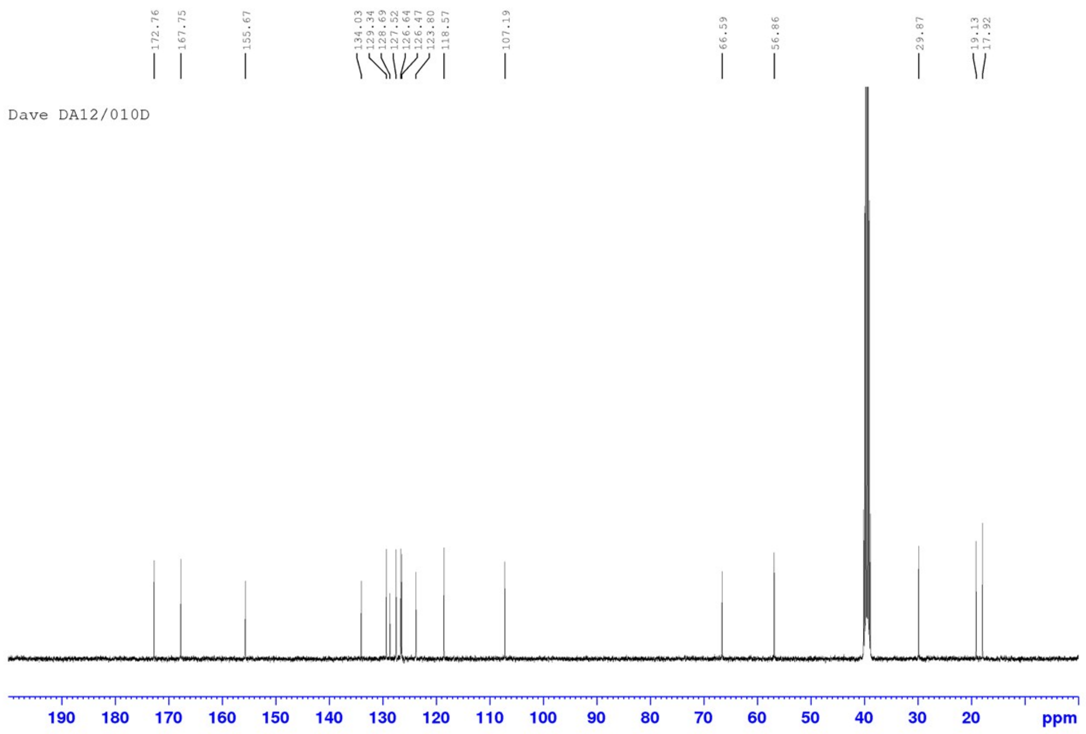

Figure 83: Carbon NMR of 2NapV(L)OH.

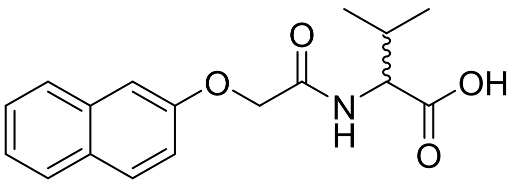

2NapV(rac)OH. δ_H (400 MHz, DMSO-d$_6$) 12.80 (1H, br s, COOH), 8.21 (1H, d, *J* 8.64, NH), 7.86-7.83 (2H, m, H_{Ar}), 7.75 (1H, d, *J* 8.08, H_{Ar}), 7.46 (1H, ddd, *J* 8.13, 6.89, 1.23, H_{Ar}), 7.36 (1H, ddd, *J* 8.08, 6.88, 1.20, H_{Ar}), 7.28-7.23 (2H, m, H_{Ar}), 4.76 (1H, d, *J* 14.57, OCH_aH_b), 4.71 (1H, d, *J* 14.57, OCH_aH_b), 4.26 (1H, dd, *J* 8.66, 5.66, CH^*), 2.18-2.09 (1H, m, $CH(CH_3)_2$), 0.90 (6H, d, *J* 6.88, $CH(CH_3)_2$). ^{13}C NMR δ_C (100 MHz, DMSO-d$_6$) 172.74, 167.73, 155.67, 134.03, 129.33, 128.69, 127.51, 126.63, 126.46, 123.79, 118.56, 107.19, 66.59, 56.85, 29.86, 19.11, 17.92. HRMS (EI) m/z: $[M]^+$ calcd for $C_{17}H_{19}NO_4$ 301.1314; found 301.1301.

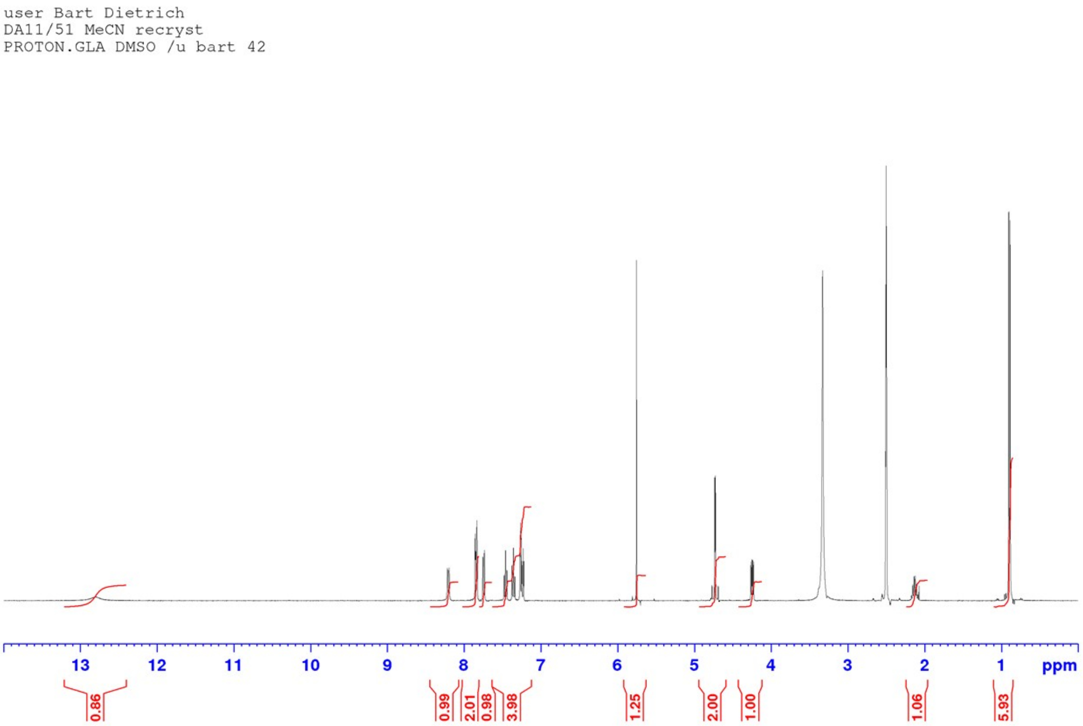

Figure 84: Proton NMR of 2NapV(rac)OH.

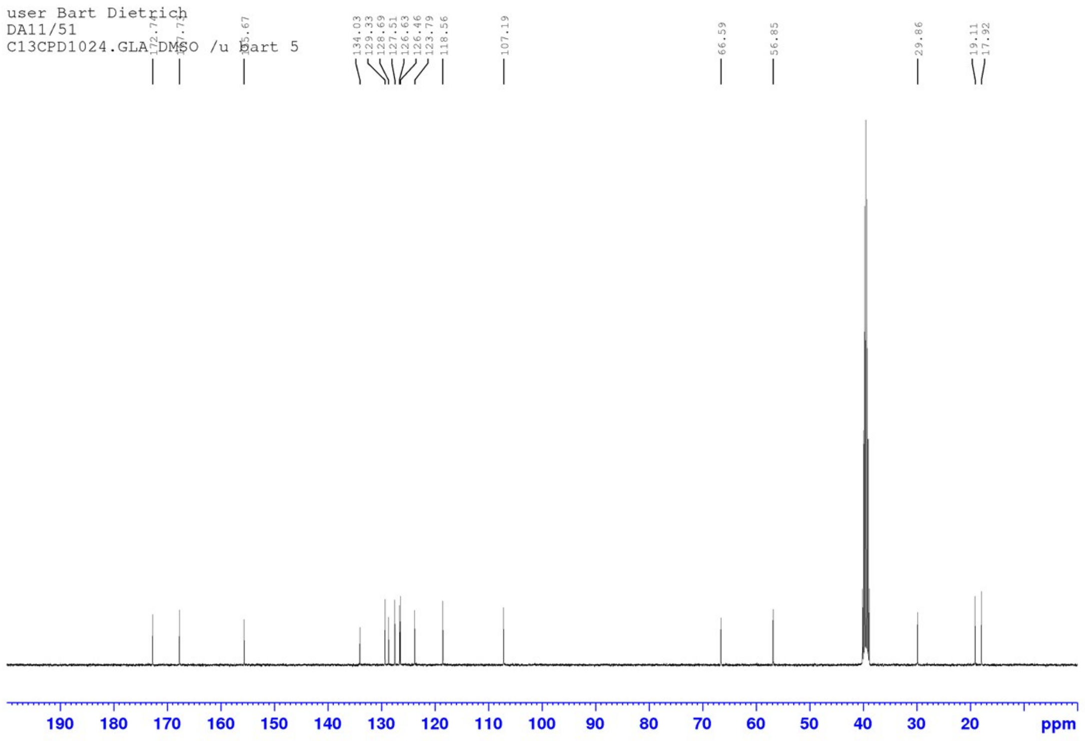

Figure 85: Carbon NMR of 2NapV(rac)OH.

Methyl 2-[(2*S*)-3-methyl-2-[2-(naphthalen-2-yloxy)acetamido]butanamido]acetate

In a typical procedure, 2NapVOH from above (2.33 g, 7.74 mmol) was suspended in chloroform (50 mL) and cooled in an ice bath. Glycine ethyl ester (1.08 g, 7.74 mmol) was added into a separate flask and suspended in chloroform (40 mL). To each flask was added *N*-methylmorpholine (0.85 mL, 7.74 mmol). Isobutylchloroformate (1.01 mL, 7.74 mmol) was added to the flask containing the 2-napthoxyacetic acid. After stirring for around 30 seconds, the contents of the other flask were added, and the flask rinsed with chloroform (10 mL) which was also added. After stirring overnight, allowing the temperature to increase to room temperature as the ice melts, the solution was washed sequentially with water (100 mL), dilute hydrochloric acid (100 mL, 1 M), and water (100 mL). The solution was dried with magnesium sulfate, filtered and the solvent removed in vacuo. The resulting solid was used directly in the next step with no purification.

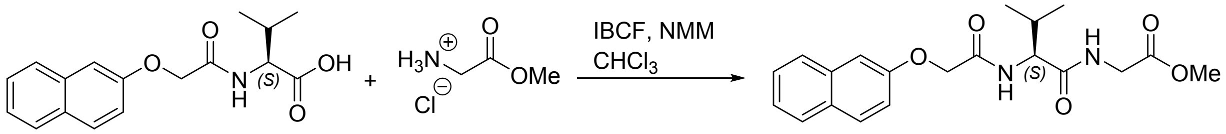

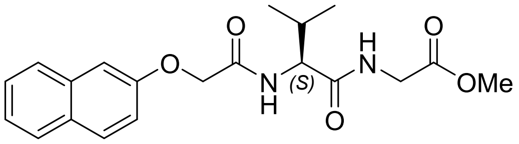

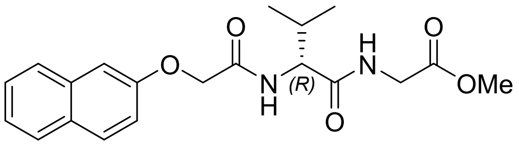

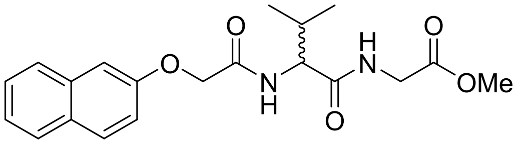

[(2*S*)-3-Methyl-2-[2-(naphthalen-2-yloxy)acetamido]butanamido]acetic acid (DL-001)

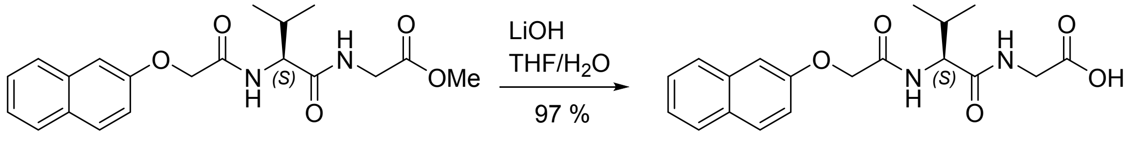

DK-005 **DL-001**

To a solution of **DK-005** (1.65 g, 4.43 mmol) in tetrahydrofuran (40 mL) was added a solution of lithium hydroxide (4 eq, 424 mg) in water (40 mL) and the mixture was stirred overnight. After this time, it was poured into 1M hydrochloric acid (*ca.* 400 mL) and stirred for one hour. Filtration, washing with water in the filter then drying by repeated azeotropic distillation with acetonitrile afforded the title compound as a white solid (1.41 g). The aqueous washings developed a precipitate on standing. This was filtered off, washed with water and dried as above to afford another crop of title compound (135 mg). Combined yield 1.54 g (97 %). Purity (NMR) 99.7 % (balance residual acetonitrile).

^{1}H NMR δ_H (400 MHz, DMSO-d$_6$) 12.55 (1H, br s, COOH), 8.44 (1H, t, *J* 5.86, NHCH$_2$), 8.01 (1H, d, *J* 9.04, NHCH*), 7.86-7.83 (2H, m, H$_{Ar}$), 7.75 (1H, d, *J* 8.08, H$_{Ar}$), 7.46 (1H, ddd, *J* 8.13, 6.91, 1.21, H$_{Ar}$), 7.36 (1H, ddd, *J* 8.08, 6.92, 1.16, H$_{Ar}$), 7.28 (1H, d, *J* 2.48, H$_{Ar}$), 7.24 (1H, dd, *J* 8.88, 2.60, H$_{Ar}$), 4.75 (1H, d, *J* 14.65, OCH$_a$H$_b$), 4.69 (1H, d, *J* 14.61, OCH$_a$H$_b$), 4.31 (1H, dd, *J* 9.04, 6.48, CH*), 3.80 (1H, dd, *J* 17.45, 5.88, NHCH$_a$H$_b$), 3.72 (1H, dd, *J* 17.45, 5.84, NHCH$_a$H$_b$), 2.08-2.00 (1H, m, CH(CH$_3$)$_2$), 0.88 (3H, d, *J* 6.76, CH(CH$_3$)$_2$), 0.84 (3H, d, *J* 6.80, CH(CH$_3$)$_2$). ^{13}C NMR δ_C (100 MHz, DMSO-d$_6$) 171.04 (2C overlapping), and 167.38 (C=O),

155.60, 134.05, 129.40, 128.73, 127.54, 126.65, 126.51, 123.84, 118.50, and 107.28 ($\underline{C}_{Ar}$), 66.72 ($O\underline{C}H_2$), 57.09 ($\underline{C}H^*$), 40.60 ($NH\underline{C}H_2$), 30.82 ($\underline{C}H(CH_3)_2$), 19.15 and 17.86 ($CH(\underline{C}H_3)_2$). HRMS (ESI) m/z: $[M+Na]^+$ calcd for $C_{19}H_{22}N_2NaO_5$ 381.1421; found 381.1407.

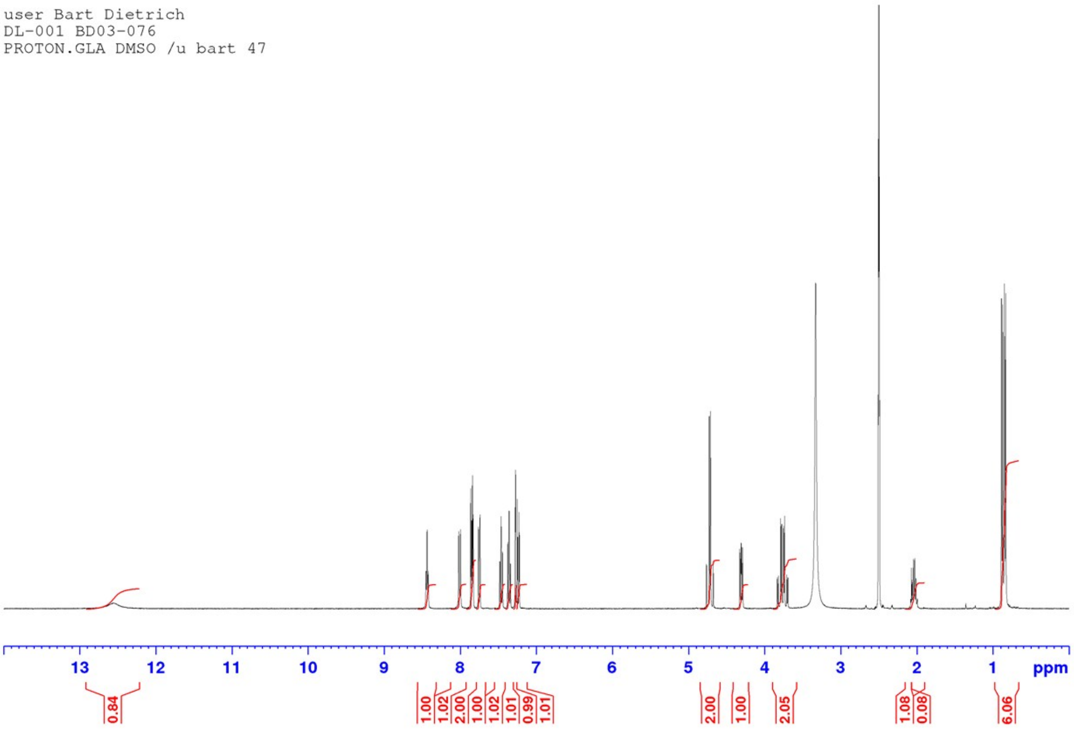

Figure 86: Proton NMR of **DL-001**.

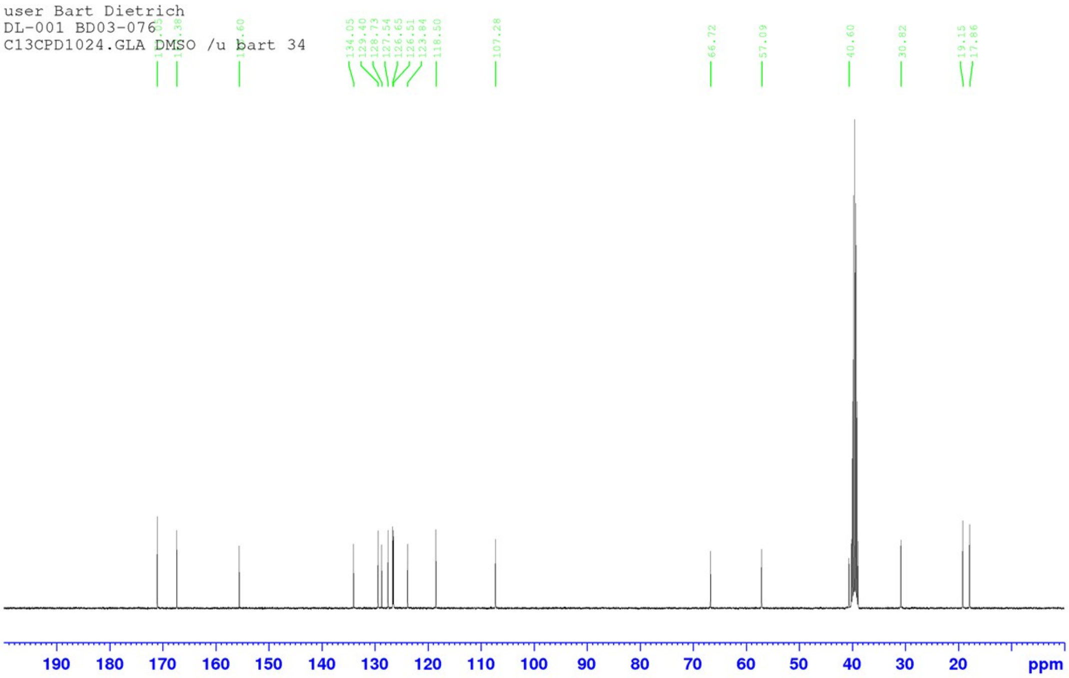

Figure 87. Carbon NMR of **DL-001**.

The D and the rac compound were prepared in a similar fashion.

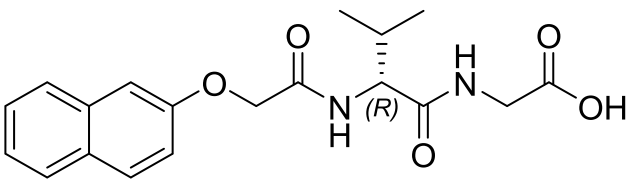

2NapVG(D)OH. ^{1}H NMR δ_H (400 MHz, DMSO-d$_6$) 12.60 (1H, br s, COOH), 8.44 (1H, t, *J* 5.80, NHCH$_2$), 8.02 (1H, d, *J* 9.04, NHCH*), 7.86-7.83 (2H, m, H$_{Ar}$), 7.75 (1H, d, *J* 8.12, H$_{Ar}$), 7.48-7.44 (1H, m, H$_{Ar}$), 7.38-7.34 (1H, m, H$_{Ar}$), 7.29-7.23 (2H, m, H$_{Ar}$), 4.75 (1H, d, *J* 14.61, OCH$_a$H$_b$), 4.70 (1H, d, *J* 14.61, OCH$_a$H$_b$), 4.32 (1H, dd, *J* 9.02, 6.50, CH*), 3.82 (1H, dd, *J* 17.45, 5.88, NHCH$_a$H$_b$), 3.74 (1H, dd, *J* 17.45, 5.84, NHCH$_a$H$_b$), 2.09-2.01 (1H, m, CH(CH$_3$)$_2$), 0.89 (3H, d, *J* 6.80, CH(CH$_3$)$_2$), 0.85 (3H, d, *J* 6.80, CH(CH$_3$)$_2$). ^{13}C NMR δ_C (100 MHz, DMSO-d$_6$) 171.03, 171.01, 167.36, 155.59, 134.03, 129.39, 128.71, 127.52, 126.63, 126.50, 123.83, 118.49, 107.27, 66.71, 57.08, 40.60, 30.80, 19.14, 17.84. HRMS (EI) m/z: [M]$^+$ calcd for $C_{19}H_{22}N_2O_5$ 358.1529; found 358.1521.

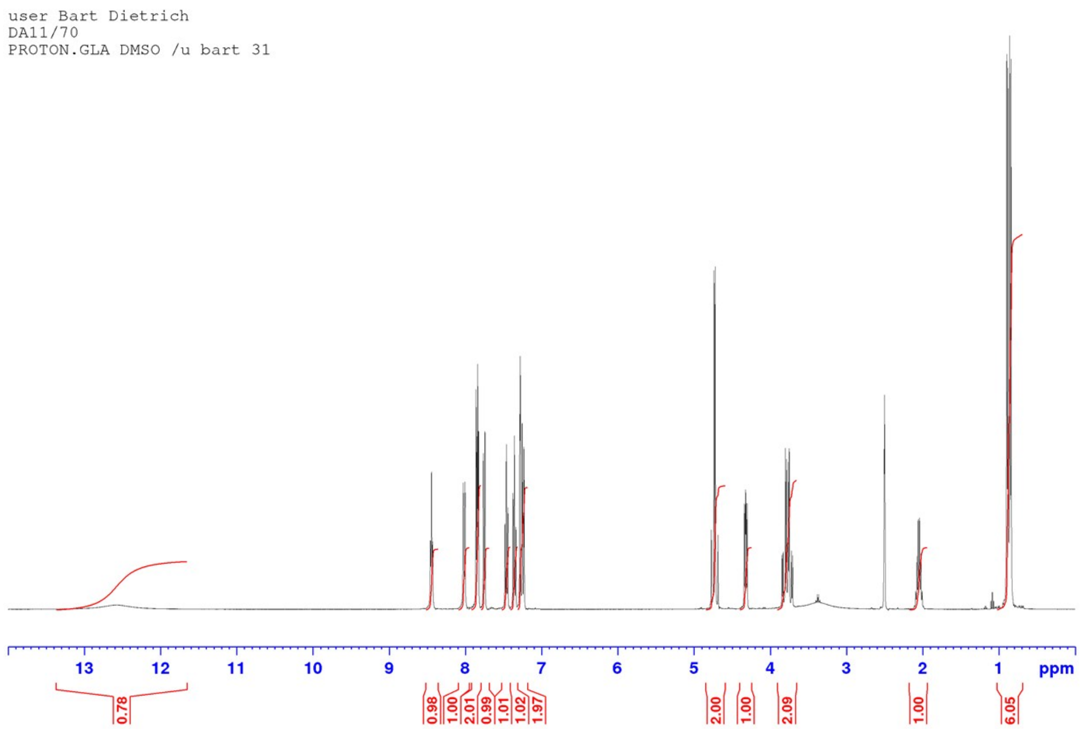

Figure 88. Proton NMR of 2NapVG(D)OH.

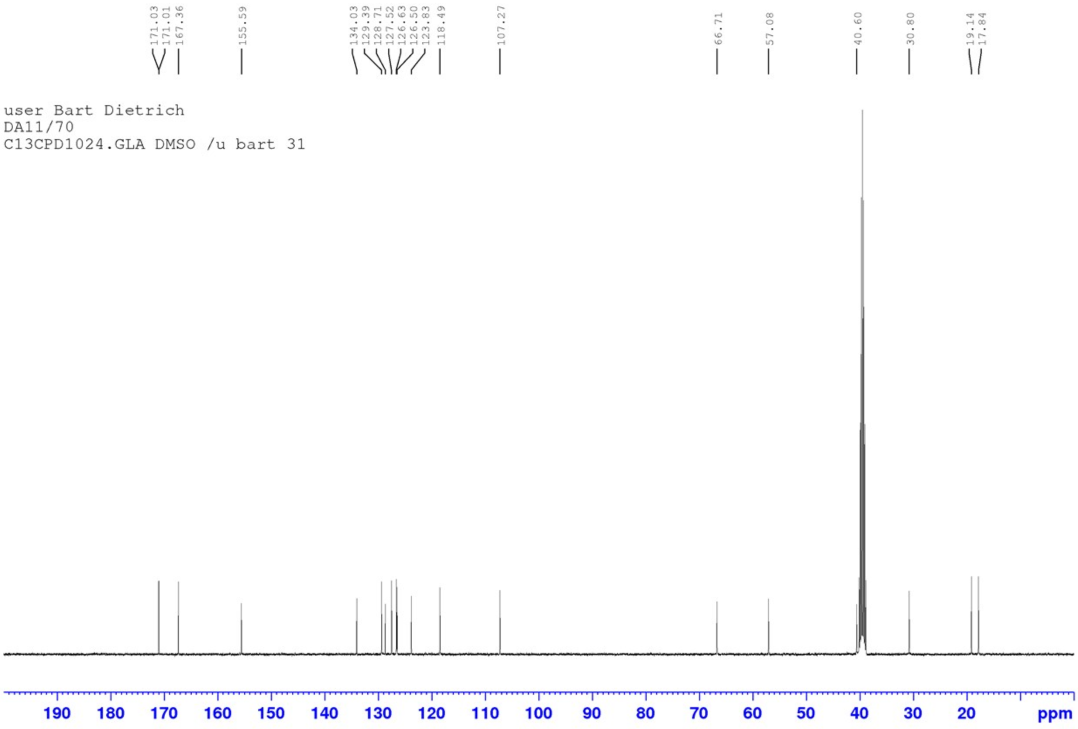

Figure 89. Carbon NMR of 2NapVG(D)OH.

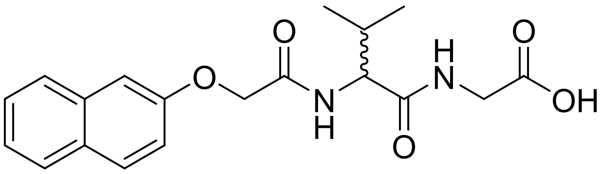

2NapVG(rac)OH. ^{1}H NMR δ_H (400 MHz, DMSO-d$_6$) 12.56 (1H, br s, COO<u>H</u>), 8.43 (1H, t, *J* 5.78, N<u>H</u>CH$_2$), 8.01 (1H, d, *J* 9.04, N<u>H</u>CH*), 7.86-7.83 (2H, m, <u>H</u>$_{Ar}$), 7.76-7.74 (1H, d, *J* 8.12, <u>H</u>$_{Ar}$), 7.46 (1H, ddd, *J* 8.08, 6.96, 1.14, <u>H</u>$_{Ar}$), 7.36 (1H, ddd, *J* 8.05, 6.93, 1.13, <u>H</u>$_{Ar}$), 7.28-7.22 (2H, m, <u>H</u>$_{Ar}$), 4.75 (1H, d, *J* 14.61, OC<u>H</u>$_a$H$_b$), 4.69 (1H, d, *J* 14.61, OCH$_a$<u>H</u>$_b$), 4.31 (1H, dd, *J* 9.00, 6.48, C<u>H</u>*), 3.81 (1H, dd, *J* 17.45, 5.88, NHC<u>H</u>$_a$H$_b$), 3.72 (1H, dd, *J* 17.45, 5.84, NHCH$_a$<u>H</u>$_b$), 2.08-2.00 (1H, m, C<u>H</u>(CH$_3$)$_2$), 0.88 (3H, d, *J* 6.76, CH(C<u>H</u>$_3$)$_2$), 0.84 (3H, d, *J* 6.80, CH(C<u>H</u>$_3$)$_2$). ^{13}C NMR δ_C (100 MHz, DMSO-d$_6$) 171.00, 167.33, 155.57, 134.02, 129.37, 128.70, 127.51, 126.62, 126.48, 123.81, 118.47, 107.26, 66.69, 57.05, 40.56, 30.79, 19.12, 17.83. HRMS (EI) m/z: [M]$^+$ calcd for $C_{19}H_{22}N_2O_5$ 358.1529; found 358.1547.

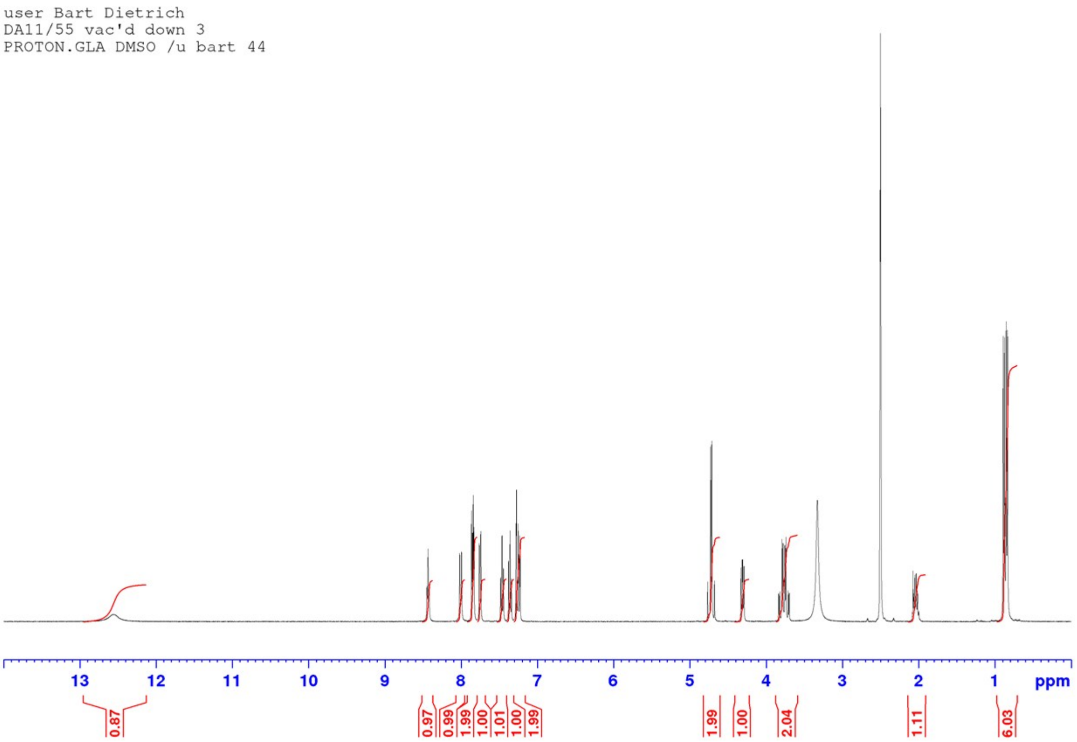

Figure 90. Proton NMR of 2NapVG(rac)OH.

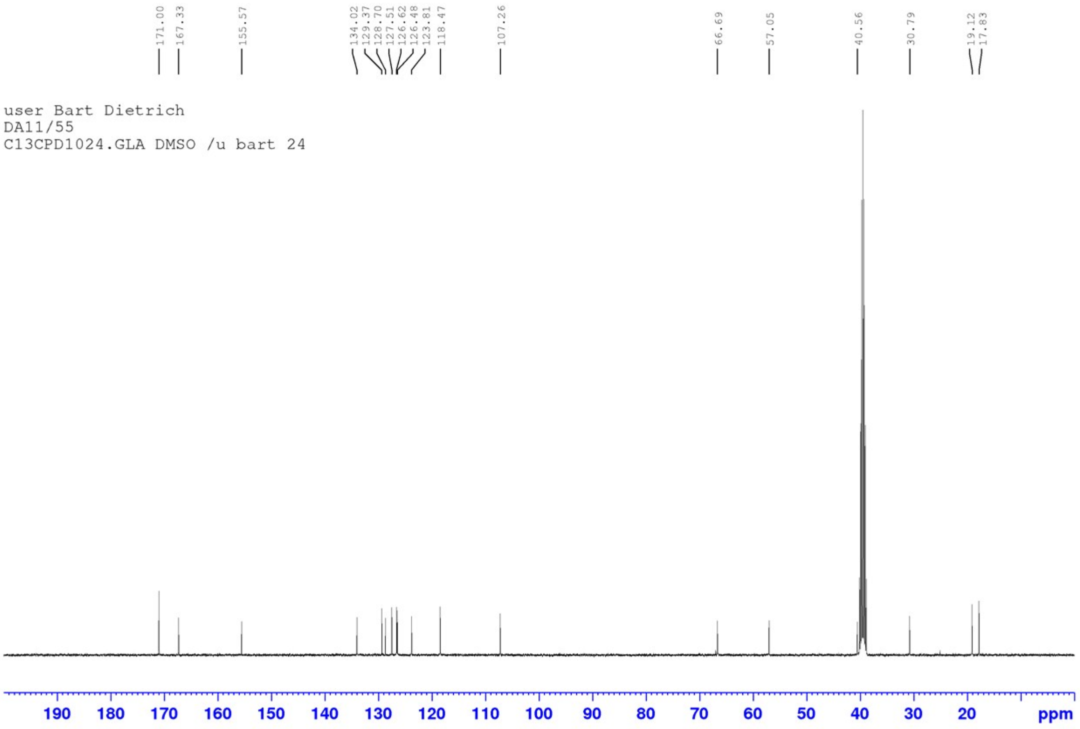

Figure 91. Carbon NMR of 2NapVG(rac)OH.

(2S)-2-{2-[(6-bromonaphthalen-2-yl)oxy]acetamido}propanoic acid

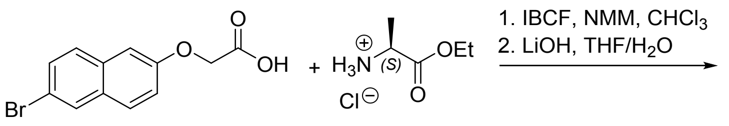

In a similar fashion to the above, 6-bromo-2-naphtoxyacetic acid was coupled to alanine ethyl ester (either the L-, the D-, or rac-), and deprotected using lithium hydroxide to give 6-Br-2NapAOH. This was then coupled to glycine ethyl ester, followed by deprotection using lithium hydroxide as described above.

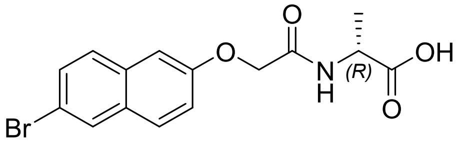

BrNapA(D)OH. ^{1}H NMR δ_H (400 MHz, DMSO-d_6) 12.68 (1H, br s, COOH), 8.45 (1H, d, *J* 7.48, NH), 8.13 (1H, d, *J* 1.80, H_{Ar}), 7.86 (1H, d, *J* 8.88, H_{Ar}), 7.75 (1H, d, *J* 8.80, H_{Ar}), 7.58 (1H, dd, *J* 8.74, 2.02, H_{Ar}), 7.34-7.30 (2H, m, H_{Ar}), 4.67 (1H, d, *J* 14.65, OCH_aH_b), 4.62 (1H, d, *J* 14.69, OCH_aH_b), 4.37-4.30 (1H, m, CH^*), 1.34 (3H, d, *J* 7.28, CH_3). ^{13}C NMR δ_C (100 MHz, DMSO-d_6) 173.80, 167.16, 156.01, 132.64, 129.89, 129.35, 129.29, 128.95, 128.58, 119.80, 116.54, 107.50, 66.77, 47.24, 17.06. HRMS (ESI) m/z: $[M+Na]^+$ calcd for $C_{15}H_{14}{}^{79}BrNNaO_4$ 373.9998; found 373.9983.

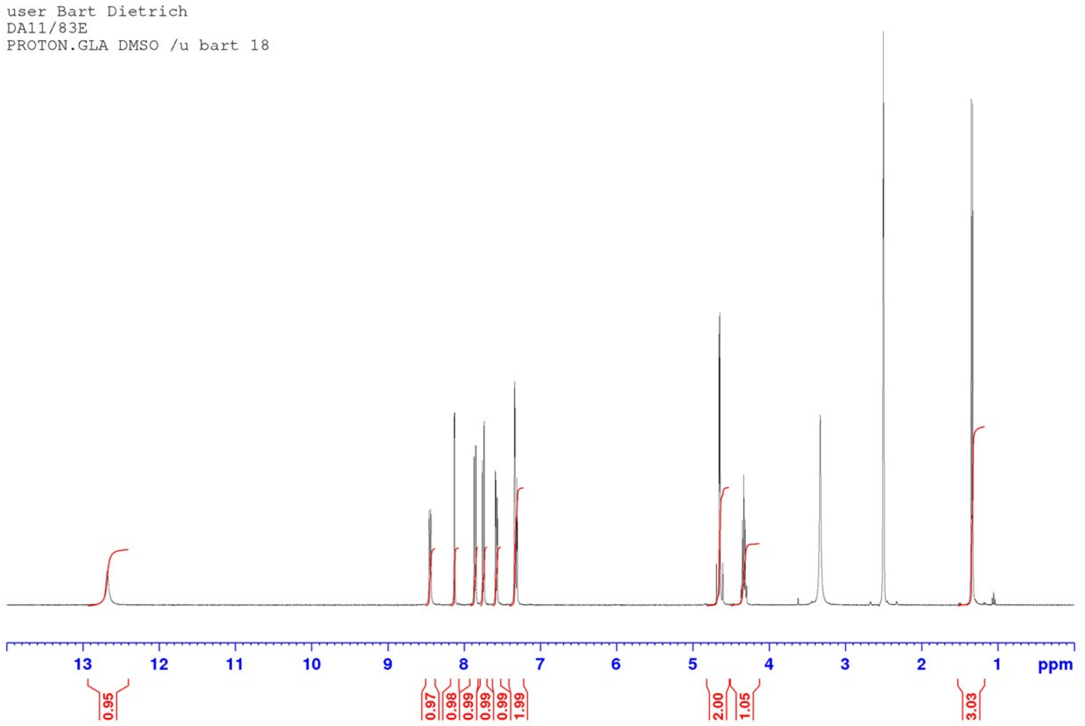

Figure 92. Proton NMR of BrNapA(D)OH.

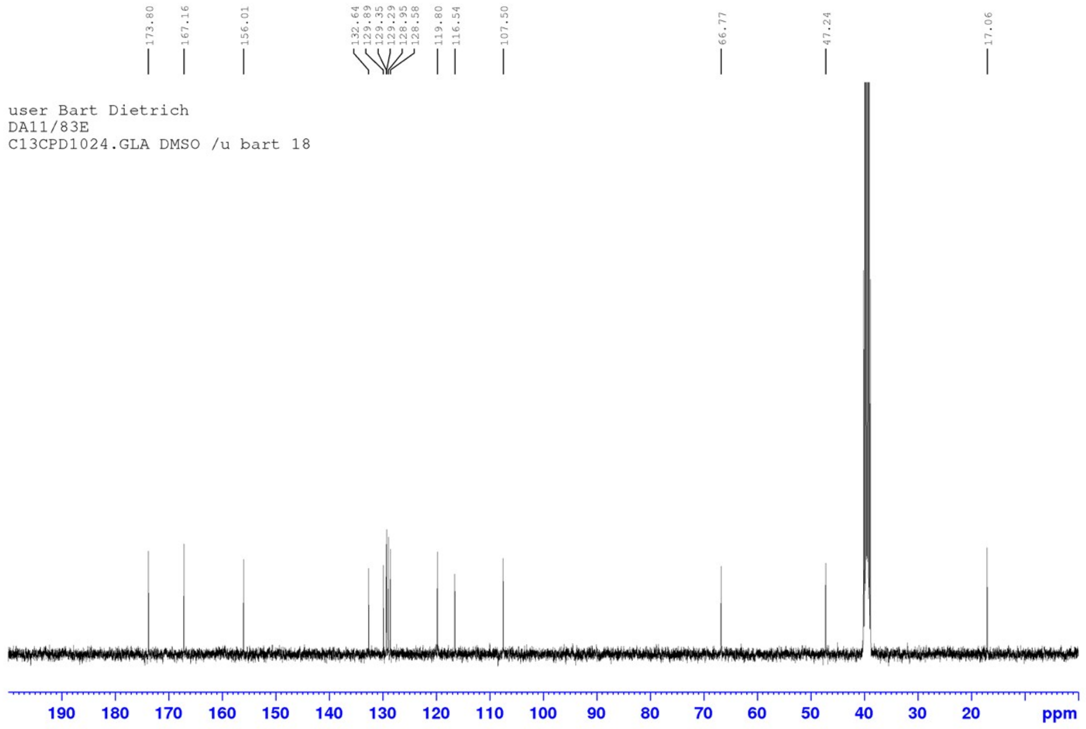

Figure 93. Carbon NMR of BrNapA(D)OH.

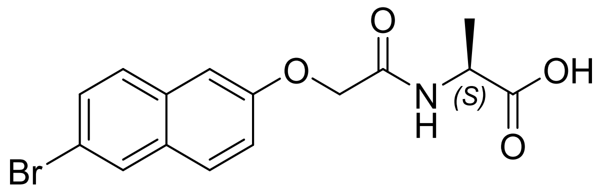

BrNapA(L)OH. ^{1}H NMR δ_H (400 MHz, DMSO-d$_6$) 12.68 (1H, br s, COO<u>H</u>), 8.45 (1H, d, *J* 7.52, N<u>H</u>), 8.12 (1H, d, *J* 1.80, $\underline{H}_{Ar}$), 7.86 (1H, d, *J* 8.88, $\underline{H}_{Ar}$), 7.75 (1H, d, *J* 8.80, $\underline{H}_{Ar}$), 7.58 (1H, dd, *J* 8.74, 2.02, $\underline{H}_{Ar}$), 7.34-7.30 (2H, m, $\underline{H}_{Ar}$), 4.67 (1H, d, *J* 14.69, $OC\underline{H}_aH_b$), 4.63 (1H, d, *J* 14.61, $OCH_a\underline{H}_b$), 4.37-4.30 (1H, m, $C\underline{H}^*$), 1.34 (3H, d, *J* 7.28, CH_3). ^{13}C NMR δ_C (100 MHz, DMSO-d$_6$) 173.81, 167.18, 156.01, 132.65, 129.89, 129.36, 129.30, 128.95, 128.58, 119.80, 116.55, 107.51, 66.78, 47.24, 17.07.

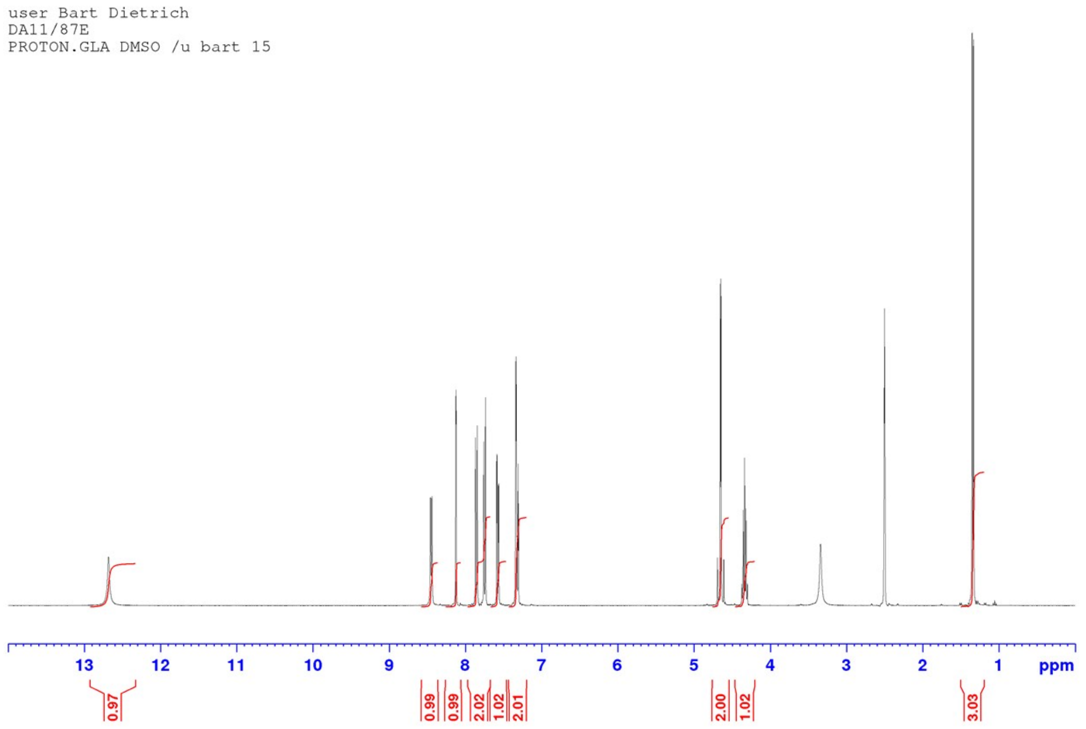

Figure 94. Proton NMR of BrNapA(L)OH.

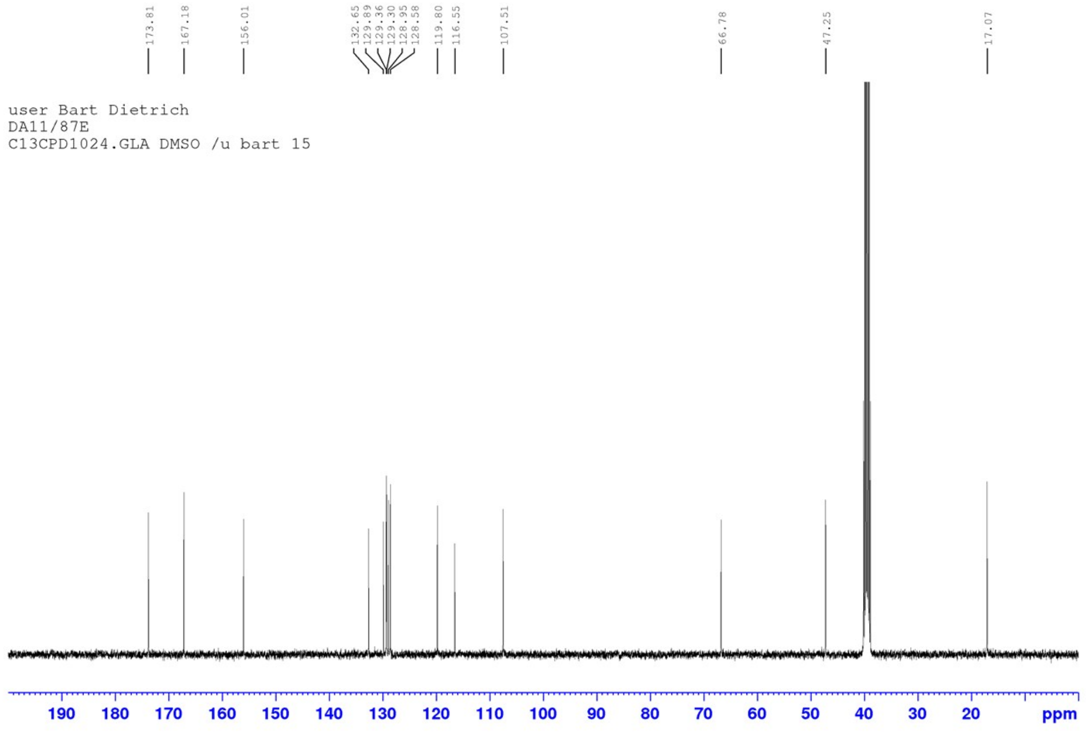

Figure 95. Carbon NMR of BrNapA(L)OH.

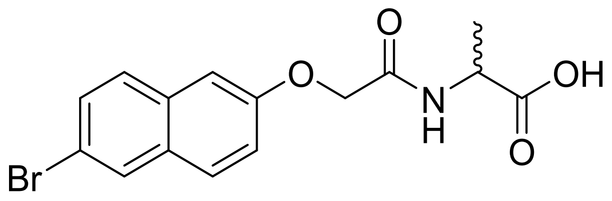

BrNapA(rac)OH. ^{1}H NMR δ_H (400 MHz, DMSO-d$_6$) 12.69 (1H, br s, COOH), 8.45 (1H, d, *J* 7.52, NH), 8.12 (1H, d, *J* 1.92, H_{Ar}), 7.85 (1H, d, *J* 8.92, H_{Ar}), 7.75 (1H, d, *J* 8.84, H_{Ar}), 7.57 (1H, dd, *J* 8.76, 2.04, H_{Ar}), 7.34-7.30 (2H, m, H_{Ar}), 4.68 (1H, d, *J* 14.65, OC$\underline{H}_a$H$_b$), 4.63 (1H, d, *J* 14.65, OCH$_a\underline{H}_b$), 4.38-4.30 (1H, m, CH*), 1.34 (3H, d, *J* 7.32, CH_3). ^{13}C NMR δ_C (100 MHz, DMSO-d$_6$) 173.82, 167.18, 156.02, 132.65, 129.90, 129.36, 129.30, 128.95, 128.59, 119.80, 116.56, 107.51, 66.78, 47.26, 17.08.

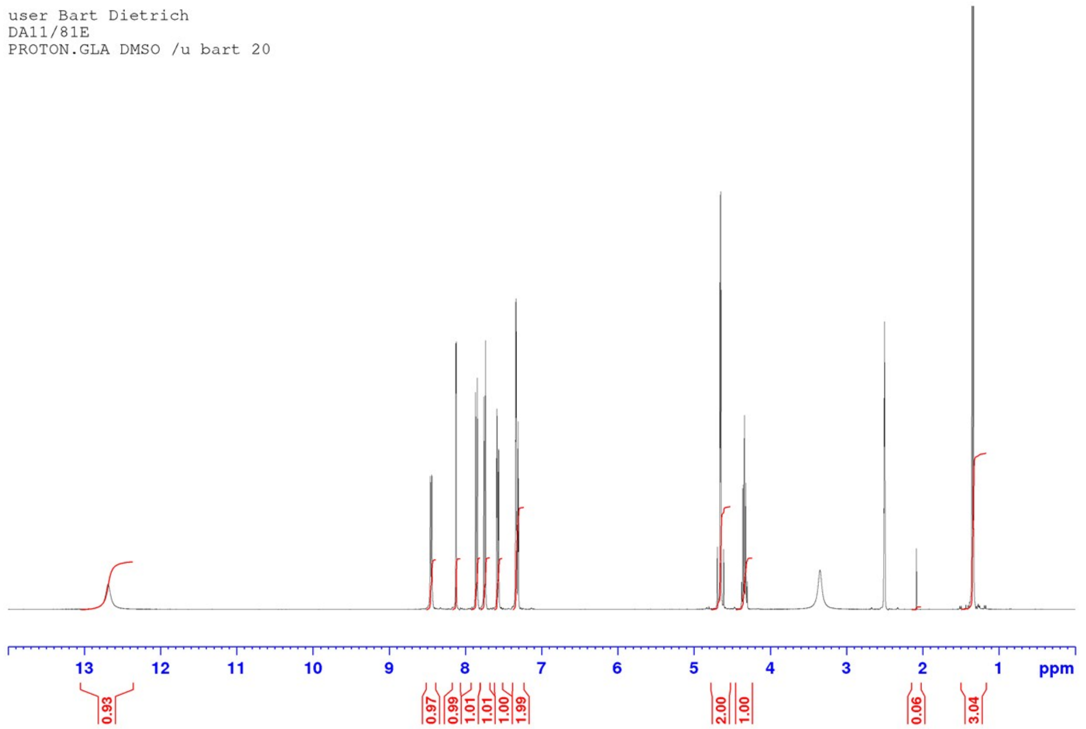

Figure 96. Proton NMR of BrNapA(rac)OH.

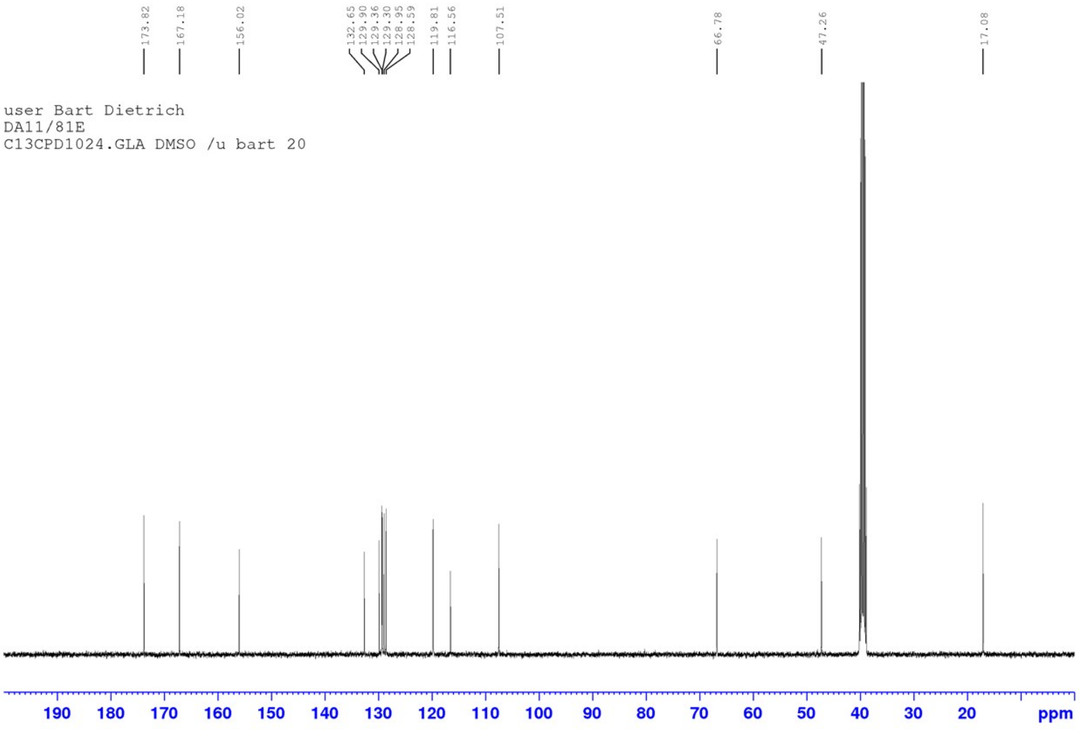

Figure 97. Carbon NMR of BrNapA(rac)OH.

2-[(2S)-2-{2-[(6-bromonaphthalen-2-yl)oxy]acetamido}propanamido]acetic acid

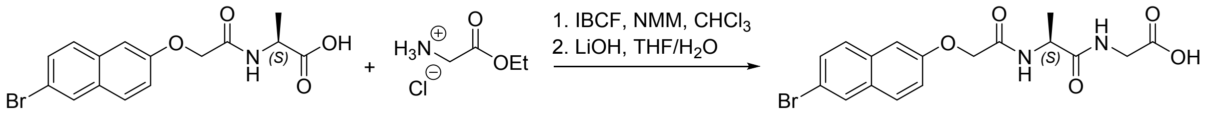

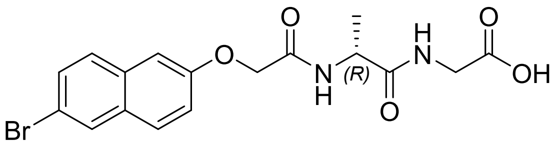

BrNapA(D)GOH. ^{1}H NMR δ_H (400 MHz, DMSO-d$_6$) 12.60 (1H, br s, COOH), 8.31 (1H, t, *J* 5.78, NHCH$_2$), 8.25 (1H, d, *J* 7.76, NHCH*), 8.13 (1H, d, *J* 1.64, H_{Ar}), 7.86 (1H, d, *J* 8.96, H_{Ar}), 7.76 (1H, d, *J* 8.84, H_{Ar}), 7.58 (1H, dd, *J* 8.76, 1.96, H_{Ar}), 7.34-7.30 (2H, m, H_{Ar}), 4.68 (1H, d, *J* 14.69, OCH_aH_b), 4.64 (1H, d, *J* 14.73, OCH_aH_b), 4.47-4.40 (1H, m, CH*), 3.79 (1H, dd, *J* 17.57, 5.96, NHCH_aH_b), 3.73 (1H, dd, *J* 17.63, 5.94, NHCH_aH_b), 1.29 (3H, d, *J* 7.08, CH$_3$). ^{13}C NMR δ_C (100 MHz, DMSO-d$_6$) 172.26, 171.04, 166.91, 155.99, 132.67, 129.89, 129.36,

129.32, 128.96, 128.64, 119.73, 116.56, 107.45, 66.79, 56.01, 47.68, 40.61, 18.54, 18.36. HRMS (ESI) m/z: $[M+Na]^+$ calcd for $C_{17}H_{17}BrN_2NaO_5$ 431.0213; found 431.0205.

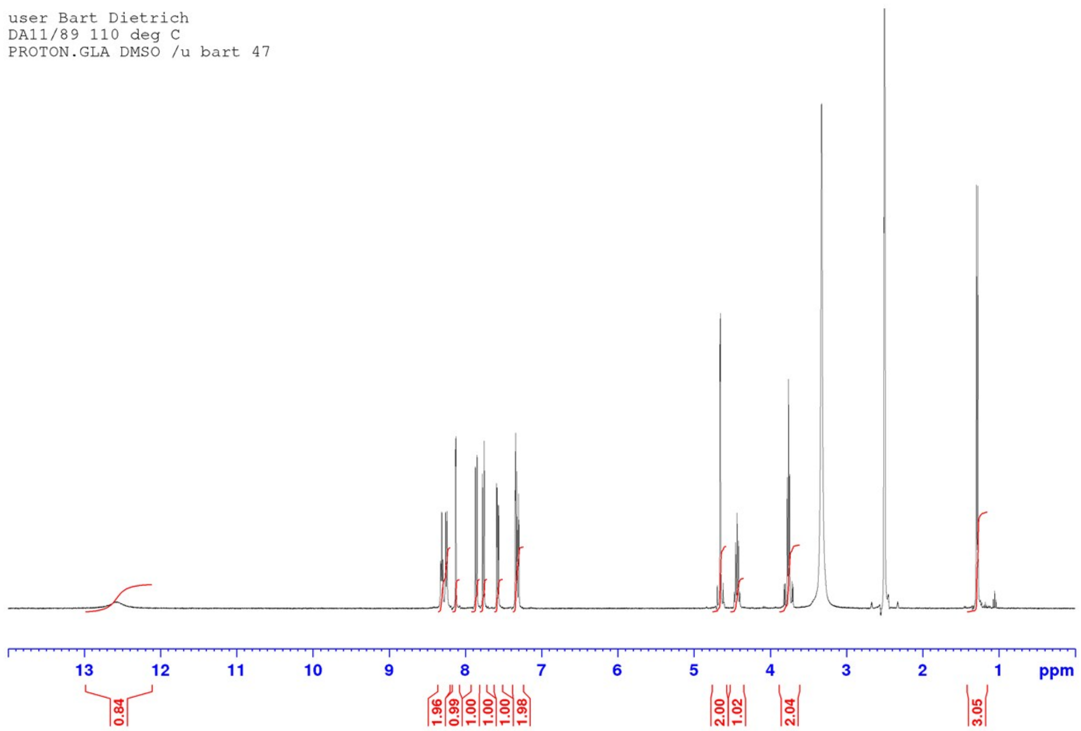

Figure 98. Proton NMR of BrNapA(D)GOH.

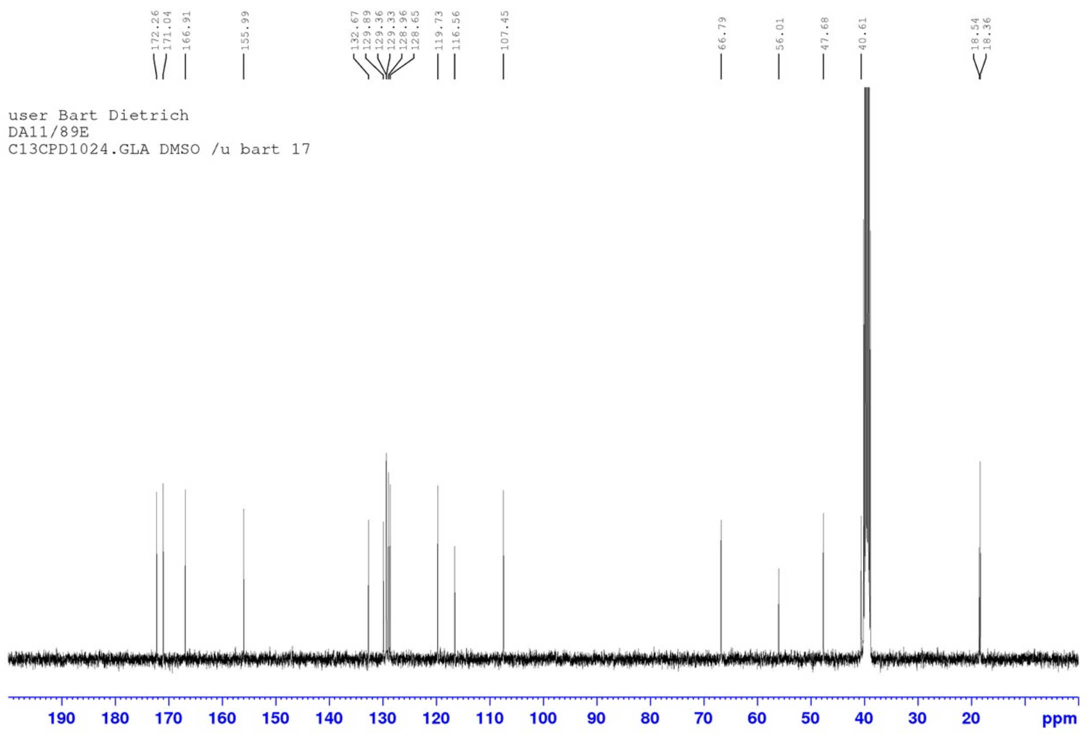

Figure 99. Carbon NMR of BrNapA(D)GOH.

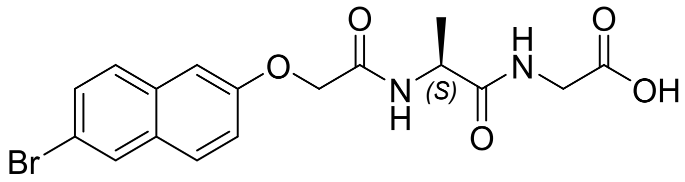

BrNapA(L)GOH. ^{1}H NMR δ_H (400 MHz, DMSO-d$_6$) 12.64 (1H, br s, COO<u>H</u>), 8.29 (1H, t, *J* 5.78, N<u>H</u>CH$_2$), 8.26 (1H, d, *J* 7.80, N<u>H</u>CH*), 8.13 (1H, d, *J* 1.88, <u>H</u>$_{Ar}$), 7.86 (1H, d, *J* 8.96, <u>H</u>$_{Ar}$), 7.76 (1H, d, *J* 8.84, <u>H</u>$_{Ar}$), 7.58 (1H, dd, *J* 8.76, 2.04, <u>H</u>$_{Ar}$), 7.35-7.30 (2H, m, <u>H</u>$_{Ar}$), 4.68 (1H, d, *J* 14.61, OC<u>H</u>$_a$H$_b$), 4.63 (1H, d, *J* 14.65, OCH$_a$<u>H</u>$_b$), 4.47-4.40 (1H, m, C<u>H</u>*), 3.78 (1H, dd, *J* 17.49, 5.84, NHC<u>H</u>$_a$H$_b$), 3.72 (1H, dd, *J* 17.49, 5.84, NHCH$_a$<u>H</u>$_b$), 1.28 (3H, d, *J* 7.12, CH$_3$). ^{13}C NMR δ_C (100 MHz, DMSO-d$_6$) 172.26, 171.05, 166.92, 156.00, 132.68, 129.90, 129.36, 129.33, 128.96, 128.65, 119.73, 116.56, 107.46, 66.80, 56.02, 47.69, 40.62, 18.54, 18.37. HRMS (ESI) m/z: [M+Na]$^+$ calcd for $C_{17}H_{17}{}^{79}BrN_2NaO_5$ 431.0213; found 431.0208.

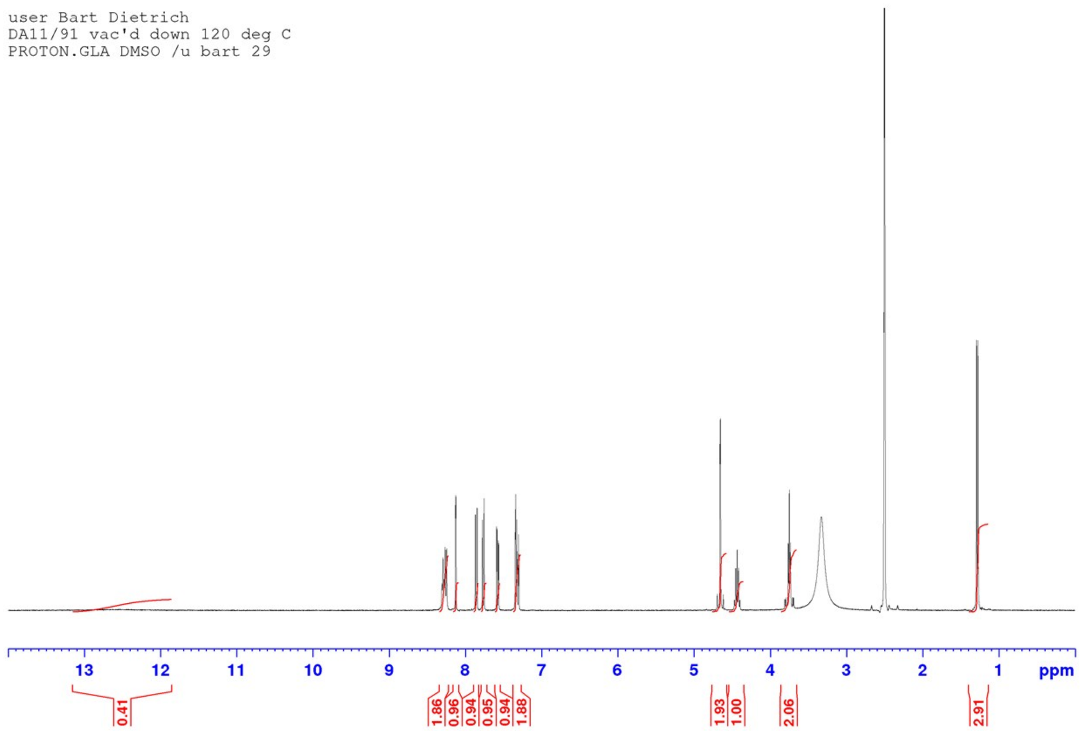

Figure 100. Proton NMR of BrNapA(L)GOH.

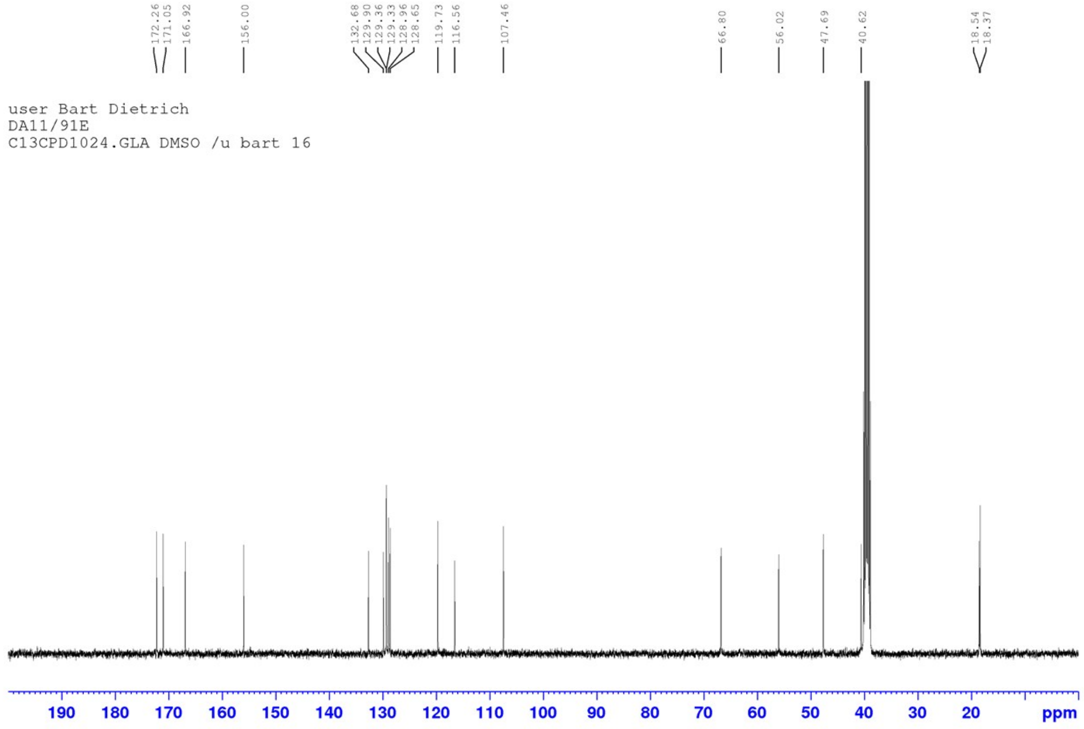

Figure 101. Carbon NMR of BrNapA(L)GOH.

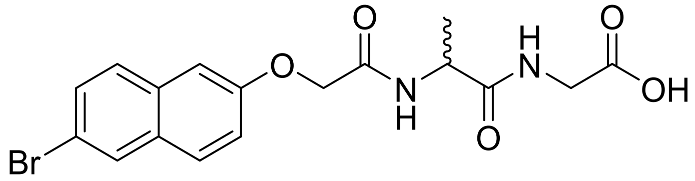

BrNapA(rac)GOH. ^{1}H NMR δ_H (400 MHz, DMSO-d_6) 12.59 (1H, br s, COO<u>H</u>), 8.31 (1H, t, *J* 5.86, N<u>H</u>CH_2), 8.25 (1H, d, *J* 7.80, N<u>H</u>CH*), 8.13 (1H, d, *J* 1.88, $\underline{H}_{Ar}$), 7.86 (1H, d, *J* 8.96, $\underline{H}_{Ar}$), 7.76 (1H, d, *J* 8.84, $\underline{H}_{Ar}$), 7.58 (1H, dd, *J* 8.76, 2.04, $\underline{H}_{Ar}$), 7.35-7.30 (2H, m, $\underline{H}_{Ar}$), 4.68 (1H, d, *J* 14.65, $OC\underline{H}_aH_b$), 4.64 (1H, d, *J* 14.61, $OCH_a\underline{H}_b$), 4.47-4.40 (1H, m, C<u>H</u>*), 3.79 (1H, dd, *J* 17.59, 6.02, $NHC\underline{H}_aH_b$), 3.73 (1H, dd, *J* 17.61, 5.96, $NHCH_a\underline{H}_b$), 1.29 (3H, d, *J* 7.08, $C\underline{H}_3$). ^{13}C NMR δ_C (100 MHz, DMSO-d_6) 172.27, 171.05, 166.93, 156.00, 132.68, 129.90, 129.37, 129.33, 128.97, 128.65, 119.73, 116.57, 107.46, 66.80, 47.69, 40.62, 18.37. HRMS (ESI) m/z: $[M+Na]^+$ calcd for $C_{17}H_{17}BrN_2NaO_5$ 431.0213; found 431.0197.

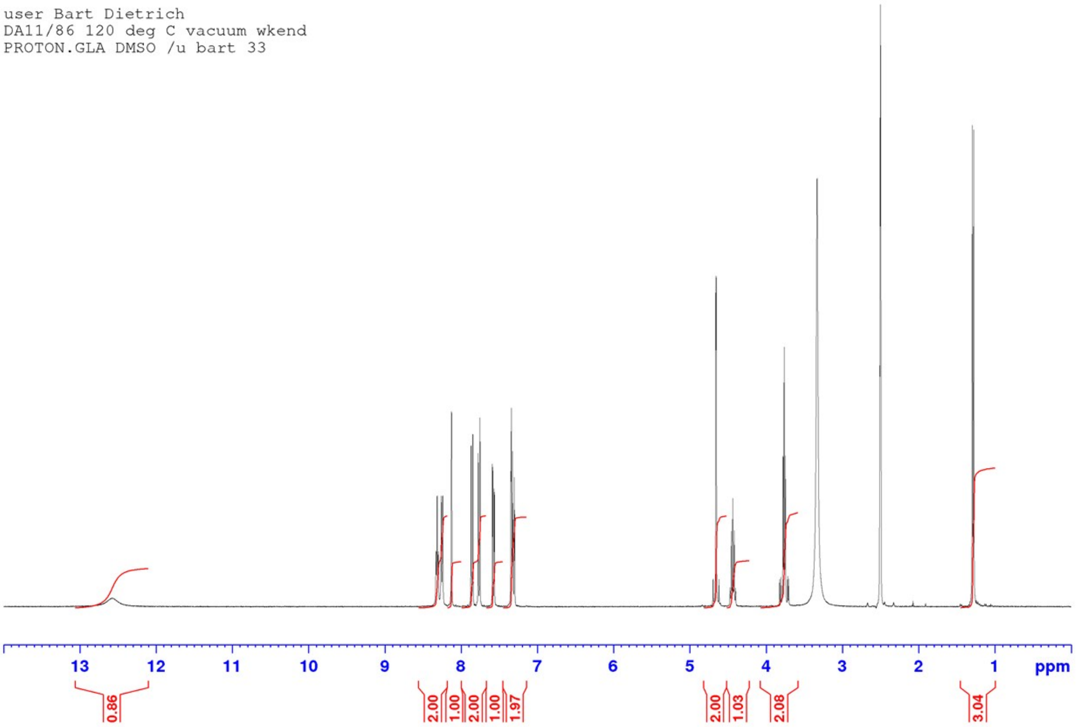

Figure 102. Proton NMR of BrNapA(rac)GOH.

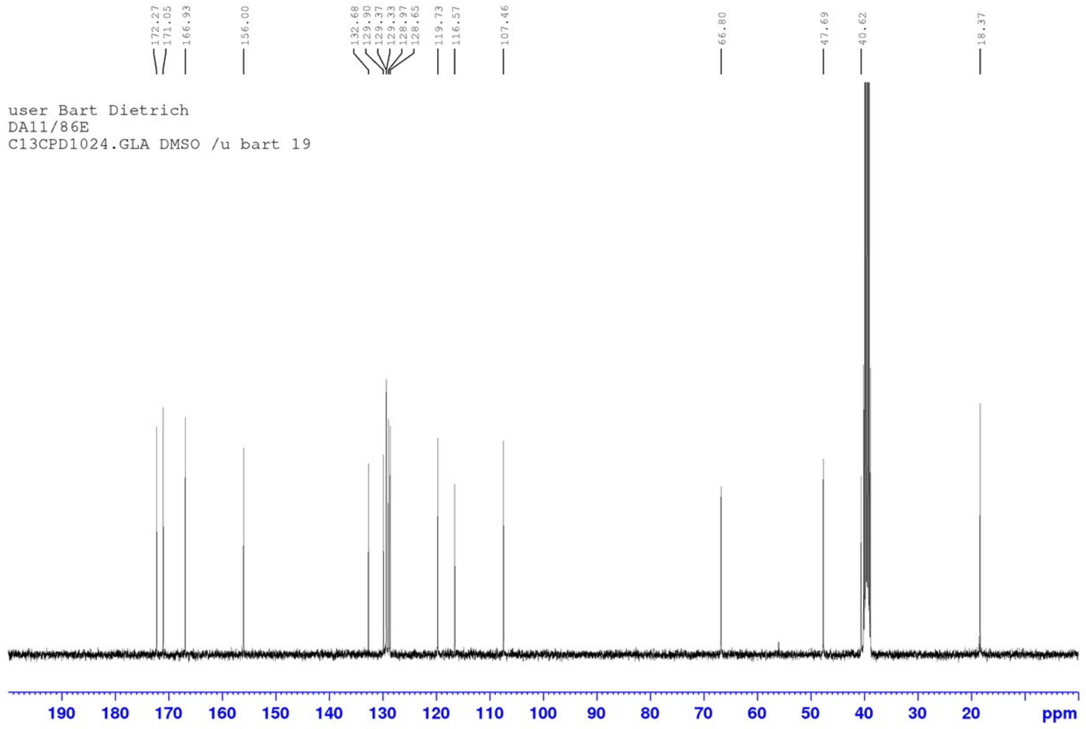

Figure 103. Carbon NMR of BrNapA(rac)GOH.

References

1. K. L. Morris, L. Chen, A. Rodger, D. J. Adams and L. C. Serpell, *Soft Matter*, 2015, **11**, 1174-1181.
2. P. Terech, J. J. Allegraud and C. M. Garner, *Langmuir*, 1998, **14**, 3991-3998.
3. W. Zhou, M. F. Islam, H. Wang, D. L. Ho, A. G. Yodh, K. I. Winey and J. E. Fischer, *Chem. Phys. Lett.*, 2004, **384**, 185-189.
4. P. Terech, G. Clavier, H. Bouas-Laurent, J.-P. Desvergne, B. Demé and J.-L. Pozzo, *J. Coll. Inter. Sci.*, 2006, **302**, 633-642.
5. L. Chen, T. O. McDonald and D. J. Adams, *RSC Adv.*, 2013, **3**, 8714-8720.
6. L. Chen, S. Revel, K. Morris, L. C. Serpell and D. J. Adams, *Langmuir*, 2010, **26**, 13466-13471.
